# Supplementary material for: “If we lose it, we are worried”: Individual and provider level perceptions towards weight change among people living with HIV who undergo TB screening in routine health care settings in Gauteng Province, South Africa
Source: PLoS One. 2025 Sep 22;20(9):e0331904. doi: 10.1371/journal.pone.0331904 (PMC12453174; doi:10.1371/journal.pone.0331904)
Supplement: S1 File — (DOCX) [file pone.0331904.s001.docx]

| FGD topic guide section A:Perceptions around weight change |
| --- |

| ***Explain the following to the participants:***  *“If a patient comes to a clinic the clinic nurse or doctor may ask about the patient’s weight”* |
| --- |

***Icebreaker to initiate interaction: Participants & researchers introduce themselves to one another/ Name of a support group/social or employment status (researchers to re-enforce the objective of the study as it appears on a consent form)***

1. When you attend a clinic for your appointments, do the clinic staff (clinic nurse / clinic doctor) ask you about your weight or if you have lost weight?
   - *Why do you think they ask this?*
2. When a person living with HIV is weighed or asked about their weight, how do you think they may feel *(use visuals)*:
   - *Before they are weighed*
   - *During the weighing*
   - *After they are weighed*
   - *If they have lost weight*
   - *If they have gained weight*
3. If a person living with HIV is asked about weight loss by clinic staff what would make them say they have lost weight? If a person living with HIV says they have lost weight, what do you think this means?
   - *Why will a person living with HIV report they have lost weight when asked by clinic staff?*
     - *e.g. change in dress size, feel weak, they don’t have appetite, they have been feeling sick, they feel stressed, they don’t think their treatment is going well*
   - *Is there stigma attached to PLWHIV losing weight? Do others gossip about them? Do they call them names? What names? How would this make PLHIV feel? (stigma)*
4. If a person is attending a clinic for HIV care, how do you think their weight should be? And how about the shape?

Probes:

- *Use visuals (Stunkard silhouettes)*
- *What changes might happen to a person’s weight? And what about the shape?*
- *If treatment is going well compared with not going well?*
- *What about ideal weight and ideal shape? Why? Men versus women?*
- *What about most attractive weight and shape? Why? Men versus women?*
  - *Is this influenced by the community you stay in?*
  - *Is it influenced by your culture?*
  - *Is it influenced by your family?*
  - *Is it influenced by health services?*
  - *Is it influenced by media?*

1. Why do you think people living with HIV may actually lose weight?
2. Why do you think people living with HIV report their weight has changed but clinic weighing scales show no change?

*Probes:*

- - *PLHIV reports weight loss but scales don’t confirm this, why do you think this happens?*
  - *PLHIV reports weight gain but scales don’t confirm this, why do you think this happens?*
    1. *What do you think gaining weight means to a patient attending for HIV care?*

1. In your experience, what is the best way to ask people living with HIV about losing and gaining weight?

- If someone asks you “Have you lost ***more than*** a dress/trouser size ***unintentionally*** in the last 6 months?” do we understand that question? What does it mean?
- *Use visuals of dress / trouser sizes, and ask about dropping e.g. size 36 to 32, or gaining e.g. size 32 to 36. Ask participants to demonstrate using the visuals.*

| FGD topic guide section B:Experiences of weight and shape changes |
| --- |

1. How do you think the following will respond to a person living with HIV who has lost weight? *(Probe for stigma-gossip, shaming, blame, name calling, distancing)*
   - *Spouse*
   - *Children*
   - *Wider/extended family*
   - *Friends*
   - *Community*
   - *Health care workers (doctors, nurses, counsellors at the clinic)*
2. How do you think a person living with HIV will feel about the above responses?
3. How do you think the following will respond to a person living with HIV who has gained weight? *(Probe for stigma)*
   - *Spouse*
   - *Children*
   - *Wider/extended family*
   - *Friends*
   - *Community*
   - *Health care workers (doctors, nurses, counsellors at the clinic)*
4. How do you think a person living with HIV will feel about the above responses?
5. In general do you think body shape changes in people living with HIV?

Probes:

- - *Can you describe how? Men versus women?*
  - *Why do you think these changes occur?*
    1. *That which is caused by HIV itself / symptom of HIV. What is the disease doing? Feelings related to this.*
    2. *That which is caused by HIV treatment / ARVs in the body. What is the treatment doing? Feelings related to this.*
    3. *Is it related to PLHIV eating more or less (stress of not having the food they need / being able to access what they need)?*
  - *Probe regarding lipodystrophy (fat gain in abdomen & neck; fat loss face / buttocks /arms / legs)*

1. How do you think the following will respond to a person living with HIV whose body shape has changed? *(Probe for stigma)*
   - *Spouse*
   - *Children*
   - *Wider/extended family*
   - *Friends*
   - *Community*
   - *Health care workers (doctors, nurses, counsellors at the clinic)*
2. *How do you think a person living with HIV will feel about the above responses?*

| ***Concluding remarks:***  *“We have come to the end of this discussion. Thank you very much for participating and for your time.”*  *Interview instructions: Please ensure reimbursement is provided to participant.* |
| --- |
