# Supplementary material for: “If we lose it, we are worried”: Individual and provider level perceptions towards weight change among people living with HIV who undergo TB screening in routine health care settings in Gauteng Province, South Africa
Source: PLoS One. 2025 Sep 22;20(9):e0331904. doi: 10.1371/journal.pone.0331904 (PMC12453174; doi:10.1371/journal.pone.0331904)
Supplement: S2 File — (DOCX) [file pone.0331904.s002.docx]

| FGD topic guide section A:Perceptions around weight change |
| --- |

***Icebreaker to initiate interaction: Participants & researchers introduce themselves to one another (researchers to reinforce the objective of the study as it appears on a consent form)***

| ***Explain the following to the participants:***  *“If an HIV-positive patient comes to a clinic a health care worker may ask about his/her weight”* |
| --- |

1. When an HIV-positive patient attends for an appointment, do you ask about his/her weight or if s/he has lost weight?
   - *Why do you ask this?*
   - *In what circumstances are you more likely to ask / less likely to ask? (e.g. if patient looks thin or ill, time pressure)*
2. Do HIV-positive patients voluntarily consult you because they are losing weight?
3. If an HIV-positive patient reports they are losing weight, what would you usually do?
   - *Why would you do this?*
   - *What questions do you ask?*
     1. *Other symptoms (TB, diabetes, thyroid, cancer, oesophageal candidiasis, diarrhoea, side effects)*
     2. *Personal circumstances (diet, finances, stress, acceptance of diagnosis)*
     3. *Lifestyle (exercising, healthy living, alcohol, condoms)*
   - *What tests might you arrange? In what circumstances would you arrange tests?*
     1. *Are tests arranged only if patient is “sick” or reports cough or other symptoms?*
     2. *Sputum tests*
        1. *What if patient cannot produce sputum?*
     3. *Chest x-ray or ultrasound scan (sonar)*
        1. *In what circumstances would you arrange these?*
     4. *Blood tests*
        1. *In what circumstances would you arrange these?*
   - *What referrals might you arrange? (e.g. to nurse, doctor, dietician, social worker, pharmacist, NGO)*
     1. *In what circumstances would you arrange referrals?*
   - *What recommendations or treatment might you advise? (vitamin supplements, diet changes)*
   - *Are you able to arrange* ***follow up****? What* follow up *might you arrange? In what circumstances would arrange follow up?*
4. Why do you think HIV-positive patients may ***actually*** lose weight?
   - Illness, socioeconomic factors, stress
5. Why do you think HIV-positive patients may ***report*** weight loss?
   - *e.g. change in dress size, feel weak, they don’t have appetite, they have been feeling sick, they feel stressed, they don’t think their treatment is going well, “in the mind”, not accepting diagnosis, not complying with treatment.*
   - *Interpreting body shape changes, e.g. thin face and legs (lipodystrophy) as weight loss*
   - *Is there stigma attached to losing weight? Do others gossip about them? Do they call them names? What names? What have your patients shared with you about how this makes them feel?*
6. If a person is attending a clinic for HIV care, how do you think their weight should be? And how about the shape?

Probes:

- *Use visuals (Stunkard silhouettes)*
- *What changes might happen to a person’s weight? And what about the shape?*
- *If treatment is going well compared with not going well?*
- *What about ideal weight and ideal shape? Why? Men versus women?*
- *What about most attractive weight and shape? Why? Men versus women?*
  - *Is this influenced by the community they stay in?*
  - *Is it influenced by culture?*
  - *Is it influenced by family?*
  - *Is it influenced by health services?*
  - *Is it influenced by media?*

1. Why do you think HIV-positive patients report their weight has changed but clinic weighing scales show no change?

*Probes (feelings / meanings that patients have shared with health care worker):*

- - *Patient reports weight loss but scales don’t confirm this, why do you think this happens?*
    1. *What do you think losing weight means to a patient attending for HIV care?*
  - *Patient reports weight gain but scales don’t confirm this, why do you think this happens?*
    1. *What do you think gaining weight means to a patient attending for HIV care?*

1. In your experience, what is the best way to ask people living with HIV about losing and gaining weight?

- How do you usually ask about weight loss?
- Do you think patients will understand the following question “Have you lost ***more than*** a dress/trouser size ***unintentionally*** in the last 6 months?”
  - What does this question mean?
  - Do you think patients understand “***unintentiona***l”?
    - What alternative would you suggest?
- *Use visuals of dress / trouser sizes, and ask about dropping e.g. size 36 to 32, or gaining e.g. size 32 to 36. Ask participants to demonstrate using the visuals.*

| FGD topic guide section B:Perceptions around body shape changes |
| --- |

1. When an HIV-positive patient attends for an appointment, do you ask about changes in his/her body shape?
   - *Why do you ask this?*
   - *In what circumstances are you more likely to ask / less likely to ask?*
   - *How do patients usually respond?*
2. If an HIV-positive patient consults you because of changes in the shape of his/her body, what would you usually do?
   - *Why would you do this?*
   - *What recommendations / treatment / referrals might you arrange? (e.g. to nurse, doctor, dietician, social worker, pharmacist)*
3. In general do you think body shape changes in people living with HIV?

Probes:

- - *Can you describe how? Men versus women?*
  - *Why do you think these changes occur?*
    1. *That which is caused by HIV itself / symptom of HIV. What is the disease doing? What feelings have your patients shared with you about these changes.*
    2. *That which is caused by HIV treatment / ARVs in the body. What is the treatment doing? What feelings have your patients shared with you about these changes.*
    3. *Is it related to PLHIV eating more or less (stress of not having the food they need / being able to access what they need)?*
  - *Probe regarding lipodystrophy (fat gain in abdomen & neck; fat loss face / buttocks /arms / legs)*

1. Have your patients shared with you reactions from the following (stigma in particular) in response to their body shape changes? *(Probe for stigma)*
   - *Spouse*
   - *Children*
   - *Wider/extended family*
   - *Friends*
   - *Community*
   - *Health care workers (doctors, nurses, counsellors at the clinic)*

What have your patients shared with you about how this *(stigma)* makes them feel?

| ***Concluding remarks:***  *“We have come to the end of this discussion. Thank you very much for participating and for your time.”*  *Interview instructions: Please ensure reimbursement is provided to participant.* |
| --- |
