# Supplementary material for: “If we lose it, we are worried”: Individual and provider level perceptions towards weight change among people living with HIV who undergo TB screening in routine health care settings in Gauteng Province, South Africa
Source: PLoS One. 2025 Sep 22;20(9):e0331904. doi: 10.1371/journal.pone.0331904 (PMC12453174; doi:10.1371/journal.pone.0331904)
Supplement: S4 File — (ZIP) [file pone.0331904.s004.zip › S4 Transcripts_final/FGD 1.docx]

FGD 1

- **...** Ellipses indicate talk omitted from the data segment
- **(( ))** The transcriber’s comments.
- **( )** Empty parentheses indicate some talk was not audible or interpretable at all (we include the line for instance 20:15)
- **(.)** A dot enclosed in parenthesis indicate a short silence.
- **[ ]** Square brackets indicating beginning and the end of overlapping speech.

A focus group discussion starts.

M2: Okay please speak a bit louder for our tape recorder so that when we get home, we will hear what you were saying. When you arrive at the clinic for your appointment with the doctors and the nurses, do they ask you about your weight, if you have lost or gained weight? Do they ask?

P: They did not ask me.

M: Okay.

P: No, they did not ask me either.

P: No, they did not ask, until I explained that I sometimes feel as if I am losing weight.

P: No, they did ask me how I feel.

P: They ask me.

M2: They ask you.

P: No, they have not yet asked.

M2: Why do they ask you about the weight?

P: Yes, my sister I think that that is when they want to check the amount of weight that you have either lost or gained. You see, maybe that which you can feel it on your waist, yes.

P: Do you check your weight; I do check mine.

M: Oh you have a tendency of checking it out?

P: Uhum I do check myself because they sometimes ask what your weight is and if you are okay.

M: So in other words they ask if you have gained or lost weight.

P: I have never been asked.

M2: You have never been asked.

P: They used to ask me when I get here.

M: Do they weigh you though?

P: Even though I have not been to the scale as yet.

M: You have not started taking ARVs yet?

Ps: No.

M: Okay. How many of you amongst us here have not started taking ARVs?

Ps: All of us.

P: All of us.

M: All of us have not started taking them, what is the name of the support group that you are in?

P: I do not belong to a support group.

P: Neither do I.

M: What were you meeting for earlier on?

P: It’s the ARVs class.

M: The ARV initiation class, right? Okay, we wanted to do an icebreaker right, we would like to get to know each other, who you are, where you work and stuff like that, we have already introduced ourselves as researchers, okay?

P: Yes.

M: Yes, maybe before we continue maybe for the benefit of the whole group, if perhaps my brother can tell us who he is, if he is working or not, stuff like that. Let us start with number 1the, the numbers that we allocate to you are 1, 2, 3, 4, 5, 6 and if perhaps you could recall every time, you talk you do not mention your number but your number, my brother can you perhaps start by telling us who you are and if you are working.

P001: I am xxxx (position).

M: Okay, we are happy to be with you today. Yes number 2?

P002: I am xxx (position).

M: Okay, number 3?

P003: I am working as xxxxx (position).

M: You work as an xxx (position), yes gentleman number 4?

P004: I am xxx (position).

M: You are xxxx (position).

P004: xxxxxx.

M: Number 5?

P005: Is xxxx (position), xxxxxx.

M: Okay, number 6?

P006: Number 6 is xxx (position).

M: Okay, I like what number 6 says because he says he is xxxx (position). Okay, thank you for that, we are just researchers, we are happy to be with you ((M asks M2 to continue)).

M2: A person who has HIV, if he arrives at the clinic, how does he feel?

P006: I think if you had lost weight, you will be afraid that everyone will see that I am not well, you will feel sad.

M: What is it number 6 that actually makes you to be concerned if you lost weight?

P006: If you lost weight.

M: What exactly makes you to feel concerned if you lost weight?

P006: What brings in the worry is that I start having questions like maybe I am about to get sick, I will start having piles, you see.

M: Uhm, what do others say?

P005: I was once worried, my -CD4 count was low, I was very scared that hey I am about to die, the other day as I was sleeping the bed changed the direction. I do get worried when I lose weight, I was very worried. You see when you are relaxed and you think about one and the same thing but sometimes I just tell myself that it’s life.

M: Okay, all right, what do others say?

P: Losing weight is not a good thing since once you start losing weight everyone notices, you see that type of a thing, you become a problem, even when you are sick, it’s better to look good and have energy, you recover soon but if you will lose weight and have not strength, it’s a problem, it actually is a problem.

P: I was once, ei, my results came back saying that I am positive and my CD4 count was 73.

P001: It’s painful to lose weight not knowing what you are suffering from whereas if you lose weight and you know what you are suffering from, you will get help, support from other people whereas if you do not know, what could my brother be suffering from, what is uncle suffering from? In that instance, it’s very painful.

P: I agree with number 1, if you do not know what you are suffering from, your stress levels go up, when you have a lot of stress you lose weight but if you know what you are suffering from, it’s easy to tell yourself that actually you will get assistance, yes.

M: Uhum, how many of us were weighed when we got here, you said they weighed you, isn’t it?

P006: Yes, at my local clinic, not at this one.

P: They once weighed me.

M: So number 6 was once weighed.

P: I have never been weighed.

P: We have been weighed someplace else and not here.

M: But you were once weighed at different clinics before?

Ps: Yes.

M: Because we would like to know how you felt before you were weighed, how did you feel before you stood on a weighing scale?

P006: Okay I was afraid that eish since I have been diagnosed with HIV it means I have lost weight so I was nervous that ei perhaps I have lost weight.

M: Was that before you stood there on a weighing scale?

P006: Before standing on the scale, I was afraid that ei they are about to weigh me now and it’s possible that I may have lost weight.

M: Okay, number 2?

P005: No, number 5, I was frightened when I received the result, when I proceeded to the weighing scale I was convinced that I will get bad results, I were to receive unpleasant results.

M: So how did you feel?

P005: I was frustrated but I had to accept it because I get support and also the fact that I am in this situation, I am able to continue living my life.

P004: I was once weighed when I visited the hospital, I was in town accompanied by my partner, I then said my brother I do not understand my weight, kindly give me 50 cents so that I can check my weight, the guy laughed when he read it, he said you are about to die, can you see what you weight reads, when I arrived at the clinic and weighed myself I was no longer afraid since I got here knowing very well that I had lost weight.

Ps: Yes.

M: Uhm you were not afraid anymore]

P004: [I had already realized that I was dying.

M2: And during weighing, when they were weighing you now you have stepped onto the scale, how do you feel?

P004: No, I am okay, I do not have stress.

M2: You do not have stress. Others?

P006: No, I feel good also since I was shocked on the first instance when I had to stand on a weighing scale but anytime when I go to be weighed I am relaxed since now that I have received counselling, I am okay. Yes.

M: Is it possible that all of us who went to weigh ourselves got the results that you lost weight, us, who have weighed ourselves? (.)

P005: Let me say so my sister, I have been a person who usually weighs himself but there was this one instance where I had flue, I decided to visit the clinic so that I can find out what I was suffering from since I had realised that I didn’t have my usual power. When I got there, I learned that I had lost weight, I did all my stuff since I received and I am still receiving counselling, I believe that I will be okay like other people.

P006: I used to not feel very sad as I did not lose a lot of weight, it used to drop by 3kg, you would find that perhaps it drops by 2.5, that did not frustrate me that much but standing on the scale was frustrating, I had those thoughts that hey maybe I lost a lot of weight, but when I did it, I found out that it was 3kgs only.

M2: And after weighing you?

P006: Eh that is what I found after being weighed, that I had lost 3kgs only so I was better.

M2: Okay. Number 5, after?

P005: That is what I can say also since we have just been weighed. (.)

M: If perhaps a person who is living with HIV is asked by the clinic staff about weight loss, what would make a person say they lost weight?

P007: I think that what can make me say that I have lost weight, I think I can say it’s stress, frustration that hey perhaps I find that counselling is not effective, a person keeps thinking of the situation that they are at, I think that it will be in that manner, when they do not understand counselling and the situation that they are at and realising that they will live like other people and also not understanding what makes you to lose weight.

M: So in other words, what makes a person to report that they have lost weight is that after they have been stressed out they think that they lost weight.

Ps: Yes.

M: What else will make me to say I lost weight if a nurse at the clinic asks me if I have gained or lost weight?

P006: I think that it will be knowing that you have a virus inside you, then there will be that thing where you will tell yourself that actually since one of the symptoms of a person with the virus is losing weight, then I have lost weight in that manner.

M: Weight loss is one of the symptoms.

P003: I say as a person, you know your body, if you lose weight you can see how that happens and people that know you will actually tell you that, we know you as being okay, fit and well what is causing you to lose weight like this, you see that type of a thing. Even you when you look at yourself, your pants are bigger than usual now and you have changed the belt size, you make your pants smaller than usual now, you see that type of a thing. Even when you put on t-shirts they are just hanging they no longer fit you in a manner that they used to before, you see that type of a thing. Other things are not about stress sometimes you just see yourself as having lost weight, you see whereas sometimes not knowing what is happening until maybe you visit the clinic where they inform you of your condition that’s where you realize that eish this is what made me to lose weight, you see that thing.

M: So you are saying you yourself can tell, people comment, the belt shows and the clothes]

P003: [The clothes will also tell ((show)) you.

M: Other than that which has been mentioned, what else makes to us to report weight loss at the clinic?

P004: Sometimes we do not tell ourselves that we are sick we will recover or you just do not have that hope that you will heal.

M: So not having hope brings about weight loss.

P004: You see that type of a thing.

M: Okay, we are trying to find out, what it is that will make you to report to the doctor or a nurse that you lost weight? Other than that which we have mentioned, what makes us report weight loss?

P005: Food also, you see, as HIV positive people we need food, good food, if you are not going to eat good food, the nurse will say I lost weight, you need food that is trusted to have a good effect.

M: Okay, in other words what makes us to say that is that there will be instances when we do not have good food hence we will think that we lost weight? You wanted to talk my brother, what were you saying?

P: I have forgotten.

P004: Sometimes I ask myself a question, sometimes you will try to eat good food and find that you can’t eat it, you see when you are trying to eat junk food, chips and bread, you see you can eat it but when you eat pap and spinach you do not have appetite, you eat 1, 2 spoons, you are full, if you try meat, you also do not have appetite but the food that is not good for your health is easy to eat. So what causes that?

M: Okay you are saying that at the clinic you will report that you lost weight because you did not have appetite to eat good food.

P: It is indeed something that happens, you see, it will be that, you will dish up good for me and I will just take a bite and put it on the side, but junk food will go down the throat quite easily. When you are trying to eat good food, it’s not appetising, you will feel like eating burgers, quarters ((common type of junk food in the South African townships- quarter of white bread with the following contents, a fried egg, cheese, polony, etcetera a high cholesterol content)), you see.

M: Okay, what does losing weight mean to a person who is now living with HIV?

P003: I think that it’s the antibodies maybe the CD4 count is low, yes.

M: Uhum what do others say?

P006: I think that an increase in the viral load in the blood makes a person to lose weight.

M: Okay, it’s the viral load and theCD4 count, what do others say? What does losing weight mean to us?

P004: Perhaps thinking a lot also, you see.

P002: Sometimes not receiving enough support, you see. You will find that you are okay even if you are trying to avoid stress, but your mind is fixated on a problem, you see, you do not have a lot of time to laugh. You will find that you are quiet all the time and obvious when you are quiet, there is nothing else that you are doing but thinking.

M: Okay, let me ask this question in this manner, if I am HIV positive I see myself losing weight, what goes on in my mind, what thoughts to come to my mind when I see myself losing weight if I have HIV?

P: You know that you are sick.

P001: I get an idea that I need to go out and inform someone close to me, the one whom I regard as a neighbour, I will talk to them and see what their responses will be. To get advice from the people that I trust not someone who will run around the street and tell people that I am sick.

P006: I am afraid and scared such that I can have the thoughts that perhaps I am going to die, it’s just being afraid and fear.

M: Okay, all right is there perhaps stigma attached to a person who is losing weight and aware of their status that they are HIV positive, what is stigma in isiZulu? (.)

M2: But do you understand what we are saying?

M: Do you understand what we mean by stigma?

P: Y:::es

M: That when you are HIV positive and you have also lost weight, what do people in the communities that we stay at usually say about us when they are pointing or gossiping about us, stuff like that?

P: They speak ill.

M: What do they say?

P004: I think this is no longer a joke, it’s no longer a joke because it is now something that they play with.

M: Okay, why do you say that number 4?

P004: I am saying so my sister because everyone is talking about it even young children at the crèche they are talking about it, no one can say they don’t know this thing, I think that it’s no longer something that people can be afraid of. Or laugh at another person to say can you see what they are suffering from, it’s no longer something to laugh at or to point at person in a bad way, it’s now the same as when a person is suffering from flu.

M: Okay, I would like to remind you of something, you see as we are in a-group, we will have differences in opinions, what my brother thinks may not be same as that which -number 3 is thinking of also what number 6 thinks may not be the same but I am just going to request that we just talk even though our opinions are not the same. Having said that, I am trying to find out as my brother is saying that the situation is no longer the same, people are not laughed at, there are no names, there is no gossiping, is it possible that the situation is like that in all areas that we come from or is there someone who can say, what I know is not the same as what my brother thinks.

P003: I am saying no because not all people will like you, others do say, where did he think he was going to end, you see something like that.

M: Others still say?

P003: Others still say, you see they still laugh you see since not all people may like you since you are not money, you see.

M: Yes.

P004: Can I a bit ask]

P006: [Now, another thing is once people find out that you have HIV and stuff like that, the thing is that previously it was easy to notice a person since ARVs were not available then, you find that you were going to be noticed since you will lose weight, then people will say you have a virus that’s why there is still that in people’s minds, once you lose a bit of weight, they say that you have HIV, yes.

M: Okay, okay.

P005: I also add especially at work, you see if you are someone who is employed, if people notice you, they talk, they will say this one is suffering from this, yes. Some people still have that thing, yes. It’s still taking place because I am still repeating my brother’s word that not all people will like you, some people will be able to advise you, others criticise you, yes, saying he is this and that so things like that especially at work can sometimes can lead to one losing a job since one between the two of you ran to perhaps inform a manager, as you see him, he is this and that, you see until the manager takes the decision to ask you to leave and you wonder why am I losing a job. I lost a job and then I am in this situation, where am I going to get support so that I will be able to support myself. You will find that you lose your job through people.

M: Okay, are there perhaps names that they call us by, once we have lost weight such that we can be identified that we have HIV in the community? What names do they actually call us by?

P001: They call you 3 series.

M: What do they mean by the3 series.

P001: HIV, they count the three letters of alphabets or they say it’s a BMW.

P006: I sometimes hear them say it’s a Z3.

M: Uhum, what other names can you still recall? ((Ps laugh)).

P: They say you are three names.

P: Or 3 million, you won the lotto 3 million.

P: Others says it’s OMO

M: Who?

P: OMO, since it has three alphabets and that it washes clothes like HIV washes your body.

M: So these names are used openly?

P: They will actually say, so and so bought OMO can you actually see so and so, so in that manner they call you by those names when you are on the passing by and you would think my goodness.

P: They do is so that you cannot understand you see, you may not know why you are regarded as having bought OMO, when you look around no one bought OMO, they regard you ass that since you lost weight.

M: Okay, all right if a person is attending an HIV care clinic perhaps where people get ARVs what do you think their weight should be? Here I am, I have found out that I am HIV positive and my CD4 count allows me to take ARVs, I go and take ARVs, how do you expect my weight to be when I am taking ARVs?

P003: I am expecting it to be in a certain condition.

M: To be in a condition, what type of weight is regarded as being in a certain condition?

P003: It’s that which I had before I lost weight.

P006: As number 6 I think that you lose it until you recover when you are on treatment. That is when it has dropped but once I perhaps started taking ARVs then I regain it. That is when it has dropped then once I start taking ARVs I get back my normal weight.

M: Sorry?

P006: I mean that before I take ARVs I would have lost weight anyway maybe after some time after taking ARVs I can then gain weight and then go back to my normal weight.

M: Okay, what about the shape, the manner in which the body is shaped

P003: I am saying the shape itself automatically, the more you regain weight, you will get it back, isn’t it it’s your body, you will regain it automatically.

M: Okay, so you think that the shape will be in line with the weight]

P006: [It will be in line with weight gain. Yes

M: Okay, all right, I hear you, I will give you these photos which I will ask you to look at, these are males okay, their weights are different, I will give you time to look at them before I ask questions, look at them my brother as well.

P002: How much time?

M: Look at the photo that you are holding (.) We have seen them right?

Ps: Yes.

M: If perhaps we look at these photos, what can you say about the ideal shape or ideal weight, amongst the photos that we looked at which ones can we say, this one on this photo, number 1, we see him having an ideal weight, do not forget that I said we can have different opinions and we have to respect that?

P003: I think it’s number 5.

P005: As number 5, I think it’s -number 5.

M: You, number 5 also.

P001: I think it’s see number 5.

M: You think it’s number 5, what do others say?

P006: I think it’s number 6.

M: Okay.

P002: I think it’s number 5.

M: You like -number 5 also?

P002: Yes.

M: Okay.

P004: It’s number 5.

M: You like number 5. Why do we like number 5?

P001: He has a good body weight, he is not thin and he just seems healthy.

P003: His shape is good.

M: If we say his shape, what do we mean?

P003: The way his body is shaped, the -body shape.

M: Okay, his shape is desirable.

P003: It’s the shape that I also want.

M: What can we say about that of men and that of women? As we have these photos in front of us here, can we say perhaps that a good weight for men is this, and that of women should be that? Do you see a difference between men and women or do you say it’s the same?

P001: It’s clear.

P003: There is.

M: Okay, what is the difference?

P006: For women, the weight that I see as being okay for women is number 4.

P005: For women, I think the weight is 5 again.

M: What do others say?

P003: I still think it’s number 5.

P004: Number 5 is okay.

P002: I still think it’s number 5.

M: It seems as if number 5 is likeable, why do you choose number 5 for women?

P005: His shape, is what I see as being in a woman’s condition, if she reaches there, she should not exceed this.

M: She should not be bigger than number 5, okay, for both the weight and the shape, what do others say?

P006: Anyway, the weight that I like for a woman is that of a slender. Number 4 so is what I choose.

M: Okay, this that we are saying, that this one looks healthy, this one’s shape and all that, where do we get that from, these opinions that we have about these photos what we are looking at. Where do we get them, we learn in different ways, sometimes we learn in the community, sometimes we say certain things because of culture, the manner in which we were raised, sometimes we say things that we hear our family member saying, sometimes at the health services, they said this and that at the clinic, sometimes the things that we say are those that we have seen in the media for instance the television, the radio or the newspapers. So where did we learn this that we are mentioning now?

P001: About women?

M: Just all, men and women, this weight which we regard as being good and not good?

P003: There is one problem, isn’t it life is about natural different choices so that is why then each and every individual will have their own choices, it’s just something natural, you see.

M: We are trying to find out, what are you are saying, is it something that you learnt in the community or is it something that you read on the newspaper or did you hear it being said at home? Or is that how you were raised?

M2: Or is it the TV?

P003: We observe things outside here.

M: If you say you are observing outside here, what do you mean?

P003: We observe things as we move around outside, as men we can say that, we observe things, we look at people, we check that this one has a good body and this one has this type of a body, that is where learn that it means that this person has a good shape, you see.

M: So you observe on your own out there, is there perhaps something else which you think you get from the community?

P005: There is that thing, where you find in the community, you see as we are seated, you know I don’t want a person who is like this at least I want this type of a person, where do you get that, you get that in the community that oh most people like this type of a person, they like this type of a person, we do get other things from the community.

P006: I think that some is from the -health facilities, for instance they do not recommend being overweight because you find that we get attacked by diseases like heart attack and BP, it’s not good to be underweight also at the health facilities.

M: I get you and what about family? Does it happen that things that are said at home shape our thinking in terms of us having to say a person has a good weight, this person does not have a right weight.

P003: Yes, I think since there can be one who is a -role model so you also -adopt their style, you see.

M: Uhm, a role model within the family influences your thoughts and the opinions about things, what do others say, what about the media ? Is there a role it has played in terms of it informing us that number 5 is okay, number 4 is okay?

P001: Of course the media also play a role, for instance, let’s look at women perhaps you find that there is a beauty competition isn’t it within the beauty contest they want a slender, I think that in that manner it plays a role.

M: I understand.

P001: On the man’s side also, amongst the body builders perhaps you find that there is a competition for body builders, perhaps you will find that you have lost weight like number one, you will find that you are no longer able to qualify in those type of competition.

M: Okay, all right, what about culture, does-culture has an impact in us saying that person’s body shape is good?

P001: I do not think that culture has an impact. It does not have an impact on the body shape and body weight.

M: Okay, what do others say? Do we all agree?

P003: Actually, I don’t think that culture fits it there, it doesn’t.

((Ps talk at once)).

M: Actually, I can see all of you shaking your heads. Okay, let’s continue then.

M2: Okay, sometimes you can visit the clinic, you tell yourself that you have lost weight but when you stand on the scale, you show that you did not lose weight and you are still the same, why do you think that happens?

P006: Actually, that happens my sister, you will find that even maybe by the clothes that you put on, they show that you have lost weight but when you stand on a scale you will find that you did not lose weight, why, you will find that your bones also play a role. You can think that you lost weight but find that your bones show that your weight is still okay.

M2: Okay, what do others say? You are at a scale you did not lose but when you look at yourself you say yes, I did lose?

P005: I do not think my sister that I can think that I lost weight but when I get home]

M2: [You do not think that it happens?

P005: No, I don’t think that I can say I lost weight feeling that I have lost weight and then gain, no, I cannot gain in that manner, feeling it, I can feel that myself, isn’t it this thing is in me, I can feel that I lost weight, I can see and I can tell or another thing when I am dressed I look at myself, I cannot actually gain]

P003: [Can I please ask, is the weight on the bone or the flesh?

P004: Can I please respond to my brother, the thing is, the body does play a role why because sometimes you can see your body looking as if you have lost but since your bones still have your previous weight, you find that you are still on that weight. P005: What remains is you lost weight. But when you stand on the scale you find that you are still okay, sometimes you lost a bit of weight.

M: Okay, so we are saying that, that is when the difference is very little?

P005: that is when it is small because that is when maybe it’s 2kg.

P004: 2.5

P003: 3.

P004: Something like 1.5.

M: What do you think losing weight means to a person who is attending an ARV clinic, someone who takes ARVs?

P001: Can you repeat that my sister?

M: It means I went and collected RVs but I was seen losing a lot of weight, what does that mean?

P004: The food that I eat makes me to lose sometimes that is when you are not taking your tablets accordingly or not doing things accordingly.

M: Okay.

P004: You are not doing it accordingly.

M: Okay, all right, not taking the treatment accordingly. Did you have your hand up number 5?

P005: I am saying, if you keep visiting the clinic but you are still losing weight it may happen that maybe there is a disease that you did not check or you are not taking your treatment accordingly and you are drinking it with alcohol, you smoke and the drugs so to speak, that does make you to lose weight even when you are collecting your treatment.

M: So there is an underlying disease which is there that is hiding?

P005: Perhaps there is a-disease in your blood which is not seen.

P006: Sometimes it happens that it’s that thing which is rejecting it why, if you will keep…, you are -HIV positive okay and then you continue sleeping with different people without a condom so each of those people give you their virus, so these viruses are easily consumed by your body.

P001: To summarise this, you are not doing things accordingly.

M: That’s why I am losing weight?

P001: Yes. You are not doing things accordingly.

M: If I take ARVs and gain weight, what does that mean?

P005: It means you are following instructions according to how they have been set, according to how you were taught when you were taught about what to do with tablets.

M: What do others say?

P001: You mean being overweight my sister or?

M: No, I gain weight; it means I get bigger than what I am now. (.) I take ARVs, when I started taking ARVs I gained weight, what does this mean?

P001: After having lost weight?

P003: That means the treatment was received well by the body, you are complying with everything.

P005: You are doing everything okay.

M: Other than the treatment is there perhaps something else that can make me to gain weight when I am receiving HIV care including counselling and all that stuff?

P001: Yes, there is isn’t it sometimes you would have received counselling and accepted, you see, the body then becomes normal.

M: What does gaining weight mean to a person who is living with the HIV virus?

P003: It means that life is still there, yes.

P001: It means that you are relaxed you are not obsessing over your HIV status, you see, you do not put yourself under stress, you think that I do have tablets. If I take tablets everything is okay.

P006: The treatment boosted the -immune system inside and lessened the viral load in the blood and increased theCD4 count.

M: Okay, my immune system is boosted and that is why I am gaining weight.

P003: I am free, I accepted, you have accepted the situation that you are in.

M: Perhaps then which way do you think is the best way to ask people if they are losing weight or they are gaining weight, people who are living with HIV?

P005: When I see them losing weight I ask them if they take the treatment accordingly, if they are taking it as expected.

M: What do you think perhaps will be the best way to ask a person if they lost or gained weight, how can we ask a person if they have lost or gained weight? Which way do you think will yield an accurate answer, to say this person has indeed lost or gained weight, that which will give us an -answer?

P004: What will give us the answer is just for that person to stand on a scale.

M: Ahh okay people should be weighed, what do people say, do you want to say something?

P001: I want to say something similar to this that it is to stand on a scale, that’s what I also wanted to say and my brother has already mentioned it.

M: Okay are there people who want to say something different from what my brother has mentioned.

P003: No, I think that is what it will be, that they should just stand on a scale. Now since you can see that they have lost weight they do not want to accept, how will they respond and what will they start with?

P005: When I meet them, I will greet them, how are you my brother sure, you see, now we talk, ei you are losing weight, what is going on, he will be the one to tell me that no he was involved in a car accident or something like that that is what made him to lose weight, just that thing of asking, if they have lost weight.

P004: Just ask him directly?

P005: That hey, he is the one who will be giving me results to say no, I am losing my weight because of this that and that.

M2: What do you think your partner will say if you lose weight?

P004: The first thing to consider is the depth of the conversation that you and your wife have regarding your disease or the disease that you both have but if you discuss and advise each other about it, I doubt she will ask you about weight loss and stuff like that, she will not ask, isn’t it she can see that you gained weight. It will be to ask what did I say I was going to give you, perhaps she cannot see, she cannot see and even our lifestyles, she cannot ask.

M2: Okay, what do others say?

P006: The first thing is, she will think that you will leave her ((you are going to die)), there will be that fear that if so and so’s father lose weight it means automatically I will also lose mine since we are in the same situation, there will be that comparison with me that she will be like me also.

M2: What about children?

P001: They will just support them.

P003: You see my sister, I don’t think that they are people to be told, I think that if you get along well with your wife, children, it will depend on their level and that which you could discuss with them if you sit down with them perhaps when they are adults, when they have grown up, you see, you can be able to tell them but if they are still growing up, no it cannot happen that you can take your 13, 7 year olds and tell them, you can tell those that are 19 that you and your wife have a virus, no, we do not tell them, we keep quiet, it’s something that only both of us know we will tell them only once they are grown up or if someone gets very sick and when we realise that actually they are about to die. We then call them and say hey it’s like this and that in this family, so you must know if I die as I am in hospital that I died from this type of a thing. I don’t want you to hear from people.

M2: And family?

P005: I don’t think that a family are people to be told since they shout at you from the street.

P006: I think that it depends on the family, you know, there is a family that supports you and that you share ideas with. You will find that sometimes the family is fragmented, you see, in that case, it will not be easy to tell your family what is going on.

P001: I was agreeing with number 6 in such a way that when I told them, I asked them not to tell so and so for the time being, because we know how they are.

M2: What about friends?

P005: I will not tell a friend, it will depend in our friendship, for how long have we been together or how do we help each other.

P004: I do not tell a friend, we discuss that there is something called HIV, if he can be infected how can he be helped, I do have it, I want to hear from him and what his response will be at that time without me telling them that hey man, I am like this and that, just talk with him.

M: When do you that, are you putting in the feelers to see how he would receive it if you were to tell him?

P004: Yes, if I tell him, isn’t I am telling him now that there is something, I want to hear if…, I want to see if he can keep this thing within himself or if he will spread it all over.

M: But it sounds as if friends are not the people whom you would like to inform, am I wrong if I say so?

P004: It is so.

P001: Actually, it is indeed so.

M: Why are friends not people whom we will want to inform?

P001: Yes, we are not the same, I informed my friend directly and said my man, things are no longer okay with me, the lifestyle is changing, please visit the clinic for a test because I am already infected by the virus, I was diagnosed with it, he was very sad, he was so sad such that he asked if there is anything he can do to help.

M: In other words it means friends are different?

P004: They are very different, there are those that spread it and there are those that know that okay if my friend and I sit and talk, it ends between me and my friend. There is this friend who will hold you by hand and just pass to tell others.

P001: Sometimes you will see them and tell them that my man, can you see that you are coughing, they do testing at the clinic, let’s go, he will ask you if you have been there, you will then say I was there yesterday, I tested and I also want to show you that I am getting tested here isn’t it you know that you are here and you know the route, you bring him here to check again. You know that you are here, you are no longer following many queues, you know that you are taking tablets, he is the one who has to follow this queue.

M: So we encourage one another.

P001: Yes.

P004: It’s just to push them to go there since most do not want it, when you tell them they get very angry.

M2: Okay, what about the community?

M: Ei 1 is shaking his head. 3 takes heavy breaths when we mention the community.

P003: No, it’s difficult in the community

P005: No, as number 5, I do not see the need for the community to know about my life.

P004: What the community knows is to just say there is a disease called AIDS and they point…, if you lost weight, they will say did you see so and so he has started losing weight, what is he eating him up, that is their way, by just looking at you, you are telling them. If you pass by going to a spaza shop, did you see him, if he is telling them verbally]

P003: [To speak the truth, no it has not been accepted as yet, it is indeed not the same as before but there is still that thing, there is still that thing that so and so bought OMO.

M: 1 shook his head when we were talking about the community, I am interested in knowing what is it that made him to shake his head when he thinks of the community?

P001: You see if it’s a community, you are referring to many people so telling them really, how are you going to tell them. It will be a big group of people, that you are going to be speaking to at that time, you cannot be able to face them and tell them about your condition, unless if people like me attend. When you talk about this type of thing, it will be as if I am supporting you, I will just give evidence since I know the situation that I am at but I will not disclose my status.

M: Right now my brother we are discussing how the community responds when it see a person who is living with HIV and who has lost weight at the same time?

P001: Actually, it’s obvious they will say something happened to so and so hence such drastic weight loss, they do not care of the fact that you got tested or that you did not, they will just say that so and so bought OMO.

M2: What about the doctors and the nurses at the clinic?

P005: You see at the clinic, the nurses scold at you if you visited them when you were really too sick and badly affected, you can see that you have lost weight, the nurse scolds at you and say you are this and that why did you not come and visit me ((says P mimicking a female nurse))) they scold at you there. They scold and shout at people asking why did you wait until you got to this condition, whereas you didn’t want to get tested.

M: It means they scold at us once we get there at a later stage.

P005: Yes, it’s difficult because they do not know also which tablets they should start you on, you see.

P001: That is why they say that visit them when you can still walk, when you are still well, when you can walk on your own not when you are pushed in a wheelbarrow.

M2: How does a person living with HIV feels when friends talk like that. How does she or he feel?

P001: Say it in Zulu.

P003: You mean how do you feel when they talk bad about your weight?

P005: Can I make a comment in this manner, the thing is, you will sometimes hear a person saying hey I am from talking with so and so, he lost weight, a person will not tell you in your face that you lost weight but you will hear that friends were seated in some place saying hey when you were passing that side, they talked about you saying you lost weight, they will also be trying to get the inside story also, if you have indeed lost weight.

M: How does that make you feel for instance if those type of things reach your ears?

P005: If I know already that I am HIV positive it will not make me to feel bad since I am already aware of what is eating me.

P001: What makes me lose weight, I am not the same as a person who does not know that they have lost weight.

P006: You know that you are taking -treatment, you are going to recover whereas even when he sees that I lost weight he will not know.

P005: I think that it’s very difficult if it’s before you got tested since you have that fear that hey if I go for testing maybe I will be diagnosed with HIV.

M: Say perhaps I am in the community and I hear these people talking about the Z3s, I hear them saying I have bought OMO all these things that we have mentioned which we say people say to people who are HIV positive when they lose weight, generally how do they make you feel? How do you feel about these things that are said by these people whom you have mentioned?

P005: Let me once again go back here, I once came here on my appointment date to get tested and was seen by one of my neighbours here at the clinic, when she saw me do all my stuff, when he saw me in my neighbourhood, she asked why were you at the clinic? Did you go there to test for AIDS, just like that, you see, she was shouting me in front of people. I did not like that I simply told her that yes, I needed to know about my health, to ‘qualify’ if I am healthy or not you see, she had brought her child who had had boiling water burns here, I just told her that, I was there indeed to get tested that AIDS that you are talking about, she did not mention any other reason for my visit, she just said, you were there to test for AIDS. I then said, yes, I went to get tested for it, the thing is I needed to know my current status.

M: How did you feel?

P005: She kept quiet.

M: I am referring to you as a person?

P005: I did not feel bad because I can see that my condition compels me to take treatment so that I can go back to my original condition but now, I could see that okay, as time goes on, she will come face to face with the problem that I encountered.

M: Uhm. Number 3 what are you saying, I can see you shaking your head like this (.) What’s wrong now, would you like us to stand for 2 minutes.

P004: He says he feels the pains now.

M: Oh okay, I am sorry.

P005: There is this thing of people speaking anyhow which is taking place, you see if a person simply talks anyhow in the community but then if you are aware of your status you are better than when you were not.

M: When you say shouts, you are saying shouting in what way?

P005: When a person shouts like the person I am referring to, she shouted at me on the street you see, hey so and so what did you go to the clinic for, were you there to test for AIDS, I said I was there for that.

M: Okay what do your partners say when you gain weight?

P006: It brings back hope.

M: What are we saying there, are we okay?

M: What do others say, we are talking about gaining weight now? Our partners, what do they say when we gain weight?

P001: We have not gained weight as yet since we have not started taking ARVs yet.

M: You will recall that earlier on, when we were starting the conversation, we said in some instances we will not talk for ourselves only, we may know people who are HIV positive in the community, stuff like that, and the things that we sometimes hear people mention about their partners and that which they say when they see them gain weight, perhaps they say this that and that, do we have a person who has heard those type of things?

P006: Yes, I have a brother who is HIV positive, he lost an enormous amount of weight, it was not a nice experience but when he gained weight, there was that hope that yes, he is now recovering.

M: Uhm so it’s a hope for recovery.

M2: What do children say?

P004: Isn’t it children do not know if you have gained or lost weight, they do not know that.

M2: And you are saying you do not tell all of them in the family?

P004: We do not tell all of them.

P003: It will be this one and that one, they are okay.

P001: They have a tendency of telling you when you arrive, they will say my goodness, you have gone back to your normal condition now.

M: Ahh, who says that, family or children? You must not forget that in as much as you have not informed your children as a group here, we can still talk about the neighbours or the people that you sometimes hear from?

P003: And from the TV or something like that?

M: Exactly.

P006: As I am saying that my brother is in that type of situation also, his children were aware of the type of situation he is in.

M: How old are his children?

P006: One is xxx years and the other one is xxx years.

M: But he informed them?

P006: Yes, he informed them, isn’t it they say we need not hide anything. Even at school, they learn about this thing. So why would you hide it since he will be the one to ask, what’s wrong mummy, why do you now look like someone we were studying about at school, what is happening to you, you can see that it’s obvious that you need to explain to him what is happening, you see and that is where they will be okay when you make them aware of what is happening. Even when you gain weight, you need to explain what is happening. Indeed, we do not hide anything anymore.

P005: Even these photos that we look at, a child finds these photos, actually there is a number of them, the child will also see that this person lost weight, this photo does not look like my father, dad, what is your problem.

P006: They also learn about these things at school.

M2: If you informed them, what happens when you gain weight?

P001: They are okay, they will be okay.

P006: They get happy that their father gained and they no longer look the same as before, they will be happy.

M2: Regarding the family, you said they will say, they can see that you are gaining weight, what do others say?

M: You said, you said you are regaining your body back now.

P002: They say, hey, you are regaining it, you are coming now.

P003: Who says that, your child?

P002: The family, yes, the family.

M2: What about the community?

P006: We did talk about the community.

M2: When you are gaining weight?

P006: Okay, when you are gaining weight?

P003: It depends, if the community is used to talking about those things. If it’s not used to talking about those type of things, they will not discuss that ((a cell phone rings)).

M: What does number 1 say? What is the community’s usual comment when they see us gain weight, when you regain your body, gaining weight now?

P001: They just say he is taking the tablets, can you see, we can see the bums now. Ahh okay, they say look, he is regaining his cheeks.

P003: Yes, he is speaking the actual truth.

P001: But they will not say that to me directly, they will discuss that around the corners, they will gossip and say can you see him, they will say can you see OMO has started taking the tablets, we can now see the stomach. Can you see Z3 that is your name now.

P003: If they are talking about ladies they talk about hips, can you see hips.

P001: Actually, the big stomach they say he has bought a 12.5…, what is this, what do they say you bought, they mention this thing man, there is a term in my township which refers to what you have bought.

P006: If you lose weight, they say the big tummy has become flat, they say the big stomach has been made flat, what is happening, they want to know, but they will not come to you.

P004: But others will tell you that others say this is the situation, you are in trouble man.

P007: One will come and ask you, he will tell you indirectly I hear them say this, is that really the case.

M: So if you have a big stomach, they also associate a big stomach with the use of ARVs as they say you have put 12.5 inside your stomach?

P001: Isn’t it, it depends on your weight, how is it shaped, isn’t it even when you hide, they will see this stomach that you are losing weight, it will become flat. When you regain your big stomach perhaps you are taking ARVs also, it accepts them, they are received and working well, you regain your big stomach, you start again, it means your body regains its condition, the way we know it to be.

P004: Let me say, you can see that I am slender but if you can I take these tablets, you see and acquire a big stomach, they will then say he is in this condition, he is now taking these tablets, isn’t these tablets are making him to have a big stomach.

M: So in other words you will still be slender but the stomach will be big?

P005: Yes, I will be slender but this stomach will protrude.

P006: Now, even that, you find that sometimes if perhaps you are drinking alcohol heavily, and you do not take the tablets on time, obvious the stomach will be big and they will make comments then.

P005: If you were asked to take the tablets at 8 and you take them at 9, 11, you see and you are taking them with alcohol, you did not eat and they will find alcohol, they will just make you big ((inflate)) like yeast, you see this one for baking, yes, they will just make you big and you will be small this side.

M2: How do friends respond when we gain weight?

P005: They are okay, friends are that hheh my friend you have worked now, you are regaining your weight, they are okay.

P001: We will go and look for work then.

M: Sorry?

P001: I am saying, once I gain weight my friend will say, you see now, we will look for work together.

P005: He was no longer keen is walking alongside you.

P001: Yes. They thought that they will be embarrassed now that he has gained weight they then want to be close. They were afraid of walking next to you when you didn’t have weight, being worried that you will fall dead there at the firms.

M2: What about the nurses? The doctors?

P005: They get happy.

P004: They are happy when I gain weight, they can see that their work is good, they can see that their work is going well and they can also make you an example perhaps you have just come to collect your tablets, they will say, you see you defaulted, look, look, look you are fresh now.

M: How do you feel when they use you as examples?

P001: Happy.

P003: I am happy now.

P004: She is exposing me now, that I am…, I do not think that I will feel good.

P001: I am happy because this thing, they do not say it once.

P004: But she shows that you are someone with HIV, you see, she is disclosing that you have HIV, if she says here is so and so he is doing well, the tablets are good on him, he is disclosing your status there, you are a book that people can read since you have HIV, you see.

P005: But on the other side, she is encouraging others, so that they can take their tablets and she is disclosing your status on the other side, by the time they leave the clinic, they know that I take this tablet, isn’t that is where it is not good. She has not been given permission; she is abusing my right.

M2: How do you feel when you gain weight?

P003: We feel good, it means that the treatment is working.

(.)

P004: It is working.

P005: That makes us happy.

(.)

M: Okay, in general, do you think that the bodies of people living with HIV change? (.) Their shape?

P005: The shape is what you were; I do not think that it changes.

P004: I think that it will change if your CD 4 count is low, you will just lose weight, you will also find that your viral load has increased.

M: So you will lose weight right?

P004: Yes, but once you start recovering; it will go back to its original size if you will do everything accordingly and follow all the rules.

M: Is it the same for men and women? That the shape does not change]

P005: [Our bodies are usually different for men and women.

M: How?

P005: You know sometimes you will find that the tablets make a woman fat, you see, they usually gain weight whereas our weight is normal.

M: Do we sometimes hear of the body shapes that change when people are using ARVs maybe a person will say they have a buffalo hump, that which grows at the back of the neck and grow upwards or a person will grow a big stomach or fats will leave some places and locate themselves someplace else, people will develop huge bellies so much such that we will think that they are pregnant? (.) Have you heard of the existing body shape changes that are caused by the ARVs.

P001: Yes, we have heard about that.

Ps: Yes.

M: Okay, what do you think cause these changes in people’s bodies?

P006: I think that for others, the tablets were not good on them. I think that they were just not good on their bodies maybe until they need to change the regimen. Besides perhaps that there is something that he did, maybe that he did not take them in a correct manner, it’s just that they are not good on him, they then change his shape.

P005: Sometimes they have a tendency of taking them this year and then leave them in the middle in the following year, he will then start to be sick on the following year and then go back to the clinic to ask for the tablets, they will then ask for everything from him, another disease and they will make him to have a big stomach and change his appearance.

M: Okay, so we think that it’s caused by defaulting.

Ps: Yes.

M: What are we saying, we are saying it happens that the bodies change, is it changed by HIV? The mere fact that a person has HIV, her body shape changes as compared to that it was before? For instance, there is a loss of fats in the face?

P006: People’s bodies are not the same, for one person, there may be no difference, you will find that they will just be good on them and his body shape will be good.

P005: Another one, there will be that, they look dry and look as if they have ring worms you see and it will make them look dry and there will be no fat on the body. That does happen because the bodies are not the same.

P004: Uhm.

M: Okay.

M2: So if a person’s shape changes what are wife’s or partner’s comments? (.) We are talking about the shape now and not the weight.

P006: She will ask you, can you see though what these tablets are doing to you. They are changing your body, your shape has changed.

P001: They will ask if you can notice your appearance.

P004: Sometimes if you are perhaps a person who was not well educated, she will say stop taking them.

M: Uhm?

P004: She will say, she will say you see this thing, it’s making you to grow a big tummy and then do this and that in your blood, isn’t it when you are not focusing on yourself as your partner who always looks after you, she is.

P005: You see, then.

M: Does it ever happen that perhaps from the discussions or the conversations that you have or just things that you hear in the community, that so and so’s wife or the husband says they must stop taking ARVs since they were changing the manner in which he looked. Does it ever come to that point?

P005: It happens.

M: Does that person stop taking tablets completely?

P004: As I am saying my sister that you see another person will stop in the middle of the year after having started taking them, isn’t it at that time he is okay, isn’t it when you are taking them they give you boosters to say I am well and alive, you see, when they make you develop these things is the time where you should be going to the hospital and perhaps a clinic to say they are making me to develop these things but they are boosting my immune system, it’s the time then my wife says, hey leave that thing, when I am sick she is the one also who takes me to the clinic. At the clinic she just drops me, she says follow the queue here and she runs away. Isn’t it she is the one who suggested that I stop this thing and do this.

P005: It means we can just say that it’s the lack of knowledge, it’s not having the information.

M2: What will children say when you change the shape, isn’t it you have told them.

P004: They will comment.

Ps: They will -comment.

P004: People like to keep commenting, they will comment and say hhah dad, you have a big stomach now, what is happening.

M2: The family?

P005: It’s the same, children and the family are people that just make comments.

M: A comment can either be negative or positive, what type of a comment is this that we are talking about?

P005: It’s usually negative, you know people, you do good, they will comment, you do wrong they will comment, even when you have tried and gone to collect the ARVs, once you use them and they are good on you, they will be happy, even if they are not good on you, they will comment nevertheless.

M2: We are talking about the family, right?

P005: Yes.

M2: What about friends?

P004: It differs with friends, there are good and bad friends, their comments differ. There are those that give positive comments whereas others give negative comments.

M2: If they are positive, what are they?

P005: They will encourage you and say continue with this treatment, can you see that you are okay, you are no longer the same as before, continue with the treatment.

M2: If you are negative, what are they?

P001: If they are negative they will criticise you, they will say you have a big stomach and make you feel like you can stop taking the treatment.

M2: And the community?

P002: The community will keep talking in the corners.

M2: If your shape changes?

P001: They will say, did you see how he is, I saw the stomach.

M2: What about the doctors and the nurses?

P001: Isn’t it they]

P004: [They will ask you how you are feeling health wise?

P005: Are you taking your treatment accordingly.

P006: These tablets that we are giving you, what do you think they are doing to you.

P005: Isn’t it they know that if they make you to have a big stomach, there is something that you are not doing right, then they will tell you that in order for you to have a good stomach, do this and that and that also.

M2: Okay, a person who lives with HIV, how do they feel when they talk like that? When they comment about his shape?

M: This which is no longer good as a result of ARVs?

P004: Isn’t it when I am looking at myself in the mirror, I do not like my shape also and obvious when people comment, it will be painful.

P005: It will not be pleasant.

M: What do others say? (.) It was a last question. Thank you so much for the information that you shared with us considering that you were already tired from the other group ((ARV initiation) that you were in before we started. We very much appreciated your input. Thank you.

P005: It’s our pleasure my sister. You know my sister you feel like you are cured when you talk to another person.

M: Yes.

P006: The thing is, if people get close to you when you are in that situation, you then see that I am still important because you know that another person will tell himself that since I am in this situation, people will no longer get close to me, you see that type of a thing.

P006: You see, for instance since I tested positive in xxxx (year), it’s for the first time that I sit and talk about HIV, talking about my status.

M: Right, actually, thanks to you.

P001: It’s our pleasure.

M: Thank you.

A focus group discussion ends
