# Supplementary material for: “If we lose it, we are worried”: Individual and provider level perceptions towards weight change among people living with HIV who undergo TB screening in routine health care settings in Gauteng Province, South Africa
Source: PLoS One. 2025 Sep 22;20(9):e0331904. doi: 10.1371/journal.pone.0331904 (PMC12453174; doi:10.1371/journal.pone.0331904)
Supplement: S4 File — (ZIP) [file pone.0331904.s004.zip › S4 Transcripts_final/FGD 2.docx]

FGD 2

- **...** Ellipses indicate talk omitted from the data segment.
- **(( ))** The transcriber’s comments.
- **( )** Empty parentheses indicate some talk was not audible or interpretable at all (we include the line for instance 20:15)
- **(.)** A dot enclosed in parenthesis indicate a short silence
- **[ ]** Square brackets indicating beginning and the end of overlapping speech.

A focus group discussion starts.

M2: Hi, I am XXX [ Facilitator], I will be facilitating this focus group, yes, we have agreed that English is the language that you are going to use but if anyone wants to speak Zulu or Sotho both of us can understand so you can carry on in Zulu and Sotho. I am going to ask you to introduce yourselves and just tell us about yourselves but remember not to use your name, just say I am 009 and I work as a…, whatever you work as. Okay?

009: I am a xxxx (position)

008: I am xxxx (position)

007: xxxx (position)

006: I am a xxxx. (position)

005: I am a xxx (position)

004: I am xxxx (position)

003: I am xxxx (position)

002: A xxxx (position)

001: xxxx.

M2: Thank you, I am going to ask a few questions and we don’t have to answer in a raw, anybody can answer when you are ready to answer. When you go to a clinic and you make an appointment, when you come to the clinic for the appointment do the staff ask you about your weight, do they ask you if you have lost weight?

P: No.

P: Yes, they just check on the scale, ja.

M2: Okay, how many of you say that they don’t ask you? (.) Who have they never asked about weight? (.) There was one.

P: No, me, I check for myself.

M2: Why do you check yourself?

P: Isn’t it as they did it last month, I gained a bit, previously, this is where I was.

M2: So you check on the scale at the clinic?

P: Yes, ja.

M2: The gentleman here is the only one who has said that they don’t weight, everyone who comes here, do they weigh you? Do they ask you about your weight?

P: Ja.

M2: Why do you think they ask that?

P: They say, they want to see if we are losing or gaining weight.

M2: And why do you think that that is important for them?

P002: It’s important to take a scale so that they can monitor your disease.

M2: Okay, anyone else?

(.)

((A cell phone rings)).

M2: Why do you think they ask you about weight? ((A cell phone continues ringing)).

M: Can we kindly ask that maybe we can have our cell phones on silent mode. If you get a phone call you can quickly leave us and then come back after you have had that conversation on your phone, you don’t necessarily need to switch it off but please let’s have it on silent because we are using this gadget so it’s going to interfere with it, the recorder.

P007: I think they check the weight to see if…, because weight loss is one of the signs and the symptoms of TB so I think the whole point is to make sure that they check if you are infected by TB so that it can be treated quickly before it gets severe.

M2: So when someone who is living with HIV is weighed or if they ask you about your weight, how do you think that makes that person feel?

P007: Can you repeat the question?

M2: When you get to a clinic and they ask you about your weight, how do you think an HIV positive person feels when asked about weight? (.)

M: For instance, we have created these photos maybe before we even get to the question that xxxx (assistant moderator) is asking, we have this photo when you are coming to the clinic, we want to take you back to think…, on your way here, when you were coming here, knowing that in most instances when you come to the clinic, you get weighed just to check what your weight is. What was going on in your minds as you were coming to the clinic this morning, knowing that you are going to get weighed? How do we feel when we leave home on the date of our visit knowing that today we will be weighed? What goes on in our minds maybe we are still in a taxi, travelling to the clinic where we collect ARVs?

P001: Sometimes you just need to relax.

M: Uhum, what do other people say?

M2: So this is before you even get weighed, before you stand on the scale, how are you feeling?

M: In the morning, just when you are thinking to yourself, I am coming to an ARV clinic, I am going to be weighed, they will put me there on a weighing scale so that they can get a recording of my weight. How does that make you feel?

P003: I personally feel indifferent.

M2: Is it?

P007: I don’t feel intimidated at all, I know it’s one of the things I have to go through. I am not sure exactly how I feel after the weighing should it have an increased but I haven’t experienced it yet so I can’t comment on that as yet.

M: Maybe before we continue, are we all here in this group taking ARVs?

Ps: Yes.

M: Okay, all of you. Okay, he says he feels relaxed, number 3 says he feels indifferent, what do other people feel on the date, on their way to the clinic when they know that they will be weighed on that day?

P002: I am hoping that when I come here along the way, I hope that my weight must not go lower, it must go up.

M: Uhm so on your way, you are hoping and wishing that you gained weight. Should it be up or should it remain where it is?

P002: No, up.

M: Okay, all right, what do others say? What goes on in our minds, number 4?

P004: I wish that it could stay normal.

M2: When you say normal you mean the last time when you were last time so it mustn’t go down and it mustn’t go up?

P004: Yes.

M: Uhm okay all right, do we have somebody else with something different?

P006: It’s number 6 again, like before taking ARVs, they said you are positive and your weight was lost, so if you are taking ARVs, this is the amount of the effect of the ARVs on you, we must be expecting that.

M: Uhm okay, let’s what we expect once we start taking ARVs. Here, we have this photo here, we are waiting here to be weighed, you see we have tried to create a visual when we are waiting at the clinic before we could be seen by either a nurse or a doctor. What goes on in our minds when we are waiting there to be seen?

P: I beg your pardon?

M2: While we are sitting and waiting for the nurse in that waiting room like that picture shows, what are you feeling before you get weighed? 009?

P003: I wonder what other people are thinking.

M2: So you are wondering what others are thinking. ((Ps laugh)). Okay, anyone else?

P006: For me, what has been done is done so I don’t think of anything.

M2: You don’t think anything, you just sit and wait.

P009: I am hoping that my CD4 count will go up.

M2: Okay, you are hoping.

M: But otherwise how is the feeling? How are we feeling at that particular time?

P: I am supposed to be relaxed.

M: You are supposed to be relaxed, but are you relaxed?

P: Yes.

M: Okay, do we have somebody else with something different from that which has been said? (.) Okay because now I want us to think about the time when we are there on the scale, we are standing on this scale now, we are weighing ourselves, what goes on in our minds, what are we feeling at that particular time?

P006: Ja, at that stage is the time you think last time my weight was this, I want to see this time if I am improving or not improving and I must know the reason why because I am on treatment.

M: Uhm, improving or not, all right, what do others say, what do we think when we are standing here?

M2: 008, what are you thinking when you are standing there?

P008: I recall I once weighed 40, but now my weight is 60 something. I am very happy.

M: You have gained now?

P008: I came here when I had lost weight, even if I tell people they don’t believe me, the tablets made me ((to be like this)).

M: Uhm, we had someone who wanted to say something, I don’t know if it was number 3 or number 4 but between the two of you, I think you wanted to say something, what goes on in our minds?

P007: At that time on the scale, I just tell myself let me just feel happy even before you stand on this, it’s hot before to stand, even to feel my body, to feel my whole body is heating, to stand there, before to see even the number, to feel…, even last time 69, even today, you are gonna see 73 before even standing on the scale. To feel my body now, I was too fit, yes.

P003: To add on what he is saying, actually I feel optimistic, ja, I feel optimistic, I can already see that there is an improvement before I even see what the results say.

M: Okay, all right, after they have weighed you now, you have seen your results your status and all that, how do you feel?

P007: It’s just the same you just get relaxed because if you lose weight you will wait after sometime things are going to change.

P006: What I think after I take my scale, if I lose some weight I decide what should I change with my diet, the way I am living, just sort that thing.

M: Depending on the outcome, depending on what the scale read on that particular day?

P006: Yes.

M: Okay, lifestyle changes, what do others say, we have been weighed now, we know that this is what I weighed today, how do we feel?

P001: Lifestyle changes, you must take care of yourself, once you know that you have HIV, you must be very careful, you must take care.

M: When we say careful, what do we mean actually? What are we actually careful about?

P001: You must be careful with everything maybe let’s say you have someone you don’t know her and you want to sleep with her, you must take care, you can’t say you want to spread this disease, ja, you must take care of yourself.

M: When you see take care, do you mean protect?

P001: Take care means a lot of things.

M: That’s why I want you to clarify for me, what exactly you mean by take care? ((They laugh)).

P001: Ja, take care, just part and parcel of protect

M: Okay, all right, if you have lost weight, how do you feel on that particular day? Have we been weighed already or are we still going to get weighed?

Ps: Not yet.

M: We are still going that side. For instance, if we go there and we find that we have lost weight, I believe that this is not the first time you are coming here, you have been coming and you are already familiar with the procedures, if you go there and you find that you have lost weight, how do you feel on that particular day?

P009: I feel that because I lost weight, I must start thinking so that I do not lose weight (further).

M: You mean the cause, what is causing you to lose weight?

P009: Yes.

M: What about other people?

P003: I have not experienced it as yet but I will feel worried, I would panic.

M: What will bring about concern or panic as such, what is it that will make us to be concerned or worried if we have lost weight?

P003: I think the main panic will be…, I would assume that probably the drugs are not working as they should be, so there could be a possibility that I might either develop resistance.

M: I see, I get you, what do other people say? When you stood on the scale on that particular day and you heard that you had lost weight, how did you feel on that particular day? What was going on in your mind?

P006: I felt the same thing, you can’t be happy because you are losing weight, yes, you have to be normal for you to gain weight at least you are going to see that things are improving. If you are losing weight that may mean that drugs are not good to you.

M: And then on the clinic where we went to the clinic and we found out that we gained weight, how did we feel? (.) For instance, I am going to ask number 8, you told us that the other time you were 40 and then you gained weight, what was going on in your mind when you learnt that you were 40 at that particular time. ((P asks M to speak in vernacular language)).

P008: And now I am okay.

M2: When you weighed forty- how were you?

P008: I was worried.

M2: Why were you worried?

P008: ((Not clear)).

M2: ((Not clear)).

M: Okay, let’s hear from someone else amongst us who gained weight or someone who picked it up? What went on in your mind when you saw that you were gaining weight?

P007: I feel happy.

M: What made you happy?

P007: Gaining my weight, ja.

P008: Your mind tells you everything so that you know you have gained the weight, so you must be happy.

M: So we become happy when we gain weight?

P009: You can be happy but you do not know what is actually happening, let’s say you were weighting 60 and now you are at 70, you must ask yourself what is happening between that time, how come am I weighing another 10. It must be a worry to you.

M: So in other words we are happy that we are gaining weight in some instances we are concerned as to why it shoots from 60 to 70.

P009: Exactly.

M: Ahh okay.

P006: But the worry is better to gain than to lose. 50 to 40, 40-30.

M: We are from the ARV clinic here, we are leaving now, everything has been done, how do we feel in most instances? This woman is from the ARV clinic, we are just making the visuals in most instances, when you leave the ARV clinic going back home, how do you usually feel?

P009: I am feeling proud because I have got something to defend myself with.

M: And what is that?

P009: The pills.

M: All right, what do others say? Other than that, how do we feel after having been to the ARV clinic, after everything has been done on that particular day?

P006: It depends, if everything goes according to plan, let’s say maybe you weigh 60 but that day you weigh less, on my way home, I am going to think what have gone wrong, what am I supposed to do now, I must get those answers.

M: Uhm depending on that outcome of what that particular visit was. Okay. You can continue ((M leaves the room to ask people next door to the FGD venue to speak softly as they are disturbing the group)).

M2: Let’s think of someone who is asked about weight loss, when you go to the clinic and they ask you about weight loss and they ask you, what do you think has happened and you say you have lost weight. So, if a person who is HIV positive says, they have lost weight, what do you think that means?

P006: Mostly, the diet, secondly too much stress, thirdly, maybe you are not responding to the tablets.

M2: Okay, any other answers?

P003: I think the person may not be adhering.

M2: What does adhering and not adhering mean?

P003: Not sticking to the way, they should take their drugs on time. So I assume that they might have developed some form of resistance and so the drugs are not functioning and then it has resulted in weight loss.

M2: Okay, okay, any other answers? (.) Does the size of your pants change or your shirts, when you have lost weight, is there a change?

P006: Yes, there are some changes mostly on your face, these areas ((says P pointing at his face)).

M2: You see with your face first?

P006: And your shoulders.

M2: Face and shoulders.

P009: I see by my trousers, if they are becoming big because I am losing weight.

P003: Maybe on the waist.

M2: What size were you before and what size are you now?

P009: I was 130 now I am 127 ((the door opens)).

M2: Do you think there is stigma…, do we all understand what stigma means, is there stigma attached to HIV positive people when they lose weight?

P003: I think so.

M2: You think so. Okay, can you tell me why? What makes you think there is stigma?

P003: Because the general assumption with people is that HIV means weight loss and death and so the moment somebody looks at you and tease you with those words, they basically put a lot of conclusions like this person is going to die.

M2: Anything else, what kind of…, what other stigmas have you experienced from people because of weight loss?

P001: People are talking behind our back.

M: What are they actually saying?

P001: They say, you see that one is a carrier.

M2: So they call you names?

P001: They call me names and they don’t want to communicate with me.

M: Uhm.

M2: Why do you think they don’t want to communicate with you?

P001: They say I will infect them.

M2: Anything else?

M: The gentleman said, they call them names, what names do they call us?

P006: Mostly if they know that you are positive through appearance, the loss of weight, they start ignoring you and mostly the names like killer.

M: My goodness, who is a killer?

P006: A person that has the virus, they can see you losing weight and they think you are HIV positive, there is a killer, ja, so most of the things, you are gonna see that life is changing for you, that’s why you see most of the people, they are always losing weight because of too much thinking or more partners or friends, they will say someone is positive cannot be your friend.

P001: Most of them think that you are an AIDS carrier, when they see you, they say, that’s it, then everybody is ignoring you, they don’t want to be near you because they say you are going to infect them with AIDS. I think if you talk with them and you say even you may be having AIDS because you didn’t go and check.

M: Uhm but you are assuming you are HIV negative.

P003: Due to the appearance ((says P and laughing softly)).

P006: Because of the appearance, this one is maybe positive.

M2: So they judge you based on appearance.

P006: And as well who is being judge, you know as well that you are positive.

M: Uhm when we talk about appearance, are we only referring to weight loss or is there any other thing other than the appearance which is due to the weight loss?

P006: You know the symptoms of AIDS, weight loss maybe some skin disease or even your face, some wounds on the mouth; they will be away for just one and back again. So most of the people think these are the symptoms of AIDS.

M: Uhm, so the names, you said they call you a killer, an AIDS carrier, what other names do they call us in the community? It may not be the names that they call us, or directed as us, it maybe that we are from the community but we know that in the community for instance, we did not disclose that we are HIV positive but I hear a person coming maybe to talk about somebody else who is known to be positive, what do they usually call those people.

P006: Any name that related to killing, they are just going to call somebody or even at night they can call you a gun.

M: They call you a gun?

P006: Ja, you are a bullet anything that can kill you.

P001: There are many names.

M: These are the names we are looking for.

P006: Z3, Z4, *ukhwekhwe*- it means rash.

P001: People think you are going to die.

M: Uhm, at this time and age.

P006: Others they say, you get amazed. Others say you are amazed.

M2: What do they mean?

P006: Did you see a person who has been affected by HIV or AIDS, he can’t even talk hey, he is just staring at you like this. They are amazed.

P003: ((35:22 not clear)) you are amazed.

M: Amazed, very interesting.

M2: And how does that make you feel when people say these things about HIV positive people?

P001: You feel like bursting to these people.

P006: We people are different, others feel bad and others can say, it’s my turn and tomorrow it’s you.

M2: So some feel bad and some are calm.

P006: Calm.

M2: When you feel like bursting, bursting on who?

P007: To the one who is calling you a name, those names?

P008: I get scared when they say that.

M: You get scared.

M2: Anyone else? (.) How do you feel?

P003: I feel upset but then I just let it go because I know it’s ignorance.

M: Uhm. What upsets you initially?

P003: Uhm, I think it’s the lack of knowledge.

M: Okay.

P003: Not the name itself, I get upset with a person’s lack of knowledge.

M: Uhm projected through behaviours?

P003: Ja.

M2: Think of someone who is attending HIV care, what do you think their weight should be? (.)

P006: Come again.

M2: Someone is attending HIV care, what should their weight be?

M: A person attending the ARV clinic, what should their weight be?

P006: It depends on people but mostly normal at the age of the 40s, they must be 65 and upwards.

M2: Are there any other responses?

P003: From what I learned from the dietician, I was told it actually depends on a person’s height something that they refer to as a Body Mass Index. It depends, if you are short and then you weigh 70kgs, you are actually overweight but then if you are 70 and you are very tall, you could still be underweight. So the actual number doesn’t really matter. It actually depends on the height and all that.

M2: Okay, any other thought? (.) And the shape, what should the shape of the body be?

P002: The normal size.

M2: What is the normal size?

P002: Not very thin and not too fat.

M2: Not too thin and not too fat. Anyone else? Number 7, what do you think?

P007: In- between.

M2: In-between?

P007: Yes.

M2: What does in-between mean for you?

P007: Not too much fat.

P003: For me a normal shape would be, I should not see the collar bone and I should not see the cheek bone and I think the skin should be elastic, it shouldn’t be]

M2: [When you pull it up then it stays.

M: Okay, we brought these photos for you ((Ms speak to each other)). Here are our photos, we want all of you to see them. Can I give you this to have a look at and pass on, they are 9 just like us ((Ms are sorting out the photos)) what do we say then about these photos that we have?

M2: So let’s think about someone now who is on HIV treatment, if you look at the pictures which would you say a person’s weight should be? Which weight looks like what you think is normal?

P006: The one I am holding, I don’t think is normal.

M2: Let me see yours?

M: That’s number?

P006: Number 3. I don’t think this is normal.

M: Let me see at the back, I just want to check the number, it’s number 9.

M2: Okay.

P008: And this number 8 is better.

M2: It’s better. Okay so you think someone can look like number 8?

P008: Ja.

M: What do others say about number 9? Do we share number 3’s sentiments with regard to number 9?

Ps: It’s not okay.

P006: He is okay because he can do something to lose weight like he can start training and stuff like that but this one is bad.

M: That’s number?

006: Number 2]

P003: I think what he is saying, it’s okay to be like this since all this person needs is an exercise but it’s actually]

P008: [Overweight]

P003: [[I personally think that he is overweight, that one is not right, even if he can try to lose weight, he is not going to be like that, he is going to have a big stomach still because he is already overweight.

M2: So which one is the one which we think looks the best?

P001: This one I think is the best.

M: It’s number?

P001: Number 3.

M: Okay.

P006: And 6, this one is better.

P001: I think this one is better.

M: What number is at the back?

P001: 5.

P003: I think it’s number 5.

M2: How many people think number 5 is better?

M: Number 5 also thinks it’s number 5 and these]

M2: [5 of them].

M: You don’t have to choose the one that is in your hands, you only have to choose that which you think is the right one.

M2: So 5 people say that number 5 is the best weight to be.

M: You were saying something about number 7?

P006: Number 6.

M: You will go for number 6.

P003: They think that number 6 is the best.

P006: If you can look number 6 here and here he is fat, it’s better from here downwards. He is shapeless.

M: But one thing that we should understand is that when we are in a group we might have differences in opinions and we will have to accommodate one another on that one.

M2: But 5 people said number 5.

M: You were saying something about number 7, number 2?

P002: I was saying it depends on the body of the person, if he is in the 40s it will be otherwise better.

M: You think number 7 is better?

P002: Uhm.

M: Who agrees with number 2, that number 7 is better?

M2: What about the shape, which shape looks good?

P003: I will still stick with number 5.

M2: And why would you go with number 5?

P003: Because there are some signs of muscle there, it’s definitely sure that there is some exercise being done there.

(.)

M: What do others say?

P001: Even his shoulder, you see, it is well build.

M2: Okay, anyone else?

P008: Number 5, I also like him.

M2: All right (.) Anyone else who thinks another shape is the best?

M: Other than number 5?

P001: This body is fine for me.

M2: What number is it?

P001: Number 3. I don’t like a big body, I like to be tiny.

M2: Okay, what changes could happen to a person’s weight if treatment is going well and if it’s not going well? ((Repeats the question)).

P009: If my treatment is going well I will not lose weight but if my treatment is not going well, I am going to lose weight.

M2: Does everyone agree with that? Any differences?

P006: Sometimes when treatment is not going well, you can develop maybe some big breasts, something like that.

M2: And why do you develop those?

P006: I don’t know but it’s because of the treatment.

M2: Okay, any other thoughts? (.)What happens to your weight when things are going well?

P006: Ja, it goes up to the normal size.

M2: Back to your normal size, ja. ((A cell phone rings)) and what happens to your shape when things are going well and when things are not going well? When treatment is going well and when it is not going well? (.) The shape, how does it change when the treatment is going well?

P007: I think once is going well, the shape might not change.

M2: They might not change, okay.

P003: I think if the treatment is going well and you are eating properly, I think you gain more flesh.

M2: Okay, and if it is not going well, how does that affect your shape?

P007: You get skinny.

M2: You get skinny, is that all that happens? (.) And the ideal weight and shape, the one we say it’s the best, do we think it should be the same for men and women who are HIV positive?

P006: The men must have the same weight?

M2: Is the ideal weight for man…, if number 5 is the ideal weight, should it be the same for men and women or should it be different?

P003: I don’t think so. I think it should be different.

M2: Why is it different?

P003: Because women’s bodies are…, I think they are normally designed to carry children so even what goes on in their blood and their periods, there is a lot of…, it’s different from men, there is a lot that happens in women’s bodies so I don’t think it should be the same. Normally, you would expect that if a woman gets pregnant, the normal sign will be fats, the hips, so I think there is a difference. There should be a difference.

M2: So women should be heavier or lighter?

P001: Heavy.

M2: Heavier because they are designed to have kids and all that. (.) And your thoughts on what is the best shape and the influences, what are the influences, is it the community, is it our dietician, is it what you see on TV, what influences your thinking?

P003: About?

M2: About what is the good weight and shape?

P006: I don’t understand that question?

M2: So you have all said number 5, 5 of you have said number 5 is the best shape and weight to be, why have you decided that? Is it because people in the community say so, is it because TV advertises that person to be best, is it because your doctor says that’s the nicest shape to have? Why do you think that’s the best shape?

P006: It’s because usually they are normal.

M2: Normal according to what?

P006: Someone with HIV, what I can say if you are taking the drugs for HIV.

M2: And where have you see that that is the normal shape?

P006: Like myself I was like that sometime ago.

P001: I think it’s the doctor’s suggestions, it will tell me if I am okay or not because he knows what weight I must weigh.

P003: I think influence in terms of being muscular has a lot to do with media. TV gives you the suggestion that someone who is muscular is more acceptable to the community, something like that but in terms of body size not referring to the muscles, the influence is from what experts say, the doctors, dieticians.

M2: They influence your thinking on size.

P003: Ja, on size.

M2: Size being, the weight or the shape?

P003: The weight.

M2: Okay, what do you think the actual reasons are for HIV positive people losing weight? What are some of the reasons; if we were to just list them?

P001: Poor diet.

M2: Some other reasons?

P006: Stress.

P007: Not sleeping well.

M2: Okay. Any other reasons?

P003: Other infections and stress.

M: And how does stress lead to weight loss?

P001: I think you think something bad is going to happen to you. You focus on one thing after the other, after this, after this.

M: Uhm and how does that make you lose weight?

P001: I think I start not doing normal things which I should have done; you see because I will not eat, I will not drink because I think of many things in life.

M: You get so pre-occupied such that you forget eating then you lose weight.

M2: Any other reason why HIV positive people may lose weight?

P005: Not taking medication.

M: Which medication?

P003: If you are HIV positive, I don’t know the chemistry behind it but I know that if you are HIV positive and you are not taking any medication and there is muscle wasting, I don’t know if the virus itself causes the muscles to be wasted and disappear.

M: Number 3, muscle wasting. I am just noticing that we have not been mentioning our numbers; it might be difficult for the person who will be transcribing who may not be here with us. You called it muscle wasting?

P003: Yes, muscle wasting due to the virus. Once you are on medication, the virus is suppressed so that muscle wasting no longer occurs. I don’t know the chemistry behind it, I don’t know how it works but I simply know that once you are HIV positive, you are just going to see the muscles going away unless the virus is suppressed. So if I go back to what we were talking about earlier on, that’s where the assumption comes from that if a somebody loses weight, in my opinion it means the virus is no longer there, it’s suppressed, it means that either the drug is resistant, you are not taking the medication properly or that they are not on drugs at all ((not clear)).

M2: Okay, sometimes we go to the clinic and you as an individual feel that your weight has changed, the scale shows that there has been no change, why do you think that happens?

P001: The body has changed but the scale does not change.

M: When you say the body has changed, what do you mean? The weight has changed but the scale did not? What do you mean about the change of the weight? (.)

M2: Say for example, you arrive at the clinic and last time you weighed 65, you believe that you now weigh 62, so you believe that you have lost weight but when you get there the scale does not show that since it shows 65. What could make you think that you have lost weight?

P006: ((Asks for clarity)).

M2: You think that you have lost weight, so your weight is 65 and you think it’s less before you go and weigh yourself but actually nothing has changed. When you get on the scale, you see it’s still 65. (.)

M: For instance, a health care worker, either a doctor or a nurse asks you did you gain weight, you say I lost weight, the last time they weighed you, you were 65 and they are asking you did you gain or did you lose. For instance, you say you have lost weight but the scale is still at 65.

M2: So what will make you think you have lost weight when you haven’t?

P003: I think if you follow a certain lifestyle during the period between…, like for example if I weighed a month ago and then during that time between a month ago and now and I am about to weigh, I know personally that my lifestyle has not been what it should be, what is expected of me.

M2: Give us an example?

P003: Maybe I was smoking and drinking, I was maybe eating once a day and then normally the first thing that will come to your mind is that I know, if I step onto that scale I would have lost weight.

M: Uhm behaviour preceding the clinic visit.

M2: Any other thing that will make you feel that you have lost weight and you haven’t? (.) What else will make people lose weight?

P003: People’s opinions.

M2: Okay.

M: What about people’s opinions?

P003: If somebody comments, you look like you have lost weight.

M: That’s still number 3, what do other people say?

P007: Eh physical appearance.

M: What about physical appearance?

P007: You see when I look in a mirror; I look like I have changed.

M: What in the mirror will make you think that you have changed in a manner which makes you think that you have lost weight?

P007: Maybe I look a bit skinny.

M: Looking a bit skinny.

P003: Such that you start to notice the cheek bones.

M: What else, what else makes us to think that we have lost weight but find that it’s still the same when we are standing on the scale, even after we have told ourselves that lost weight and told the nurses and counsellors that we lost weight? (.) What about your clothes, what happen to the clothes?

P001: They do not look good on you.

M2: Have they become bigger or smaller?

P001: Bigger.

M2: And what happens in situations where you think you have gained weight but you haven’t, the scale does not say that you have gained weight, what makes you think that you gained? (.) You are tired right? ((Ps agree)). ((M asks for refreshments)).

P003: As for gaining weight, I think you would have tried to eat and then you would have taken that effort to try and gain weight by making sure that you eat three times a day]

P002: [You eat meat also and stuff like eggs; you are actually actively doing something to gain weight. I think that is what makes you think that you gained weight.

P009: It means I must eat well so that when I go to the clinic, my weight must be normal but if I see my weight is not normal I must ask myself, what’s happening maybe something is wrong on my diet or on my lifestyle, that is when I say I think that I didn’t lose weight, my weight is normal.

M2: And what will make you think you gained weight?

P009: It’s when I eat the right food and I start looking at the clothes that I am wearing; I think I wear like a size 10 then 11.

M2: Are those the only reasons.

P003: Also if you feel that your clothes have gotten smaller, you start to feel that now the clothes are tighter on your body and the assumption is that it means I have gained weight.

M2: Okay. In your experience what’s the best way to ask an HIV positive person about weight gain and weight loss?

P001: Come again?

M2: In your opinion, what is the best way to ask an HIV positive person about whether they have gained weight or whether they have lost weight? (.) Okay, let’s see, here is an example if you go to the clinic and the nurse says have you lost more than a trouser’s size unintentionally in the last 6 months? Do you understand that question?

P001: Yes.

M2: Would that be a good way to ask you?

P002: No.

M2: You would say no, why not?

P001: Ja, it’s a good question to ask.

M2: Why do you say it’s good.

P006: ((Not clear)).

M2: Okay, any other thoughts?

P003: I think the question should not be specific, I think it should be, have you lost any weight.

M2: Question should be, have you lost any weight. So all this more than, less than]

P003: [Ja, I think it’s easier to say if it’s just like any because it’s not specific about a certain number or perhaps it’s a loss or gain, have you lost any weight and then your reply ((response)) will be yes or no.

M2: You will prefer that because by not being specific, it gives you the opportunity…, what does it do?

P003: If it’s not specific it becomes more intimidating because what happens is, you don’t want to appear as if you have lost too much weight or you are, ja, as if you have lost too much weight. ((M2 gives over to M who has brought the refreshments to the FGD venue)).

M: Do we understand that question, if a person asks you if you have lost more than one dress size ((M2 explains to M what has been said)) the question is, have you lost more than one dress size, do we understand that to begin with?

M2: They understand that but they don’t want to be asked that way.

M: Okay, can I finish, do you understand, when you go to the clinic, the health care workers ask you if you have lost more than one pant’s size? Do you understand what that means?

P001: We are lost now.

M2: Now, you have confused them ((she laughs)). If someone asks have you lost more than one dress…, pant’s size unintentionally?

P003: I will express it in my own words, how I understand it.

M: Okay.

P003: What I understand is, what they are asking me is, since the last time I weighed have my trousers become loser like if I was size 32, have I dropped to size 30 or 31 that’s more than a trouser size, for example if the trouser’s size are 30, 31, 32, 33, 34, 35 and so forth if my trouser’s size was 35 if I have gone down to the size 30?

M: All right. This is what number 3 understands okay.

P003: By unintentionally, meaning without being actively involved, without deliberately doing it like without for example you know I was not eating or I was not eating on purpose so I have lost weight but I have no idea how it came about so if a nurse is asking me, have I lost a trouser’s size without knowing what happened whatsoever because even if they ask me, I don’t know what happens.

M: Okay, so this that number 3 has just explained, do we understand it in the same manner or do we have someone who would say when they ask me that question I used to understand it differently and if that is so, how different is that from which he has just said?

P001: I am worried because why are you asking me about my trouser’s size; it’s the first time you are asking me that, what wrong?

M: But you have been asked this question, isn’t it? (.) You did get this question when you were attending or are we coming across this question for the first time? Is it the first time that we are asked this question or has it been asked before? As number 3 explains it that]

P003: [I once come across it.

M: Okay, so my follow up question then will be explaining it in the manner that you just explained, is that how the health care worker explained it to you or was that your simple understanding of the question?

P003: That’s my simple understanding and also want to say that, I think that way of questioning is intimidating.

M2: That’s what they said.

M: If we say intimidating, in what way?

P003: It might mean that people are assuming a lot of things about you, the weight you have lost is too much, so it implies that you have lost too much weight.

M: So you don’t feel comfortable being asked that question, the question I think should be kind alike rephrased to something not specific about sizes.

M2: I have written that one, we spoke about that.

M: You did come to how it should be phrased?

M2: We have done that.

M: Okay so what do others say, can you understand what the drop size mean, from what he just explained we brought these jeans, this is size 30 right and this is size 32 and size 28. If a person says you have dropped more than one pant’s size from these that I have, you moved from which one to which one? Your understanding, you move from which one to which one. We have 28, 30 and 32.

P006: Between 32 and 30.

M: That is number 6’s understanding, what do others say?

P001: 27 to 28.

M: Do we have 27 here? I want us to make examples with these that we have here.

M2: You said moving from 28 to 30.

P002: 28 to 30.

(.)

M: What do others say? To lose more than one pant’s size will be like losing weight from 32 to 28, isn’t it you see, they are following each other, 28, 30 and 32 meaning if you lose from 32 to 28, it’s more than because you have passed 30 that is what they mean at the clinic when they say that, the same as you were explaining it earlier on. Do you understand it now, but I was supposed to ask you to illustrate that but he did explain how it works nevertheless. ((Refreshments were served)).

P001: I think we can carry on; I am not sure about others.

M: Okay, I am going to it here neh and here is the drink neh. I am going to put it here, we are all men here, let’s just help ourselves. Let’s continue, I would like to know how the following people will respond to a person living with HIV who has lost weight for instance our spouses, I understand that most of us here have partners for instance when our partners or the partners of people whom we know are HIV positive have lost weight how have been their partner’s response? You can either talk for yourself or you can talk about the close person whom you know has HIV and has lost weight and whose partner has commented to the weight loss?

P001: I am talking about me, my wife asked me, why are you losing weight because you are eating well, what’s happening with you because I was scared at the last minute I told her too and then they always say just go and check, let’s go and check.

M: What is he checking?

P001: Let’s go to check at the clinic maybe you have got TB basically they are taking about TB.

M: Okay, the partner was concerned and asked you to go to the clinic to test for TB. What do others say? What were our spouses’ and partners’ responses towards us when they noticed that you are losing weight?

P004: My spouse, she also has a virus so we used to come together here.

M: So?

P004: Last month, we were aiming to make a baby then they said we must come to you and they must tell us what to do here.

M: Okay, all right, so has there been an instance where your partner had asked my husband I think you are losing weight?

P004: No, actually, I am not losing weight at the moment my body is just like this you see. My body is always like this.

M: Okay, what do others say?

P006: With me I just wondered, you are just losing weight, what is the reason? I am the one who noticed that on my partner. We came to the clinic since she also had some body pains. You are positive so that’s the reason.

M: What do others say, it was the other way round for the gentleman, he is the one who noticed the body weight loss on his partner and that led to them getting tested and becoming aware of their status. What have our partners or our friends’ partners said or commented upon our weight during the time when we were losing weight? (.)

P003: My partner is very open, very very open, the time when I got ill she suggested that we go and test.

M: So was it weight loss or what?

P003: Weight loss was included.

M: So you talked, came and got tested. Do we have someone who wants to something else about what their partners have said about their weight loss?

P001: After testing for TB, they said we must go and look for the results when we got there, they said do you prefer to go and test or ((not clear)). I was not ready, she told me let’s go, what are you scared of, let’s go, if you don’t go, people will talk behind your back, then we went there, got tested. She was negative; I was positive and started getting worried, what is happening, they said no, that is that. She didn’t comment anything, she didn’t say anything, she just said let’s go, go and take your results.

M: Okay, all right and then the children, how will children usually respond when they see us having HIV and losing weight?

P001: My children are too big, 32, 42 year old but I told their mother to tell them because if I am going to tell them, they are going to shout at me.

M: Oh okay, so you did not tell the kids?

P001: I did not tell the kids. She told the kids and then she came and told me, why didn’t you tell them and you send me and I said, you must tell them. This is a sickness, you are sick, why do you say you want to hide your sickness.

M: So our children want us to be open and talk to them, what do others say? What responses would you expect from the children or the kids when they see us losing weight as a result of HIV? (.) How do we expect our children to respond when we lose weight as result of HIV or the people that we stay within the community, share with us that which we sometimes hear, to say children respond in this manner and that manner after we lost weight as a result of HIV? (.)

M2: How do you think your family, your extended family will respond because of a loss of weight?

P006: I think my sibling, I worry about my family, they must first know than everybody who is asking me anything until they can respond and tell them that this man is sick. (.)

M2: Anyone else, how has your extended family responded?

P001: My whole family supported me, they do support me, they say man don’t worry, it’s not the end of the world.

M: Uhum number 1 got support from the extended family.

(.)

M2: And friends, how did they respond to weight loss?

P001: They start calling names first, they say hey slender, that’s the name they give us, what’s eating you? Nowadays, this sickness is no more, what can I say, you tell them straight, *ei* I am going to the clinic but it depends. My goodness, he will die anytime from now.

M2: Others, responses from friends?

M: What did our friends say when they saw us lose weight as a result of HIV, what did they say or how was their response towards us?

P001: My friends respond to me the same way as when I was not sick. They just say *((name withheld for confidentiality reasons)) keep it on and I said to them hey ((name withheld for confidentiality reasons)) you must keep it clean; you must not come to the clinic there. I don’t want to see you there at the clinic; you must take care of yourself.

M: When you say you don’t want to see them at the clinic, you mean you don’t want to see them getting infected?

P001: Yes ((responds P with a soft giggle)). Sure, yes.

M2: And the community? (.)

P008: The community, they don’t care anymore because they know, half the population in the community is sick. Everybody knows, they are no longer talking about this disease. They are talking about high blood, TB, cancer; they don’t talk about this disease anymore.

M2: Any other responses?

M: What do they say in the community when they see us lose weight or if they see someone in the community lose weight as a result of HIV? What do they usually say?

P002: They say by the way, this one is dead.

P006: They say, he is a moving corpse.

P003: They say you are juicer?

M: What is to juicer?

P003: You are burning the airtime ((Ps laugh)).

M: Okay.

P003: If you take pills it means you are juicer.

P002: It’s like secret, if you are talking, if you are two eh my friend go and put airtime and phone me, you will know that you are going to take the tablets.

M: Oh, that’s a secret language for people that are infected to remind each other.

P006: Not to set the alarm, it seems like here, now it’s 10 o’clock hey my brother go back and take my phone there, they are telling you to send you there, you know what is next.

M: Okay, interesting.

M2: And health care workers, doctors and nurses, how do they respond when you have lost weight?

P001: They will ask you, what’s worrying you. You are starting to lose weight, you were coming along okay but now you are losing weight, tell us the truth, tell us what’s eating you?

P003: The health care workers are concerned.

M: They are concerned. For them to show that they are concerned, what do they sometimes do?

P008: They encourage you to use the medication according to the description

M: Okay, so they ask you to comply, all right, anything else, responses from nurses, the doctors and those of counsellors at the clinic when they notice that we are losing weight? (.)

M2: You as an HIV positive person, how would you feel about the responses of all the people we have spoken about?

P002: Which people?

M2: Community, friends, family, the extended family, children, health care workers, how would you feel?

P001: I am not worried so long they know.

M: How do we feel when they ask us what is eating us and calling us by all these names that you have mentioned, how do we feel now that we are HIV positive?

P001: When a person speaks like that, I feel ashamed.

M2: Why?

P001: It confuses me because I must focus on getting better.

P003: Usually, when they tell you that, you already know you are losing weight, when they talk about it, it’s a way of teasing you. It’s something that you already know.

M2: How is everyone feeling, tired hey?

P006: It’s just that it’s the matter of time and we still need to go back to work and I will need to make another time since I still need to see the doctor.

M2: Okay, everyone else, how do you feel regarding time? ((They speak but it’s not clear)) Okay someone needs to go see the doctor to collect tablets?

M: Oh by the way your manager took your files aside when you leave here, you will need to go to her, she said she will collect your stuff accordingly.

M2: Okay, let’s talk about when you have gained weight, how does your spouse respond?

P001: When I gain weight?

M2: Ja.

P001: She starts to smile.

M: Ahh.

P001: They are taking their medicine; they are eating all right now because the problem is liquor and cigarette. Those are things that make us to do stupid things.

M: What do other people say? Once we gain weight what are their comments in most cases?

P006: The comments will not be bad.

M2: And comments from children when you gain weight?

P006: My own children or?

M2: Your own children and children in general?

P006: The comments are not bad.

P001: They will say, you see now, they are taking the medicine, you see, they are improving now.

M2: Number 3, you wanted to say something?

P003: Me and my spouse normally joke about these things in a nice way, like when I gain weight, normally I call the virus the Ninja and we will have comments like ja, a Ninja is in jail. In a joking way so she teases in a joking way though, in a nice way. Or that the Ninja is drunk something like that.

M2: That’s for your spouse?

P003: Yes.

M2: Okay and the extended family? (.)

M: What about your aunties?

P001: They will say listen, now you have become a better person.

M2: Any other responses? (.) And friends, how they respond when you gain weight?

P008: Most of the members do not know what is making me to lose weight because I can’t tell them.

M2: Why can’t you tell them?

P008: Ja, people are different to others the people can just say, this one is positive so sometimes it will be me and my family only.

P001: To me they will say, you are becoming healthier what are you eating. Then I will say I am eating ((not clear)) they will say can you give us because you are starting to gain weight. I say if you want them go to the clinic. They will give you because the comments are not the same, others say other things.

P003: When friends start noticing I gain weight they comment that my wife is looking after me ((says P with a giggle at the end)) that’s what they say.

M2: And members of the community? (.) Members of the community, what do they see when they see you gain weight?

P007: The members of the community sometimes will notice that you are losing weight and the moment you change they start to be happy.

M2: How do you see that they are happy? What would they be saying?

P007: They will say that you are now fine and also gaining weight as compared to the last days, when you were losing weight like I was saying that they can’t tell that you are positive, when you were just losing weight time after time after time you are gaining weight. They will ask what is involved, I tell them no, just ask the doctor to check your body and status, those that we see.

M2: Health care workers, how do they respond when they see you gain weight?

P003: I have got different responses from the health workers, one time one nurse said it’s nice keep it up and then one time…, sometime they will just be indifferent like it’s what they expected anyway. So they just don’t]

M2: [It doesn’t change anything. And how do you feel about responses you get from people around weight gain?

P002: Ja, you feel better, happy.

P007: Good comments, they will always make you happy.

M2: Number 9, you were saying something?

P009: No, he said it.

M2: Okay, is there anyone else who wants us to add anything? (.) Let’s talk about body shape quickly and how various people will respond, so how does your spouse respond when your shape changes.

P006: It depends, how the shape of your body is changing. It changes nicely and it changes badly at times.

M: When it changes nicely, give us examples?

P006: Nicely, you see the normal body, you just see someone in the community with your normal body, when you are losing weight, you will know that you are losing weight. If you gain weight, you will know that you are gaining weight and you see that this one is normal and you can see that they are fine.

M2: So for you it depends on what is happening, okay, others? (.) Okay how do children respond when your shape is changing?

P009: They always say to me, the pills are giving you power or your shape is going back to normal, the way we know it.

M2: Anyone else for children and how they respond?

P007: They start to comment.

M2: Saying?

P007: Now, you are looking better.

M: We are still taking about gaining weight but we are now on body shape which has changed as a result of ARTs? For instance, we have heard that sometimes the bodies of people with HIV change. When people start having the virus the bodies change and I think number 6 was explaining that it depends on how the body changes, whether it changes nicely or whether it does not change in a desirable manner. The question now is, when the bodies of people that are living with HIV change, how do they usually change? We want you to describe to us how that happens and how that presents itself to say the body of this particular individual has changed either as a result of HIV itself as a symptom or either because of the medication that a person has already started taking? (.) How does it change?

P009: I think it’s about medication. If you start taking the medication, let’s say they used to call me slender and now that I have changed, they are starting to say yes, you see the medicine is improving you. Keep it up. Don’t look back. Even the children, they always say keep it up.

M: We are talking about the body changes, for instance, we have heard that people at some instances, we don’t know if you have heard about this may develop fat gain here in the abdomen, other develop a hump here at the back of the neck and others will lose fats on the face and other people will lose fats on the buttocks and some people will lose fats here on the legs, you will hear people saying their legs have become thin, they have changed shape. Sometimes some people say, it’s a dislocation of fats or you will see a person developing a big stomach which they used not to have before. Have you heard about that?

Ps: Ja.

M: Sometimes you hear people saying a male has developed breasts? Is it common? (.) Do we come across that often in the communities? How do people usually respond to those changes that have come about as a result of ARVs?

P001: They are scared to ask.

M: People are scared to ask.

P006: Yes, because you cannot ask what is going on in the people’s heads.

P001: You can only find out if they tell you, then you can respond but if they don’t tell you, you cannot know.

M: Okay, what do others say? How do people usually respond to these very obvious changes? (.) Maybe you hear that a person used to have bums and all of a sudden there are no bums maybe women but you find that instead of here, the fats are here?

P006: You see, most of the days, when people see your body is changing, they just assume that you are taking ARVs. Some of the people, they know that, like they hear that we are in a meeting like this for instance, we are gaining something from this meeting. If we go out we are going to see someone with those things. I can keep a secret, I can tell my friends, I think this one is taking some ARVs because of this and that. The one I am telling cannot keep a secret, spread the news and more people know now.

M: So in general, once people start presenting with those, how do you usually respond? Once people start presenting with lipodystrophy, dislocated fats?

P006: Mostly, they go to the doctor.

M2: They don’t discuss it in the community?

M: In the community, they don’t point fingers at you once you start developing these signs?

P001: They will point fingers at you, isn’t it they will know what is happening to you but they will not tell you directly, they gossip.

P003: Yes, they gossip.

M: Oh they gossip, okay, what about friends? Have we heard instances where we have friends in the community and all of a sudden, we see our friends’ bodies changing drastically? Have you heard that? What have been other friends responses regarding that to those particular friends that have changed as a result of ARVs?

P001: They will always ask questions. They do ask questions amongst themselves, what is happening to *((name withheld for confidentiality reasons)).

M: So they ask questions but not that particular individual.

P001: Not me.

P007: Yes, they gossip. What’s wrong with me.

M: The extended family members, your aunts, your grandmothers once they see maybe I used to not have a big stomach and all of a sudden they see me with big stomach or they know me with hips and bums and all of a sudden I have a flat bum. How do they usually respond to those changes?

P008: If those family members know that you are using ARVs, that’s why I was saying sometimes it must be a secret because those extended families sometimes are dangerous, they are going to start telling each other. You see this one, his body is changing, he is now having some buttocks because he is taking the ARVs so they start to see that before you started ARVs, there was nothing. You are now taking ARVs, sometimes keeping it a secret between you and your wife is better. You and your wife, your wife must know but those extended families like uncle, your cousins and you know they don’t like because they are full of jealousy sometimes, I don’t see that although he is doing business he is buying so many things, he is on treatment, why is his buttocks or his body changing.

M: What do you think health care workers like nurses and doctors once we present with these cases, shapes that have changed, how do we think they will respond to us once we present with buffalo hump and a fat tummy and they know that this person was not like this before but now that he has started taking ARVs he has these? How do we think they will respond to that?

P009: They will help you because they know what causes that.

P006: That is why when I give some days to come and check after two weeks or even a month to see how you are responding to the tablets.

M: Uhm.

M2: So they give you days to come back?

Ps: Ja.

P006: We know that sometime you are going to develop something, rashes, high blood pressure and whatever it doesn’t change.

M: We do not have anything that has developed/grown as a result of taking ARVs, there is no change that we could say we could say, and I think I have developed 4, 5, and 6 as a result of ARVs?

P003: For me there is nothing, no.

M: We seem to be shaking heads.

P006: You see the conditions are not the same, with others they start within a month of taking ARVs, others are strong, they are not the same.

M: I get you.

M2: And how does an individual living with HIV feel about the responses to the body changes that people have, the community, family, how do you feel about the things that they say?

P008: At first ((initially)) I was very worried but now I say that I am getting well and I am getting some treatment, I don’t worry anymore because my family does not worry what about the next person, what is that person going to do? I have got my family supporting me, I don’t worry. We used to get worried before and not anymore.

P003: I will feel offended to be told about something I already know.

M2: Okay.

P003: I can see I am losing weight and you come and tell me, you are losing weight ((P laughs)).

P006: It’s like an answer. I know that I am losing weight.

M2: So please don’t tell me.

P006: Because we people, we are positive I can say that we are better because we know our status, those do not know anything that is why they say those things, that is why I am saying that if they keep on criticising us, saying many things to people who are positive

M: Uhm.

P006: Because I am taking some tablets, I can live longer than them because to die is for everyone, like today you are gonna die, any minute you are gonna die so if you are afraid to get tested that means you are afraid of dying.

M: Which is something that will happen eventually anyway.

P008: I think that he is supporting me because you can talk, you can do anything but if you do not know your status, I don’t care about you because if you say I am sick, what about you. Do you go to the doctor or do you know?

P006: Ja, because to know your status is 100% than just stay.

M2: Okay, thank you very much]

M: [Did we ask this question, in general do you think]

M2: [Oh okay, in general do you think body shape changes in people who are HIV positive? Does it generally change?

P003: I don’t think so.

P006: Sometimes it does but sometimes it doesn’t change.

M: If it does change, what causes those changes to happen?

P006: You see if you are HIV positive body changes, you will be ordered to take so many things, if you don’ have those tablets you are gonna eat what you want once a day but those tablets you are forced to eat too much. Some of the food that we eat is going to change your body maybe they are going to tell me to reduce the amount of salt and sugar in my food and other things by the time…, those times you are just eating salty things, sweet things, but the body does not need to too much salt and sugar. That’s the thing but HIV positive people they are ordered not to eat a lot of salt and sugar, not to smoke hard and drink hard, just to say stay normal as you will see your body changing because of those things.

M: So does the body change for the better or?

P006: Ja because those instructions you are ordered to do them for your body, so it means that the body will change.

P003: The body sometimes changes as a result of the side effects of ARVs which we are told about before we start the treatment that there is always a possibility that we might develop things that were not normally there before rashes or whatever or lumps as you were saying but it’s not a definite thing, it’s not a definite things, it’s just a possibility. So I can’t generally say the changes will always be there.

M2: Are the changes which happen different for women and men?

P008: Come again?

M2: The changes in body shape are they different for men and women?

P006: Ja.

M2: What is different about them?

P001: They are different because I saw my wife, she has got this thing, what did you call it, buffalo hump and each time it’s growing day by day you see and I don’t know, she used to come here and I don’t know what she is supposed to get so that she must get well because those things were not there.

M: They did not change her medication?

P001: They changed many times, they failed because it’s that hump here and a big stomach and it makes her unshapely ((lose shape)).

M: Okay, I am sorry about that so in order words you are saying that the changes are different because for some women they can develop buffalo hump and then the stomach might become a little bit bigger. Any other differences we can think of in terms of men and women and changes in body shapes?

P006: We, men, our bodies are stronger than women ((M2 laughs)).

M2: Are you sure? ((All Ps and Ms laugh)).

P006: We take time to get changes, with women; you are going to see the changes in the hips and whatsoever but we, men take time.

M: Okay.

P003: I also think that men are generally more active in comparison…, not that all women are not active but generally speaking men are more active in terms of sporting activities in that they burn fats and women, from what I have noticed, a lot of women…, a few will participate in activities like exercise, jogging, sports and things like that so I think the build of fats can be more in women and the fact that they do that fats in preparation for babies.

M: Okay, do we have anyone, who has anything to say related to this topic which has not been said?

P001: Which one?

M: Anything, but relating to this topic that we just had?

P001: I think this discussion was good, it has motivated me and it’s building me because we share the same and we believe in the same thing, we don’t talk bad to each other and we know outside there are some people who are talking, we hear them but we don’t worry. We are going forward.

M: Uhm, we get you. I think we have to take this opportunity to thank you for attending and for participating in this discussion. We very much appreciated your input and your time, ja, thank you very much and also for your patience waiting for us when we were a little bit delayed on the road due to traffic and stuff like that, thank you very much

End of discussion
