# Supplementary material for: “If we lose it, we are worried”: Individual and provider level perceptions towards weight change among people living with HIV who undergo TB screening in routine health care settings in Gauteng Province, South Africa
Source: PLoS One. 2025 Sep 22;20(9):e0331904. doi: 10.1371/journal.pone.0331904 (PMC12453174; doi:10.1371/journal.pone.0331904)
Supplement: S4 File — (ZIP) [file pone.0331904.s004.zip › S4 Transcripts_final/FGD 3.docx]

**Transcribing Conventions**

**...** Ellipses indicate talk omitted from the data segment

**(( ))** The transcriber’s comments.

**( )** Empty parentheses indicate some talk was not audible or interpretable at all (we include the line for instance 20:15)

**(.)** A dot enclosed in parenthesis indicate a short silence

**[ ]** Square brackets indicating beginning and the end of overlapping speech.

::::: Elongated talk

**BEGINNING OF DISCUSSION**

M: Okay, the date today is the xxxx (interview date). The time is xxx. (.) ((M speaks to M2 on the phone and there are participants who are talking amongst themselves)). I am very, very sorry about that. Let us begin. Eh, I am going to ask per person. Maybe if a person can say who she is and which part of xxxxx (area) she is from. Just quickly so that we can go ahead with the discussion.

P: I am xxxx.

M: Thank you. Maybe we can begin with number 1, so that it is easy. Let us proceed this way so that it is easy. I have found it xxxxx (name)!

P: Let us start here.

P: Who is number 1?

M: Let us start with number 1 so that it is easy and then go this way.

P001: I am xxxxx (name), from xxxx (area).

M: May we please use our numbers and not our names right.

P: Oh, our names.

M: Please. For confidentiality reasons.

P: Mh.

P001: Okay. I’m number 1. xxx (area)

M: xxxxx (area)?

P001: Yes.

M: Mh.

P001: xxxx (address).

M: Okay.

P002: Number 2, xxxx (area).

M: xxxxe (area), mh.

P003: Are you finished?

M: Mh.

P003: Number 3, xxxx (area name).

M: xxxx (area name), yes.

P004: Number 4.

M: Yes.

P004: xxxxx(area).

M: xxxx (area),

P004: Mh.

M: Mh, all right.

P: Number 5 has not arrived yet.

P: Five?

P: The lady who was sitting here.

P: No, it’s 6.

P: Oh, six. This lady is 5.

M: Mh. Yes lady?

P005: Number 5, xxxx (area name)

M: xxxx (area name)?

P005: Yes.

M: Mh. Lady, are you going to participate?

P: And then how am I going to do it?

M: Must I give you the papers?

P: Yes.

M: Okay, number 5 said xxxx (area name). And then number?

P: Number 6 is the lady who went out neh?

M: Number 6 went out, you are number 7 neh?

P007: Number 7, xxxx (area).

M: xxxx (area)?

P007: Yes.

P008: Number 8, xxxx (area)

M: xxxx?

P008: xxxx (area name)

M: xxxx (Are name), mh. I am xxxxx (name), from xxxx (area) here nearby, next to the lady nearby. Okay. Which number are you? Please speak up.

P009: Number 9, xxxx (area), number 10 I mean.

M: xxxx (area)?

P: area

P: You are number 9.

M: You are number 9.

P: I am number 10.

P009: Oh are you number 10?

P010: Eh.

P009: Oh, so it means I am number 9?

P010: Eh.

P009: I forgot.

M: [Okay.

P009: I am number 9].

M: Number 10? Do we now have number 11?

P010: I am from xxxx (area),

M: xxxx (area)?

P010: xxxx (area)

M: Okay, number 10. Lady*,* it means that you are going to be number 11.

P: 11.

M: Lady, where are you from? We can close the door now because I don’t think we are expecting anyone to come in. Except number 6. Huh, we no longer have something for number 11.

P: What?

M: The ((what is said now is inaudible but the transcriber thinks that M is referring to a paper)), but we will know that you are number 11. We welcome you my sister. Okay, as we have said, we are trying to know where people come from, from around here.

P: Okay.

M: Where do you come from my sister?

P011: I am from xxxx (area name)

M: xxxxx (area name) as well?

P011: Yes.

M: Oh okay, we already have someone from xxxx (area name). Okay, let us begin now with our discussion. Eh, when you attend the clinic for your appointments, as we have done today does the clinic nurse or the doctor ask you whether you have lost weight or not? May I please sit here at the back so that I can be able to hear you.

P: [They do not ask us.

P: They ask us].

M: Do they ask you?

P010: [They do not ask me, I ask them.

P: They do not ask].

P: You also see yourself when they weigh you.

M: Okay, let us wait a bit so that my sister can finish here. (.) So, they ask you but sometimes they do not ask?

P004: Right now I was at the doctor.

M: Mh.

P004: He told me that my CD4 count is high neh?

M: Mh.

P004: But I have lost weight today.

M: Mhh.

P004: It is different from what it was 6 months back when I saw him.

M: Eh.

P004: You see that?

M: Okay.

P: I heard that from him when he was telling me.

M: So he told you without asking you?

P: Yes, he told me and said I had lost weight.

M: Okay. So, in other words here in this room sometimes we are asked, [and other times we are not asked

P: Other times we are not asked].

M: But, every time they tell us whether we have gained or lost? ((Lost weight)).

P004: [Yes.

P: Mh] ((A majority of them also agree)).

M: Okay, okay. Why do we think the doctors, nurses and counselors ask us whether we have lost or gained weight? Let me start with number 8.

P008: I think that when they tell you that you have lost weight, it is when they show you that your body is not in the right form.

M: Mh.

P008: So, when they say you have gained weight they mean your body is going according to the form so you are progressing.

M: O:::h, they are checking your progress number 8?

P008: Mh.

M: Yes number 2, your hand was up.

P002: I think that when they tell you about your weight.

M: Mh.

P002: They are trying to see how their pills work.

M: They want to see how their pills work?

P002: Yes.

M: Number 2.

P: Food also goes along with it, does it not? The kind of food you eat. Proteins.

P: How you eat.

M: They want to check the type of food you eat?

P: Yes.

M: Okay. Number 7, here next to me. You wanted to say something?

P007: I also wanted to say the same thing that they want to see how we are progressing when they ask that it looks like you have dropped ((weight)), now you are not right.

M: Mh::::h, it’s progress again?

P007: Yes.

M: Yes number 5.

P005: Sometimes when they see that you have lost weight they send you to a dietician to assist you with your weight.

M: O::::h, they send us to dietitians as well?

P005: Yes.

M: Okay. Yes number 11.

P011: I think they want to check our progress and then if you lose weight then they can ask you: do you take your medication regularly?

M: Mh.

P011: Or how is your diet and then they send you to dietitians.

M: Okay, all right.

P011: Yes.

M: Does anyone want to say something which has not been said?

P004: I am here.

M: Yes.

P004: He asked me what my problem was and I told him that I was confronted with a problem regarding my child, right.

M: Mh.

P004: But it has been long. So now I had an ‘undressing ritual’ ((a cleansing ceremony which is performed by the family of the deceased)), that thing came back, you see?

M: Mh, mh.

P004: And then he said to me that must have been the cause.

M: When you say you had an ‘undressing ceremony’ are you referring to *inzilo* ((a mourning attire worn during the period after the death of the deceased))?

P004: Yes, I was taking off *inzilo* ((mourning attire)).

M: O::::h.

P004: So the doctor says, oh it must be due to that, that thing that my child passed away came back. Not it came back again as I was taking off my mourning attire on Sunday.

M: O::::h, sorry. So they are also checking how your living conditions are?

P004: How our condition is.

M: Yes number 10.

P010: So I asked him why I keep losing weight and he said perhaps it is because I recently took out my womb.

M: O::::h.

P010: I have recently done a womb operation because I had a growth.

M: Okay. It must have been the pains.

P010: Yes.

M: Okay. When they weigh a person living with HIV and ask her questions about her weight.

P: Mh.

M: How does it make them feel? For instance, let us start with you. I want you to think about this morning when you left home to come to the clinic.

P: Yes.

M: Here, some came by taxi, some drove alone. While you were on your way here how did you feel?

P009: I was happy.

M: What was going on in your mind? Number 9 says she was happy. Number 8?

P008: You know I usually ask myself what kind of doctor I will meet. Is he going to be able to listen to my problems? And according to my results from last month.

M: Mh.

P008: My results. I focus a lot on the state of my health, whether what the doctor will tell me will make me happy or sad. So I am usually not in a right mood.

M: M::::h.

P008: Until I reach that day and the doctor tells me. In other words my day starts afresh after I have met with the doctor.

M: Okay, so you are usually a bit tense thinking about the outcome of the results. Yes number 5.

P005: (. )

M: I am going to ask for one thing, if we get phone calls may we please leave and respond at the door so that we do not disturb the flow of the conversation and if we can, let us please put them on silent so that when they ring we do not interfere with the record. Yes number 5?

P005: (In audible).

M: Please speak louder dear?

P005: When I left home I was depressed because I had defaulted on the pills.

M: You were depressed?

P005: I was thinking that when the doctor comes he will not treat me nicely maybe he will scold me.

M: Mh, okay. You defaulted?

P005: Eh.

M: Okay all right. What do others say? What was going on in our minds when we were on our way to this clinic?

P004: All right. When I woke up this morning.

M: Eh.

P004: I can say as from yesterday. This thing started yesterday. When I realized that yesterday was the 19^th^, I said: oh Jehovah I did not go to the doctor! How did it happen? When I went to bed I was stressed. When I got here I spoke to one lady and I told her that I was told to come yesterday but I did not come so what are they going to say? She said no *mama*, we no longer come on Mondays and Tuesdays. We come on Wednesdays, only then was I relieved.

M: O::::h.

P: Even when they told me that my CD4 count and my viral load were right I became relieved, you understand?

M: Okay. You were stressed because you had missed your appointment. Yes number 3?

P003: Today I had questions, I was a bit stressed that when I got my results maybe my CD4 count had dropped and I would have to start taking the pills.

M: Mh.

P003: I was not right, I was a bit tense.

M: You were thinking about your CD4 count and what the results were going to be. Yes number 2?

P002: So, when I came here I was …, I become happy when I come to the hospital.

M: Mh.

P002: I tell myself that I am going to get help.

M: You were happy because you are here to get help. Right?

P002: Yes.

M: Mh.

P002: So, if I sit at home how am I going to know what is going on with my life ((health))?

M: Mh.

P002: Yes.

M: You are saying, if you stay at home what is going to happen about your life ((health))?

P002: Yes.

M: Oh okay. All right. Yes number 1.

P001: When I come here I am alright. The only problem is that I was a bit stressed because I have to change pills.

M: O::::h! When you left home …, when you left home you were aware that you were going to change pills?

P001: No.

M: We are currently talking about how were you feeling when you left home?

P001: I was all right because when I boarded the taxi I even greeted the people. They conversed, I also conversed with them and I was fine and sharp.

M: Oh, okay.

P001: Because this thing is very stressful, you understand?((Inaudible)).

M: Mh.

P001: So ((inaudible – 14:54)).

M. Okay. Yes number 10.

P010: When I come I am happy because I am going to tell the doctor everything and I am not going to hide anything because he is not going to go around telling other people.

M: Mh, you are happy because you know that your things will be treated with confidentiality.

P010: Mh.

M: You have already spoken number 9. Yes number 7?

P007: Number 7. When I come here I am happy because I am coming to the doctor and I am here to become better and when I come here with this thing I will be completely alright.

M: Mh.

P007: And another thing I was upset because at xxxx (area) …, my parental home is in xxxx (area) so my husband passed away and it has not been long since I returned to my parental home and that is why I have written down that address.

M: Mh.

P007: They shot some people there and there are no taxis, so I had that worry of whether I was going to reach the hospital.

M: O::::h.

P007: Today the guy who brought us this morning said he was going home to sleep so now we don’t know ((inaudible – 15:51)).

M: Mh.

P007: Otherwise I am usually happy when I come to the hospital. Especially if I am not all right.

M: You were stressed a bit because you were not sure if you were going to get transport to come to xxxx (hospital name)? Yes number 11.

P011: Oh I am always happy when I am in the taxi coming to the hospital.

M: Right.

P011: Like even with my repeats, I make sure that I do not default.

M: Mh.

P011: I make sure. And then what made me very happy even last time when I came is that I had filled in my policy things. The policy I took at the bank.

M: Mh.

P011: So the doctor had to fill them in for me. So, I had to check what my viral load was because they were out and he told me to come back after 6 months in xxx (month).

M: Oh okay. So you were happy?

P011: Yes.

M: Emh. We are now at the waiting room, we are now waiting. At the waiting area waiting to be seen.

P: Yes.

M: How were we feeling at that particular time?

P: Frustrated.

M: Frustrated? Why?

P: Ah, the staff is slow, number 1.

M: Right?

P: Since we have been here in the morning.

M: Mh.

P: We were sitting and it’s not like we don’t have things to do.

M: Mh.

P: But we have to come here. We are sitting and the staff is slow.

M: Okay. You were frustrated because the pace they were working in was slow. Yes, number 7.

P007: Eh, they sometimes call people who came after us before us to go and weigh.

M: O::::h!

P007: And it makes you to wonder why they weight people who came after us before they call us whilst we came first. Sometimes when the doctor comes they are called [again

P: Again] whilst you arrived at 6 and you are still here!

P007: The ones who arrived whilst you are here and you saw them.

M: So in other words, the queue … some people jump the queue?

P: It ((the queue)) does not go well ((accordingly)).

P004: No, no! Let me say it like this, it is like what happens at the main pharmacy. Like I am going to go to the main pharmacy to get my medication for the skin.

M: Mh.

P004: I am not going to get it at the same time. When they take our cards they mix them up when they take them there to be sorted for medication, do you get my point? The manner in which they sort the cards.

M: O::::h.

P004: They sort the medication, that’s why there is this confusion and you find that a person who came in last, a person who came in first …, a person who came in last is assisted first.

M: M::::h.

P004: Our cards get mixed up.

M: They mix your cards, okay, for medication. Yes number 7.

P007: No I must be honest, since I started coming to xxxx (hospital name)I do not have any complaints about the way they operate because since I started coming here there has never been a time when I left at 3 or 4.

P004: Yes.

M: Mh.

P007: So, I am still happy, once it reaches that point, I will be having a complaint and my complaint will not go to anyone but it will go straight to the headmaster or Head Office.

M: You don’t play games. ((She laughs)). Mh.

P007: Yes, ‘cause it will not be nice to leave at 4 because I also have things to do at home but since things are the way they are, I am okay and I have no complaint so far.

M: Mh.

P007: I know I have a right to go to any department because this is my country.

M: I get you, I get you my dear. Yes number 11. My sister did you sign everything?

P: Yes, I have signed.

M: Okay, we welcome you back.

P: Okay, thank you.

M: Mh. Yes my sister.

P011: I think they operate well at the waiting room, I think they operate well but when you go to the main pharmacy, like when you go to the doctor, I think at least they could change and maybe put you on the system ((inaudible – 19:39)).

M: Mh.

P011: So, you understand? If you are going to the doctor they must take out your brown files, they must follow each other chronologically and then when they are there, there are different units and there are different doctors.

M: Mh.

P011: You find that maybe there is a doctor who starts with ward rounds, they do not arrive at the same time. So each doctor has his own patients.

P004: Each arrives with their own files.

M: O::::h.

P011: That’s why you find that the files do not follow each other chronologically.

M: Oka::::y.

P011: Yes, that’s the reason.

M: Number 6, we are talking about how were we feeling at the time when we were at the waiting area waiting to be seen, waiting to get our attention right. Is there anyone else who has something to say about the waiting area, and about what was going on in your minds when you were at the waiting area before we proceed?

P: ((inaudible – 20:31)) when you have to have your weight taken, you are afraid to even go to relieve yourself ((urinate at the toilet)). Before you have your weight taken you are afraid to even move.

M: O::::h.

P: Yes. Because they might call you whilst you are still at the toilet.

M: Mh.

P: So now they delay to do things the way you think they ought to do them.

M: M::::h. We are now at the weighing scale. We are now being weighed.

P: Okay.

M: What is happening? What goes on in our minds when we are being weighed? What goes on in your minds? What are we thinking when we are being weighed? Let us start with number 2.

P002: Okay. When I am being weighed I do not have a problem, I want to know what is going on with my weight.

M: Mh.

P002: Yes.

M: How is your weight, mh.

P002: Yes.

M: You don‘t have a problem?

P002: Mh.

M: Okay. Yes number 10.

P010: I become stressed when I have my weight taken because I am gaining at a very slow pace and this does not make me happy.

M: Mh.

P010: I become stressed. I often wonder what is going to happen today. So when I ask the doctor he answers and says it’s because I have many ((inaudible – 21:34)) in your body ((inaudible – 21:41)) increases gradually, gradually.

M: M::::h, gradually?

P010: Yes.

M: Okay, number 3.

P003: I become stressed because I wonder if my weight is going to go down or the doctor will tell me that my CD4 count has also gone down because that means I am going to start taking the pills.

M: Mh.

P003: I was stressed that my weight was going to be terrible.

M: Mh. What made you to]

P004: [She has not started taking the pills.

M: You have not started taking ARVs?

P003: No, I have not been given ARVs yet, but I check my CD4 count, viral load … everything, even the weigh.

M: Yes number 6.

P006: Oh, my sister having my weight taken makes me happy because I used to have a weight problem.

M: Right.

P006: My weight used to be very low so when it goes up it makes me very happy because it means I am gaining weight.

M: Okay. So bit by bit it is going up?

P006: Yes.

M: Okay, all right. Yes number 8.

P008: My weight used to go up and down. So it used to worry me until I asked the doctor why it is going up and down because I was concerned that maybe there is something I am not doing right.

M: Mh.

P008: The doctor told me …, he relieved me and said that the weight of a normal person goes up and down after that I was free.

M: Oka::::y.

P008: I became okay.

M: It fluctuates?

P008: Yes.

M: Yes number 11.

P011: I used to gain weight, a lot of weight. I used to reach 96 / 97.

M: Sorry?

P011: I would reach 96 / 97, my weight went up a lot neh.

M: Okay, mh.

P011: So by then it was that time when I used to take medication twice, in the mornings and evenings.

M: O::::h.

P011: So when I told the doctor, the doctor said that I was going to gain even the breasts so he advised me to reduce my weight and even changed my medication, currently I only take my medication in the evening.

M: All right. O::::h.

P011: So he even started telling me about my diet.

M: Okay.

P011: So ever since I started controlling my diet, my weight is normal because he told me not to be overweight.

M: Okay.

P011: Because it’s not healthy.

M: Eh. How does it make us feel? What goes on in our minds when we stand on top of the scale?

P: This morning I was excited.

M: Right.

P: When I thought about the scale I was so excited because I have been weighing 152, so I had to hit the gym whatever it took.

M: Mh!

P: So today when I thought about coming to the scale I was excited. I wanted to see if this 152 had gone down. ((They all laugh)). When I got there I found out that it had gone down it’s 62.5.

M: From 152 to 62.5! Haa!

P004: That’s good hey? That’s good, that’s good!

M: You did it!

P004: I will not comment.

P007: I am complaining about my legs!

M: You are happy when you are on the scale. Yes number 5.

P005: I was happy today ‘cause I had lost a lot of weight, I was admitted at xxx (hospital name) ((She is inaudible because there are others talking and they are drowning her voice – 24:40)).

M: Ha::::a! Okay, so you were happy today because]

P005: [Mh.

M: Okay. May we please allow one person at a time to talk?

P: Number 7 says she is worried about her legs.

P007: *Hayi*, my legs!

M: Okay. Let us have one person at time talking. Let’s have one person at a time talking so that it will be easy.

P: Okay.

M: Neh? You wanted to say something number 7.

P007: I am worried that my legs have become this size.

M: What was the size of your legs before?

P007: My legs were okay but now they have become too small.

M: O::::h okay, sorry. Otherwise when you stand at the scale how do you feel?

P007: No when I stand at the scale I am okay, I am happy, the reading on the scale is alright but in my heart I’m saying: There … ((She is referring to her legs and they all laugh))

M: On your legs, you are thinking about your legs?

P007: Yes Lord. I am happy now that it is Winter season because I wear pants. When summer begins I’m thinking maybe they are saying: There goes Broom stick.

M: Mh, okay. You have now been weighed. You are now going home as you usually do, what goes on in your mind? For instance let us begin by asking how you feel when you realize that you have lost weight?

P001: You won’t feel good.

M: Yes number 1. Why?

P001: You will be worried. When you get home you will worried.

P004: You say why have I lost weight.

M: You become worried?

P001: Yes.

M: Okay. What do others say?

P: Same thing ((Others agree with her)).

M: Most of the time you become worried that [eh

P: Why have I lost weight.

P: What is happening]?

M: I think number 9 you have revealed a different perspective about losing weight.

P009: Mina it was stressing me because I was overweight and it was affecting my health a lot. I couldn’t move properly because I was overweight.

M: Mh.

P009: Even my heart was beating abnormally ((fast or pounding hard)).

M: Mh, and when you got home how would you be feeling? How did you feel?

P009: I’m more excited about my results.

M: Excited, and motivated as well? ((Others laugh)) About the weight loss.

P009: A lot!

M: Okay. Do we all agree that most of the time when we lose weight we don’t feel good about it? ((They all agree)). And then if you were overweight you become happy when you realize that there is progress? ((They all agree)).

P004: But today I was not surprised because I know where I come from, you see?

M: Okay.

P004: Yes, I was not surprised but I must also assist my weight so that it does not drop too much, I must console myself because I know where I come from. I saw this whole thing and I saw how I tried to help this child to get better you understand?

M: Ah::::h.

P004: So, the only thing is to remain hopeful that things will be fine, when I come back in xxxx (month) to see the doctor …, because I see him every 6 months, when I come to see the doctor in xxxx (month), I’ll be fine.

M: Mh.

P004: Do you understand that?

M: Yes.

P004: It is only today that my weight dropped, but even then it has not dropped too much.

M: Mh.

P004: So, I’m not so hurt. I’m okay, I must just be calm and take my treatment and eat a healthy diet and then I’m fine.

M: Oka::::y, alright. We indicated that when we lose weight we become concerned and worried.

P: Yes. ((Others also agree)).

M: Most of the time, how do we feel when we gain weight? Yes number 8, I have been ignoring you.

P008: ((They laugh)). No, when I gain weight I become happy but what is hurtful is the situation similar to number 9’s. You see gaining too much weight is not healthy for a person and I don’t like it, I don’t want to be fat. But I am grateful and I’m okay.

M: Mh. ((They laugh)). Okay, number 8, when you come here to xxxx (area) and realize that you have gained weight, how does that make you feel?

P008: I feel good but only if I have gained a little bit.

M: Okay.

P008: Not to gain too much weight ((an exaggerated weight gain)), no, no, no.

M: O::::h. So you become happy when it is a moderate weight gain?

P008: Yes, yes.

M: Okay, okay. What do others say? Yes number 3.

P003: I like to gain weight.

M: Mh.

P003: I like gain weight a lot especially seeing that I have not started taking treatment it means that I am still looking after myself.

M: Okay.

P003: Yes.

M: Okay. By the way you said you have not started taking treatment?

P003: Mh.

M: Oh, I remember.

P: Gaining weight is the right thing neh, it makes one happy but one must not gain too much weight.

P: It must be alright.

P: Yes, you must be right.

P: Eh you must just be alright because it will stress you.

M: Gaining weight is stressful. You talk as if gaining weight will lead a person to be shapeless.

P: [Ye::::s.

P: You become shapeless].

M: Okay. Yes number 10.

P010: I would be very happy if I gained weight because now I wear size 13 to 14 and I turn xxx (age) years in xxx (month) so I would be very happy.

P004: But you are still sick, aren’t you? The doctor has told you. So you must not put your mind too much on it. Especially because you have just had an operation. You must just take it step by step, step by step.

M: Mh.

P004: I was telling her that when some of us started being ill, I was in a wheelchair. I had a clot here and even now I still have a black mark. My first doctor was Doctor xxxxx (name), he used to give me warfarin. He gave me warfarin until he stopped.

M: Do you have a support group here in xxx (hospital name)?

P: We?

P: No.

M: You don’t have a support group. O::::h okay. What does number 10 say? You are saying that you like to gain weight. We are talking about how gaining weight would make us feel. Yes number 6.

P006: My sister I would be happy if I gained weight since I was born I was like this but the time when I was very sick I was thinner than I am now. Right now I have gained a bit compared to the body I had before.

M: Okay.

P006: I am all right now but I would love to gain more weight.

M: It seems as if most of us would love to gain weight ((they agree)) but you don’t want to gain too much.

P: Yes. ((Others also agree)).

M: Others are worried that sometimes when they gain weight their bodies will change and become different. Let’s end with your comment number 2.

P002: It is also the same thing with me. I would be happy if my weight can be back to where it was because I used to have a big body.

M: Mh-mh.

P002: From since when I was growing up.

M: Yes.

P002: So when I got sick I lost weight.

M: Okay. You are saying since you were growing up, you had a big body and then when you got sick you lost weight. So gaining weight is a good thing for you?

P002: Mh.

M: Okay, all right. Emh, if a person living with the HIV virus is asked about her weight by the clinic staff. What do we think can make that person to say that she has lost weight? (.) Yes number 6.

P006: I think a person can lose weight if she does not give herself peace of mind and keeping on saying that I have this disease and I will die and leave my children. You cannot let that thing to stay in your mind because you must also be free.

M: Okay. What I’m trying to find out from you guys here is: What can make me, as a person who is HIV positive to say I have lost weight when a nurse, doctor or counselor asks whether I have lost any weight. What will show me that I have lost weight such that I will respond and say I did lose weight? Yes number 5.

P005: I notice the way in which my body has been reduced.

M: Okay. How can you tell that your body has been reduced?

P: Clothes.

M: Clothes. Okay.

P: No, sometimes you can feel even when you walk you can feel it. ((Some are talking at the same time making it difficult for the transcriber to hear properly what each one of them is saying)).

P: You become lighter.

P: You can tell that this time it is not the flesh that is heavy, it is the bones that are heavy. You can tell when it is the bones that are heavy and not the flesh. Even when you dance you can tell that these are bones ((they laugh)).

P: Eh, you can tell when you have lost weight.

M: Guys, you are saying something I have never heard of before! How is it possible for the bones to be too heavy for a person?

P: [They are heavy!

P: The bones are heavy]! ((Others agree with her)).

M: How? How are bones heavy?

P: [You walk as if you are tired.

P: You will walk as if you are tired].

P: You become light ((like a feather)).

P: Because now you are the one carrying the bones instead of the bones carrying you.

M: O::::h, that is when you have lost a lot of weight?

P: Yes.

M: Oka, alright. I like the way you are illustrating it ((they all laugh)). Yes number 11.

P011: You can tell from the reading on the scale. You can see it on the scale when they weight you that you have lost weight.

M: Okay. In instances where you have not climbed onto the scale yet, they are still asking you before your weight is taken?

P: [It is these bones that are heavy.

P: Your clothes become too heavy].

P010: You can also see yourself.

M: Mh. Besides the clothes and your body being heavy, what else can make us notice that we have lost weight?

P: You become weak ((others also mention this)).

M: When we say you become weak, what do we mean?

P010: [You become tired

P: You become tired], and lazy and sometimes you feel dizzy.

P: Even when you have to go to a nearby place, you feel as if it is very far.

P: You even lose appetite.

P: Losing appetite as well.

M: Losing appetite. Does it make you think that you have lost weight?

P: Yes.

M: Okay.

P: Having too much on your mind.

P: Having too much on your mind can kill you.

P: Stress.

M: Stress.

P: Sometimes that’s why doctors sometimes say if your weight has dropped or gone high, don’t put it in your mind because it can cause more weight loss.

M: O::::h okay. Is that how they advise you? ((Someone is coughing)).

P: Mh.

M: Okay. All right, some of you said that worrying too much, being stressed. How does being stressed make a person to lose weight?

P: [Because you always become full and as a result you don’t eat.

P: You don’t eat sometimes.

P: Yes, you don’t eat.

P: You lose appetite].

M: You don’t eat?

P: [You don’t eat because this thing is here.

P002: You don’t become hungry, you don’t feel like eating.

P: Even when you are hungry, you don’t feel it.

P002: You just sleep].

M: Okay. Let us have one discussion please, so that the recorder can capture all of us, it’s a request. Yes number 11. (.) You were saying something, you were talking about stress and inability to eat.

P011: Yes. You don’t eat, when you are stressed you lose appetite neh.

M: Mh.

P011: You feel like always sleeping.

M: Oh okay.

P011: Nothing makes you happy, like you feel like sleeping all the time. It’s the way stress affects you.

M: Okay. Yes number 4.

P004: When I am stressed I am not able to sleep, I’m not able to eat and I work day and night. I worry.

M: M::::h.

P004: I don’t finish ((working)), you could be talking to me whilst I’m absent minded.

M: M::::h.

P004: You will even touch me and say: Hey I am talking to you!

M: Mh.

P004: I don’t know about those who say they sleep when they are stressed, I lose sleep.

M: Mh, we react in different ways.

P: Different ways.

M: Yes, yes.

P: I am also like that. I do not sleep when I am stressed.

P: For me, there is no sleep because I am always thinking.

M: Mh. What does it mean for us to lose weight whilst we are attending the clinic at xxx (clinic name)? What does it mean to us in our minds?

P: You become afraid because you think about TB.

P: Yes.

P: It becomes worse if you are coughing as well. You say there comes TB.

P: You think that I am infected ((by TB)).

P: It’s it ((TB)), you say but I am not running a high temperature but then you say man I am losing weight.

M: O::::h.

P: You do notice it ((weight loss)).

M: Mh, you become worried that you got infected with TB.

P: [Yes.

P: TB, yes.

P: Eh].

M: Okay. What do others say? Number 8, I think you wanted to say something. What does losing weight mean to you when you are attending the ARV clinic.

P008: Hey, it means there is something I am not doing right, there is something I am missing.

M: Maybe something is wrong?

P008: Yes.

M: Mh. What do others say?

P004: What can I say? Hey, let me not answer too much.

M: Oh sorry, thank you. Yes number 9.

P009: Eh just to add to number 8’s comment [that it might be something you are not doing right.

M: You know what I wanted to do neh? I wanted to shift my chair so that I could see everybody], I’m sorry. Continue my dear.

P009: It might be something you are not doing right. It might be maybe you are not taking your treatment well at the same time it might be you are stressing a lot.

M: Mh.

P009: So, those things go hand-in-hand, because when you stress it affects your immune system and everything changes and you start losing weight.

M: Mh okay, all right. What do others say? Yes number 10.

P010: I am saying, another thing that makes maybe a person to lose weight, you must check because I am HIV positive, I must check whether I have TB or not because that also causes a person to lose weight.

M: Mh.

P010: I must not only concentrate on checking that I’m positive only, no. TB also causes it ((weight loss)) so you must go and check.

M: Okay, it means you could be having an underlying disease. Yes, let us conclude with you, number 4.

P004: You know for me, this disease started with TB. When I started losing weight, I had to check, even in my file there are a lot of TB reports. I went to check until the doctor asked did you take your treatment? Is it finished? Why do you keep on coming? I said I keep on coming because I am losing weight, he said: There is something. And we went into a room and Doctor xxxxx (name) said to me: Tell me what’s wrong. I then told him about my son, my last born who was taking *nyaope* ((a type of prohibited and dependence producing drug)).

M: My goodness! ((Exclamation)).

P004: We found out that it had raptured his lungs and when he died he was at Sizwe. He was transferred from here at xxx (hospital name) to xxxx Hospital.

M: Mh.

P004: Can you understand that all of this was not weighing heavily on me. I would talk to him and say I am taking my pills can you see, so you must also take your treatment. You see that?

M: Yes.

P004: I now take it and make it one umbrella, it was difficult for me.

M: Mh.

P004: I was also suffering from my own disease, meanwhile he was also bringing something else, you understand?

M: Mh.

P004: But after the funeral, I started to pick up weight, I started to feel like a human being and had the ability to laugh, even when I put clothes on I would look good.

M: Mh.

P004: Because you can tell even on the face when a person is stressed.

P: [Yes.

P: Yes]. ((Others agree as well)).

M: Mh.

P004: You are able to tell even when they walk or do anything.

M: Mh.

P004: So I’m fine, I’m fine. Even when the doctor told me today that you have lost weight but your CD4 is up and your viral load is fine. I was not stressed, I know where I am from and what I have seen.

M: Mh.

P004: I thought of that thing, you understand?

M: Mh.

P004: So I’m fine, I’m fine. Even going to the doctor today and he told me that I have lost weight but my CD4 is up and your viral load is fine. I was not stressed because I know where I come from and what happened to me.

M: Mh.

P004: I remembered that thing, you understand?

M: Okay. Is there any stigma attached to people living with HIV when they lose weight? When we lose weight, is there a stigma that is attached to us? Yes number 8.

P008: There is. The names they give to us are more than the size of the bible.

M: Oh no!

P008: Really. Omo, Z3.

P: Z3.

P008: They are too many.

M: Okay. What does number 10 say? Stigma attached to losing weight.

P010: Chachaz.

F: Sorry.

P010: Chachaz.

F: Chachaz? It is the first time that I am hearing that one chachaz. What does chachaz mean?

P: [It means you are like that.

P: These names are many].

P: OMO.

M: I have heard about OMO, I have heard about it because it has 3 alphabets.

P: Z3.

M: I have also heard about Z3. What does chachaz mean?

P: 2 room and 1 garage, they say that.

P: 2 room 1 garage.

M: 2 room, one garage. I am also hearing that for the first time.

P: I am also hearing that one for the first time. ((They laugh)).

M: What does 2 rooms and one garage mean? Is it because it’s 2 plus one?

P: Yes.

P: [They say you go to the same school.

P: At xxxxx (area name) the more common name is KFC]. ((A lot of them are speaking at the same time)).

M: Please, please, please!

P: KFC.

M: I was trying to understand the one of chachaz.

P: Chachaz …, when they talk about you they say you are suffering from chachaz, meaning you get cold, you lose weight because you are suffering from chachaz. They now call it chachaz.

M: What is chachaz?

P: I am hearing about it for the first time.

P: It’s HIV.

P: It means there sun is out but you still get cold, you see.

M: How does that link with it ((HIV))?

P: The link is that when you are sick I sit by the door and I feel cold and then you put me back in the house and then I feel hot so it is some of the things that happen when one is sick. So they end up putting you by the door, so they know that there is something that you are suffering from.

M: To be honest with you, I still don’t understand what ‘chachaz’ means.

P004: I also don’t know it, I am hearing about it for the first time.

P: You won’t understand it because that is the way they call it.

P: I think it depends on how they call it.

M: Is it the lingo they use?

P: Yes.

M: Okay. Did anyone else want to say something? ((Someone is coughing)). Sorry.

P: Some say KFC.

M: KFC, yes.

P: In the same way that the lady was talking about her things.

M: Yes.

P: Thin legs. Do you know what a KFC drumstick looks like? When they see you they say: It’s KFC! She went to buy KFC.

P: Or she goes to the same school as ((the transcriber thinks that P is using her hands to point at one of the other participants)).

M: Where? When they say thin legs they are talking about KFC? Going to the same school as?

P: You must remember that it changes you.

P: They say she goes to the same school as (.). Those who know that she is sick and they attend the same school. When we attend clinic together they will say we go to the same school.

P: They know her. ((Some of them are laughing)).

M: Is it now people who are taking ARVs gossiping about each other?

P: No.

P: [No! It is the people who do not even know their statuses who gossip

P: Those who do not know their statuses.

P: They don’t know them ((statuses))]!

P: I will not say I don’t know my status but I laugh at another person, I can see her but I don’t know my own status.

P: She does not want to get up ((get up and go to the clinic and check his / her status)).

M: I hear when you say: We go to the same school.

P: Usually it’s someone who will know that a person is sick and they can also see that I am sick.

M: Yes.

P: When they say that: KFC, then they say: No she goes to the same school as number 3.

M: Oh, now I get you. ((They laugh)). There was a hand somewhere here. Yes number 5.

P005: Some call it Go-slow.

M: Go-slow? ((They laugh)) What does Go-slow mean?

P: It means you are now sick.

P: You walk slowly ((They say it together)).

P: I gave it my own name, I call it Chomie ((friend)).

M: Chomie? A friend? ((They laugh)). Mh. Why do you call it a friend?

P: It stays with me, it sleeps in my body.

P: Yes, yes.

P: Everything happens to me, I am the one who knows about it.

P004: Yes, it’s true.

P: And love it.

M: All right. Yes number 2.

P002: They call it Z3.

M: Z3, okay.

P002: Mh.

M: All right. Yes number 10?

P010: Like I am from the CT scan so, so when they see me in the location they say: Hey this one has frequent visits to xxxx (hospital name) because she is sick, she is sick. So I am not bothered because I go there for the sake of my health.

P: Yes.

P010: Because I do not even hide my file and one of them suffered from it and was even buried because she did not want to take treatment.

P: Ahh! Because she was afraid she was going to meet you here.

M: When you say buried, did she die? Was she buried?

P010: Yes she died, yes she was buried because she did not want to take treatment. While I was walking around with my CT scan and going to xxxx (hospital name) for the sake of my health.

M: M::::h! Is there any other thing we want to say about these words like Chachaz?

P: No!

P: No that is all.

P: My friends and I, I have friends who are also sick like me and we live in the same place and we understand each other so we do not gossip about each other.

M: Yes.

P: So, when we talk over the phone maybe if there is someone who does not know about our condition, you see? Or maybe when we walk on the street and there is a neighbor around, so when we don’t want her to hear what we are talking about we will say: Tomorrow I am going to xxx (hospital), you also know things of this world. So we have our own language in order to make sure that the other people do not hear what we are talking about.

M: Aha.

P: So that they don’t know about our things, you see?

M: Okay, all right.

P: That we eat things of this world, you also know things of this world.

M: Mh.

P: So, it becomes easy for us to [understand each other.

M: Understand each other].

P: You see? Because we are trying to avoid lots of talks from outsiders.

M: I get you. Yes *sisi*, let’s conclude with your comment.

P004: You know I told myself one thing. I talk to it ((the virus)).

M: Mh. You talk to the virus?

P004: Yes. You reside in my blood, you don’t pay any rent so you will respect me. ((Others laugh)).

P: Yes.

M: Okay.

P004: If I say I want to go to a certain place, [I go.

P: I go with you].

P004: You know they know about me at xxxx (church name) in xxx (town name) at our headquarters. I stood in front of a big congregation in our church, in a church as big as the xxx ((church name which is the biggest church in Southern Africa)). I spoke and even took them out and said: Do you know these pills? They said they don’t know them, I said: They are ARVs, they were so shocked.

M: Mh.

P004: One of them asked: Why did you speak? I said: I’m healing myself. So I talk to it in the morning, during the day and in the evening and say: You live in my blood, you don’t pay rent, you just live for free, so you are going to listen to me and go with me where I want to go today. I am going to a night vigil, I am going to church or anywhere! [You are going to wait for me!

P: Behave]!

P: Yes. ((Others agree with her)).

P004: I was admitted at that time when you controlled me, I was admitted for 3 at xxxx (hospital name), now is my time! You are not going to say: Jump! And I say: How? And you say: High. No, you wait for me. That’s all.

P: Mh! ((Others laugh)).

M: I am hearing what you are saying for the first time.

P004: You wait for me.

M: Okay, alright. Let’s continue now. If a person attends HIV care, or comes here to xxxx (area) to collect ARVs, what do we think that person’s weight ought to be? (.) The weight of a person who has already started attending clinic here at xxxx (clinic name)? Yes number 6?

P006: You cannot control a person’s weight because we have different weights.

M: Yes.

P006: We might see one person becoming fat and think that it is due to ARV, only to find that it is her body ((her original body is like that)).

M: Mh.

P006: It did not affect the body.

M: Mh.

P006: She is taking ARVs. As I am this current weight, there are people who started taking treatment with me who are now fat, but they do not have that effect on me, my weight is constant in one place.

M: Mh.

P006: I sometimes wish that I could be fat, because others are also gaining weight due to the treatment.

M: Mh.

P006: But I do not become fat.

M: So your weight will become what it is originally?

P006: Yes. ((Others also agree))

P: Yes.

M: What do others say? (.) How do we expect the weight of a person who is taking ARVs to be?

P: It must go back to its original form.

M: Must you regain it?

P: [Yes, you must regain it and it must go back to its original form.

P: You must just be normal].

P: You must just be right.

M: Yes number 7.

P007: I prefer that it should go back to its form and be right because I can see that these pills are a great help.

M: Mh.

P007: They are really a great help, like, we must be grateful to God and the government, nurses, doctors …, like people who support us like you.

M: Mh-mh.

P007: When you think way back when there were no pills how people were dying.

P004: Oh, Jesus! ((Others agree as with her)).

P007: Our parents are no longer with us today and they died whilst they were still you, without even reaching the age of 50, they were crying for these pills. They were told about traditional healers, [and told about …

P: About *ukuthwasa* ((being initiated to become a traditional healer)).

P: That they have been bewitched].

P007: They would pay as much as 5000, imagine the money that has been wasted. We are now getting them for free.

M: Mh.

P007: And you would never tell that I am HIV positive, you will never make that mistake! I can say show me an HIV positive person, hold her by the hand and show me so that I can see her.

P: Yes. ((They laugh)).

P004: [You are speaking the truth.

P: Really, really].

M: Yes number 8. Yes number 10, sorry.

P010: Okay, I say weight must not stress you too much like *sisi* here says hers is high and mine is low, because we also have other different diseases in our bodies. ((Others agree)). Like I had TB times 2, and it damaged me, one of my lungs is gone. So, I will not say because her weight is high like the lady was saying. I must accept and not stress myself.

P004: Yes, yes!

M: Eh, okay. I want us to now talk about the ideal body weight. The weight that we usually say is the right weight, the best weight. I am going to give you these pictures. (.) Hooo! They are mixed with male pictures. I want to give you pictures of females only. M::::h, I think these are pictures of females.

P004: This is a woman.

M: This is also a woman neh?

P004: Eh. (.)

M: I want us to just look at them and if we can, let us untangle it and open it up. They should be 9. (.) Thank you. And then we can perhaps make them face this way, so that I can also see what you have in this plastic.

P004: It looks like they are still mixed with the ones for males.

M: Oh okay.

P: This one is also for the male. ((They are talking whilst they try to arrange the pictures))

P004: I’d rather be like this.

P: Yes.

M: This is a woman.

P004: No, no, no! This is worse. [I’d rather be like this.

M: This is a woman]. This is a woman. ((They talk at the same time whilst trying to arrange the pictures)).

P004: No, no, no, this is worse! I’d rather be like this. ((They continue to sort the pictures)).

M: I wonder who are you saying is sick?

P: She is too tiny. ((They are still sorting the photos)).

M: Which one out of these pictures we are holding …, I am going to ask that each of the photos we are holding must be shown to the whole group so that we can see which weight or body shape we can say is the best.

P: It’s this one.

P: Number 3, number 2 and [number 4.

P: Number 4 is okay].

M: Wait a bit. (.)

P: Number 11.

M: Which one do we say is right?

P: Number 3.

P: I want to see number 11.

P: Which number do you have?

M: You have number 5.

P: Number 4 is also nice.

P: Which number is that?

M: She is saying number 5 is nice. ((They are still picking pictures of nice bodies)). Which one is nice? Did you say this one is nice my sister?

P: Yes.

M: Which number is it? Please check at the back what number it is. Please check the picture at the back.

P: At the back.

P: How should I look at it?

M: Yes.

P: Number 4.

P: Number 4, yes, it is number 4.

M: Oh, it is number 4. We have picked number 5 and number 4.

P: Number 2 is also nice.

M: Number 4 as well?

P: Number 2 as well.

P: Yes, number 2 is nice.

M: Which number?

P: Number 6.

M: Are you saying number 6 ?

P: [Mh-mh.

P: Mh-mh].

M: Someone is saying number 6 is fat! ((They laugh)). Okay, we chose 5, 4, and 6.

P: Yes.

M: Does anyone have another picture she wants to pick or are we agreeing on these 3 that we have picked?

P: We agree on these, these ones are nice.

M: Okay, let us start with number 5. Let us put all the others down and concentrate on number 5. Who is holding number 5?

P: Me.

M: Okay, let us put that other down my dear. Here is number 5. Why do we say number 5 has the ideal body weight and shape? Let us start with the weight. Let us begin with the weight. Why do we think number 5 is right?

P: [She is fit.

P: She is full everywhere].

M: Mh.

P: She does not have a hunch back and she has lovely legs.

M: Mh.

P: She does not have a big breast.

P: Her arms are also [average.

P: The arms are also alright].

M: Mh.

P: [She is just okay.

P: Her neck also] does not hang. ((They laugh)).

M: She does not have a second chin.

P: Ha-a.

M: She does not have a second chin.

P: Ha-a. This is the right weight.

M: Number 5?

P: Number 5.

M: Okay. Alright, alright. So is that all you could say about number 5?

P: Yes. ((They all agree)).

M: We have chosen number 5.

P: Mh. ((Others also agree)).

M: Let us go on to number 4. Card number 4.

P: Can I put this one down?

M: Yes, we are done with her. Now it’s number 4. Some of us like number 4. What do we like? Which characteristics are appealing or desirable, or ideal, and beautiful about number 4?

P: [Number 4 is alright but her legs.

P: Legs!

P: Her legs are crooked.

P: Her legs are not all right.

P: She has a nice figure but the legs].

M: So what does she have? Her waistline is all right.

P: She has things to hold on to.

P: She has got things to hold on to and she is attractive.

P: Look at your neighbour next to you.

M: What are thing to hold on to?

P: Hips, bums.

M: Oh, bums. Okay.

P: Okay.

P: Yes.

P: Her breast is also not too small or too bit, it is medium.

P: Just portable.

P: It’s something you can desire you see? ((They laugh)).

P: I’m sorry to say that, I’m a lesbian so I love breasts so much. So, you see?

P: Oh, you are like that.

M: Okay, alright. Number 4. 152Is there anything else we want to say about number 4. Yes number 11.

P: But her legs!

M: You are worried about the legs? ((They talk at once and it is difficult for the transcriber to hear what they are saying))

P: It looks like she exercises.

M: So you are worried about legs?

P: Yes.

P: *Potries, potries.* ((A term used to describe hard calf muscles)).

M: The *potries*. You are worried about *potries*? Okay let us go to number 6 now. Let us go to number 6. This is number 6, what did we like about number 6? What did we like about number 6?

P: She is not too big.

P: Number 6 alright and fit.

M: She is fully figured? ((Some are talking on the side)). One conversation please. Are we saying number 6 is fully figured?

P: Yes.

M: What do others say?

P: No here it is too mixed up,

P: This person is too big.

M: Number 8 says she does not like this one. ((They are still talking at once)).

P: I find her body appealing.

M: Okay. Yes number 11.

P011: This one is big, she is not like number 4 because number 4 is trimmed, and you can see her waist.

M: Wait a bit number 12.

P012: Okay, sorry dear.

M: We speak one person at time. Yes number 11.

P011: This one is different from number 4 because number 4 well shaped because you can see even here she has nothing and her waist is well shaped. So you can see that number 6 is full, even here, you can see that her arms are not bigger than her.

M: Did you say she is fully figured?

P: Yes.

M: It seems most of us would love number 6 as we have chosen her and some do not really like number 6, but and because we are a group we have to understand that our preferences and things we like]

P: [Are not the same.

P: They are not the same.

M: May not be the [same.

P: Same], yes.

M: Right?

P: Yes. ((Others agree)).

M: Okay. And then men versus women – is there an ideal body shape or a body shape that we regard as nice for women and that one nice for men.

P: Eh.

P: Yes, it is there.

M: Which one is it?

P004: Have you seen that sometimes you see a person with breasts this big with flat bums. She looks like she is pushed.

P: As if they did this. ((Transcriber thinks that P is demonstrating)).

P004: ((She is demonstrating and the others laugh)), have you ever seen that?

M: Okay, right.

P: And then with broad shoulders.

M: I don’t think you understand the question guys.

P: I hear you.

M: I am asking you tell me about the ideal – the one that you say a well-built man looks like this or a woman with an ideal body shape is built like this. That was the question. Yes number 8.

P008: I want to tell you about a man.

M: Mh.

P008: This is how I want my man to be built. ((Others laugh)). I want him to have chest muscles, so that I can be able to hold him.

M: Right.

P008: He must have a 6 pack ((a 6 pack is the muscles on the abdomen which protrude and look like a 6 pack)), if he is can have a big tummy but it must not exceed 8, you see?

M: Mh.

P008: The thigh must have a “V” ((thigh muscles which look like “V”)). ((Some are laughing)).

M: Right.

P008: His legs must be slightly bracketed ((*negwegwenyana*)) you see? His hands must also be hefty and have muscles so that he can be able to lift me up.

M: To hold you. ((They laugh)). Yes number 8.

P009: Am man with big legs, hips and big bums as if he is the woman and I am the man!

M: Do you like this one?

P009: I am saying that I don’t want him to be like that and have big legs, I want a man who is just straight.

P: Without a big stomach.

P009: Yes, he must not have a big stomach, he must also not be thin, he must be okay and medium built.

M: Eh.

P009: Not that he must have hips, and legs and bums.

P: A man with hips?

P: Eh, some have them.

P: I heard you. ((They laugh)).

M: Yes number 11.

P011: I agree with her. By the way, who is she?

M: Number 9. Mh.

P011: He must have muscles, and be tough.

P: Yes.

P011: He must be hairy, be a bit taller and be all right.

M: Okay. We have spoken about men. I can tell we like men in this room. ((They all laugh)). Let us talk about an ideal body shape of a female. The ideal body shape of a female. Okay. Let me ask this question this way: Do we expect men to have a bigger weight than women?

P: No. ((Others also agree)).

P: Or, how are we saying the ideal body weight is ideal. I want you explain that one to me.

P: Am man must not be as big ((fat)) as a woman.

M: Right.

P: A man has more power than a woman.

M: Right.

P: So, a man must just be average and only have a tummy. He must not have a big body.

M: Mh.

P: Yes.

M: Okay. Are we saying that under normal circumstances a man must be smaller and have a weight that is [all right?

P: All right].

P: Not necessarily that. He must not be too small.

P: He must not be too small, he must just be all right.

P008: Sometimes when he is too small it’s a turn off, you see? At the same if he is too fat it’s a good thing.

M: M::::h, okay.

P008: He must be average and just be okay.

M: I get you. So would you expect a woman to be fat then?

P: N::::o. ((Others also agree)).

P: A woman must be fit.

M: Yes.

P: She must have a good shape.

M: Right.

P: She must just be attractive. She must have everything that shows that she is a woman, she must have all of it and be complete.

M: And then of the photos I have shown you which one is the attractive body shape, which of these would you say is an attractive body shape and weight.

P: It’s this one. It is number 6.

M: Are you still choosing number 6?

P: Yes. ((Others also agree)).

M: That her weight and shape are attractive?

P: Everything of hers.

M: Okay. Great, great fine. Okay. What informs what we are saying? Where did we get these perceptions that we have that a person should look like number 6? Where do we get it? Where did we learn it? Where did we learn about these things that make us say that a normal person should look like this? Yes number 7.

P007: The main thing is the issue of the pills neh, they have changed us and made us different from what we originally were.

M: O::::h, okay. Okay. What I am trying to ask is this? What informs our opinions, for instance, there is culture, there is family, there is media, and there is community. The things that make us agree that number 6 has the ideal body weight and shape, where do we think the foundation of those teachings comes from?

P009: Media.

M: Media, number 9. Okay, number 9 says media. Why media? Why do we think media has shaped our opinion of thinking?

P009: Most of the time in magazines and TV adverts.

P: There are no crocked people.

P009: They will also not show fat people, they will show a person with a nice body shape.

M: Mh.

P009: [You can find that maybe they are advertising a panty and bra.

P: xxxx (international female celebrity)

M: Ahhh, xxxx (international female celebrity)

P009: They will never show a fat person]. They will show a person with a nice wait line and hips. So that when you look at it you can say that bra is nice and I want to go and buy it.

M: Okay.

P009: Because you say a person who is…

M: I get you. Media, magazines. Yes number 11.

P011: I also agree with her in media.

M: Media

P011: Yes

M: Mh.

P011: As you say that a person like that …, a person with a good shape will have a normal bra size. Her panty will be nice and have a good shape, it will be attractive.

M: I get you.

P011: I will not say it’s culture because there are other males who prefer a fat woman.

P: Yes.

P011: Some Tswanas like slenders, so I will not say it’s culture.

M: Okay, alright. So you have already started on culture. What do we want to say about culture? What we are saying here,, are we in agreement with number 11 that we do not think that culture has an influence on what we are saying?

P: What culture?

M: Mh. Have we left anything out?

P: She is right.

P: My sister is right about culture, Zulus prefer fat women.

P: They say she is well-fed.

P: She has big legs and all her things are big, Tswanas prefer slender women.

M: Mh.

P: Eh, culture does have an influence.

M: Okay, so to a certain extent culture makes us pick in a certain way. And what about family? Would you say that these things we say that a person must not have a big stomach or should not be too fat and stuff like that some of them we learnt from our families or the manner in which we were socialized?

P: Yes, that one applies to family because like let’s say there is a family ceremony, you see?

M: Yes.

P: And maybe a person of a certain shape comes in then family members say: Hey we are talking about the balloon that has just left.

M: ((She exclaims)).

P: Can you see that?

P: Or maybe say that the xxxx (surname) are shaped like this.

P: Yes.

P: Not like this.

P: [Mh.

P: Yes].

P: The (surname)nare shaped like this and not like this.

M: O::::h.

P: And you feel bad].

P: They say you notice the xxx (surname) just when they enter, when they walk and when approach that this one is xxx (surname).

P: Some even say: It looks like this one is a *bhantshi* ((*ibhantshi* is a township term to express that a child is does not belong to the man – it literally means ‘jacket’)) because we are shaped like this.

P: Mh.

M: They say they have been given a *bhantshi*.

P: Yes.

P: They say this when there is a family ceremony.

P: At home we are short. So if a child is not short we say: Hey, maybe she takes after her mother’s family, we view it with suspicion because we as the xxxx (surname) are short.

M: She cannot be this tall.

P: My father was also short.

M: Okay. You want to say something number 6.

P006: They used to say that at my in-laws they would say: We are Sothos we have big legs.

M: Sorry, sorry?

P006: They say we are Sothos we have big legs.

M: Eh.

P006: Eh, when they walk around. They are looking at you whilst you are ((The transcriber thinks that she is showing them something because they burst out and laugh)).

M: You did not fit well.

P006: I don’t know what was bothering them. I know my legs are small but my body is beautiful.

P: [Eh.

M: Please wait until she finishes].

P006: They were big. Now a person would be irritated …, I was the one who was supposed to be bothered about my small legs. Now she is the one who starts and says we are Sothos because she could see that she was heavy. ((They laugh)).

M: Okay, alright. Let us now look. Oh yes number 12, yes.

P012: What I wanted to add onto that dear. When a child is born, the family usually said the child must be undresses and displayed, why must the child be undressed? I am the one who is pregnant and I know that I was impregnated by a guy from this family.

M: Yes.

P012: Why must she be undressed and displayed here? They would turn her around and exchange her between themselves. No, this head, this nose, the eyes! My goodness, she reminds me of granny, you see all of that?

M: Mh.

P012: The parents are the ones who see this. You come with a light skinned child and all of us in this family are dark skinned, they will say: Her mother knows where she is from because she does not belong to this family.

P: It’s a *bhantshi*].

P012: It’s a *bhantshi*!

M: Or she takes after the uncle.

P012: How will she like the uncle, were you impregnated by the uncle? ((They laugh)). They say that.

M: I’m kidding, I’m kidding. And then what about health services like eh, here at the clinic, the doctors and nurses. As we have picked number 5, do we think what we are saying about number 5, like these nice characteristics we picked here. Is it possible that we get some of these things when we come here at the clinic, on the talks and things like that? (.)

P: No, I do not get it, I don’t get the question.

M: Okay. I asked about family and you explained, I asked about culture and you explained. Now I am trying to establish what made you to pick number 5, I am asking whether it is possible that the education you get at the clinic from the nurses and doctor makes you think that number 5 is the ideal one?

P: [Mh-mh.

P: No.

P: No].

M: Okay, all right. What do we think makes people living with HIV to lose weight?

P: It’s stress.

M: Yes number 3.

P003: Yes, it stress. Sometimes some sleep without using a condom. There are some who do it!

P: Mh.

P003: You know that you are sick.

M: Mh.

P003: You meet someone and you do not talk. You know that you are sick but you do not talk you just sleep with him.

M: Mh.

P003: It also counts because you will lose, it will decrease your CD4 count, and the same will happen to him.

M: Mh.

P003: Am I lying? Did I learn well?

P: Mh.

M: Okay. Yes number 2 , number 1.

P001: Diet.

M: Diet?

P001: Mh.

M: Is it dieting as in trying?

P: Not eating the right food.

P: Not eating healthy.

M: Is that what you are saying?

P001: Mh.

M: Not eating healthy.

P: Nutritious food.

M: Yes. Not eating healthy. Number 11, your hand was up. That was number 1. Yes number 11.

P011: And denial.

M: Denial?

P011: Denial causes one not to take medication.

M: O::::h.

P: Yes, and being afraid of people.

P011: Meaning that you are not accepting that you are positive.

M: Mh.

P011: You have that denial and then you do not take your medication, you do not condomise, and you do not eat healthy.

M: Okay, alright. Yes number 6.

P006: Another thing is that maybe you are hiding it ((your status)) from your family, you are not able to tell them that you have this problem.

M: Mh, right.

P006: It becomes difficult to tell them. You even hide your pills from your family.

M: Mh.

P006: You are afraid that they will discriminate you, some will be disgusted with you because some of them do not understand and think that if they use the same cup you used they will also be infected.

M: Mh.

P006: They just take it the way they take it.

M: Okay.

P006: And now you are afraid to open up at home.

M: Mh.

P006: I think if you start opening up at home, it becomes easy outside.

M: M::::h.

P006: Because if you are accepted at home, even if outside you are not accepted but if you are accepted at home, there is nothing better than that.

M: Not being accepted at home. What do you say number 12?

P012: Support is very important my dear.

P012: Yes. I have been living with the virus from ’94.

M: Right.

P012: I lost 4 children.

M: You?

P012: I lost 4 children.

M: Okay.

P012: I do not have children.

M: Sorry my sister.

P012: I have changed my status, I’m no longer a female. I’m a fem, I’m a lesbian. I’m in love with another woman.

M: Mh.

P012: You like it or not, it’s up to me it’s my choice.

M: Mh.

P012: My father chased me away when he discovered that I am HIV positive. After losing children I went to live in a Home, I went around living all over. I had families. I will tell you a shocking story. I’m from a rich family.

M: Mh.

P012: xxxxx (name) the guy who used to act as xxx ((the actor)) is my uncle (.) he is my uncle. So my family did not support me, I don’t want to lie.

M: Mh.

P012: So my family …, there are those, I cannot say …, there are those who love me but they are afraid of HIV. We were not said to have HIV from ’94, they used to say we have AIDS.

P: Yes.

P012: So at home I had my own things. If I used a cup they would stop touching it.

M: Mh!

P012: Because I had AIDS and my children are all dead. My father chased me out of his house and said: Leave because you are going to kills us with your AIDS, but today I asked him and said …, the last born that he gave birth to. We were 3 at home, his last born son dies year before last, he was buried me, the one who has AIDS. I said: the one who was healthy was taken by God. Can you see how great God is? The one who has AIDS is now taking care of you and she will even close your eyes ((‘closing eyes’ is an idiom which is usually used to mean that a person closes the eyes of the dead meaning she buries him)), you see? I never got support from my father yet today we are like this ((she is showing them)).

M: Oh, you are close now.

P012: I told him to calm down. He is the most supportive and I told him and said I’m 20 years living with HIV and I’m still alive.

P: Ha.

P012: I just need your support.

M: So we are saying that not being supported makes us to lose weight?

P: Yes.

M: Okay, all right. Number 6, was your hand up? I want us to finish discussing this. Yes number 11.

P011: I want to agree with that point of not being accepted in the family. Like myself, my younger brother, the last born at home.

M: Mh.

P011: He started with TB and he was also HIV positive. So when I accompanied him …, I live at my own place but he lives at home. He is a male.

M: Yes, yes.

P011: So this is what happened …, my mother is an elderly person so she does not know the difference between HIV and AIDS. When my brother took a cup and drank from it she would want nothing to do with that cup. So called her and tried to teach her about what HIV and AIDS are. I said I’m asking you to please support him.

M: Mh.

P011: So that he does not lose weight you can see what happens, all things that are happening.

P: Oh my Goodness!

P: Rejection.

P011: Yes. So he was admitted here at G4.

M: Mh.

P011: He had lumps on his neck. What are the lumps? What are they called?

M: They are lymph nodes.

P011: Yes. So but he because fine, I encouraged him to take his TB medication for 9 months when I went to xxxx (hospital name) OPD with him, I saw that he was HIV positive. So now he is alright and he is taking his HIV medication. My mother is only understanding it now.

P: Mh.

M: Mh.

P011: And like when you talk. Do you know how elderly people are? She will say: Huuu, that person is sick, he has AIDS. I said to her mama stop saying that.

M: O::::h okay. So family support is important? It is one of the causes of our weight loss?

P: It is important. Yes, yes. ((The others also agree)).

M: Is there something else besides family support? That makes us as people who are infected with HIV to lose weight? Yes number 8.

P008: The person you are involved with in a relationship can also stress you such that you even lose weight. Like if I don’t know that my partner and I are sick.

M: Mh.

P008: So maybe we have been coughing for some time or I lose weight or I can feel that my boy is not alright, then I suggest that we go to check ((our statuses)) and he refuses and the I go alone and when I check at the hospital I am told that I am positive. The nurses counsel me and ask me who I live with and how our situation is. I explain everything and tell them that I live with somebody. Did he go to check? No, he has never been to check. Would you be able to bring him or how will it be when you tell him? You then go home to tell him, you see? With the hope that maybe he will go there tomorrow[ to the clinic.

P004: Come with you].

P008: He say: You know what? Please stop disgusting me, pack and go or I will leave you.

M: M::::h.

P008: You are the only one who is sick, I don’t know where you took that illness.

M: Okay. So it may happen that the partners do not support you like the family members. Can we now go to the next question. What can make a person who is infected with HIV to go the clinic and report that there is a weight change, for instance say that she has lost weight at the clinic but when she is put on the scale it is found that what she reported is untrue, she has not lost weight?

P003: You can say that sometimes ((They are talking at the same time and it makes it difficult to hear what each one is saying)).

M: Yes number 3.

P003: Sometimes it is here in the mind. You are depressed and when you look at yourself you think that you have lost weight. You see something like that?

M: Mh. It’s in the mind?

P003: Yes, it’s in the mind.

M: Okay. There were hands somewhere. Number 6, yes.

P006: No, I was agreeing with what she is saying.

M: Okay. What can make me to go to the clinic and report that I have lost weight and when they put me on the scale they find that I have not lost any weight like I said? (.) What could make me to say I have lost weight when I haven’t? Number 5 are you still there? ((Others laugh)). I don’t want to put you to sleep. ((They laugh)). Yes number 11.

P011: Maybe it is because you know your previous weight, how much you weighed.

M: Okay.

P011: Yes. That is when you will have lost because when you get on the scale you will have really lost weight.

M: So you are comparing it with your previous weight?

P011: With you previous weight.

M: Okay. What does number 1 and number 2 say?

P: They say they are hungry.

P001: We are saying that we are hungry. ((Others laugh)).

M: Let us eat while we continue because time is moving on. ((They are talking at the same time)). Please, please.

P: Yes.

M: Food is coming neh? My sister, yes number 12.

P012: another thing I see about the weight issue is that sometimes you will report loss of weight because you see the clothes that you wear.

M: Clothes?

P012: Yes because I used to wear size 34 and maybe now I wear size 32. That is when maybe something bothered me and then I lost weight and maybe I did not pay attention to it, when I then wear my size 34 it is too big and I now have to wear a smaller size.

M: O::::h.

P012: That is when I will realize that I lost weight.

M: The clothes will show you that you have lost eight. Yes number 8.

P008: What made me to come to the clinic and report that I had lost eight when I did not lose weight. I like to take care of myself, you see?

M: Right.

P008: I was never told by anyone to go and check my status because I know the road I used to take and the road is known by those who have walked it. So, let me wake up like that others. So what happened is that I was not sick but my CD4 count was 83 but I was alright.

M: Mh.

P008: I was not sick or anything and the doctor said: you see, you are going dies a slow death because I don’t understand what is happening. So I was panicking and thinking that maybe I had been losing weight whilst I was not aware and the people are not telling me the truth. When I got to the scale I had not lost any weight, I was just aluyby7gright. He said: You must start taking pills, I was like this lady whilst I was sick, I was alright. That is when I started taking treatment, I started taking then in November, I was just attending clinic but my CD4 count was 83.

M: That is dangerous!

P: [As from.

M: It was dangerous]. You were a danger to yourself.

P: I started attending this clinic in 2011, but I have not started taking pills because my CD4 count is high and I am healthy. I wish I could continue with the way I am taking care of myself but maybe I will take them later.

M: And then if I go to the clinic and report that I have gained weight but when they check me they find out that xxxx (facilitator’s name) is mistaken, she has not gained any weight. What could possibly make me say I have gained weight, what could cause me to make a mistake and say I have gained weight while I have not gained any weight?

P: I can just say that it is because you are depressed.

M: Number 9 says it is overeating.

P: Changing your diet.

M: When you have changed your diet?

P: Yes.

M: So you can think that you have gained weight?

P012: Yes. Or if you can drink wine when you are not used to it. ((They laugh)). Eh maybe if you are not used to drinking wine. Not liquor, like to get intoxicated but the wine you would drink maybe after a nutritious meal, you see that thing?

M: Eh.

P012: You see that type of food.

M: As soon as I have eaten this food I go to the clinic and report that I have gained weight, I tell them even before]

P012: [I can feel myself, can I not?

M: Eh.

P012: Can you not feel yourself?

M: Eh.

P012: Yes, you can feel your weight.

M: Oh you feel like you have gained weight.

P012: Yes! If a wind can blow you can feel it ((if you are light)) and you will feel hungry even if you are not. You see that thing?

M: I can hear you. what do the others say? What can make me think that I have gained weight when I have not gained any weight?

P: No that is just a thought, you will just be thinking it’s like that.

P: You are just imagining that you have lost weight. Just saying this to yourself.

M: Is all of this in your mind?

P: Yes, it is telling yourself. It is the mind, it is in the mind.

P: Mh.

P: It is not like that.

M: Do you remember that at the beginning we spoke about how gaining weight most of the time makes us feel good or happy?

P: Mh.

M: Is there someone among us who can tell me what it means to gain weight. Maybe some of the things we did not mention earlier.

P007: I am happy about gaining weight.

M: Does it make you happy number 7?

P007: I am happy about weight gain even if someone can tell me that my face has become I become very happy. It means that this thing of mine is treating me well you see?

P: Your health is alright.

M: What?

P007: My ARVs [are treating me well.

M: They are treating you well]. Okay. The ARVs are good on you or are treating you well, mh. Yes number 12.

P012: Even changing the way in which you live your life.

M: Mh.

P012: Like myself.

M: Yes.

P012: I was fem.

M: Right.

P012: I used to date males.

M: Right.

P012: I would be very sick and lose weight my dear. I was a resident patient of this hospital; they might as well could have built me a room here. ((They all laugh)). I was a real patient. I would be admitted my dear, I don’t want to lie to you, God is my witness. I used to be admitted in this hospital!

M: Were you sick as a result of your weight?

P012: Everything made me ill. I used to keep everything bottled up my dear.

M: Mh.

P012: I used to always be admitted. As soon as I changed to become a lesbian, hey *sisi*! I found love I never found before from a female person.

M: Yes.

P012: If I can say to her *baba* ((daddy)) I do not feel like going out, you see? ((Some are talking on the side and they make it difficult for the transcriber to hear properly)).

M: Yes.

P012: I call her *baba* ((daddy)) because she is butch ((a term used to describe a person who plays a male role in a lesbian relationship)), I’m fem ((a term used to describe a plays a feminine role in a lesbian relationship)), because in this relationship of ours we are also different ((Some in the groups are discussion cold drinks)), we are not all fem some are butch … [listen up please!

M: Please listen to number 12].

P012: In this relations of ours we are not all fem. There are those who are fem and those who are butch, and those who are fuge.

M: Yes.

P012: Yes. So, my partner really treats me well. She sometimes asks me how am I feeling today. You see, even that is part of being given love. You can feel it even in your body.

P: Yes.

P012: You know no one has ever asked me what you are asking me my dear … how I woke up. Just phone you 3 times a day to ask you how have you slept my love? Did you you’re your pills, [maybe if you do not live together.

M: That’s beautiful].

P012: Just phone you.

M: You are making a point of saying the change of [lifestyle.

P012: Yes, lifestyle].

M: when you live well, you feel as if you are gaining weight.

P012: My dear, I’m negative when I live well and getting support.

M: Yes.

P012: All of that. It is not even in my mind that I am sick, I just go to treatment to comply with doctor’s orders.

M: Yes.

P012: And eat nutritious food. And limit, you see?

M: Mh.

P012: Not indulge too much on parliament … ((the transcriber thinks that the participant is demonstrating something)).

M: I hear you, I hear you. Okay. Do you still remember the things we said at the beginning of our discussion?

P: Yes.

M: We talked about losing weight, we also said that at the clinic the doctor and nurse sometimes ask us if we have lost weight or not.

P012: Yes.

M: So, now my question is: what do you think is the best way to ask people who are living with the virus about losing or gaining weight? I will not point at you now number 12 because I want to also give the others a chance. I can see that you are still fit ((energetic)) because you have just joined us. I appreciate that point.

P012: It’s just that there is something that the doctor said about what you have just asked.

M: Right.

P012: He said he likes the way my body is it shows that I look well after myself.

M: Okay.

P012: It’s a compliment.

M: Okay, all right. What do others say? The doctors and nurses here at xxxx (clinic name), here at the clinic, when you come here, what do you think is the best way to ask you if you have gained or lost weight? What do you think would be the best or easiest way?

P: How do you feel about your weight?

M: How do you feel about your weight? How do they ask you at present? How do]

P: [Even their manner of approach when they talk to a person. When you talk to me you must remember that I am a patient, I am ill. You don’t know why I have lost this weight. Do not just come and say to me as a nurse when I come: Can you see this, what is your problem? What is the matter now? What happened? If you have a problem, call me to the side, do not just talk to me in the presence of other people.

M: Mh.

P: Call me aside and ask: what happened? I am also human, I will tell you what happened.

M: So you are talking about approach, they must approach you properly?

P: Yes.

M: Okay. We are trying to find out which way would be the most ideal or the best to ask about weight. For instance …, let me ask this question like this: If someone asks you if you have you lost more than a dress size or a trouser size, but for us it will be dress size – I’m going to make that example, unintentionally in the last 6 months? Do we understand that question?

P: Yes ((Others also agree)).

M: Do we understand it?

P: Yes ((They all agree)).

M: Number 1 says no. Number 7 and other this side say they understand it.

P: What?

M: Have you lost more than 1 dress size unintentionally in the last 6 months? What does that question mean?

P: This question asks if you have unintentionally lost weight.

M: O::::h, unintentionally, without intending it.

P: Yes.

P: And without being aware.

P: Without being aware.

P: Unaware.

P004: What is your problem?

M: So, can you understand number 6? Do you understand now?

P002: Unintentionally.

M: Okay, alright.

P002: Without being aware.

M: Okay. I brought along these skirts, neh? They are 3, and they are here to demonstrate this. I need one person to be a volunteer.

P: What size are they?

M: Someone who will show us what we mean when we say a person has lost more than ((some are talking on the side)). We are not going to wear them sweetheart, we are just going to demonstrate. ((They are still talking on the side)). This is number 6, I mean this is size 36.

P: 38.

M: Okay. Are you going to want to stand that side? (.) I need …, I am going to ask number 3 to stand here next to …, to stand on that side of number 2.

P: Must I wear them?

M: No sweetheart, we are not wearing them neh. I want someone to volunteer. We are trying to answer the question that says: Have you lost more than 1 dress size in the last 6 months? If a person were to give you all these skirts, from which one of them would it be, maybe size 38 to which size, so that I can see that you understand that more than 1 dress size mean?

P: I am from …, like I’m bigger than that.

M: Being from 38 to?

P: 34.

P: Yes, 34.

M: 34?

P: Yes.

M: Do we agree?

P: [Yes.

P: Yes.

P: Yes]. ((They all agree)).

M: Mh! Interesting. Number 12, do you have a different opinion?

P012: I used to wear number 36, now I wear 34.

M: Okay, so you have only … you have lost what dress size? (.)

P: You are from 36 now you wear 34?

M: Yes. You lost 1 dress size?

P: Yes. ((Others also agree)).

M: Not more than.

P: Yes.

M: So, when they ask you if you have lost more than 1 dress size, we mean you dropped from 38 to?

P: To 34.

M: 32.

P: Yes, 34 to 32.

P: More than 1.

P012: More than 1, yes.

M: Thank you so much ladies eh, for demonstrating this for me. I appreciate this. So, it is unintentionally. So, as I was busy asking this question, you are used to coming to the clinic and you understand this question. Do you think any man or any woman on the street, would understand this question and what it means?

P: [No, no.

P: I don’t believe so].

M: Is there an alternative? Is there any suggestion you have to ask this question in an easier manner … this very same question?

P012: Yes.

M: What are you saying? (.) I can’t see the one who is saying yes, sorry.

P: Number 10.

M: Yes number 10.

P: 12.

M: Number 12.

P012: The question I usually hear, like, if you used to wear 36 or 40.

M: Yes.

P012: And then you are now 32. Maybe have you have a death in your family.

M: O::::h.

P012: You see?

M: Mh.

P012: Have you lost someone in your family, someone you were very close to, you understand that thing?

M: Mh, right.

P012: And then now that you wear size 32, in my mind many things come. It is possible that maybe you were sick, it might also happen that maybe something upset you, you see?

M: Mh.

P012: After you lost that person. Because there are many things, like counseling and everything.

M: Mh.

P012: It’s not only about being HIV.

M: Yes.

P012: It’s not only HIV that can make you to lose weight ((inaudible – 01:30:50)).

M: Okay.

P012: Maybe you were raped and you are still unable to talk about that thing because it is not an easy thing to talk about.

M: Most of the time when we lose weight, for instance maybe, how do our spouses usually respond?

P: By the way, what is a spouse?

P: [Your partner.

M: A spouse is your partner]. Your partner, irrespective of whether it’s your boyfriend or your girlfriend. (.) Em::::m, number 5 you have been quiet.

P005: I think they can react a lot ((negative reaction)) because he is noticing how I have lost weight. He will ask himself: *Hawu*, you were well fed, what is eating you up? You know he will have questions.

M: Questions?

P005: Mh.

M: Are they constructive or destructive or a-derogative questions? Yes number 8.

P008: Firstly it will depend if he is supportive or not to the changes in your body when it changes.

M: Right.

P008: If he is a supportive person he will support you and he will not make you feel bad and ask you about things that will stress you, he will support you until you pick up and become alright.

M: Some will be supportive.

P: Another one will not support you, he will just tell you that: Go and get tested and if you come back with AIDS, it is yours! Because I do not have it.

P: I am not linked to that thing.

P: Yes, I am not linked to that thing!

M: So, it depends on the person you are involved with at that time?

P: Yes. ((The others also agree)).

M: Does it ever happen that there are names that they call us with after we have lost weight or the time when we are thin and the body weight has dropped, or find that your partner has left you and you end up without a partner because you lost weight [as a result of HIV?

P: You are now showing ((everyone can see that you are sick)).

P: You are showing, eh].

M: Yes number 4.

P004: You know, I don’t know. I’m talking through experience.

M: Right.

P004: I had a house at xxxx (area)

M: Aha.

P004: I went to the clinic and got tested and found to be positive. I came back home and told this man.

M: Mh.

P004: He said to me: I don’t know, I am negative. You know what happened? He would go and leave me in the house. He ended up taking the house and giving it to other estate agents. I am also an estate agent, so I jumped him and sold the house.

M: M::::h.

P004: You understand?

M: Mh.

P004: I did not get any support.

M: Yes. You did not get any support from your partner.

P004: From the partner, you understand?

M: Mh.

P004: But now, he wanted to come back, and I informed my doctor and my doctor said that I must come with him so that we can talk to him.

M: Mh.

P004: I don’t know what he is afraid of. So I am also looking at him because I am taking my treatment, I am taking my own things. If he does not want to take treatment it is his own problem. I was at home, who knows that maybe he is the one who infected me?

M: He distanced himself in other words from you? [He distanced himself and moved away from you?

P004: Yes, he distanced himself]. You see a thing like that?

M: Eh, okay. When we lose weight and it is clear that we are sick, how do our partners usually react?

P: Yhoo!

M: Does it ever happen that they distance themselves from us, or abuse us or call us by names?

P: Yes they do it.

M: Yes number 3.

P003: What I am going to say is about 1 of my friends. I am a widow neh, my husband has been dead for 8 years. He did not die as a result of HIV, he was healthy. My friend comes from the situation that the lady is asking us about. After my friend found out that she is sick, she went home to tell her husband and the husband left her in the house.

M: Left her?

P003: Left her.

M: So, sometimes they leave us?

P003: [Yes.

P: Yes]. ((Others also agree with this)).

M: Alright, okay. Yes number 8, sorry.

P008: I went to test, and I reported at home that I am positive but I got a lot of support. But my husband does not talk but I can see him. Not that he is sick but there are things like his skin has changed maybe it is the symptoms you see? And two of his wives died. But he is quiet, he is supportive towards all of this, he does not say anything that makes me to feel bad.

M: Okay.

P008: I tell him that it is still his right if he does not want to talk. You see that thing?

M: Yes.

P008: Because everybody has a right to speak out at a time when he feels like speaking.

M: Ye::::s. And what about children?

P: [Oh!

P: That one is difficult!

P: Oh no!

P: It’s tough]!

M: Can I have number 6, can I have number 6.

P006: My sister, I got sick in xxx (year).

M: Yes.

P006: I think it’s only been 3 years since my children knew that I am ill.

M: You mean this past 3 years?

P006: Yes.

M: The past one?

P006: Yes. I used to be accompanied by the eldest one. When people talked about this thing I would feel like chasing him out, it was difficult for me to tell him.

P: My Lord!

P006: he would come with me but he would not be understanding what was going on.

M: To him mom.

P006: When I was sitting and a person came and said HIV something, something, I would say to him: Please go outside. ((She laughs)).

M: You would chase him way?

P: My Lord!

P006: It was sad.

M: Otherwise, what was their reaction as you indicate that they have known for 3 years.

P006: They did not understand. When they pills were introduced I was already sick. As soon as they were introduced I got them. When they were shown on TV I would change the channel.

M: Mh.

P006: So that they would not see what the pills were for and that I had the same pills in the house.

P: [Awu!

M: Oka::::y].

P006: I would think that they were seeing them. They are 2, a girl and a boy.

M: Eh.

P006: They fetched their granny and told her. They said mom why did you not tell us? I cried and we all cried in the house.

M: A::::h!

P006: I told them that it was difficult and I thought you were going to be disgusted with me. But after that I realized that I became relaxed.

M: Mh. But what was their reaction after all that, how did they treat you after they found out?

P006: They did not understand what was happening because I was always angry.

M: Mh.

P006: I was always angry and shouting. The boy even said you know what? You have a problem and you don’t want to talk.

M: Mh.

P006: I thought that the girl saw something because she is the one who used to accompany me so I wanted to divert her attention from that. I always shouted at her till they discovered.

P: Jesus!

P006: They ended up talking and saying that this is this disease and you do not want to tell us about it.

M: But in the end did the support you?

P006: They supported me a lot, they supported me.

M: Okay. Yes number 5.

P005: Eh I want to say it is difficult. Like my mother, my mother started being ill a long time ago, in (year).

M: Mh.

P005: It was June.

M: Your mother, yes.

P006: When she was first admitted in hospital, she came back and hid it. We were taught at school how a person with HIV looks like ((symptoms of HIV)).

M: Yes.

P006: I thought to myself that the things that I am told at school, I am now seeing on my mother.

M: Yes.

P006: Let me steal mom’s file then go and read it.

M: Yes.

P006: Maybe I am going to find something. I found it. I cried the whole night but when she told me about her life story she told me that her mother dumped her in the veld and she was brought by a certain man that she did not know, he just brought him. Our house is near a veld, so when this man brought her she was welcomed by the neighbours. She was raised by the neighbours. So as she was rejected at home, I decided to see how I was going to cope with my mother.

M: So what about you now? I’m trying to cut it a little shorter. As a child after you saw the file and found out that your mom is HIV positive, losing weight and stuff, how did it affect you? How did you react after you found out that mom has the virus?

P006: I was very angry.

M: You were angry. What made you angry?

P006: About why she was hiding it from us and not telling us.

M: Okay.

P006: I was thinking about a lot of things.

M: And then in the end?

P006: In the end, I ended up supporting her because my sisters kept their distance.

M: Okay.

P006: Everyone. No one supported her.

M: All right. Yes number 11.

P011: When I found out. I decided to go and check at NAR because I worked here at ((inaudible – 01:39:35)). So, my child was a clerk.

M: Mh.

P011: Here at the mortuary.

M: Yes.

P011: So after finding out that I was HIV positive I was counseled and told to tell one person so that I could start medication. The doctor asks you who do you live with? A person [who will check you.

P: Who will check you], yes.

P011: Who will help you with this and that with regard to medication.

M: Yes.

P011: So I told my eldest child, the one who also worked here. So, she was so supportive.

M: Mh.

P011: She was working so she found somebody for me – my cousin, we told her so she was the only one who knew. My cousin and my mother.

M: Mh.

P011: So I got support from my mother.

M: Mh. So, most of the time they support us?

P: They support us. ((Others also agree)).

M: Family. We are talking about children and our parents.

P: They support us.

M: Okay. What about the extended family?

P: Jo!

M: Aunts.

P012: Can I please comment there.

M: Yes number 2. Sorry.

P002: Okay. When I found out that I was ill my mom called the family .

M: Right.

P002: Both from my father’s side and her side and told them. Then otherwise they were very supportive.

M: Support.

P002: Yes.

M: When we say they supported us, what do they do that makes us think that they are supportive?

P002: They loved me and told me that I was not the first one.

M: Okay, oh.

P002: You see.

M: Mh.

P002: And if I accept myself I will be alright.

M: Right.

P002: And then when it comes to the children. The boy who is my first born, is now 17. He is the one who knew. The youngest one is not 12.

M: Okay.

P002: I did not tell her anything.

M: Okay.

P002: I only told her now because I thought that it was going to disturb her at school.

M: O::::kay.

P002: You see? Only to find that she was more supportive than any person.

M: My goodness!

P002: When it is time to take my pills she brings them and says mom it is not time for you to take your pills.

M: Haaa!

P002: When it is time to eat, she brings food and says mom this is what you are supposed to eat. You see?

M: Mh.

P002: Mama we must gym, you see?

M: Okay.

P002: So when I told her I first asked if she knew what the pills were for and she said yes.

M: My goodness!

P002: That’s why I gave them to you, I want you to get better so that you can raise my children.

M: *Hawu bakithi*. ((Others also exclaim)).

P: Hawu Jesus!

M: Okay so we get support most of the time from family?

P: Yes.

M: And what about from friends? Yes number 4.

P004: You know I have got a friend, even today she’s my friend. ((Some are talking and making noise)).

M: Can we please wait a minute. I wonder what are they doing to us? I wonder what are they doing to us. Let us now listen to number 4.

P004: I’ve got a friend. Can I please talk *sisi*. I’ve got a friend we live on the same street.

M: Yes.

P004: We grew up …, we were kids and used to play together. When I was sick she was supportive. When I was admitted here at xxxx, she was supportive. Even when I was discharged from hospital and went home and my sister was at work she would fetch me and take me to her home and give me a bath. At home it’s a two roomed house, so we don’t have a bathroom and they have a bathroom at her home.

M: Haaa!

P004: She would bath me. She works at xxxx (company name) and she would bring me salads. Every weekend I would not sleep at home but I would sleep at her place. Until today, we are still friends.

M: Haaa.

P004: No friend has ever been like her. No matter what the situation she would bath me. She should make me Morvite in the morning, being late for work, because she wanted to make sure that I had eaten.

P: Yes, make sure.

P004: I would eat until I was full and she would wipe my mouth and say bye-bye, I’ll see you. When she entered she would leave her bag and go to the pots and come to me in the bedroom. *Sisi* how are you? How did you sleep? She would come and massage my feet because feet tend to lock when one becomes ill.

P: Mh.

P004: She would massage me and we would wake up. We would go around the house whilst she was helping me to exercise.

M: So that you’d be fit.

P004: Eh. When we got back she would put me into a bathtub and bath me. Sunday afternoon, she would accompany me home with a food tin, [taking me home.

M: Taking you home now].

P004: I had a friend, and I will never forget her even now.

M: So number 4 had a positive experience of disclosing to a friend and she was kind to her. Do we have a person with a different story from a supportive friend? Yes number 9.

P009: What will I say? It differs, you understand?

M: Mh.

P009: Sometimes you get those friends who are supportive.

M: Yes.

P009: And then you get those friends, all they do is go around talking about you.

P: Yes.

P009: Do you see her? She is dying!

P: Mh, they judge.

P009: So with me, it’s like that. I had those friends who were supportive. It was my neighbours who were actually supportive.

M: Mh.

P009: They were very supportive.

M: Friends and neighbours?

P009: Yes. When I started to become ill they were there for myself.

P004: Oh yes.

P009: And the ones I was expecting to be of help were not there instead they were going around spreading that did you see that she is dying?

P: Mh!

M: A::::h.

P009: You understand?

M: Mh.

P009: So it happens in 2 ways.

M: I get you, I see.

P004: It makes you worse and the weight can even go down because you will be thinking of these people who are gossiping about you, you don’t think about your health. That’s why they say that if I disclose to you there is no need for you to go around to planting it outside because you don’t know what your status is.

M: Okay number 4. Let us hear from number 8 now. Sometimes they support us and sometimes they discriminate against us and gossip about us. Yes number 8.

P: It’s like that.

P008: I had a friend who passed away. In my father’s side of the family, where I grew up, they were 12 and two people there died as a result of this disease.

M: Mh.

P008: So at home I used to help those who were sick.

M: Mh..

P008: I remember that the last person who was sick was my cousin. I was not on good terms with my aunt but my cousin was sick. He was 23 and I would put adult Cimbbies ((disposable napkins)) on him.

M: Mh.

P008: So I am from that situation, I am so strong that I was able to cope with all situations. I also knew it …, my aunt, the one who recently passed away, used to say I must always be accessible because I was the only person who was able to look after him, you see?

M: Mh.

P008: So, in the end I helped a person who was sick. When I spoke about bones heavy bone. I would see how bones are on a person, I would see how bones combine on a person ((skeleton)), you see? So I know what a person looks like when he is sick, the flesh becomes finished. I lived with that person for 8 months. I also had a sick friend, my best friend. I will never have a friend like that in this world. I also nursed my friend because I knew that if I had been the one who was sick, my friend would have done the same thing, you see?

M: M::::h.

P008: That’s why I nursed her, I nursed her so much that I could not even talk at her funeral. I just said, my friend, what I have done for you I do not even want to be thanked or praised because I know you would have done the same thing for me.

M: Mh.

P008: You were also going to carry me. You were going to bath me, you were going to do my laundry like I did for you. So, it depends what kind of friends you have. And you must stop talking to a person that you know is a radio.

M: A radio. Yes number 3.

P003: I wanted to talk about family.

M: Mh.

P003: I started to come here and it was fine. I used to accompany my friends to come here, I liked things so I accompanied them here.

M: Mh.

P003: I did not know that I would also have this disease.

M: Mh.

P003: Then one day when I had accompanied one of my friends, I asked to open a file, I checked and discovered that I was ill. I went home to tell my mom.

M: Yes.

P003: Mama I am now like this. My mom: Hawu my child, this and that. Only to find out that she told this to my younger siblings who are male twins, she told one of them who lives in our yard that your sister is now like this.

M: Yes.

P003: One day he had a fight with my child.

M: Yes.

P003: He insulted my child. That your mom has HIV, she has AIDS. ((Others exclaim)). So some families will be supportive, they are not the same. ((They talk at the same time and it is difficult for the transcriber to hear what they are saying)).

P008: People in the world are different.

P003: By that time I had not told my [child that

M: The child]

P003: I am now like this. But he heard it when his uncle was talking about it.

M: He was now being insulted about your disease.

P003: He was insulted by it.

M: And what about the community? I think number 9 had already hinted on being gossiped about and things like that. And I think we also]

P009: [They gossip a lot. There’s one thing that I will tell you, if you are not strong [when they laugh at you

P: You will fall, you will fall].

P009: ‘Cause, let me tell you something. When I tested HIV positive, I thought okay fine, who am I going to tell? I thought of my mom.

M: Mh.

P009: When I went to my mom, do you know what my mom said? I won’t be part of that, see how you sort yourself out with that boy who infected you with the AIDS you have. My own mom told me that but I still love her very much, I do not hate her, I love her because whatever she told me made me the person that I am today.

P: Mh.

P009: So, you tell yourself as a person where you want to be. If you are going to listen to what people say.

P012: You won’t survive.

P009: They are going to stress you, you will keep thinking what so-and-so said and you will not survive. This is earth, you won’t!

M: Mh.

P004: To add onto that my sister, I have one sibling and it is just the 2 of us who are left now, our last born sibling went missing in 2005. I got divorced and came back home sick. When she pushed me here … I went to the same school with Sister xxxxx (name). When she pushed me she would tell say to me you are annoying, stand up because you are just pretending to be sick!

M: Was that said by your sister?

P004: Yes. My younger sister who comes after me. In my opinion I thought o::::h, I am now back and she is living at home with her boyfriend.

M: Your sister?

P004: So it means I am now the problem, you understand?

M: Oh, she lived with a boyfriend in your yard?

P004: In the house.

M: O::::h.

P004: Do they know that you are rotten underneath and taking ARVs?

M: Where are you rotten?

P: Underneath ((the genitals)).

P: Eh, that is what they say.

P004: You know because God is good.

M: You did not mention that name at first! ((They talk at the same time)).

P: [I told you that there are many names for this thing.

P: It is known as many names].

P004: You know what, let me tell you. the one I serve is great at all times. He is alive, he does not slumber or sleep! I got up. She ((the participant’s younger sister)) went around to our extended families and asked who had put me on their policies. Can you believe it? I am not your child. I went back to xxxx ((estate agents)) to go and work. I bought myself a new bedroom suite and I bought myself everything, do you understand me?

M: Yes.

P004: She herself is the one who now wants money from me, she wants money from me. Please lend me so much. Please lend me I will give it back. When the end of the month arrives she says: you know at the bank, and I say: hey don’t make me a fool, I also know how banks work so stop making me a fool. You understand me?

M: Okay, alright. Actually I can see that you like the issue of the family members. I was now trying to hear if there is anything we wanted to say about the community. The way the community responds when they realize maybe that we are ill such that our weights go bad and we become very thin. Yes number 8.

P008: You see in the community, I want to tell you *sisi*, HIV is really a problem because as a person you relies how much HIV has become the common cold ((the participant likens HIV to the common cold to illustrate how common HIV has become common and prevalent in communities)) because it is now a flu. Every home has experienced it.

P004: Yes. ((Others also agree)).

P008: None! You know when my aunt was sick. My aunt was very well mannered but she became sick and died. Before she died …, I overheard ladies who live I think two houses from my parental home talking to each other … they are the same age as my late aunt and they were saying: Hawu xxxx ((the participant’s aunt)), a red and white tent will be erected.

M: They were talking about a tent? O::::h, meaning she was going to die.

P008: Yes, a tent is going to be erected. I just kept quiet. I was still young. Eventually my aunt died and their will happened. My aunt died and was buried. I later met them on the street when I was a bit older. Hey baby are you okay? And I said: hey, Jehovah is still preserving us from tents that get erected. ((They laugh)). Jehovah is great and he is able to preserve people from tents because the thing that is finishing people at home goes by house numbers.

P: Yes.

P008: It will go to 48, enter 49. Even at house number 50 it will go and 51 as well. I said, just relax my sister, because it also goes the colours of houses so it will also come. Like you have a blue coloured house, it will also come to our red coloured house. ((They laugh)). I said to her you see the tents will be erected! ((Others also concur with her)).

M: And then what about health care workers, the doctors, nurses and counselors. When we lose a lot of weight, how do they respond most of the time? How do they treat us? Number 10, do you want to say something?

P010: They console us. Like my doctor tell me that you will be okay, and send you to a dietician so that you will eat well, like in my case I was getting porridge ((some nutritional powders with can be mixed and eaten as a porridge)) because my weight was very low.

M: Mh.

P010: So I ate that porridge until I became alright. She would say to me I love you and I would also say I love her.

M: Awu. That’s beautiful. What do others say? What do they say when they console you?

P: They are supportive you know, yes, they are supportive.

P008: My aunt works in xxx (town name) and she is positive.

M: At xxxxx (rural municipality)

P008: Yes, she teaches there. She says to me, you know xxxx (name), I do not go to the doctor because as soon as you leave ((the consulting room)) then she turns to the nurse and says, you see that one with a red and black outfit. She is dying. A doctor! She says I do not even think about it. And I say to her, aunty! And she says to me here at xxx (town name) I am known as a sick person because of this doctor. ((The others laugh)). He says you see that one? She is sick, she is finished and she can die anytime. ((They continue to laugh)).

M: So, it is not the same. Sometimes they console and support us. Whilst others talk badly about us. Does anyone want to say something else about health care workers?

P008: But mostly they are supportive.

M: Yes number 6.

P006: I was once admitted here at xxxx (hospital name).

M: Yes.

P006: My sibling came to fetch while I was sick and the doctor said to her please give her these pills, if she does not take them you are going to lose her anytime. ((They laugh)).

M: She scared the child! ((They are still laughing)).

P006: He said you are going to lose her anytime. She must take these pills.

M: So, you took them. I’m sure they were also afraid.

P006: And you also swallow them with a hurt heart ((they laugh)) he said I am going to die.

P004: Yes, it also happened to me when my sister came to fetch and she asked the doctor why are you discharging her? The doctor said: This woman is strong so she must go home. She said: why don’t you keep her for another month? He said: No, she’s strong. I thought to myself that he was afraid that I would need to bathed ((be a burden)). So I would say please bring me some water so that I can bath myself.

M: But in all we have said about the way we were treated by our children, our partners, extended families, friends and the community and healthcare workers. How does it make us feel to be in the condition that we are in? Yes number 9.

P009: I feel happy, not because they are around me but because I choose to be happy, happiness is a choice.

M: M::::h. You chose to be happy?

P009: Mh.

M: That’s interesting and profound. Yes number 8.

P008: I am happy because knowing your status feels like a heavy burden has been lifted from you.

P: Yes.

P008: Unlike a person who does not know his or her status. Unlike a person who does not know you see? Now you know the kind of life you must live because you now know. you see, a person who does not know his or her status is lower ((inferior)) than me.

M: Mh.

P008: I see myself as your President. ((The others laugh)).

M: Mh.

P008: Because I know what I am supposed to do. ((They are still laughing)). Hey I am your President really.

M: Alright, number 12.

P008: Because I am alright and I know my status.

P: I know my status.

M: Okay. We do not have an English one. (.) Oh, here it is, here it is. Sorry my dear. We need a stapler there. Okay, alright. So, most of the time we sound like we are happy about the responses we have received. Number 1 no number 12, we want you to share that with us. Yes number 12.

P012: I wanted to address this issue of ((inaudible – 01:56:53)).

M: May we please finish first because we are running a little bit short of time.

P012: For me it is the issue of knowing your status my dear. The issue of knowing your status is very important because I have known about it since xxx (year), I’m very proud because it has been 20 years and there are people I used to attend the clinic with who are no longer with us ((they died)). My cousin passed away in front of me. I would advise her and tell her to limit alcohol intake, limit boyfriends. I can see you are beautiful and all that. Reduce your pride.

M: Mh.

P012: What hurts the most is that my father dates people who are positive. I attend the clinic with them here. Elderly women who are HIV positive. They even say it, one of them recently passed away and she lives near my parental home in xxx (area). She used to say: I bulldoze ((ngiyakudodloza)) and if a person does not want a condom I share my HIV with him. So I gave my father some space to find himself.

M: Mh.

P012: So I told my father that did you know that the person who passed away was HIV positive and attended the same clinic? He said: I now know that because I heard about it after she died. I was not at her funeral but I knew because we used to come here with her. My father did not know it. I don’t even know if they used a condom or not.

P: I don’t think *baba* used a condom.

P012: I’m not sure my dear because they were drinking.

M: Okay, what are we saying? We are saying that we are happy. We have accepted because we know.

P: Yes.

M: And we have chosen to be happy.

P: Yes ((they all agree))).

P: We walk with confidence.

M: Does anyone have a different feeling from happiness. Yes number 2.

P002: And I can even teach a person on how to live because I know from myself.

M: Mh.

P002: And then I can support a person who comes to me and says she is sick because I am from there, I experienced this thing.

M: Yes, yes.

P002: And then I can even advise the family on how to treat her.

M: Mh. Okay. I also spoke about how we lost weight when we were ill but then later regained it, gained weight and got to the position we are in right now. How did our spouses feel when we got back to our normal weight? When we lost weight after having been overweight or after gaining weight?

P: I do not even want to see a spouse. I do not have a spouse.

P: I also do not have it.

P007: I do not even want to know.

P: I also do not have a spouse.

M: Number 7 says she does not even want to know. Number 8 ((they laugh)) does not want to know or even comment, ((they all laugh)).

P: In my case, he was supportive.

M: He was supportive when you got back to your weight. Yes number 2.

P012: Mine is very supportive.

M: Does anyone say the partner was not supportive when she gained from having lost weight?

P: Those who have partners must talk.

M: And what about children – children. We were ill and now we are going back to our normal weight.

P: [They become happy ((Others concur)).

P: Our children are happy.

P: They are happy].

M: They become happy.

P: They even say God we are grateful. We can see that you are coming along fine. They are very happy.

P002: They even tell you about the time.

M: Sorry?

P002: That’s why I say they even tell you about the time.

P: Yes, they tell you about the time.

M: Oh even the time? They will remind of the time.

P: [Yes.

P: Yes].

M: They remind you of the time to take pills?

P: Yes.

M: Okay. And what about the extended family? Do comments change when we go back to our normal body weight? Yes number 12.

P012: Like my family. Last year I turned xxxx years old, I was going to turn xxxx years old in xxx (month) . My cousin said to me, she cried and said: Cousy, I am grateful to God that we are going to see a new year with you, we are going to be together in the new year ((F is saying something inaudible to sis’ Jessie but it is not part of the discussion)). You went through so many things and lost children are still here with us.

M: Mh.

P012: And what I like the most is that I am able to advise her and say Cousy if you also want to go and get tested feel free to and then talk to me. If something is bothering you come to me. So she took my advice and said: when I want to go and test I will not run around because I know that here is a person who can counsel me and I will not go to the clinic because you are here and I will get everything from you.

M: Mh.

P012: But she is very supportive.

M: That’s great. So we get support even from extended families?

P: [Eh.

P: Yes].

M: Okay, then what about friends? They say certain thing when we lose weight, do they sing a different tune when we gain weight?

P: Sometimes it does not change. It does not change sometimes.

P012: Sometimes it does not change.

P: Some say she is now outgoing can you see.

P: I can understand being outgoing, some say: she is taking pills. The pills are really treating her well, it is making her fat.

M: O::::h, so even when you gain weight they say it is due to the pills.

P: They say: you are even able to shake your bum.

M: Ha-ha-ha!

P: Yes.

P008: You don’t know contemptuous people are.

M: Mh. And then nurses, doctors, counselors, and health care workers. How do they react when they see that you have gained weight?

P: They become happy. ((They are talking at the same time and it is difficult to hear what each of them is saying but they agree that healthcare workers are happy about their weight gain)).

P: They say you do not look the same way you did when you came here.

P: They say you are attractive, you do not look the same way you did when you first came here. Look at how beautiful you are. ((others also concur)).

M: Okay. I want us now talk about body shape changes. I think this is almost our last question. Body shape changes. Usually how do the bodies of people who are living with HIV change? How do their bodies change?

P: They develop big breasts.

M: Yes.

P: They develop shoulders.

M: Shoulders.

P: Their bums become flat. They develop ((namanqina – the translator cannot translate this)).

M: Please slow down a bit, you are too fast. It’s a flat bum, it’s big breasts, big tummy. Big …, broad shoulders ((some are talking at the same time on the side and they make others to be inaudible because they are making noise)). Okay, alright. Is there a difference in the way body shapes change between males and females? That women become like this, mean become like this? Or is it the same?

P: It is the same ((They say this in unison)).

P: It is all ruined and the same.

P011: What I have noticed on males is that they ((bapotoka – the translator is unable to translate this)) a lot here.

P012: Yes, and this bone becomes exposed.

M: Eh.

P: And then some develop breasts.

P: They use alcohol to deal with their stress.

P: Some develop breasts like women.

P: Men are ((inaudible – 02:04:16)) but we take care of ourselves.

M: So most of the time they lose fat on their face?

P: Yes. ((others also agree))

M: And then they develop breasts.

P: And here and become big here.

P: The neck.

M: Mh.

P: Eh. They just look like frogs.

M: Okay. ((Some are laughing)).

P: Like a Robocop because you can’t tell if he is going this way or that way ((they laugh)). I am only explaining it the way I see it.

M: Yes. Let us give her a chance. Number 5, I am going to give you a chance.

P: He liked me shame I do not want to lie.

M: He admired you?

P: Unfortunately he is one of these. ((Transcriber thinks that the participant is demonstrating with hand signals)).

M: What is he, is he a police officer?

P: No, he is a prisoner. He is very handsome. But hey! Here I do not understand whether he lifts weights or what. In my mind I decided to just leave him ((inaudible 02:05:18)).

M: Wait! Were these flat bums caused by the fact that he has HIV?

P: Yes, ye.

M: By HIV?

P: He was not like this.

P: Eh, the people we are talking about are those who look like us, who go the same school with us.

M: O::::h. Yes number 5. You were discussing something with number 8 we also want to hear it, we beg you.

P008: It was about the shapes that we were talking about.

M: What are you saying about the shape?

P008: We were saying males sometimes have legs which look like a beer bottle which has been inverted. ((They laugh)).

M: What …, do they]

P008: [And then they have exposed veins as if they gym.

M: Exposed?

P008: Eh.

P: Eh, As if they are swollen neh?

M: Okay. So you are finished explaining about males. Interesting enough there is not a single male in sight, so you are busy gossiping about them. What about women? Most of the time when a female’s body changes what does it change to?

P012: For me it started with breasts and a flat bum.

P: A flat bum.

P: And legs.

P: Breasts.

P: I developed breasts and had a flat bum.

P: [Females change and lose the bums.

P004: The doctor changed my pills].

P: And then the legs change and become very small, in females. Yes.

P: ((Ankles – the translator does not know how to translate this)).

M: Okay, we have already said that, most of the time that is known as lipodystrophy. Now I am trying to find out what exactly causes these body shape changes that you talk about.

P: [It is the pills.

P: Pills]. ((They all concur)).

M: Pills?

P: Yes. ((They all concur)).

M: Is there any of these changes that is caused by HIV itself as a condition that you can say before I took ARVs, I was like that, before I knew about my status. When I became infected and it started progressing in my body I noticed 4, 5, and 6 as changes? Yes my sister?

P: Yes I can say they are there because here on my face I looked like I had dust. ((They laugh)).

M: But I like it because she herself is laughing ((F laughs)). What did it look like you had?

P: It looked like I had dandruff.

M: O::::h, I get you. The layer on the skin?

P: Yes.

M: Okay. We are talking about body shape neh?

P: Yes.

M: But it is a change in the skin. Yes number 3.

P003: If I can start taking pills and I change and I notice the changes on my body. For now I am not taking any pills and my body is still right.

M: Mh.

P003: But as soon I start taking it and my shape changes, I will say it is the pill that has changed me because right now I am alright.

M: Alright.

P003: It is not what I have right now. I will say I was changed by the pills.

M: So for now there is no shape change, okay. What does number 12 say? ((They laugh)). We are talking about changing in form.

P012: Before I became positive.

M: Yes.

P012: Before I started taking the pills, I was a girl amongst girls. ((They laugh)). I was confident! I would wear a 2 centimeter skirt ((2cm is a term used in the townships to indicate a mini-skirt)). I would never wear that now my dear! ((They laugh)). My legs are very small! I am very afraid of it!

M: What changed them? Did they just change?

P012: The pills.

M: Okay.

P012: In xxx (year), my body started changing. The breast became the size of Dolly Parton’s breast ((the others laugh)). The breast! I used to wear size 36B, and now I wear size 36 double D ((DD)) and I am not find my bras at any other place but at xxxx (shop name).

P: Yes.

P012: And they are very expensive. The other issue is here, when you wear a straight cut pair of pants they become like this.

M: I like the way you are explaining it. ((They laugh)).

P012: When you want to wear a pair of pants that is skinny you feel like picking a smaller size so as to reveal your body looking like the one of xxxxx (international celebrity) but when you look at yourself ((inaudible – 02:09:54)). You take a long time looking at the mirror my dear. Since I started taking the pills those are the changes I noticed. When you leave the house you say: I want to be attractive when I walk on the street. Yes I am proud of knowing.

M: Yes.

P012: But there is that thing that reminds me that my body is not the same as it was before. I will never wear a 2 centimeters; I don’t want to lie to you my dear. Even on the tummy.

M: The pills change us.

P012: [They change us.

P: They change us too much].

M: Okay. The pills have a disadvantage in that, we end up being afraid to wear things we used to wear because we are embarrassed about the things that might be revealed.

P: Yes ((they agree)).

M: We are afraid that we will reveal things that were not there previously.

P: Yes.

M: Okay. Most of the time since we became like this, since our bodies have changed as a result of taking ARVs, how do our spouses respond to that? This change of body shapes as a result of ARVs? I am not going to point at anyone now. Yes number 8.

P008: Mine only commented about my big tummy as if ((inaudible – 02:11:22)). But he can tell that I feel bad about my tummy so he asks me what is wrong with the big tummy.

M: Mh.

P008: I can see you are complaining about a big tummy, anyone can have a big tummy.

M: So your partner is supportive? Is there anyone here who says that after our bodies changed the response we got was not a good one? (.) Okay. It seems to me it’s either we do not have partners.

P012: I have a girlfriend.

M: A girlfriend is also a partner.

P: Yes, she is a partner.

M: And children. What did the children say when they saw our bodies changing? Yes number 6.

P006: My children are happy because I am not feeling any pain.

M: Okay. They are happy because you are not feeling pains?

P006: Yes.

M: Did someone get a negative reaction from her children about the change of body shape? Yes number 2.

P002: In the case of my kids …, I do not like …, since I was growing I did not like …, I always had a big body.

M: Mh.

P002: I do not like to wear …, maybe when I wear pants then wear a shirt that will reveal here like this.

M: Yes.

P002: So, now that I have lost weight, when I get dressed and wear something that hides my bums they say: why are you making yourself a granny? You must wear the latest fashion, and I say: hey I am not a child. So they do not like those ones, the want me to wear what they want me to wear, you see? Even applying things on my face all that stuff ((make-up)), the girl says: Mama, why don’t you put this thing here? I then say: No, I don’t put those things on! And they say: you are still very young, you are beautiful so you must put these things on.

M: So they want you to wear the things you used to wear before?

P002: Yes.

M: But you have changed and you don’t do that anymore?

P002: Eh.

M: Okay, all right. In our extended family members, is there anyone who makes a comment about something and say you have small legs or you have big breasts and stuff like that? Yes number 5.

P005: At home we had an aunt, so they would say: Aunt such small drumsticks! Such small drumsticks, what is happening?

M: How small were they?

P005: They used to be big but now they were small.

M: You mean here it was big?

P005: Yes.

M: My goodness! Okay, okay. So how did the aunt take that comment? Was it just a joke or did it make her feel uncomfortable?

P005: She would laugh and say: I will get fat soon, I will be like you.

M: My goodness! Eh.

P005: Mh.

M: How does the community usually say when they see these changes in the body shapes of people who have started taking ARVs.

P: They asked me what happened because I used to have a nice body but I talked and told them that it is because I am sick, so?

M: Mh.

P: And I have started taking ARV and they say sorry, you will be fine.

M: *Awu bakithi*. That was not bad – support.

P: Yes.

M: Yes number 7. Do you want to say something?

P007: No they say nothing, they just look at you.

M: They?

P007: They just look at you. They just judge for themselves that you are like this and you used to be like this, but they do not comment.

M: O::::h.

P007: But you can tell that they are talking.

M: I see. But what about the healthcare workers. Oh yes number 6, sorry.

P006: Like me. When I started being sick my eyes changed. My eyes were okay.

M: Really?

P006: So, there were many talks …, what happened to the eyes, I would say it is this disease that I have.

M: Aha.

P006: And leave them alone.

M: Did the eyes change after you started taking ARVs or were they changed by the virus?

P006: It started when …, it was before I started taking them.

M: That was before you started using them?

P: [Yes, it’s before.

P006: Yes.

M: Okay. They changed from what to what?

P: I had something called a belt ((shingles)). I don’t know what they call it.

P008: Shingles.

M: Shingles. On one eye?

P: Eh.

M: Okay.

P006: Sometimes they criticize me but I do not care because my children love me, [I don’t care what another person says.

P: Oh yes]! ((Others also agree with her))

P: Another person from outside, forget about him or her, there is nothing that he will do for you.

M: We are very grateful for your time. I am very thankful.

P012: You are just thanking us because you wanted to laugh. ((They all laugh)) You only wanted to laugh. ((They continue laughing)).

M: Can you guys tell that I like to laugh?

P008: You would bow your head down and laugh! ((They continue laughing)).

P004: When I started to pick up weight, this thing makes you to have broad shoulders.

P: Yes.

P004: When I go the wardrobe, then my daughter says: what do you want so that I can take it out for you? And I answer and say: yoh I am on a hanger! ((They all laugh)). The shoulders are like this at that time. I have nice dresses and beautiful clothes but they just hang. She asks: Why do you wear this and not wear that? I then say: No I am okay. She says: who says you are on a hanger? I say: can you not see that my shoulders are like this? The shoulders become pronounced as if the clothes are on a hanger.

M: Mh, mina what I like about the whole thing is that you have accepted and you are making fun.

P: Yes.

M: And you are sharing with each other how have you been coping all along and you are making one another realize that whatever they are going through, some of you go through those things.

P: Yes.

M: And now that you are talking about clothes and all that. On the groups we have had we noted that sometimes people lose weight such that pants no longer fit and so they wear many pants in order to fit into a certain pair of pants. As a result that has an effect on the scale. Is that common?

P: [Yes.

P: Yes, it happens.

P: It happens.

P: They wear many clothes].

M: Do you see it or do you tell it amongst yourselves that today I have on many pants or a tight underneath?

P004: You know how it happens? You may find that a person is open to you and so she tells you that.

M: Eh.

P004: Yes, because sometimes there are those who do not want to ask you anything and they will not also not ask you thing. Another one may just come and greet you only. When we go that side we pray.

M: Oh!

P004: We pray and we also sing. We converse.

P: We are sharing.

P004: When will you be coming back? And you tell them when your next appointment is. It depends on you whether or not you still want to bottle this thing up. That side you find yourself [another family.

P008: Friends, you see that?

M: Yes.

P: You understand?

M: Oka::::y. Thank you so much guys.

P: We also thank you.

P: Please give me your number.

P: We thank you. ((They talk and laugh)

M: End of discussion, the time now is xxxx minutes. End of the focus group discussion at xxxx (clinic name) with xxx participants.

**END OF DISCUSSION**
