# Supplementary material for: “If we lose it, we are worried”: Individual and provider level perceptions towards weight change among people living with HIV who undergo TB screening in routine health care settings in Gauteng Province, South Africa
Source: PLoS One. 2025 Sep 22;20(9):e0331904. doi: 10.1371/journal.pone.0331904 (PMC12453174; doi:10.1371/journal.pone.0331904)
Supplement: S4 File — (ZIP) [file pone.0331904.s004.zip › S4 Transcripts_final/FGD 4.docx]

FGD 4

Transcribing Conventions

- **...** Ellipses indicate talk omitted from the data segment
- **(( ))** The transcriber’s comments.
- **( )** Empty parentheses indicate some talk was not audible or interpretable at all (we include the line for instance 20:15)
- **(.)** A dot enclosed in parenthesis indicate a short silence
- **[ ]** Square brackets indicating beginning and the end of overlapping speech.

A Group Discussion Starts

M: The time now is xx:xx and this is a focus group discussion with a group of 7 ladies at xxx (facility name), the date today is the xxxx (interview date) , it’s the beginning of the discussion.

M2: Before we start, we are going to ask you to introduce yourself but please do not mention your name, you can say my name is 006, your number and where you are from.

P007: Okay, my name is 007, I am from XXX [Name of the location].

P005: My name is 005; I am from XXX [Name of the location].

P006: My name is 006, I am from XXX[Name of the location].

P003: May name is 003, I stay in XXX[Name of the location]

M: Is XXX [Name of the location] one place, is there anything like Soweto, there is XXX[Name of the location] .

P003: No.

M: There are no extensions.

Ps: There are but not like in XXX[Name of the location]

M: Are you all coming from the same extension as you are here?

Ps: No.

P: xxxx [Name of the location]

M2: Okay, you are saying xxxx [name of the location] ?

P003: xxxxx [Name of the location]

P: [Name of the location]

P003: xxxx [Name of the location]

P002: My name is 002; I stay in XXX [Name of the location]

P001: My name is 001; I stay in XXX [Name of the location]

P004: My name is 004, xxxx [Name of the location].

M2: Okay, language, preferred language?

P: Zulu.

P: Zulu.

M: Zulu.

P: Why all of you ((speak)) isiZulu?

M2: As we were recruiting there by the clinic, it means most of you who agreed speak isiZulu. What language do you speak?

P: I am a xxxx (ethnic group).

M: Okay.

P: But at school I studied xxx (language).

M: Really? Okay, I understand.

P: But I can speak isiZulu.

M: Oh you are also able to speak the language?

P: And to write it.

M2: When you visit the clinic, do the sisters ask if you have lost weight or not?

P006: They do ask.

P007: They do not ask me.

P: They have never asked me also.

P: No, I have never been asked also.

M: How many of you have been asked?

P004: They used to ask me.

P003: They have never asked me.

P002: They never asked me.

P001: They have never asked me.

M: So the one who has been asked is 6?

P006: Yes.

M: And 4.

M2: So, two have been asked and 5 have not been asked. So those who have been asked, why do you think they ask you if you have lost weight?

P: The thing is they check here on your file to see how much you weighed and then if you lost they will tell you that you lost this time, they will tell you.

M2: Why do you think they ask you?

P003: The thing is before I weighed less maybe if I gain maybe ((not clear: 04:38)), ja.

M: Okay even though some of us have not been asked, why do you think they ask, why do they measure this weight and tell you if you have lost or not, why is this weight measured every time you come to the clinic?

001: I think that they want to see how the treatment is on you.

P: If you are taking them accordingly.

M: Okay, what do others say?

04: One said you do gain weight, which allows other diseases like high blood to attack you, so I need to monitor it.

M: Oh they monitor health?

04: Yes.

M: Okay, yes number 2?

P002: I was going to say the same thing.

M: Yes number 3?

P003: I am thinking again that as number 4 said that as you continue gaining weight you can acquire high blood, same thing applies if you lose a lot of weight, that is when other diseases like TB attach you, I think they are trying to check and help you keep your weight at its best.

P: For instance, if bloods were taken from me and I do not get my results since they say my blood clots, they then come back and take blood since they want to check diabetes, they find out that I don’t have all these things but they never explained anything to me, I just attend and go in and out.

M: They do not explain.

P: They do not know what they are going to find also.

M: Is it possible that all of us here have started receiving ARVs?

Ps: Yes.

P007: No.

M: How many have started?

Ps: 6.

M: Okay, it’s number 7 only who has not started, do you attend initiation classes? (.) You have not started, and you are not attending the initiation classes for ARVs either?

P007: They are only giving me the results; I had come to collect results actually today for my CD4 count so it’s still high so I am still getting Vitamin supplements only.

P001: You must keep it that way. ((Ps laugh)). Keep it like that until forever.

M: Okay they are encouraging you, this morning as you were coming to the clinic here maybe you were in a taxi or you were driving your own cars coming this side, what was going on in your minds, how do you usually feel when you come to these clinics to collect your ARVs?

P003: Initially, we used to be shy but not anymore, we are free now as you can see, we are just talking. I was shy at first because I am old, kids will just be shocked, but I used to say if they want a reason I will explain, I am not shy because you will expect both bad and good things in life.

M: Initially, you are saying you used to be shy.

P003: I used to be with young people, people used to say this grandmother, you see those kinds of things, but they never laughed at me, they never judged me they knew that it was life, many things happen in life.

M: Okay, is there someone who wanted to say something?

P001: I was never shy because of the manner in which I was sick, I needed to start taking them because previously in order for us to start, because I started taking ARVs in xxxx (year), you used to take long before you take ARVs, you used to find that in one week, you come once, I was just wishing that I was taking them, seeing those that were before me were well, so I just used to enjoy coming to the clinic hoping that my days to start taking them were nearer.

M: Okay, you were looking forward to it.

P003: So just to repeat, another thing that used to make me shy, when I was 7 months in my new job as I am working even though I am old, 7 months into that job, I met this…, I went through rape anyway, I attended for my treatment at the hospital until I finished in 6 months, I kept getting negative results, they said I am done, I attended counselling, everything, I was sick on the inside not on the outside since even my employers supported me, they did not fire me, I only worked for 3 weeks into the job, they did not fire me so when I went back they accepted me, I was attending a clinic in xxxx (name of location) . In xxxx (name of location) they kept saying my blood clots, it’s missing, I used to say I do not understand what is happening, they will keep saying that it clots and I used to say I don’t understand what is happening, I didn’t care because if you do not want bad things to happen to you, who do you want them to happen to, you see that type of a thing, when I finished that six months, they said I am okay, I am negative. So, when I started the following year they said I must come back after 6 months so that they can be certain that I am negative, when I got there, immediately after taking my blood at xxxx (area name), they said I am positive.

M: Okay let’s continue then, we are trying to find out how you were feeling when you were coming this side today?

P007: Since I started attending here in January to be quite honest I have never been shy, it’s things that I want, yes, my blood is positive but my mind is not positive so when I come here, even when people are looking at me, you find that you enter, you know when people are seated there, you know at the back of my mind I always tell myself that at least I know that you do not know yours ((status)) so I am not shy to be quite honest, I just don’t have them at all this is my life.

M: Is there someone else who wants to say something?

P001: No, I have never had a problem.

M: Just how do you feel in the morning when you are in a taxi or in your cars on your way here?

P005: I walk by foot; today actually, I was walking all alone. Normally, I walk with my friend, they know us here at the clinic, we are always talking, there are those whom we have made to accept, we have come here for our health, to be just healthy for our children or whoever it is that we live for at home, others sometimes say, you are lying, you do not have this disease, you have come to laugh at us.

P007: It’s true.

P005: I will then say to them if it’s ((the virus)) already there, there is nothing that we can do.

M: So, you do not have a problem, yes number 2?

P002: What I can say is, I informed my friends, the thing is I am not shy because the time I found out that I am positive, I found out in xxxx (year) then I started here at a support group for three years, then this support group for three years, we used to go out and meet people from XXX [Name of a location], you name it, we will then go to XXX [ Name of the location] because their office was in XXX[ Area], we used to go there with t-shirts on, it means we were just showing everyone that you know what even if you find out that you are positive or what, you need to appreciate because here we are, living with this disease and another thing that I liked there was this TV programme, I forget its name, what do they call it, I forget its name, they used to teach us about HIV positive, how to…, how to look after yourself, that you should not change your life and be normal as a person who is living a normal life]

M: [So you have never had problems.

Ps: No.

M: You are happy, and you look forward to it, when you were there seated at the waiting area, waiting for the nurses and the doctors to attend to you, how do you feel? What goes on in your minds at that time?

P007: The only problem that I have while I am seated there waiting for a doctor or a nurse with other patients, is this issue of the files, that our files have that thing, why are they different from other patients’ files, that is the only thing that I am not happy about.

P002: “cause from now people know]

P007: [Our files are not the same as those of other people that are sick, but other people know that these ones.

P003: And they start gossiping.

((All participants speak at once at this segment)). It’s a docket like this.

P004: At first, we were asked to sit all by ourselves this side, we complained about that saying they are separating us because you used to know that if I pass the dentist, I go straight there, everyone knew that my goodness, she is going to collect the ARVs so they change, they brought all of us together but still, it still the same because everyone knows that I am sick because I am carrying this type of a file, this one who is carrying this type of a file is positive.

M: How does the file look like, is it different because of its colour or the size?

P002: It has the number and they write a date by hand this side, if you try to turn it around trying to hide it, they wrote that on the 21^st^ you came, and ours can be seen that from the 21^st^ and whatever date, if you turn it around, your name, age and everything is written in big letters but for diabetic patients, it’s not outside, they put theirs on the inside.

P004: And others are yellow (in colour) isn’t it?

M: Okay, okay when you are standing on the scale, how do you feel?

P004: You wish that you gained today, that they will not tell you that you lost or that you have become fat.

M: You wish that you gained weight.

P004: That it is okay so that you can be happy.

M: How do you feel?

P005: I can say that I don’t feel okay because there are things that I also do on my part, that of not eating accordingly. You see that type of a thing, I can stay without eating for a whole day but I cannot go to bed without eating since I need to take the medication, you cannot take them without food. So I cannot say it’s treatment or whatever.

M: By way, why do you not eat number 5?

P005: I just do not know.

P006: You are just like me; I used to think that it’s the medication perhaps.

P005: You see, they even say that I lost weight, the doctor told me today, others do not care, he was asked why is am I continuously losing weight, I am just not gaining, I just kept quiet and did not respond.

P001: They do not tell me if it’s right or wrong, they keep quiet, they ask you to stand on the scale, they will ask ((questions)), what does that read, you tell them, they say okay and the record it (they all speak at once).

M: So they do not tell you what you weigh on that visit?

Ps: Uhm, uhm ((meaning no)).

P004: You are lucky if you find the one who will tell you, it depends on the person who is assisting you, yes, it depends on people.

P005: Because for me, there is this one who asks, what was happening my friend, you see that type of a thing, the thing is others do not care; I will just assist you and leave it there.

P003: You see today, I was weighed twice, they said I must weigh myself, I weighed myself and no one wrote it down for me, I went outside, when I was outside they asked did you weigh yourself grandma, I said I did, they said we cannot write something that we do not know, go and do it again. I was weighed twice today.

M: But you still do not know?

P003: I know but they cannot write that which I tell them, they need to see it; I had to go back and weight myself again so that they can see ((so that they will see)).

P007: They have never weighed me. I weigh myself at home, but I have realised that I gained weight, they are not even asking.

M: We were still talking about the moment when we are on the scale weighing ourselves how do we feel. We are trying to find out what goes on in our minds when we are weighing ourselves on the scale?

P004: Here on the scale, you just stand, the main thing that you want, is to know what the weight says.

P001: They will tell you if you have too many clothes that you need to take off, you go there because you are there to write the time during which you got there ((they all speak at once)).

M: Can I request for one thing guys neh, I am asking for one thing, if we talk right, can I request that one person speaks at a time. So that our recorder will be able to capture that so the transcribe will also be able to hear what we were saying. So, I am trying to find out when we are standing on the scale, how do we feel, she explained that she does it at home.

P007: Yes.

P004: When I am standing there, I am in a hurry to know if I lost or gained ((weight)).

M: So you are anxious. Uhum what do others say?

P007: Indeed, I am thinking that when everyone has to stand on a scale, they want one thing and that is to see that their weight went up and when you are there, you get afraid and say oh my gosh, I hope I did not lose weight.

M: So can it be that we all do not want to lose weight?

Ps: No.

P: No one wants to.

P007: No, no one want to lose weight, it should just be moderate.

P003: It should be moderate because when you lose, you get stressed out, you are not able to sleep, you are not a able to eat, you are not able to do anything and then you are afraid asking yourself what is wrong, what happened, what did I do.

M: All right and then some of us we have said already that we get here and weigh ourselves, now the mother is going back home, she is from collecting ARVs at the clinic, what goes on in our minds, what are we thinking, for instance number 5 said, she found out that she lost weight today when she weighed herself. How do we feel for instance if we lost weight?

P007: When I get home, I will eat; I will sleep and rest, and nurse myself so that I can gain weight again.

M: Yes, so we are thinking is what is it that we can do to gain weight, yes number 5?

P005: I sleep late; I can go to bed even at 2 and then wake up at 2 in the afternoon, when I am craving for a cigarette because I smoke.

M: You smoke okay, so now that you have found out that you lost weight most of the time if you ever stand on the scale and find out that you have lost weight, how do you feel on your way back home other than thinking about what you will need to do to pick up on weight? Yes number 2?

P002: The thing is, you just become stressed out, you just cannot concentrate on the things that you are doing at home, do you understand, you become short-tempered, you ask yourself what is going on, what did I do, what mistake did I make in order for me to lose weight in this manner sometimes you ask yourself that oh perhaps it’s these things that I was doing last week, perhaps you did not eat the whole day or I did not eat today or whatever, you see those kind of things.

P: Stress also makes you lose it ((weight)).

P002: If you receive bad results then you get more stressed out and you lose a lot of weight.

M: So it stresses you out that you weighed less?

Ps: Yes.

P005: A lot.

M: Okay, yes number 6?

P006: Since you sort of lost weight when you started to get sick, you then started these ARVs and it was as if you are picking up on weight as time passed by you lose again, you don’t want to go back to that thing because you know that you don’t want to go back to that thing ((weight)) since you know how people look at you if you ever lost weight, you see that type of a thing.

M: Uhm, okay, let us take one response on how it feels if you have lost, and one then proceed to if you gained.

P002: On this thing of weight sometimes you gain and sometimes you lose, I asked the nurse who said sometimes ARVs can cause that, do you understand that sometimes you gain weight, sometimes you lose weight that’s why sometimes you find that other people’s shape change, they will have a big breast, you will have a big stomach, you were develop calf muscles, do you understand those type of things, you will develop veins that are just funny, you will not understand, do you understand me but nurses tell us all the time that if you have a problem maybe you see the changes in your body you have to come to us and ask.

M: So at least you understand that the ARVs on their own sometimes may do that. Okay, if you gained weight, how do you feel on your way back?

P001: You feel good when you gain but what you do not like is that sometimes you can gain on the upper part and lose weight on the lower part. So that is what makes us sad, when I started the medication I had a good body shape, I was okay, you know what when I started the medication, I was okay, my whole body became full, after that I started lifting the irons ((gaining weight on the upper part)), I gained here on the upper part, my breast became big, I started having breasts, I developed thin legs and veins.

M: Which veins? Those on the legs or?

P001: Here on the legs, the veins that appear, that look constricted as if you are an athlete, things that make you look as if you are lifting ((weights)), as you progress it’s as if you are lifting something.

P002: You become extra fit.

P001: Not just being extra fit ((they all laugh) a flat bum, yoh my dear, you will never regain your bums, you never get them back ((Ps agree with one another)).

P003: Even if they change the regime.

P002: You do not get them back.

M: Okay, okay, okay so what are we saying then, we are talking about gaining weight, how we feel on our way home, number 1 says gaining weight is good depending on where you gained weight, it’s painful when you gain on the areas where you do not wish to gain it, what do you say number 4?

P004: For me gaining weight is good but the problem is that my body gains weight in a manner that I do not like, yes, I see myself gaining more for instance my breast, I do not have a big breast by nature and I do not have a tummy but these days I feel as if I have a tummy so I feel bad and get stressed out if I need to get dressed and I see myself as looking like another grown up lady.

M: Okay, all right so you do gain but feel that you gain on undesirable areas. What do others say, number 5?

P005: Since I started taking ARVs my weight has never reached 60, it’s always around 51, 2, 3, 1 something like that, it keeps going down, it has never reached 60, it will then go back to 40, it goes down again, you can see as I explained that I cannot say it’s ARVs, that are doing this and that to me. Isn’t it there, there are things that I am doing wrong.

M: I understand.

P007: I am not fat ever since I have been growing up so for now the tablets that I was taking ((Vitamin supplements)) I think there were also those of TB but I finished those for TB, isn’t it the procedure if they find out that you are HIV positive the procedure is that you take the tablets to prevent TB.

M: Oh the NIH?

P007: Uhm so I don’t know what it is that makes me to gain because everywhere people say you have gained and I can also see that gained and as number 4 said, I think I will develop a big stomach ((Ps laugh)).

M: But you are not yet taking ARVs isn’t it?

P007: No, I am still on Vitamin supplements.

M: So most of the time when we gain weight we are happy but it depends on where we gained. All right if they ask a person who is living with the HIV virus about weight loss at the clinic, what could make them say they have lost weight? For instance, when I am at the clinic, they ask me did you lose weight and I say yes, I lost weight, what will make me say I lost weight?

P001: It happens maybe there is a problem that I have at home, maybe you have children, children have that thing of having things that will make you to lose weight when you keep talking to ((reprimanding)) them sometimes you will find that you are looking for a job and you are not getting one, you need to take this medication, how are you going to take this medication when you did not have a meal since these tablets can make you hungry, you are unemployed and you are not getting the pension sometimes you will find that you will receive them maybe 3 months, maybe 3 months after starting them your CD 4 count goes up and then they have given you a letter to go and see the doctor who will prepare pension for you, when you get there the doctor says your CD4 count is up, I will no longer issue pension for you, so I think it’s those things.

M: But when we come to the clinic and say I lost weight, what is it that we have seen that actually tells us that we indeed, did lose weight?

P005: I see it with the clothes that hang on the body, you know that this jean that I have on fits me tightly but now it no longer fits me tightly, it’s hanging, it means I lost.

M: Okay, yes number 3?

P003: Number 3 agrees with number, the wardrobe shows, it will no longer fit me in the same manner that it fit me before, but I am okay now.

M: Other than clothes, what else shows us that we lost weight?

P003: You also become weak, yes, you would feel even when you are used to doing things yourself, you find yourself not being able. You will then realise that you are weak.

M: So it’s feeling weak, the clothes, what else show us?

P002: You are lazy now.

P003: Even if you used to walk fast, you are no longer able now; you walk slowly since you are weak.

M: The lady once mentioned stress, that she sometimes has a lot to handle pertaining children, what else makes us to see that actually, we have lost weight? Or what is it that can make us to report at the clinic and say that we have lost weight?

P001: Not sleeping well.

M: What makes us to not sleep well?

P001: It’s thinking a lot.

M: Not sleeping enough, okay, all right and then if we have started taking ARVs for instance, what does losing weight when we are already taking ARVs mean to us? What do we usually think is actually happening to us?

P007: You must not forget that I have not yet started taking ARVs, but I have friends that are there, I have heard stories that sometimes they affect maybe you will gain or lose a lot of weight or there is just something that is going to happen. So yes, the ARVs sometimes they affect our weight a lot. Sometimes you do not eat well, sometimes you do not eat a lot.

P002: Agrees with number 7 as much as I know about ARVs since I am a person who is taking them, then there are side effects when we are taking ARVs, yes, sometimes you will find that you do not sleep accordingly sometimes you suffer from veins and you just experience nightmares that are horrible, do you understand me and then those are other things, the head aches and all those things, others experience a hearing impairment, become blind, others will tell you that I no longer see clearly, yes, things like those, another one will tell you that my nails change, my hair is falling and then the colour of my skin is changing. Those are the side effects.

M: We are trying to find out what losing weight after we have started taking ARVs that is what I am trying to find out?

P001: It is painful because you think about where you are from, that you once lost weight anyway and here you are, if you regained weight and when you lose again, you become sad that eish I wonder what is happening now why is this happening, you then end up not understanding what is happening.

P003: when I first started taking ARVs I was not eating, I just hated everything, I do not know why, when I look at something my heart just did not long for anything, I was contemplating to stop taking them, I used to ask if there are no other tablets that I could take, they used to say grandma you will take those as they are. On TV when they showed anything that looked like tablets, I used to vomit, I did not eat for 6 months but I did not develop anything else, I persevered until I was okay. What they told me is, I preferred taking them in the morning, they said I should change and take them at night so that I will stop feeling irritated, I take them at night now, but I used to be not able to eat for 6 months.

M: Otherwise just losing weight whilst you were on ARVs, what does that mean to us?

P006: Up until today, you will never know what causes it because when you ask the nurse, she says.., she says come and ask me if there is some changes I will help you, but you keep continuing until you get used to it over a period of 5 years, you get used to that situation, have you seen that. You don’t know because even the nurse themselves do not explain what actually causes it.

M: Number 2, what does it mean for you to lose weight whilst on ARVs?

P002: For me sometimes I get a feeling that maybe these ARVs do not get along with my system and then have another feeling that hey I will stop taking them since I do not see its results, I keep losing weight, that is why most of the time you find that after people have started ARVs.., you find that a person does not take them anymore and they will say, if I don’t take them I regain my weight, do you understand but if I keep taking them, I see that my shape actually my weight change, I will gain today and tomorrow, those are the things that frustrate us especially women since we do not want to become fat, you don’t want that, you just want to be fit, and just look good in your clothes since what is important for us is the weight, yes.

M: So you are saying most of the time, in your mind it’s as if the ARVs are not working well?

P002: They are not working well ((effective)).

M: Is there perhaps stigma attached to us losing weight when we are already living with this HIV virus?

P007: Yes, there is a lot, if you lose weight, you feel as if everyone can see, everyone is saying, she is positive. It’s like as number 2 said that we have a problem because we are women we want to maintain our good body weight, if you lose a lot it’s like everybody, you know another thing that causes us to say that the ARVs are not working because you will have stress and everybody will be like they can see that I am positive, why did I lose so much weight and then when you gain a lot of weight, there is thing that says ARVs are damaging her.

P001: Yes, they used to say that.

P: Uhum.

P007: They are saying that so we are no longer certain of what is right because the only disease that exist amongst people now is HIV, I do not think that HIV is the only disease that make people to lose weight, many diseases make people to lose weight but people will only tell you about HIV, actually this one has HIV, she has lost so much weight, she has even become darker in complexion, actually, she is suffering from this disease now, when you are sick now, it’s HIV.

P004: You will find that a person lost weight, it’s diabetes, she has it.

P001: They have names, it’s OMO, it’s a high heel ((Qhoks)) others will tell you that he was shocked by the CTM wires.

M: What do they say ((Ps laugh))?

P002: They say she has been shocked by the electricity wires, Eskom and then they call it different names, Z3.

P001: Others say it’s a high heel ((iqhoks)).

M: When they say it’s a high heel what do they mean? A qhoks is a high heel?

P001: Isn’t it you are thin, you are thin at the bottom.

P002: Yes, you are big at the top.

M: O::h, I used to not understand ‘iqhoks’, why people called AIDS a high heel?

P001: Yes.

P002: If you gain weight they will tell you that it’s ARVs, you see, if you lose weight, no, she is suffering from the Z3 my friend.

P004: She has AIDS now, it’s no longer HIV, it’s the same, there is no difference to them. This thing stresses us a lot as women.

P: If you lose they say actually this one has defaulted, it means they are no longer collecting the tablets.

M: There are doctors out there ((They all speak at once)).

P002: They say they are not going to the clinic; they are not sick and forget that you go to the clinic to find out what you are suffering from. For instance, I was never sick, for me to find out that I have HIV, even though I was losing weight, I tested for my CD4 count which was 117 I had to start ARVs, I was not sick, I was just a person who liked testing herself up until I found out even when I find out I was shocked and said how come but I told myself that life is the way it is.

M: Okay, all right, we are looking at stigma, if people we stay within the community now that we have started taking ARVs, anyway we gained our weight back, we know what is happening, we are careful about life, we are cautious since we already know what is happening perhaps the people that we live within the community, do they ever say things relating to stigma other than the Z3s and the high heels that you mentioned, are there other things that you see them doing to say, this is stigma that makes them to behave like that to a person who is losing weight and who is highly noticeable?

P007: You know, I have realised that the community that we stay at, there are so judgemental you see if they see you losing weight, I will be judged in the manner in which I lived my life, we have seen her anyway…, she has been promiscuous, she has always been this, she has always been that, you know to be quite honest ladies, I found out that I am HIV positive in xxx (month) this year .I just came to the clinic to test and then I haven’t told anyone except my boyfriend because I feel that hhah I don’t want drama, the people who will ask me that…, they will judge me, and that is why we bottle up everything because of it’s not that we don’t want to talk, we do want to talk but maybe we need things like these, we are not able to discuss them because of the stigma, we will be judged.

M: Okay so we are saying there is stigma and that it’s rife out there?

Ps: A lot.

P005: Even your family still does that thing, they say hey don’t stand too close to me you will infect me with this thing. I just say it’s your ignorance, I wish you knew about that thing. Who said I am HIV ((positive))? ‘cause I did not tell you that, you should have asked me, you ask your sister to accompany you and to be your treatment supporter. For me, my sister should have been my treatment supporter but instead it’ my cousin that supports me, my sister that I come after said yoh you want them to say I also have this thing. Imagine your blood sister doing that to you, what is an outsider going to do. That is why I have told myself that, I have a friend that I usually attend with here, today she is not here I do not know how, she is just a person that I am with all the time.

P006: In the community, there is a lot of stigma sometimes it depends upon how you ignore it, as a woman, there are other women that we came here ((area name)), we bought our houses and came with our children, when you keep losing weight until you are very thin, they will say but your children have big bodies but you have just become a tiny thing, what happened. I have children with big bodies; you see that type of a thing now it’s what makes you to keep locking yourself inside the house, you see and not visiting them.

M: So stigma sometimes appears in a form of people speaking in riddles.

Ps: Uhum.

P006: They gossip about us behind our backs, not even speaking in riddles. You see that thing.

M: Okay, otherwise a person who has started taking ARVs in general, what do you think that person’s weight in general should be?

P001: I was thinking that since you have already started taking the tablets, you should be okay, be well so that others can see that ARVs help since you will find that another one will say that actually let me just die, because you find that you are always complaining about this, you are complaining about that, you are complaining about your legs, you are complaining about your head, you are complaining about the breast, one will say, I know this was not your body shape, you have changed now that you have started taking ARVs. ARVs once they are in our blood, we were supposed to look the same as before, regain our strength, go back to normal and be okay, we shouldn’t be sick but as time goes on, you go back to the diseases that you once suffered from, a head ache that you used to have before you started ARVs, when you are in the middle of using ARVs that head ache comes back.

M: Okay, all right so we are saying that a person’s weight ideally should go back to its normal place. Is there someone with different views than those shared by number 1, there were many hands up, people wanted to talk.

P003: She said it in a manner that I would have said it.

M: Otherwise, what about the shape, how do we expect a person who is on ARVs to be regarding shape?

P003: Her normal way.

P005: Isn’t it when you started ARVs they said it has side effects, they said that if something happens to you, you need to report but even if you report you will not regain your normal shape, you will be busy with your big stomach and those breasts since you will not go back to the way you were especially for men, you see those type of things so what’s the use, they ask you to change the tablets but they will not take you back to where you were before.

Ps: But it’s just the same.

P005: it has caused damage already; imagine being small here, big on the upper part, I was never like that before, you see that type of a thing.

P001: What I did is, I tear off all my photos before I started taking ARVs because when you look at me and when you look at the photos, you can see that no, is this your twin?

M: It’s very different?

P001: It’s different, you see I had straight legs, I had a good body shape, I had those legs but once I started using ARVs hhah even my kids said no, mum.

P004: You do not buy the boots because it’s too lost here, you just do not buy boots, imagine you were light in complexion but you have become too dark, that is why they will say you have defaulted even if you did not.

M: I am going to ask you to take one and pass, these are our photos that we have brought so that we can facilitate this discussion, which changes can happen to a weight of a person who is taking ARVs, we have discussed that we would expect that a person will regain their body and all that so if we could perhaps show them to one another, so that we can see who has what. Okay, here are our photos then, which weight would we say is ideal here.

P: It’s this one underneath ((they all speak at once)).

M: Okay, okay, number 6, wants number 7, so you choose number 7, as a shape that you like.

Ps: Uhum.

M: Is there another one which you chose which you said you like. Which one is that?

Ps: That of number 5.

M: Please check the back for me, what is written?

P: It’s 6.

M: It’s number 6, how many like number 6, 3 people like number 6. How many like number 7?

P007: I also like her.

P002: Number 7 is okay.

M: In other words, this means number 7 was chosen by 4 people. Okay, that’s the total. Okay, let’s start with these ones for number 7, why did you choose number 7, why do you say number 7 ha an ideal body shape and body weight?

P005: You see it’s not that big. It’s not too small, it’s okay. It’s moderate.

M: Okay, moderate weight.

P007: I will say all her body parts are proportional.

M: Okay, body parts are proportional. Okay, what do others say?

P006: She is not big on her upper part as if she is a body builder.

M: Her shoulders are not big.

Ps: Yes.

M: Let us talk about number 6 now.

P003: Number 6 is right does not have a big stomach, does not have big shoulders, has a good body shape.

Her shoulders are good, the stomach is flat, and her thighs are good.

M: I am laughing because even those that chose number 7 are now commending number 6 ((Ps laugh)).

P: We are not commending her; we are checking it out.

M: And then the ideal body weight, what kind of a weight is it that we say that one is good?

P: It’s 34 for me. For me, it’s size 34.

M: Okay, what do others say?

P007: 34 or 36 for me.

M: What is it that makes us to say that XXX’s weight is good? (.) What makes us to say that a person has a good weight?

P001: If they are dressed up they look good, that is first.

P004: It a person who is forever young.

P002: A person who is able to put on a high heel, they are able to put it on and it it’s not heavy on her. They are not just dragging their feet, yes, you check there if a person has a good weight.

M: All right, the good shape, when do we say that person has a good body shape?

P004: That is when we check the breast, the big tummy, the hips, the bums, and the legs.

M: Okay what do you look for?

P002: A person is actually attractive with a body which is proportional, not with big knees, the thighs, the legs are also proportional, do you understand me, that is why when you check the phone, they make jokes out of us, as women, they say check her out she has unnecessary fats on the waist area, you know it would have been better if I found someone like XXX [Name of a Celebrity ], you see. Isn’t it[Name of a Celebrity ], actually small, she has a small waist area, she has breast that is proportional with her body, her bums are okay, do you understand me so we cannot afford since we are not working, we cannot be able to eat this type of food where they say you need to eat in that manner, So the xxxx [Name of a celebrity] afford and they go to the gym, they eat these powders, what do they call them, these that they eat with milk.

P: The shake.

P002: If they say here is a Herbex, I also run to it, when I drink it, it makes me worse and I swell.

M: Okay, we mentioned bums, we mentioned hips, in men then, what shape do we say in men is good for men?

P007: In men, a big stomach is an absolute no.

P002: Bums are also an absolute no.

M: So you are also expecting men to have a flat tummy.

P007: Yes, a 6 pack.

P002: But when they are old, they cannot always have a 6 pack.

P007: Never mind but they should exercise like white people, the people whom you see jogging on the street, whites are on the bicycles sometimes, they become fit and okay.

M: What do you say about bums?

P007: They should not have bums, they should not have a flat bum, they should be proportional with the body.

P004: They should be good and look good. Otherwise I will invite him over when I am inside the house or when it’s dark ((they laugh)). Isn’t it they say that, they say we are people for the night. Hey you my mother will be at home during the day, she will sleep at 10, she is scaring you away isn’t it.

P007: You are able to walk with him during the day but he cannot walk with you during the day.

P002: It’s painful man.

P007: It’s painful.

M: This is very interesting and what about the body weight for the males, do we expect that women’s body weight…, I think you talked about size 34, the 34 that you mentioned, did you mention it irrespective of the height of a person, regardless of how old the person is, is it that you just want a person to be size 34?

Ps: No, no.

M: What were you referring to?

P007: 34, 36 we were mentioning it for old people, anyone I think who is above 50 we are not expecting that person to wear size 34, even the body shows that that person is old, we are expecting that the size will be big. For me with regard to the males’ sizes, size 34 for a guy no, it’s too small.

P002: Actually, the males’ sizes are from 1, 2 it means 4, 5, 6 once you reach 20 something it’s the kids’ sizes. If it’s an adult it starts from 1, 2, 3, the waist area, so size 34 is for women, it means I can fit in on size 34 since I am this weight?

P007: No.

P002: Even if it were to fit me, there will be a need for a size 34 women’s size, isn’t it. When I see you on size 34 just because we are of the same weight, I cannot then want to put something that you have on.

M: Okay.

P002: For me there is a need for a size 34 that fits me because they will see that]

M: [Okay, these things that we are mentioning, where do we get them from, can it be that we get them from the community that we stay at or perhaps before we get there, amongst these pictures that we have, which ones are attractive, let’s show them to one another again?

P002: Okay, pictures that are attractive here are 1, 2, 3

P003: I see that one.

P002: It’s that 32 that you are referring to.

P003: Actually no, that one is 28.

M: Which number is that one?

P007: It’s number 1.

M: You are saying number 1 is too small. Okay, I am looking for the one that is attractive right now. The one which you could say it’s attractive, people will be attracted to that weight? Which one do you choose?

P007: It’s number 6.

M: It’s number 6 again?

Ps: Uhum.

M: Okay.

P002: It’s number 5 and 6.

M: Let’s hear about number 5, why do we say number 5 is attractive?

P004: We are saying number 5 is attractive because when you take ARVs you need to go there.

M: You are choosing 5 now.

P004: Actually, I thought it’s number 6.

M: Who chose number 6?

P004: We almost all chose her.

M: There are those who chose 5 and 6, they said 5 and 6 is attractive, okay what would make a person say number 5 is attractive.

P005: In all respects.

P002: It is okay.

P007: It’s a normal person.

M: What about 6, why do we say 6 is attractive?

P002: 6 is not fat, is not thin, the lady is just of moderate weight.

P007: Has some flesh.

P004: Is of moderate weight.

M: Who else other than us whom we chose…, who else do we think will be attracted to number 6 other than us?

P002: There is no one else ((Ps laugh)).

M: For instance, if perhaps you are going to think of people that you are close to maybe your partners, maybe your family members who will look at that thing and say that thing is likeable, that thing is attractive?

P007: *Eish* I think my partner will like it.

M: Your partner will be attracted to number 6. Okay, what do others say?

P006: I like th:at one, my family will be comfortable in it, even my neighbours will like this one.

M: That is number 7.

P006: Yes.

P001: My kids will be happy if they can see me look like this one, they can say, you are okay now.

M: Okay, all right I was saying, let’s now continue and look at these things that we are mentioning, where we get them from, can it happen that as we chose them so eloquently, where did we learn to choose these people, where did we learn to choose these people? Can it be that it’s teachings from the community, teachings from our families or teachings from health services or teachings from the media? (.) Where did we get these things that we are mentioning?

P001: Even in the community they are there since when your weight changes people talk, there is that which they discuss, you will hear them say she has a squint shape, she is not shaped well, she is this and that, she is thin, she has HIV now, that is what will make you wish you were of moderate weight, not big, not thin, and just be of moderate weight, that is the manner in which people talk.

M: So number 1 says it’s the community and what about culture, do you think culture played a role in making us chose the people that we chose?

P007: Yes.

M: How?

P007: When we were growing up, we know that if you are going to get married there will always tell you that you must not bring us a thin bride, a shapeless one, we want a fit girl, with legs, they normally say that.

M: Okay, what do others say?

P002: It’s just something that exists amongst us, it’s our culture as Africans, we like to judge and forget what is inside these people’s hearts, we will point at them and say, she is fat, she’s short, she is tall, she dark, our son has chosen that one, leave him alone, that is where he is happy, he has made his heart happy. We cannot just interfere and judge, that will never help us.

M: Okay, what about the family? The family has an influence in us saying what we just said…, it’s one of the things that we heard at home when we were having conversations or family teachings.

P004: The family, most of the time does not like a bride that is thin, yes, if you go to get married, you left home fit isn’t it, you have gone to get married when you get there you lose weight, they will say he is not providing for you, you will now end up wanting to maintain this weight since you are afraid of what people will say.

M: Okay, all right and what about health services, where we get assistance for health, like doctors, nurses at the clinics and hospitals, is it possible that there is something that we mentioned which is in line with the teachings that we get there?

P007: Yes, the health services, they normally tell us that it clashes because they will tell you that you should look after weight and protect yourself from heart attack, you will have a stroke, you will get high blood pressure because of how we eat.

M: ((A cell phone rings)) I am going to ask that we put our phones on a silent mode but respond to them. If we receive a call, we can go outside and respond to it from there. It’s not a problem. You are saying they warn us against it and say we should not gain too much because we will get diseases, you also mention that we should eat good food.

P007: Yes, that is why I said when you take ARVs it’s like they confuse you because if you got to the doctor, he says hheyi you must look after your weight, don’t gain too much, blah blah blah but when I am alone seated at home because of the stigma out there, I can say this doctor is crazy if he says I must lose weight, I need to gain weight because people out there will talk about me so you see that us as women, we are like in a very tight situation here since we are confused, we do not know who to satisfy in this instance.

M: And what about the media, what do we get, what do the teachings of the media say, that which has had an impact on what we said now. Which role has been played by the media like your TV, your newspapers, your radio to give us an idea that a right weight, a right body shape is this and that.

P003: No, there isn’t ((they all deny it)).

M: Isn’t it you talked about XXX [celebrity name], where do you see xxx [celebrity name]?

P002: On TV.

M: You see.

P003: There are many things that appear on TV, okay, it depends on what you chose, others are right and others are wrong, you cannot concentrate on those things since some of them do not help.

P004: I used to listen to the show called, Siyayinqoba: Beat It, they sometimes discuss those things, what we should do, if you have time to watch you can gain a lot of knowledge.

M: Did you want to say something?

P002: No, I am covered.

M: Sure, okay, what do you think can make people who are living with the virus lose weight?

P002: It’s stress.

P004: And not feeling well.

P005: And not taking the treatment well since everyone has a set time to take their treatment, not feeling relaxed.

M: If we say not feeling relaxed, what do we mean?

P002: Enough hours you spend sleeping.

P004: And working in a strenuous manner for instance for others, they work such that they do not have a choice, cold or whatever it is, they have to do washing, we as people who are not well, when it’s cold, you just find that a person is sick, they are coughing even when it’s cold but they are doing the washing, you will find that there is no one helping them, they do not think for them to say if this is the situation, the washing should be done once, you see?

M: Okay, if we say, it’s not being treated well, in what manner?

Ps: Being abused.

M: O:::h.

P006: If you have this disease, you do not need to be angry all the time, you just need to be okay, you need to be happy all the time because it also decreases the CD4 count, you are always angry, fighting and things like that, it’s obvious things like those are related to weight.

M: What else do you think makes us to lose weight after having acquired the virus?

P006: It’s the tablets. You will eat in a manner that you like and gain weight, you do not gain it. Even if you eat in a manner that you were told to eat, you eat, you are always happy, you try but it’s the same, you do not gain it means they ((tablets)) turn against you.

M: Maybe what is it that can make people that are living with HIV to report that they are experiencing weight changes whilst the scale does not show that their condition changed?

P007: Just like number 5 said earlier, you would see that the clothes look big.

M: It means in other words; it happens that we feel that we lost weight]

P007: [But you can also feel it yourself.

M: But once we are standing on the scale, it says you did not lose.

Ps: Uhum.

P003: It means it’s the bones, some people have big bones, others have a heavy flesh, you will find that the bones will keep holding but you can feel that you are weak, there is a shortage of flesh on the outside, it’s one of the things that cause that.

M: Uhum, okay, all right, what do others say? What is it that can make me to go to the clinic and say I lost weight and the nurse say, you did not?

P004: You will see by the clothes, it’s the clothes.

M: What else can make us to go around saying we have gained and for people at the clinic to say you did not

P002: Your shape can tell you.

P003: Even when you are walking you are just heavy, you get tired even when you are walking, you can just feel that my weight is heavy on me, what is it now.

P005: You will then realise that I need to go to the clinic and test for TB, you see those type of things.

M: It’s feeling as if your body is heavy. What were you saying number2?

P002: I was just saying, you can just see the body if you gained weight.

M: But you are saying you gained but the scale indicates otherwise.

P002: But sometimes the scale can do that thing also, where you will find that sometimes you gained weight, but it will tell you that you lost

M: You are saying in some instances it does not work properly.

P002: Yes, when it is not working well.

M: Uhm scale issue, but then in general, what do we think gaining weight means to a person who is living with HIV?

P007: It means that I am healthy.

P002: Yes.

P003: It means I am happy, loving it and well.

P002: It means the virus is in one place, I have recovered, HIV is now in one place, it has locked itself now.

P006: It removes stigma, those who will tell you that you are thin now, you are sick.

M: It takes away the stigma.

P006: Uhm.

P007: And I think as we are living with HIV, it is good to meet the people that you have not seen in while saying yoh you have gained so much, I think it makes you to feel like a va va voom ((They laugh)).

M: What does a va va voom? ((Asks M laughing)).

P007: That oomph of some sort.

P002: That I also know that I am well.

P003: I am also the same as this one, it’s just that I take tablets and she doesn’t.

P004: If a person says that, you will find that a person was small, you see, they appreciate that. You see when a person says you gained weight, I get angry, I do not want to gain. I don’t like it, I like my body, my body weight was not this big, it was moderate, but now I can see that I am fat. I see it with my clothes and people are saying, what’s going on why are you this fat.

P006: I tell them that I am a woman.

P004: A person will speak as if they are suspicious of something.

P007: Why do you lose so much weight? Others say you need to stop taking these ARVs; they make you look like a child.

P004: You see they don’t say good things, they say it in a manner that there is something that they are suspicious of.

M: Okay, perhaps from your experience as people that are living with the HIV virus, which way is better to ask if people are losing or gaining weight?

P007: To be quite honest here, I do not think that there is a better way either way it hurts our feelings. I think we do not want to be asked. Either way, you can ask me if I gained, you can ask me if I lost weight, it hurts me somewhere. It’s like number 4 said, it’s like there is something that you see in me so just don’t ask me.

M: You are a clinic setting right now, you have come to ask for help, in order for you to be helped, and they ask you, did you lose or gain weight?

P006: Her at the clinic, it’s okay not outside.

P004: Because the one on the outside will ask you in a manner that is not good.

P006: Yes.

P004: But if this one asks I will understand it but the one who will ask me here will ask me in a manner that I do not like but if you ask me when I am with them, I will be free when they ask me, who will say actually you seem as if you gained, then it will end there but the one from the outside isn’t she is not going to ask you in that manner, they will exaggerate it, ooh you are thin, you see it’s as if now there is something that they see that you do not see.

P006: One will say, never mind her weight, her children are fat, yoh she is this thin, what is the problem, I am a mother I agree, I gave birth, these children are mine but why do you tell me I am this thin and that my children are fat.

M: Yes, why are you being compared? If someone guys were to ask you, have you lost more than a dress size unintentionally in the last 6 months would you understand what that person is saying? Do you understand what that question means? (.)

P007: I think a little bit but.

M: What does this question say?

P007: If I am right, I think wants to find out if you lost weight intentional or did you lose too much without an intention or something like that. I am not sure.

M: What do others say, is there someone who has a different opinion than this one of number 7? (.) For instance, I brought along some skirts here, so that we can show what we are talking about here, this is size 36, and this is size 38 and that is 34. Here are my 3 skirts, if a nurse or researchers ask you if you lost more than one dress size in the past 6 months unintentionally, she means from which size to which one using these skirts?

P004: You are from here to here.

M: You are from 38 to 36?

P004: Yes.

M: What do others say? Is there someone else with a different opinion?

Ps: No.

P007 &5: From 38 to 34.

M: 5 and 7, and 2 they say from 38 to 34, are there people who hold different views from theirs?

P003: You can move from 36 to 34, I don’t know what causes that but it happens.

M: But we recall that we said ‘more than one dress size’? Isn’t it moving from 38 to 36 that is one dress size but if we ask about more than a dress size, you are from]

P002: [38 to 34.

M: Yes, I wanted to see if we understand each other. Okay what do you think the following people will respond to a person who has the HIV virus who has lost weight? For instance, our spouses when we were sick, when we had lost weight perhaps not knowing what was happening and stuff like that, what did our partners say, how did they react to us as a result of that weight loss that was caused by HIV in our blood?

P005: You are dying; can you see that you are dying?

M: Did a partner say that?

P005: These ones, the family.

M: For now, I am asking about the partner, if perhaps we can concentrate on the spouse or a partner for now?

P001: A spouse sometimes they feel bad, such that they will say, my goodness my partner I can see you are losing weight now, what is happening. So tell your partner as well that I can see my partner, he will then say, why don’t you go and test. Even when they say that you must go and get tested but they themselves are afraid. My husband died refusing to take the tablets, he was afraid, asking me to go alone, I started this journey. He died then; they do feel sorry if they see you lose weight, hhah.

M: So they get sad when they see us lose weight.

Ps: Uhum.

P001: It’s sad.

P005: Isn’t it us as women we acquire these diseases easily, if they go to get themselves tested, they find that they are negative, they can see that no, they are not sick; this is the one who is sick. I am not sick, I am okay, that is when they start spreading it, this is the one who infected me with this disease.

M: So we point fingers at each other, to say you have it not me. What do others say?

P006: They always do that as they say you are losing weight, they do not know that they are there also, they will tell you first because you react faster than him and lose weight, they will then say, go and get tested.

P005: Another one may impregnate women outside of wedlock; they want to hear from this one, the mother of the child obvious, since he knows that when you are pregnant you get tested. I will go for testing, so how did it go at the clinic ((says P mimicking men)) no, they say I do not have this disease, he can just see that he does not have it. If you have it, he can just see that this one is mad, this child is not mine also, you know where you got them from. Men always blame women pertaining this disease.

M: I don’t understand what you are saying number 5? They ask you to go and get tested if you are pregnant; they want you to tell them?

P005: They do not go to test for themselves.

M: If on your return, you tell them that you are positive, what does he say?

P005: If you are positive, he no longer comes to you, it means he can just see that you know where you got this disease.

M: He leaves just like that.

P006: Uhum. I don’t have it.

P001: Isn’t it there is this thing that when you are still dating, you find out that you are positive and that he is not positive ((discordant couples)), things like those happen. So he did not get himself tested, he will tell himself that you are positive and that he is not positive since I heard from the mother of my child that she went got tested and did not find it, maybe it’s still hiding, not because that is the case. It’s hiding, this thing hides, many people suffer as a result of that.

M: So they feel sad, there is some blame sometimes, they hide, disappear and what about children, but before we go there, can it ever happen that our partners call us some names once we find out that we have a virus?

P: Exactly.

M: What names do our partners call us?

P002: They will call you a golf (fast car) man, you are feeding us, can you understand that they call us by those names that we are golfs isn’t it you know that a golf is fast? It’s fast. It means you have been running for very long.

M: When you are still dating.

P005: Maybe we have been dating for 10 years.

P002: And you have never found another person, he will tell me that no, no, you are a golf ((type of a car)).

P007: I have never come across that problem even though he is negative, he is supportive, he does not even want me to say I have this thing, he gave it a name, he says, you have a condition, luckily I have never received those terrible names.

P005: You are lucky, he loves you, I cannot say I know what mine says, I sometimes hear from a friend, it’s a friend of mine where he stays since immediately after finding out I abstained, I did not go back, I have not been dating since xxx(year).

M: Uhm okay, all right so in other words, it’s not your partner who decided, you just decided on your own that you are abstaining now?

P005: Yes.

M: Okay, all right, abstinence and what about children, what do children say when they see us losing a lot of weight even if it’s not our biological children, as we are here, the children of people with the virus that we used to watch losing weight, maybe looking at their reactions and responses?

P007: You see, when coming to children, this is painful because we are adults, we are able to tolerate stigma, to tolerate rejection, to tolerate being judged and all that, it’s not easy for children since as adults we talk and children listen to that which we discuss, when my child plays with your child on the street, you child will tell my child that they cannot listen to anything about your mother who is thin and suffering from AIDS, do you understand what that does to the children, so I think this thing is worse amongst children than us adults.

M: Uhum so they use our ((HIV)) status to swear at them.

P007: Ours, yes, our children are being made to carry our burden.

M: Perhaps as we are here, if maybe amongst us there is someone who has children and someone who has gone through drastic weight loss, to share with us maybe responses from our children, what did our children say?

P005: They are very sad such that they can get sick, you can be strong, but they feel sad, you see. It’s the stress that you are going to die, they are telling themselves that as you keep on losing weight how small are you going to be in a coffin, you will be a child who is 9 years now that you have lost so much weight, you can see that that thing makes her sad, she is not able to eat, this person cannot do anything until you become strong for them, pick her up and make her feel good.

M: Uhm they become sad and say that their parent is going to leave them.

P007: Especially mine are still young, for instance my first born is xxx (age) and the young one is xxx (age). For instance, when I lost weight after my divorce, they were like, you can see that they wanted to carry my load, that they could take away this thing from my shoulders, have you seen a child who from school will say sit down mum today I will cook, I am talking about a child who was xxx (age) at that time, I will cook mum sit down because she can see that you are not strong enough to stand on the stove and cook and prepare their meals, so our children a heavily burdened.

M: So they become strong for us such that they want to relieve us.

P002: They feel that pain, I used to see with mine, mine is xxx (age), my first born, he is a boy, the thing is, I used to have a short breath. I lost weight, I was 23 ((kg))…, I used to put on the clothes for people that are actually 7 years old from the underwear, 7 years, the way I had lost weight from here to here yoh, yoh, yoh, it was tough, my boy child used to carry me on his back, sister XXX knows him, he used to come here to collect me, he will then leave me here at the clinic maybe after completing my registration and everything, he used to leave me and say mum, here is your lunch box and sit with me. I used to be carried at the back like a child, for instance he used to carry the blanket and carry me at his back here.

M: Did you have TB or was that just HIV only?

P002: I was affected by both of them at once, TB also, do you understand, even when I was coughing you had to take the tissue and press, it was as if I have a hole on this and that side but since sister XXX was the one who was managing people that have TB even if you don’t come to collect the treatment, she used to look for the address in your file, she collects you or she will ask why did you not collect the treatment for so and so, she used to do it like that.

M: Okay, it seems to me our children gave us support more than anything else?

Ps: A lo::::t.

P002: More than our partners.

M: Okay and what about extended families like aunties, grandmas and granddads?

P005: They will judge you and ask, why do you not chase this one away since she will infect us with this disease.

P007: Actually, you don’t even discuss this disease with them, you know what, if you are seated there is a function or there is a family gathering their comments about a person who has this thing, ooh you know what, did you hear that so and so has HIV, at that time you keep quiet, for a short time you don’t say anything]

P006: [They say, do not allow her to cook.

P007: She should not peel stuff; she should just sit like this.

P005: Since she will peel and cut herself with a knife, her blood will spill over there, she must sit there, she must sit there until the funeral leaves for the cemetery, you also leave.

M: It means in other words, once they start talking bad about people that have HIV, you ask yourself what they will say about me.

P002: That’s why we do not disclose, others are afraid to disclose, there is a person who is now taking treatment until now her family does not know that she is taking ARVs, they do not know that she is positive, then I ask her my friend how do you cope when the time to take ‘your sweets’ come, she says you know what my friend, I just go the bedroom and pretend as if I am reading the Bible then when I see it’s that time, I take them, I ask her until when, she says she is afraid, if I disclose, I will be told 1,2,3 up to z in this house, do you understand me so I see even at home even when I am just …, I saw with my cousin even when she had to take a bath, we had to pour Jik before or after if she finishes taking a bath, her plate, she had her own plate, she had a cup, she had a spoon, they used to put them in the oven, these were not mixed with other utensils even when they were being washed.

P005: This person does not have TB, she has HIV, do you understand, that is why you will find that some people are still afraid to say, I am positive since our families, others, okay, they can accept you but others no, even though you make a mistake yes, it’s this AIDS of yours]

P006: [It’s making you crazy, it makes you mad ((Ps laugh)), that is what they actually say.

M: What about friends?

P002: Some of the friends they are right, you will tell a person who will tell you that you know what my friend think about your kids first, don’t think about what I will say, because even if I go and tell people, I don’t know where I stand. What I am happy about is that you as a friend gave me the light to go and get tested and not be like you because you are now telling me to go and get myself tested, do you understand me.

M: So with other friends when you disclose to them, you become a role model, they go and get themselves tested.

P005: I don’t have many words, most of my friends have HIV, I will not tell those who do not have it because they will judge me, ooh you see, those type of things that is why I tell myself that I am going to tell those that know about this thing since they know what is happening because they also have it.

M: So most of your friends are infected. What do others say about friends?

P004: Like most of my friends do not have it, those that have it are few but they do not have a problem and I contracted it long ago isn’t it, I started having it in the 90s, before the ARVs were available, so when I told them what the doctor that I visited said, they said to me ooh, you know, you are going to die, that is what they said.

P002: Just like that.

P004: That is what they said, I had lumps so he had to check what causes lumps, so he said he knows that what causes these lumps is Ebola, cancer, HIV so we will take blood and check it, after testing my blood he said to me, your blood has HIV, I was just healthy, I only started tablets last year, I never got sick, it took me 16 years after knowing for me to take tablets, I started last year and I was not sick just to bring down the CD 4 count ((we thing P meant increase the CD4 count)) so when I spoke to my friends, telling them that you know what guys, there is type of a thing, I would be lying not even one of them ask them why, you see, it’s just that they had to be more careful than before, yes.

P005: It’s these people that are aware of this thing and who have learned about it.

P003: Another thing that I am going to agree with my sister this side is that she is very strong because the time she had this there were not yet available, we are lucky because they are there, at that time you know a person who was positive hey hey hey ((with a sigh)).

P004: I had a friend who died during that time but the strength you know what made me strong even now is brought about by people who were before me. You see the people that kill you are the people who are before you. What makes you strong is who was before you, if I tell you as a friend, you know my friend, then I didn’t check on each and every visit, I was plating my hair at the salon, dandruff, after dandruff I realised that I had developed lumps ((glands)) I used to say I wonder why, I visited doctor xxxx (name), he is the one who said ooh, actually it’s over with you my sister you know but I had a friend whom I knew that I discuss stuff with, she healed me even now, I do say. She said to me, take good care of yourself, do this and that. Take care of yourself and forget about this doctor, until I started taking ARVs last year after many years without taking them, I totally did not get sick, my CD 4 count dropped but that is why I say people that are before you are important.

M: My sister, what did you want to say, did you want to say something about friends?

P006: Friends sometimes you tell them your condition but they are the ones that tell your family, you see that type of a thing, you did not send them there so you end up deciding to not have friends, you see, since they used to just tell my sisters, my sister said, I should come out clean if I have it, you have heard, what did I say, have you see that type of a thing, so I ended up not saying anything until she herself last year found out that she also has it, I said my goodness, you, you see and I have known myself for very long. I have known myself for a very long time since xxx (year) , have you see that thing, so even now I am afraid to tell her that I also collect them at 14, I just say uhm, uhm, ((she listens)) actually, they are good, they have since been saying, they gossip about you, they spread gossip that you are this type of a seed ((person)) so why should you.

P003: And the good thing is tablets have since been good to you ((says P with a chuckle)). You are healthy that is why she asks you it’s because you are well.

P001: Even the nurses themselves, sometimes the professional nurses have not accepted this thing, I once got employment from one professional nurse, I spoke thinking that he is a professional nurse, I said ‘sister’ this is my condition, we were still talking, discussing how much she was going to pay me, how am I going to get paid, we finished everything and he told me when I was going to start work. I then told her that ‘sister’ this is my condition, she said okay my sister, I will call you and tell you when to start. My sister that was the end of it.

P003: Really!

P001: Now, they say we should stop doing interviews because we will run out of jobs even with a male you cannot have an interview because you are not going to find a job because if you tell a man about your condition, they run away. If you tell them that this is my situation, they run away.

P005: That is why I just stayed on my own for once because where will you start telling this person, maybe she has his, he is afraid to tell you, you are able to tell them about yours but they are quiet with theirs.

P001: You give them a condom, if he says he does not want, you say you know, why, bye bye my brother because he knows.

P004: Isn’t it they do not talk. They do not talk.

P002: Oh I was still going to support my sister as she says she loss a job, the thing is, the thing is that problem that she has, I explained to my boss, she is the one who was calling me, she is asking me the time during which I will be back at work, I am telling her that I am still in the queue, I have come to take the treatment, when I told her about my treatment, she asked me what the treatment is for.

P007: Is she supposed to ask you that?

P002: She asked me what the treatment is for.

P001: He does not fit in anywhere there.

P002: I told her that it’s for the High blood then she said to me, when I come back isn’t today date is the 21^st^ , I told her last week that on the 21^st^ I am going to collect my treatment, she said, when you come back, I want you to show me that treatment.

P007: E-, e- ((meaning no)) it does not work like that, that is not her story.

P002: You heard the phone, I was talking to her, she is asking me about the time that I am going to take.

M: Now you are going to be expected to show the treatment.

P002: I won’t. The only thing that I am going to give her is the paper; I will say here is your paper ((A doctor’s note)).

P004: No matter what you are suffering from, it’s not her problem.

P002: She wants the tablets for high blood, that is what she said this morning.

P004: Hhah she is cunning. Is it a black or a white person?

P002: It’s a white person, she is lucky, she must be very happy.

P001: In my case it’s a black person, a whole nurse ((Ps laugh)). Worse then she is working with this thing, I was open, hoping that she was going to help me, hoping that when I say I am suffering from this, she was going to tell me what to do. She said, no my sister, you will hear from me, I will call you. She was no longer taking my calls, where I was asking, when do I start work.

M: We are trying to finish this up, the community how was its response towards you after you had lost a lot of weight because of HIV?

P002: As we have mentioned and as our mother has mentioned, these people gossip.

P004: You see by the move of curtains, come and see her, I told you, she is very sick ((says P mimicking gossiping neighbours)).

P002: We will get free lunch here.

M: What is a free lunch?

P002: My funeral.

P006: The problem is she is pointing fingers at me and these 3 are facing her, she says uhm such a fresh person whilst I am this thin ((Ps laugh)).

P005: Even when I am coming here for my visits, they frown at me, you said you are not dating but all the time when you say you are going to the clinic what do you go to do there, to take an injection, I am contracepting with an injection, I keep the small card handy since I just say, I am going to take an injection, can you see what is written here, Family Planning, I keep going for an injection, if I come here, I come here to get an injection but I was here on the xxx (date) to take an injection, I am back on the (date) , what are you going to do at the clinic, I said I am going to undergo a pap smear ((Ps a laughing out loud at this point)). Uhm, I heard, they said they are going to do a pap smear on us, maybe she is still going through the pap smear, when I get there, I will complain, that queue was long, everyone wants to do it, hey it’s packed, it’s for the uterus.

P001: There is another thing that kept us mad sister ((says P addressing M as a professional nurse)) regarding these ARVs previously when you take ARVs for a long time, as we have been taking them for a long time, we started taking them in xxxx (10 years ago) , they say once you reach your 10^th^ year after using them, you die.

M: What do they do to you?

P001: They say you die, everything else ends, you die, I even came here to xxx to ask and said I have completed 10 years what is going to happen, they say they are finished and they become yours, these have gotten too deep into your system so they are no longer working and so after 10 years you need to expect death and I once had that frustration my sister. That is why they say you live for more than 10 years. I had that frustration that as I am finishing 10 years it means I am ‘leaving’ so I was lucky I found sister xxxx (name) and asked her if she knows that I will be dying, I said to her look at the number of years, she said no, there is nothing like that. So she said I should not listen to people since people say a lot of things, all those who knew when I started them said hey, can you see that the time is getting closer, how does it actually work, you see.

M: You have started talking about health care workers, the doctors, the nurses and the counsellors, how do they usually respond after we have lost a lot of weight as a result of HIV? My sister has indicated that sometimes they allay our fears and anxieties and tell us that there is no such thing. What else do they do?

P002: Even for them at the clinic, it depends on the sister that attends to you since there are others who do not address us properly, when you lose weight, they will say, you are just reckless what is happening]

M: [What is *ukuvaqula*?

P002: It means you are just opening it up, you are going to die, you are going to die, it means when you lose weight or when they see something it means you are just going to die. Even me today my viral load was found high but I last had sex last year ((2013)), I got divorced last year September, I do not have a boyfriend but they are telling me that the viral load is high then they sent me that side then when I got there I asked that nurse, she looks me in the eye and then says and then your viral load is now reading thousand why because all the time, she opens these pages where it was low or undetectable and why today am I saying I am shocked, I also said I am surprised, she asked are you using a condom, I said for what because I am not dating, I don’t have a boyfriend, do you understand, I am surprised why are is it like this, do you understand, she asked me what is it that I am doing now, then a thought came to mind that oh by the way there is a herb that I am using. I said no, perhaps it’s a herb that I am using, she asked what are you using it for, the herbs that they usually sell even here at the xxxx ((taxi rank)) there is a small complex in there, there are these herbs that they sell for the chest, for the high blood, arthritis and all that, now I think that it’s these herbs that made my viral load to be high, it suppresses these ARVs, I told her and she wrote down and I explained and said, you know what I am going to stop using it completely because I am shocked by what you are telling me since I asked this guy, to explain what this 1000 is doing in my viral load. He said actually my sister sometimes it may happen that you are perhaps not using a condom; I said I am not dating.

M: So you are saying that it depends on the nurse that is helping you.

Ps: Yes.

P002: Yes, others treat us well, others judge us, and they make assumptions regarding what we did.

P004: They are a step ahead of us.

P002: Do you understand me.

M: I understand you. So in general, for you as people who are living with the HIV virus, how do we usually feel when these things happen, there are ladies here on the side, they are gossiping, here are our aunty when there is a party they discuss these painful things about people that have the virus, there are nurses also when we visit the clinic they say we did not use condoms?

P004: It painful, you feel shy of the circumstances. They are killing us in actual fact.

P005: As I was saying that is why people are afraid to come to the clinic to collect the treatment, things like those. The patients say, that nurse said this to me.

P002: They are afraid, a person realises that if they were at the clinic the other day, they wonder what they will say when they meet next time, most of them decide to go back home when they are at the ((clinic)) gate.

P003: What am I going to say, I am trying to add on this that you have said, you become afraid because we are fighting with the virus, you are controlling yourself isn’t it, you are trying to forget it, no one can like that we are trying to forget about it, that is why we are seated like this, I would have left quickly after seeing these children, they are asking me to discuss my issues in front of these children, I am not afraid of anything, they can say whatever it is that they want to say but it’s painful if it’s said at home. You sleep at the same place, you eat with them and they keep discussing this story that you are trying to remove from your mind because another thing that makes you to lose weight is stress. You do not rest, your mind works night and day.

M: So we feel sad?

P003: We feel sad. You keep asking yourself, the mind; it’s as if I created this myself whereas it happened on its own. You see that type of a thing.

M: How do they respond when we gain weight?

P005: They say the ARVs are working, they call them *izinkobe* ((loose dry maize)) .

P003: Really, they are good on you.

M: Who says that?

P002: It’s the community. No, these sweets are actually good on you.

P001: Others call them *umgwinyo* ((something that you swallow)).

M: Number 1 has xx (Province name) ((Ps laugh)).

P001: You know we have been luck at xxxx (province name) for accepting this thing, what I have noticed here in xxxxx (name of city), people have not yet accepted this thing, because when you talk, they ask you to not mention that thing, you must not say that you have this thing ((says P in a whisper)). In xxxx (province name) wherever we are, at a party, you will hear us saying when are we going to collect ARVs, when are we collecting ARVs, when are going to the fountain of life, when are we going to go and get life, you see that helped people, you see, you will find that I will tell you and say so and so when are you going to go and get your fountain of life, when I get home, someone will visit me and say actually mother *((name withheld for confidentiality reasons)) I have come to discuss this type of a matter, this is my condition, please help me what did you do, be free my sister, if you want us to go, I will take you there, I will take you there, we live that type of life even where we are drinking we discuss this thing. We just talk non-stop, when we are drinking having this and that, our wines, then we will say guys hey people are dying, you must go and get yourself tested for the virus, you no longer die after testing it]

M: [So it means once we gain weight, others are happy, others say it’s the ARVs that are making to be like that, otherwise our spouses, our partners when they see us gain a bit of weight?

P002: They say, actually we are happy; we can see *izibhejeje* ((fats at the back of your waist area)).

P004: Even when you want to go somewhere, where are you going now baby, I am just going to the shops, you see that type of a thing, no, I will accompany you or if they do not come along, you will see him standing there at the gate looking at you, when he sees you looking his side, he will direct his attention on the other side, whereas this person has been looking at you since you went to the shop.

M: So they are happy when you gain weight and what about children, what do they do when they see the body gain some weight?

P002: Same applies to them also ((Ps agree)).

M: What do the extended families say?

P002: They talk as if they are happy for you even though they are not.

P003: It’s these ARVs.

P006: They have a tendency of talking as if they are happy for you even though they are not.

M: Oh they are the ones that say that.

P002: They are people from the outside isn’t it? ((Ps laugh)).

M: Hoes does it happen that extended family members just become like people from the outside?

P006: It’s the same as when you do not have money, once you get money, they say, you look like you do look like you have it now.

P005: At home, I stay at my great grandfathers place, it is kids now at home, there is one who has two kids, from my big aunt’s side, they are 3 in actual fact, we leave the two at my mother’s and father’s that are still there, they are able to call her a step child but she is also my mother’s child, you see.

P004: Things are not good.

M: And how do health care workers feel when you gain weight?

P003: They are happy.

P005: They are happy, isn’t it they can also see that this person complies with her treatment, they will go back to the CD 4 count results and say can you see, you are very much better. You know you cannot start like this, you see those type of things because sometimes if you don’t understand you want to know what is happening now, am I doing well or they will tell you, you are very much okay, you are complying with the treatment, you are okay, you should continue like that my sister, you must stop this and that. They do not ask us to stop everything isn’t it, they say we must have limits.

M: How do you feel when they say these good things about you after you have gained weight?

P005: You wish to come back for your next visit ((Ps laugh)).

M: Oh, they give you the motivation?

P002: Yes, isn’t it you tell yourself that if you keep coming, you will keep receiving this news.

P005: You also want to take it and share it with others that have not yet accepted.

M: When they call it sweets and *izinkobe* ((loose dry maize)) saying these are the things that make you gain weight, how do you feel?

P003: We continue and take these corn that they are talking about so that we will be able to live, by the way we are not living for them isn’t it we are also living for our children or those family members that love us, isn’t it.

M: We talked about changes in our bodies. I think some of us indicated the manner in which ARVs changed us, my body shape was not like this, my body shape was thing and that. I want you to perhaps describe how body shape changes present themselves?

P005: A flat bum.

P002: Dark complexion, the change of the colour of the skin.

P004: You get cramps on your feet.

P001: Breast becomes big.

P003: Calf muscles.

P005: Hair becomes weak, are a few and fall off.

P002: The last time when I went for blood test, I heard others talking about the teeth, saying that as we continue taking ARVs, you end up losing your teeth. You just lose them.

P001: For now, mine are breaking. It will be like a bone and break; you see on this side, they have become yellow.

P002: I am shocked, I think they did not explain to us about the teeth, they never touched it. I only heard about this one of the teeth last month when I came to take bloods.

P004: I even put on false teeth; I don’t have them here.

P001: For me, it has started on this side, when I make any move, it falls as if it’s soil, when I check, my goodness, it’s a tooth. I have never filled them with cement; I wanted to make an appointment here since I wanted to find out what is happening because I do not understand my teeth. I have never had a tooth problem. I will never feel the pains, it will just break, it will be like a pumpkin and then break, it’s not painful. They just keep breaking.

M: Okay, it’s teeth, hair, calf muscles and developing breasts]

P003: [Men develop breasts]

M: My sister also mentioned that her breast is growing, so in both men and women the breast will develop. The calf muscles, are they in women only?

P002: It’s in women most of the time.

P003: Men, by nature do have them.

P006: Thin legs! As summer is approaching, you want to show off this here. The knee, Jesus it; sharp, it’s a sharp point.

P001: Nightmares, a sheet gets torn.

M: What do men’s body become when their body changes, other than their breast?

P002: There are those whom we were attending the support group with I noticed that his skin was changing, it will be like you turn grey, he will have dandruff, then other’s nails become brown here at the top on the inside, others develop sores, what do they call it, a rash, others will have shingles, yes, the belt, they call it the belt and others will have piles, those type of things.

M: These things that we have mentioned, for instance, do we have those which we would say were caused by HIV as a condition on its own and then say these ones were caused by ARVs?

P002: The thing is, when you start getting sick, the symptoms appear isn’t it in HIV, others get infected with TB first, those are symptoms, it means as my sister has stated that she started by having dandruff and then developed glands, I had piles that could not heal before I could start taking ARVs, then I had another discharge on the vagina that could not be understood, those type of things but when I started taking ARVs all those subsided, boils that I used to have did not appear anymore, do you understand me, but what changed my complexion was a TB treatment, since I changed my complexion on the face, I was dark, every morning and evening I used to vomit when I first started these ones for TB that is why I am saying the HIV itself has its symptoms, the ARVs also have their own symptoms, do you understand me, that is just how it is.

P001: Before you can start taking them sometimes pneumonia is a problem, it attacks you frequently, it will be a sign that thing are bad, persistent head ache, that cannot be cured by tablets, you will then start having that head ache, then it’s diarrhoea hhah, my sister you visit the toilet often ((Ps laugh)) actually my dear, it’s something that cannot be controlled, vomiting, loss of appetite, it also happens that even on the early days of its use you have a running stomach but with time it ends, but pneumonia subsides completely once you start using the tablets. Pneumonia is what attached me.

M: All right, have you ever heard of this thing called lipodystrophy, a dislocation of fats in the body of a person like for instance some people will have fat gain in the body, like it would look as if a person is pregnant. Another one will develop a buffalo hump here at the back, others will lose fats on the face, they will have bony faces, other will be…, you mentioned a flat bum, and the arms may become bigger for others, as you have mentioned thin legs and the calf muscles, those are the body shape changes, how do our spouses usually respond to these? Maybe even if you are not mentioning yours, just mentioning maybe a friend that we know whose shape changed and where her partner said this that and that.

P005: Ahh can you see what these tablets have done to you ((Ps agree with her)), look at how you are because of these tablets, why don’t you stop taking them whilst they know very well that you cannot take them.

M: You wanted to say something number 2?

P002: I am saying, you know the people that gossip, if you have developed those calf muscles and a flat bum, you know with a flat bum they say the balloon deflated. If you developed calf muscles they will say, you participated in a South African cross-country marathon but ((not clear)).

M: If they say a balloon deflated, what do they mean?

P002: It means a flat bum, imagine how a balloon looks like when it’s inflated.

P005: One will end up asking you, ooh what is happening with you, I know you as being a very fit person now your body shape has changed, what is going on. I know you when we were studying together; you were a student that had a good body shape, what has happened. You are maybe next door ahh there she comes, it’s these tablets, it means she also has this disease, you see, those type of things, this disease.

M: What were you saying number 6?

P006: He will say you are a tortoise; he will call you a tortoise.

P002: She is telling the truth, I also heard them in XXX [Name of the location], I heard them say that thing.

M: They say, tortoise?

Ps: Uhm.

M: How do children respond when you change?

P006: They die on the inside.

P002: Isn’t the child will not tell you because you are their mother, they are afraid to tell you since you will be sad.

M: They just keep quiet.

Ps: Uhm.

P002: Ja or they will ask what is this now mummy? You see, you will also need to explain in another manner.

P001: Mine did say, they said mummy, they were aware that I had asked them to tell me if it’s time to take the tablets, so I will hear them all the time saying mummy, it’s 8 now, take them, the one will come with water and the other one with tablets.

P003: Oh that’s sweet.

P001: Maybe I am still in bed, another one will come with food, when I started changing, my son, the one who is a born-again ((Christian)) used to say, you know mummy stop taking this thing, I will pray and you will be healed, I will just say, in the Name of Jesus Christ, you will be cured of this thing mummy, can you see how much you have changed, you had a good body shape mummy, you know, iyoh I said you know my child, it’s you who believes in that and I don’t. I am going to die, so let me just continue.

M: What about the extended families, what do they say when they see me develop thin legs?

Ps: Hhah.

P002: Isn’t it we explained how the families are, they will give you bad names, yoh yoh they will call you by them man.

M: So the extended family and the community]

P002: [They are the same. It’s the same thing.

P004: The thing is extended families can tell people that are not relatives about you, who will then just gossip about you, my aunt told me something else about you, she said you have this disease.

P006: It’s a dishcloth; we will dry dishes with it ((they laugh)).

M: What is a dishcloth now, number 6?

P003: Isn’t they are telling you that as the flesh is loose on the bones, the body moves/shakes.

M: I am getting to hear this for the same time ((Ps laugh)). I know the one of OMO.

P001: If you know the one of OMO, you need to know of a dishcloth and.

M: I have heard of that one, whilst I was still in xxx (province name)

P001: Where is XXX from the XXX family, you will then know that they are referring to us, then we will say ‘that is us’ ((says P as if she is singing)) ((Ps laugh)).

M: You see that one of XXX I know it from xxx (province name), that’s why I am saying you have the xxx (province name) terms. The health care workers like nurses, the doctors and counsellors, how do they usually respond when they see people changing their shapes and having shapes that are completely different from the ones they had before?

P006: You also tell them that you just see yourself]

P005: [You need to tell them, to say, can you see what these tablets have done to me now.

P002: They will not tell you, you are the one who will need to tell them that my body is changing, please check sister what these tablets are doing to me.

P005: It’s these people that are inquisitive, that are now aware]

M: [If you tell them, what steps do they take?

P002: They respond well, they will tell you that we told you that if there is anything that is changing, you must come back, we need to take your blood and check the tablet that is not good for your body so that we can be able to change it, for instance I used to take Stavudine. Actually, Stavudine used to be very bad for me, I used to experience a severe short breath to be it’s as if I am dying, I have some pain here at the back then when I came here, on the first week when I had come to tell xxxx (nurse’s name), she simply sent me for bloods, they tested me, when my results came back, they said it’s Stavudine.

M: Okay, you then stopped?

P002: Plus, on the face, I was losing fats ((says P mimicking)), a balloon now, I don’t know what is happening, it was as if I put on the false teeth, you see that type of a thing, I told them, I had bones here.

P001: During our times, tablets were not many ((to choose from)) when you say here it, please change me to another one, they would say, there is nothing we can do my sister. Stavudine is Stavudine, it was Stavudine only.

P006: Yes.

P002: At that time, it was just Stavudine.

P001: It was only in 2006 that there were Tenofovir, they changed me then but from 2004 it was just Stavudine, no I used to look like I am lifting weights, it went on and on.

M: Your upper part just getting bigger?

P001: Actually, it was getting bigger but even when I started taking Tenofovir nothing changed, I am still like that, this is my body shape now.

M: But it is no longer getting worse?

P005: It means even if you changed them before, you were not going to regain your original shape.

P001: Isn’t there is a lot of Stavudine in my blood.

P006: They will say it’s full, you drink drip, I used to drink drip until I didn’t know what to do anymore, drinking a drip.

M: Why were you drinking it?

P006: They said it takes Stavudine out of your system.

M: Does it do that?

P006: I don’t know.

M: Who said that?

P006: The nurses at the clinic.

M: I didn’t know that you can drink a drip.

P004: There is this one that you can drink.

P005: There is this one that you drink and prepare for yourself. You put 8 sugar and 8 salt ((tea/spoons))]

M: [Not Sorol.

P002: Yes, it’s Sorol.

P006: I used to include it in my grocery list at the chemist all the time, I had to have it and then I used to pour it in a bottle, I used to have my bottle, I used to drink even at work, I was not drinking that water, I am drinking a drip.

M: Okay because my understanding is that it gives you power.

P006: The power for what because you want to gain weight ((Ps laugh)).

P002: Or to regain your complexion ((Ps laugh)).

P005: I cannot say a thing about what the tablets did to me since they never did anything to me, they ask me what the tablet are doing to me, I tell them they are not doing anything to me, even my file is written, no complain.

P002: They take time, even for me they took time, the thing is, when I started taking Stavudine I had a very flat waist line ((flat tummy)) when I get in…, there was this security who used to say actually I brought you lunch girlfriend, then I used to know that I will get lunch when I am here. I had a beautiful figure, hips and legs. Actually, I was very beautiful; when I enter the security guard used to say ((they all speak at once))

P005: What did the security guard say?

((Laughter)).

P001: But they do not do anything to other people, there are those who started in 2004, I meet them they do not have anything. There are those that we started with.

M: Number 7, why are you this quiet?

P007: Remember that I am new here.

P001: You see as you are starting with a Combination one, you will not experience problems, I am telling you my sister.

P005: My complexion has become darker because it’s itchy, I didn’t want to take it, they said we issue to everyone, you cannot choose.

P006: I tried all of them, with none of them suiting me perfectly.

P001: I used to take 3, I only started on the xxxx (date) to use the Combination pill, they changed me to it.

M: You are now on the Combination pill?

P001: I started in xxx (month).

P005: Actually, they are difficult to swallow, on those that came in 3s, there was this one which could not go down the throat.

P006: This one is now worse.

M: The Combination one?

P005: This one, this is the one that we are saying it’s not easy to swallow ((P show M the pill)) the other one is white whilst the other one looks peach.

M: This tablet is not very big?

P005: This one, it’s very small ((Ps discuss the sizes of tablets)).

M: Can I close, we have come to an end of our discussion, right. Thank you very much. The time now is xx:xx end of focus group discussion

The group discussion ends
