# Supplementary material for: “If we lose it, we are worried”: Individual and provider level perceptions towards weight change among people living with HIV who undergo TB screening in routine health care settings in Gauteng Province, South Africa
Source: PLoS One. 2025 Sep 22;20(9):e0331904. doi: 10.1371/journal.pone.0331904 (PMC12453174; doi:10.1371/journal.pone.0331904)
Supplement: S4 File — (ZIP) [file pone.0331904.s004.zip › S4 Transcripts_final/FGD 5.docx]

FGD 5

Transcribing Conventions

- **...** Ellipses indicate talk omitted from the data segment
- **(( ))** The transcriber’s comments.
- **( )** Empty parentheses indicate some talk was not audible or interpretable at all (we include the line for instance 20:15)
- **(.)** A dot enclosed in parenthesis indicate a short silence
- **[ ]** Square brackets indicating beginning and the end of overlapping speech.

A Group Discussion Starts

M1: Date xxxx (interview date), FGD starting at xx:xx with x participants, xxx (gender).

M2: Do not use your names, so we will say, 007 is from XXX [Name of the location]and what else do you want to know?

M1: Are we employed so that we can know each other better.

M2: Okay.

P007: I am from XXX [Name of the location], I am not working.

P004: I am from XXX [Name of the location], I am not working.

P002: I am from XXX [Name of the location], I am not employed.

P001: I am from XXX [Name of the location], I am working.

M2: What do you do my brother?

P001: I am from XXX [ Name of the location] I am working.

P005: I am from XXX [Name of the location], I am not working.

P006: I am from XXX [Name of the location], I am not working.

P003: I am from XXX [Name of the location], I am not working, and I am a volunteer care giver.

M1: Okay, I am XXX [ Facilitator], a researcher, I am very happy to be with you today.

M2: I am also a researcher, so when you visit the clinic for your appointment, do the professional nurses ask you if you lost weight? (.) They ask occasionally.

P007: They put us on a weighing scale.

M2: They do not ask, others?

P001: They do not ask.

P007: they ask you to be weighed, what your weight say, you will weigh yourself and tell them what your weight reads, from there, they will tell you they will not tell you why you lost or gained weight, they are just less concerned about the weight.

P001: In my case, I realised that I had lost weight and I told them and then but I think they should ask, they should not not ask.

M2: Why do you think they ask? Why do they want to know about the weight?

P004: I think that perhaps they want to know if the treatment is working.

M2: Okay.

P002: I think that they want to know if we are using the treatment in a right way.

P003: They want to know if you are taking the treatment accordingly or if maybe there other things that you are happening in your body.

M2: Okay, what do others say?

P001: I think they ask because they want to measure your weight against…, what do I want to say, your weight can tell you a lot about your progress and the type of treatment you need to take.

P007: I think that they want to check before how you were before you started using the treatment and now that you are using the treatment how is it on you.

M2: A person living with HIV, if they ask them about the weight, how do they feel, how do they feel about being asked?

P002: No, I think that they feel good since isn’t it they want to find out how they are, they are asking them because they want to know how they are so I don’t think that you can feel bad if you get asked something like that because you are aware that the disease is in their body, they are looking after you, they are observing things that are taking place.

M2: Okay, let’s then use these pictures to help you think, you are at a taxi now, you are coming to the clinic, what’s going through your mind, how were you feeling?

(.)

M1: Today, as you were coming here, what were you thinking, when you were still in your transport, on your way here?

P002: We were thinking that I wish that when we get here, we will find that our weight is good.

P003: For me, today, since I know that I have come to take bloods, I just want to know the level that my CD4 count will have risen to. I want it to have increased and not decreased.

P006: I do have a problem when I come to collect my medication since there are things that are written here on my files, the thing that I cannot see the results even when I ask a professional nurse to explain what it means, I encounter a problem with my files, she does not tell me but she does tell me sometimes, your blood is right or that it is not right, I will say I need them to explain what I need to do so that my things can be okay.

P004: My appointment date is not today, it’s on the xxxx (date), to take blood, the treatment they gave me was inadequate, so in a taxi on my way here I was thinking that I will find the professional nurse since they like to scold at us, why did you come today since it’s not your date.

M2: Now, when you are waiting there at the clinic, waiting to be attended to, what are you feeling?

M: Generally, when you are seated at the waiting area waiting to collect the ARVs, how are you feeling?

P006: I will get there, it is quite a number of us if it is a date for us to collect the medication, I am not happy because I will be asking myself if the place that I am at is a right place, you see?

M: What do you mean when you say, am I in the right place?

P006: I mean, I have come to collect the HIV treatment, why I am the one, what does that mean, I feel as if I have been disowned, I do not feel good, I do not feel the same way as I usually will as a person.

P007: What frustrates me is that our cards are not the same as those of a person who have come to collect the treatment for another disease, for instance, HIV people are given green cards so sometimes you do not need to tell a person and sometimes you meet your neighbour here at the clinic, they do not know that you are HIV positive then, when you are seated there, they will see with this green card that we carry that it means this person has come to collect the HIV so we are not the same, of which then, we do not like that thing, you cannot disclose to anyone that you are HIV positive.

M: So the cards disclose for us?

P007: Yes, can you see that thing my sister, without you telling them, you meet a person that you attend church with, you meet your neighbour, you cannot disclose to everybody, there are people that you need to disclose to so now they can see that at the clinic she had a green card, so it means she is taking the treatment, that is what frustrates me then my sister.

P001: Coming to that issue the card, it feels as if we have been separated, you see, it’s as if they are here on the other side, so there is no such thing as we have come for treatment or she has flu or anything so we who have come for the treatment and for everything it can be seen that we are on this side, it makes other people to be afraid that we have come for treatment and obviously if you are experiencing difficulties, when you are already weak people see that you have this thing, so the weight loss affects many people especially because of the environment that we find at the clinic but because you want help, you will go.

P003: I do not know, isn’t it we attend on different dates, maybe the professional nurses once mentioned it maybe when we were not there, on this issue of the green-card I think if you take it out for the first time, when it comes back, it comes back with this card ((in the files)) so I think, according to my opinion, I was thinking that this card ((not clear)) today, I used it ((someone agrees with P on the background)), I had it in the bag so that when I get to the consultation room with the professional nurses, she is the one that checks the date for me and even when I go to take bloods, she always tick somewhere, I have been there 3 times, I now have 3 cards here, if I take out this card, they draw my file, when they give it back, they return it with this one inside, so I learnt it on my own, that now that we are all together at that hall, we used to be separated but when I ask from the counsellor why, they say I complain, you are saying we must put you in this room, you will recall you were here, they say it’s for people that are taking treatment for ARVs, so now the one who is suffering from the high blood, diabetes, and everything else, we all gather there, we have been put together but those that take out this card are a few, there are just these 4 cards that they give us, I don’t know there is that day where they explained which one has to be used, I don’t know, they just put them here, when I got here, I am sure there is another one here, here it is, when I come here next month I will take this one out, I then asked myself, my goodness, it means this card…, even now, I decided to test it , I took this one out, isn’t it has my file number, I used it and she didn’t even ask why it does not have a date, it is plain, what am I going to do, she just said they must come and draw bloods, I didn’t understand if she says blood because there are many people who take blood, I went to her and asked about the blood she was referring to, I have come to take blood for theCD4 count they told me about the treatment, she said, those are the ones I have come to collect, they took our files to the side, called us and we were in a queue in another place, as they have brought us here ((to the focus group discussion venue)), I am not sure about others, I don’t know.

P001: This of accessing your file, the green one is for everyone and then this one which looks like is lime green is the one the one that you take out when you are inside the consultation room with the professional nurse, privately, you don’t just give them this but this the one you use ((don’t let them see it when you are just seated – adds another respondent))

P003: Actually, the way I look at it, maybe they explained it perhaps some other day when we were not there.

P001: It means they fixed it from there.

M: These discussions are useful, you see?

P: Because even at hospital if you come with card, then there is a blue card that you use, what you are suffering from is the information that is in your file with the professional nurse in the consultation room.

M2: Okay, when they are weighing you, you are standing on the scale, how are you feeling?

P004: You ask yourself if perhaps today I lost or gained weight.

P001: I used to weigh myself even before I got infected so even before I started having symptoms, I knew before since I used to weigh myself, so now when I weigh myself, I am just on the lookout and hope that my weight has not increased but that it is okay for my height.

M: What do others say?

P006: Getting on that scale frustrates everyone a lot, sometimes I take the medication, I do not skip days, but sometimes you will find that it reads 60 or 50 something you will find that you are asking yourself, why is it on and off, sometimes it goes up, sometimes it decreases instead of being stable in a right manner, you see I once asked the professional nurse why in some instances my weight goes up and down, I said I take the medication accordingly, I never skip a day, you see, she said maybe you do not eat, it’s not eating well, not being free, I said, I cannot not eat well since she asks me about food, sometimes I eat and sometimes I don’t, you cannot have food all the time, you find out now that you are not working and there is no one at home who is working, you see?

M: So you are saying that you feel uncomfortable when you are at a scale because sometimes you experience weight loss. Okay, what are you saying at the corner there? We do not want to make you sit at a place that will hide you from us whereas we are having these very interesting news, we don’t want to leave you out of it.

P005: According to the issue of the cards, I understand that they gave us blue cards so that we can all be the same but the files are not the same, our files, we people with HIV are different from those that are suffering from other diseases, the files are different, it’s still the same that we have been separated since our files are not the same as those we are seated with, my HIV file is not the same as the one next door who has diabetes or whatever.

M: Okay, if you are standing there at the scale, how do you usually feel?

P005: I feel better on the scale, the situation is, when I am to climb to scale I am expecting anything, having lost or gained weight because of the conditions that I stay under. I can eat healthy whereas I am not happy at home then that thing affects you and makes you to have stress and you lose weight.

M: Okay, as we were going around having discussions with people in different areas, most of the time, we used to hear that people feel frustrated if they have lost weight. It’s for the first time that we hear that you get worried even when you have gained weight. We are interested in finding out exactly what it is that keep you worried when you have gained weight?

P002: No, if we gain weight, I believe that we are happy, because it means that no, we are happy, we are eating well, we are doing things the right way and that our medication is working well but if we lose it, we are worried because it’s like we are not taking these tablets.

M: Okay, okay, so number 2 says, he is happy if he gains weight because he feels that he is healthy and eating well. What does number 3 say?

P003: I get very motivated if I gained weight, I feel happy to say, no, life is good since because when I lose it, I get worried, sometimes I lose weight even when I thought I live healthy, I want to know what happened, it’s better if I had a common cold or a bit sick, I will then say, I lost weight because I was not well, now if I lose it whilst I know that I complied on treatment that’s when I get worried. If it is up, I get very happy, it motivates me.

M: Okay we have heard from those that want to gain when they are on a scale, we now want to hear from those perhaps that can be a little bit worried if they can find out that they have gained.

P007: My sister I will have a problem if I gain a lot and not lose a lot, but to gain and go back to that weight that I was in before I started taking treatment not that thing of gaining weight until I become overweight, you see because then you see I am no longer okay, I no longer feel myself properly and also losing weight, I would not want that, when I started taking ARVs I was 52 (kgs), do you understand so my weight before I started ARVs and before I got sick was 89 so at least when it reaches 99, 100 it should not exceed 90 because before I started taking treatment it was 89, you see, so once it’s 99, 100 so I don’t like it, I am not comfortable since there was once a case that I had gained weight, I was worried of the cause, you see? So anything that can bring about weight gain or something and I don’t think food makes a person to gain weight.

P004: If you gain a lot, you will get diseases like high blood, it’s better if it’s normal.

M: We were talking about the time we are weighing ourselves there at a scale, once we are done weighing ourselves having left the scale, this is a woman who has finished everything that she had come to do at the clinic, she is leaving now, she is going home.

P002: It indeed good when I realise that I did not lose a lot of weight as such, it is within its range or if you have gained a bit, you are free but if you lost it, actually you have a pain and ask yourself what is happening.

P001: As people there’s no place like home, if you go home, all the burdens are lifted especially when you are from here, knowing very well that you have done everything to look after yourself and that what remains is for you to look after the people that you love, your children and your family and how you eat, so when I am from here ((clinic)) I feel that something has been lifted.

M: So you feel relieved, okay how do others feel after they have taken their time and having done everything?

P004: We are happy because we leave our homes early in the morning and sit there, we left home without eating and without even drinking tea, at that time, we are happy that we are now going home to eat ((Ps laugh)).

M: Whilst on that, we have refreshments, we do not know, we would like you to eat when you are done or would you prefer that perhaps you have your refreshments whilst we are talking, we ended up not asking you?

P002: No, we can be happy ((Ps laugh)).

M: What do others say, we are on our way home now, we are leaving the clinic, we have done everything, is there someone who has not said anything who says she feels somehow different from that which you have mentioned? (.) I think you have explained how you feel if you lost weight, is there someone who has not said how she feels after losing weight?

P005: It is that which they have mentioned, I feel good.

M: All right, you can continue my sister?

M2: Sometimes when you visit the clinic, a professional nurse asks you if you lost…, before she weighs you, she asks if you have lost or gained. You respond and say, you lost, why do you say you lost weight? What makes you think that you lost weight?

P004: You can just feel that your body is weak.

M2: You feel weak.

P005: Sometimes you will find that you can see that your clothes hang on your body, to say I lost, I lost weight, when it’s tight, you can see that no, I have gained weight, I weigh more than what I normally weigh.

M2: What do others say?

P007: Sometimes my sister, you realise on the time before you go to the clinic, perhaps before your clinic visit, there are things that frustrates you at home, you are not working, children want this and that, you feel hurt, then what does the body do, it loses weight.

M2: What do others say? What else makes you to think you lost weight?

P001: We all have clothes that we like, for instance that when you have them on, you feel good but if you do not feel good on them you know that something is wrong. The other thing is before, you find that you tighten your belt you know I see myself regaining my weight because even today I don’t have the belt on and these are my old clothes so it shows the improvement, I also get encouraged since I can feel that you know what, I am getting better, yes.

M: What do women say, how do we see that no, we spoke about the dress size and spoke about feeling weak, we also mentioned that sometimes if you have been stressed, we also talked the belt and how it fits you or dressing without it, we then realise that we have regained our weight. (.) ((P coughs)) how do we see that we have lost weight. What is it that makes us to tell ourselves that we have lost weight when we arrive at the clinic, even when they do not ask us but just knowing that I lost weight?

P004: When I first came here for a test, I had lost a lot of weight but so far since I am taking the treatment I do not have a problem since my weight keeps getting better.

M: How did you see that you lost?

P004: I just saw with the clothes, I had started putting on a small size which I never put on in my life, I realised that there was something wrong that was happening in my life.

P002: I used to see that I have lost weight through the clothes that I used to put on, they were no longer fitting me, that was when I used to come to collect the tablets. So if I see that there is something wrong, I would feel as if I don’t have power and will therefore feel weak, I have never had another problem, my body is smooth and everything is okay.

P007: Like besides the clothes, you can also feel that you do not have a good weight, you can feel that you are not okay so you will feel that there is something wrong in your body besides the clothes, you just feel your body.

P003: I once felt weak, that was before I could lose weight and the clothes were loose and big, I used to feel tired and realised that there is something wrong. I realised that the CD4 count has dropped.

M2: When people living with HIV lose weight, is there stigma out there, do people say, can you see this one?

P001: Yes.

M2: Okay, what do they say?

P001: You know people are very insensitive, yes, even if you lose weight not even due to HIV, there will be such things that they will say to you, there are names that they call people with HIV by, so they talk about it as if you do not know the language or the lingo without even knowing that they are referring to you so those type of things, yes.

M: Really, there is a lingo?

P001: Yes. Anyway isn’t it we are young and we are still socializing and the stuff, we understand them and that is why it’s difficult when you are positive to be a person that you were before because you think everyone says the same things maybe that you were saying to people.

P002: People talk after you lose weight and they say that actually this one looks like this because of this disease so if you are passing by you find that they are talking maybe they are talking about another one if you are also like that ((positive)) you do not feel comfortable, you can see that actually these people are talking about me since this person that they are talking about is like me, what they are suffering from is what I am also suffering from so you do not feel okay.

M: What do others say?

((A cell phone rings)).

M: I am going to ask that if we can put our cell phones on a silent mode, if we receive calls go outside and respond to them, we do not have to worry, we are worried that this noise can affect the recorder. We are saying that they talk, what are they saying and the names that they give us when we lose weight, what names do they give us, the lingo?

P003: They call it Z3.

M: We get to learn of this name, it’s a BMW right?

P001: The thing is Z3 is more like old school, you see it’s old people that like to talk about people, within the younger generation, they talk about a tracker, for instance that you are tracking obviously isn’t it a tracker…, if your car has a tracker attached to it, if it’s stolen the police are able to find it, no you are tracking, fire and all those words, you see, if you are not familiar with that township language you won’t know.

P006: Since I was once with them, stayed with them, they talk, the thing is they don’t know that I have it, you see, they say, did you see that person there with AIDS, here, these days they say you are suffering from the Surf ((we thing P meant OMO since it’s a soap with 3 letters)), guys, what is Surf, really, my man, you do not know that Surf has three letters 3, you see it means that obvious everybody knows Surf, they say, my brother, they are failing to clarify, Surf is three letters, the thing is I am not happy with those things, I ended up leaving them, you see, it’s better if I stay with people that have the Surf (Ps and Ms laugh) the thing is if you sit with guys that speak bad and also, I will not tell them that I have Surf as you pointing at people that have Surf, you see.

M: We are asking for these names.

P001: There will be more to come ((Ps laugh)).

M: A person that is taking treatment, okay if we look at the back what do these numbers say ((M asks for numbers from Ps)) so a person who is on treatment, who attends the clinic, if you look at these pictures, what should their weight be?

M2: Let’s carry them in this manner so that everyone can see which one and which one is there; let’s have them facing this side so that we can all see them?

P001: I think, I have 6 on me.

M: Why?

P001: I think he is still well build and has a normal body.

M2: What do others say?

P005: I am also choosing 6.

P004: I also think that number 6 is fit.

P007: I also think that number 6 is a bit fresh.

M: My goodness, no one chooses another number? ((They all speak at once))

P005: This one has a big tummy.

P003: This one looks like this one.

M: Which number is that one?

P006: This one is number 9.

M: So you are also saying 6?

P004: He is normal.

M: It’s his shape? If you choose a shape, which shape do you think is the best here?

P002: It’s 5.

M: Why do you say 5?

P002: I see 5 as being normal, is of moderate weight, is not thin.

M2: So we like number 5’s shape, which shapes do others like?

P005: I like s number 6’s shape.

M2: Why do you like number 6’s shape?

P005: The thing is, there is nowhere where she seems slim, I don’t like number 5’s legs ((ye, they are thin)) they agree with P)).

P001: I get confused but I see 5 being more energetic than here.

M: One said he is normal, you are now saying that he is energetic. (.) Is there anyone else who likes another body shape other than that of 5 and 6? (.) ((M brings the women silhouettes and distributes them to the group)) why are you startled when you see number 8? ((P laughs)) what are we saying then about ours as women? Actually, we are trying to find out if there is a weight which we say is better for men, it’s better for women or something like that? Number 2 nods his head.

M2: So for the females what are we saying? Please bring back the one for males then.

M: Let’s not look at the numbers; let’s look at the type of a body weight and a body shape that we like, for women now.

P004: I like this one.

M: He likes this one, number 4, this one.

M2: Why do you say you like number 4?

P004: I like her because she looks like she has a good body and well-shaped.

P006: I like number 4, number 4 is well shaped and the manner in which she is shaped shows health.

P002: I also like number; she is well shaped and likeable.

P005: I like the one that this lady has on her hands, which is number 3

M: Why do you say you like number 3?

P005: the thing is, this is a manner in which I would like my body to be shaped as well ((Ps laugh)).

M: Number 3, you wanted to say something?

P003: I like this one, number 5.

M2: Number 5 because?

P003: I see her having a good shape, I think she has proportional body parts, from the top to the bottom; there is nowhere where I can say she is smaller than other parts.

P001: For me since it’s very hard for ladies because the factors that contributes to their weight are the ones that we see especially physically because we can find that a person has big breasts whereas she has a small body so o feel that if a person is comfortable in her own skin, I think that’s the right way for them because here we are putting them in categories, you see so it seems as if it’s a contest if she feels energetic and healthy and then that’s the way to go so I don’t think there is a specific category that we have to put them. So you feel comfortable by being slender or thin just you, I think you must see to it that you live a much better life so I am not able to allocate it to any category.

M2: Okay, that’s for the weight. The shape, which shape do we like? (.)

P004: Well the shape I will go for that.

M: You are choosing number 4.

M2: Why that shape?

P001: She can wear anything, yes as can be expected on the dress code according to where she belongs.

M2: Okay, others? (.)

M: Which shape do we prefer?

P006: It’s number 4.

M: which other shapes do people like. There is no way that we can all choose number 4?

P003: I still continue with number 5.

M: What do you like in number 5’s body shape?

P003: I still like the ways she is.

P007: I like number 5’s body as well, she is not fat or small, she is of moderate weight.

M: what changes can we expect if the treatment is going well and when it’s not going well?

P005: It means it can change.

M2: Change, how?

P005: You can be big on the top, you can develop breasts, you can develop a stomach, you can no longer have bums, hips and the legs, it can be something else.

M2: Will the treatment be going well or will it not be going well?

P005: You are asking if I took it well?

M2: When the treatment is going well or not?

P005: It means it was not going well because when I complained, they changed my regimen so that I could regain my shape even though I have not regained it completely, since my breast is still big, it was never restored to its original size, yes.

M2: Okay, what do others say?

P003: I started by gaining, I was okay, afterwards I lost weight, these legs became very thin at the bottom so it means they (ARVs) were not okay after that they changed and said, it’s late you will no longer go back to your normal condition, indeed even now I did not regain it, I can just see the legs, I even developed constricted veins, I am now developing thin legs, I was not like that.

P007: On this thing of changing people, the treatment changed me also for instance I was someone with big bums, but I just got worse, I developed big breast, here on the thighs, I no longer had hips, it was as if something has just been put on me, you see even when you have a straight cut skirt…, I no longer put on the straight cut because since it just looks plain, they changed the medication and said, you have been taking it for a long time, it will also take time before it becomes better, they do not promise, you see, the thin legs also, they look dry and very thin, you see.

P004: When I started treatment I had lost a lot of weight, but now I can see that I am going back to my normal shape since I am someone with big bums you see but then the problem is that it’s making me to have a big stomach, I now have a big stomach, something that I didn’t have.

P002: Since I started treatment I gained weight, I had lost weight, I am still where I used to be, I did not gain more weight, I did not lose it either.

M2: It’s going well?

P002: It’s going well and I have never changed the treatment since I started.

M2: Okay, what do others say? The changes if the treatment is going well?

P001: I regained mine but like since I have not yet started buying new clothes, I am still okay, I feel that I am getting stronger each time.

M2: Okay, others? (.) What influences your thinking around the ideal shape and weight, is it culture, is it the media, is it your family, why do you think number 4 for example looks right?

P004: I think that it depends on how you behave yourself, how you eat, do you eat healthy food like number 4, the way her shape is, she is okay, she is fit but not fat, she is not thin, I think they behave…, they eat well maybe they are even jogging.

P002: I think that number 4 exercises, the manner in which she looks good and she eats healthy food.

M2: So that which we say that this person, number 4 is okay where do we see that a person should be like this?

P001: I think the environment and the culture plays a lot, obviously we grew up knowing that a person with a big body is healthy and stuff like that but that’s not true you see where we are sometimes you will see when they will point at a person who is HIV positive even when they say they are HIV positive, they will say whilst you look this good, so we take the thinking of an ideal person and make it seem as if it’s the person who is okay whereas even the environment that we stay at makes us to make the decisions that we make.

M2: It is culture and the community.

P001: Yes, it’s the culture and the community because those are the things that we respect so we think that’s the ideal person whereas they are not.

M2: What do others say?

M: Where did we learn or got to know these things that so and so looks fit, so and so looks healthy, energetic? Where do we get those things, what informs our thinking? There is culture, community, TV and media does it ever happen that the media or on the TV or the newspapers also to a certain extent make us to think that a person should look like number 4?

P003: The radio, I like to listen to the radio and the TV if when I switch it on, there are discussing something that I like, I listen and learn a lot, even on the TV, others ask what about this case, a person will say that they have this type of a problem, that it when I manage to learn that there is this type of a thing.

M: What do others say? The family…, Does it ever happen that the family that we grow up in makes us to think that a right body shape is that one or a body weight should be like that, it should not go beyond that or it should not be under that?

P003: In the family it’s very difficult, they will say oh you think that someone has AIDS so to speak which means even when you get sick it means even when you ask them if they went to the clinic, they will say they did go, I did test for everything and all is okay, whilst you can see as we are aware of the signs and symptoms, so you fail until you ask yourself if they did go and test because there is nothing else that can make you see that she did do it but you can see them lose weight, coughing, they cough and it gets very difficult. Us since we are carers they will say even my aunt can see that I am this and that, I will then say, please go to the clinic, you must do what they will tell you to do, the following day, I ask, did you go to the clinic they will say they did, what did they say, they took everything and said I don’t have anything whilst they are still sick so that’s why this problem continues, I just get sad because other people outside listen, I will people out there and people in my family do not want my help I just get a real problem even now I have a very big problem, you need to start at home before you go out there but it does not work like that.

M: What about the health services, can in happen that in terms of the ideal body shape and ideal body weight we learnt some things from the health care services, for instance the clinics, the hospitals, nurses and stuff like that?

P004: I think before you take the treatment, you attend classes for three days they tell us about those things, uhm.

P007: To support this thing that the lady is saying, they enrol us on classes such that should you not attend on the date allocated to you, you will start attending classes all over again.

M: Okay, all right, according to your opinions perhaps what is it that can make a person who is HIV positive lose weight?

P002: Another thing that can make a person with HIV to lose weight is to not comply with her treatment. Number 2, and that she is not happy at home, there are discussions that are not pleasant, you will then find that they are not happy and that thing on its own sometimes make a person to not eat well. If they have many things, sometimes they are not working, they are always thinking about finding work that *eish* if they can get a job, they can do this and that, I think that that causes a person to lose weight.

M: What do others say?

P003: To second my brother, it’s not being treated well at home and not eating healthy, and unemployment make a person to think a lot and another thing that makes people to lose weight is not using protection when they are having sex and the viral load in the body goes up.

P004: And drinking alcohol, you should not drink alcohol as we she has already said we need to use protection.

M: Uhum, what do others say?

P005: I agree with the lady here, it is not using a condom, alcohol and not taking the treatment accordingly.

M: The guy next to you is smiling, do you want to talk, do you want to say something?

P006: Indeed, I agree with them, stress, the fact that there are things that you do not like at home, you see or if you are sick you are stressed out but if you are not working you are always under stress, you see, there are lot of things but you cannot get them, you see, yes.

M: The issue of being stressed out at home keeps coming up, things not going well at home. Is there perhaps someone who can just make an example generally, if things are not going well at home what is actually going on at home even though you are not talking about yourself as such maybe just talking in general, those things that cannot go well at home that can make us to lose weight, it’s this that and that.

P005: I will talk about that which happened to me and my family, when I came to test, they asked who do I trust that I could tell, I told my sister, this thing spread to the whole family, she was spreading it. The thing is they drink and I do not, when they are drunk, they insult me with this thing, I have children, I have a son who has a child, he sometimes visit, he does not stay there, he is also insulted with this thing, I am always insulted with that thing to say there is AIDS in that house, once you get flu or someone gets sick, actually AIDS is rife in that house, it’s AIDS only there, you see, those type of things, even when you get a job, they will go and stop it, don’t give her work we want her to suffer until she dies, the problem is that they don’t like me, I will say that because they do utter that word. They do not like me, they say I will spread AIDS; they even locked me out of the toilet such that I used my neighbours due to my sickness. Even when I explain that you cannot get HIV by sitting in a seat or by drinking by a cup that I use or using the spoon that I used, it’s transmitted through blood, no, they don’t understand that, the thing is I am talking about something that is happening to me, we stay in one yard and we are talking due to the issue of the virus.

M: So this stigma and the discrimination within the family, the lady has explained the condition. Okay, so we had alcohol abuse and not taking medication, and not being treated well at home as things that make us to lose weight, we also had things pertaining behaviour those of not using protection. Is there anything else which we think makes people that are HIV positive lose weight?

P005: According to what I learnt, from my own experience, they told me to not use an enema, to not induce vomiting so when I had a stomach problem of having a bloated stomach and not being able to defecate, I used an enema, when I came for my 3 months visit, I found out that I lost a lot of weight, I then started here, I stopped doing those things until I was okay

M: Okay so you learnt from experience there, they said I must not do this, I should try and not do it maybe they know why, okay, is there anything else that we think makes people living with the virus to lose weight?

P001: Acceptance, if you are not feeling that way, it closes everything such that you even lose appetite even though you can take B-Co for it but acceptance, the appetite is very important and it starts with you but if it goes to other people, it’s very painful they are no longer treating you in a manner that they used to treat you before. So I think that we lose weight because we want to be accepted in a manner that we are, yes and then guilt within you perhaps because if you are stressed out that thinks eat you from the inside until you cough it out.

M: Okay, all right, we come across people most of the time saying stress makes a person to lose weight, what is it in stress actually that makes us to lose weight? How will having stress make me lose weight?

P003: I can say that if you have stress, isn’t it you also do not want to eat, you cannot eat so that makes losing weight a reality.

P007: Not sleeping when you are stressed out, you do not eat also so if you do not sleep, you will lose weight in that way.

M: What do we think happens, to make people living with the virus to report that their weight changed but the scale that they ask them to stand at there at the clinic does not indicate any change. What can make me to go to the clinic as xxxx (facilitator’s name) and tell the sister that I have lost weight but when the sister asks me to stand there ((on the scale)) and maybe checking my records, you find that I did not lose weight but I say I lost it, what could have made me to come to the conclusion that I lost weight?

P003: Sometimes isn’t it when you walk, you can perhaps feel that you are light, maybe a person can think that they have lost weight, and just feel that they are weak.

M: Feeling weak okay, we once mentioned that earlier on.

P007: It’s the manner in which we think, it’s thinking that you lost weight, it’s how you think.

M: It’s your thoughts if they are along the lines that you lost weight.

P001: I think even the negative thoughts or negative comments from the people; they can make you doubt even the treatment ‘cause you think these people know you better than yourself whereas you are the one who knows. People can break you down if you also do not have a high belief on what you are doing.

M: Uhm what do you think weight gain means to people who have started taking ARVs or getting HIV care? I think we once mentioned it earlier on, what does weight gain actually mean to a person who is taking ARVs or who is on ARV care and who complies what does that mean?

P001: I think that it means that the treatment is working but they need to keep monitoring it so that they do not become obese or you see but I think the treatment shows it is in you and that it has started working and then they will notice areas that they will need to improve on you, what you need to get and what you should do more.

P004: It means that the treatment is working because there is a lady that I know, she is my neighbour, she has never taken the treatment, I do not know how she does it but she is HIV positive so she comes and test here at the clinic so last time when she came to test, they told her that she is now HIV negative but it’s not that since she is negative this thing has been cured, they say that is when it is…, since I once asked professional nurses at the class about this thing, I have a never who says she tested, she just tested negative? They said, yes, it does happen that is when it is hiding somewhere, it’s not that it has been cured, it is hiding somewhere maybe there is something that she is doing, maybe she has imbiza or something that she is drinking, we do not know.

M: So in other words you are saying that imbiza sometimes makes you gain weight?

P004: She gained a lot of weight; I do not know what she is taking.

M: Why do you say she has HIV?

P004: She showed me the results, she is my neighbour.

M: She also showed you when they had turned negative?

P004: Yes.

M: Was it negative or undetectable?

P004: Yes, yes, they say it happens, maybe she is using the treatment somewhere since she goes somewhere so when I asked here, they said it happens, it’s not that it’s cured from the body but maybe that is when]

M2: [It’s just dormant.

P004: Yes.

P007: To take over from my sister, I don’t think that she was negative ‘cause I once took bloods and the results were that it’s undetectable but it does not mean that it’s not in your blood, it’s there, it’s just that it’s too low, do you understand, not that it’s not there, it is indeed there, that is what they said to me but I am not sure about her case but that is what they said in my case.

P001: That’s why they say you should not take performance enhancers into your system and stuff, that is what makes it to be not detectable, you find that after some time the after sometime the viral load is more higher, you see so things like those that give you energy are not needed, they are able to hide it so that it’s able to hide itself in them, that is why when you go to a weighing scale they tell you that you weight is very low and then you feel very energetic whereas you are drinking Energade and so on, so that’s why they just say substances are a problem, they make examples with those things, the enhancers, those herbal things they will say do not eat things that will give you energy and stuff so that the ARVs will give then the true condition of the viral load, so the enhancers are just defeating the purpose of you taking the ARVs ‘cause at the end it’s not good for you, yes.

M: Okay, we are learning here. What does weight gain mean to a person who is taking ARVs?

P003: So if they take it as suggested when she is gaining weight, it means that the viral load has dropped, her CD 4 count has increased.

M: What do you think is the best way to ask people living with HIV if they have lost or gained weight?

P001: Can I ask something you mean a manner in which you can ask a person who has already been infected?

M: Yes, so that we can get an accurate answer?

P001: I think if a person is-honest, you just ask them how the -ARVs are on them, that’s where a person will tell you that hey I do not sleep, I do this and that, I think you can get a more concrete answer than if you ask them about their weight ‘cause the weight alone, I don’t think it’s accurate because it has to be measured against the previous one often. So if they can tell you what they are experiencing in their bodies and then one will tell you that if I take these tablets I have night mares, I do not sleep, I sweat, if you say you are sweat, they will want to test you for TB and stuff and you will find that you don’t have those things, it’s just the tablets reacting in their own ways so if you ask a straight question, since you started taking the treatment how do you feel, that type of thing. That’s the question that they ask you. In my case my hands were swollen and the feet a little bit I could walk properly but I tried but then at the end I got better because the system got used to it.

M: Okay, what do others say? Number 1 says we should ask how they are on a person. We want to know how we can get an answer or how can we ask in a better manner if people are losing or gaining weight?

P003: I think that it’s that way, as my brother is saying asking them how they feel, they will tell you that it’s doing this thing, they are not able to eat, they have lost weight, they will mention their side effects since not all of us are the same anyway, they will make another person to have a running stomach, all those things.

M: Okay, all right, what do we understand perhaps if a person is asking you if you have you lost more than a dress or a trouser size unintentionally in the last six months ((M translates this into Zulu)) what do we understand by that? I have three pants here, amongst men, can I have a volunteer that will show me how a person lose more than trouser size, we are asking for a person to show us what that means? If we can get someone who will volunteer for us and say no my sister they mean this and that, here are our pants.

P002: If a person lost ((weight))?

M: If perhaps you visit the clinic and you are asked questions about weight, a nurse asks you if you have lost more than one dress size, what do they mean? What is our understanding, if there is anything that we understand at all?

P001: I think that it’s from a size 32 down maybe to 28, I think so.

M: It’s from-32 to 28, can you be able to show that using these pants and mention these sizes that we have?

P001: I am looking at them here, -32- it’s this one on the side, can you see it, and then 28 is the last one, this one is small, let me take it out. I think I am trying to say that in terms of maybe losing weight but the length stays the same, so I think you move from here to here.

M: My tape recorder will not record from here to here.

P001: It’s 32 to 28 for me, you see?

M: Okay, do we agree with what number 1 says?

Ps: Yes ((a resounding yes from Ps)).

M: Do we all understand it in that manner?

Ps: Yes.

M: We will not have a problem if we go to the clinic when they ask us have you lost more than one dress size?

Ps: No.

M: Okay all right, here are those for females, because this group is mixed, here are those for women then, we have skirts here, this one is size 38, -size 36 and size 34, if a person says they have lost more than one dress…, a skirt’s size, if we want to illustrate with these skirts, how can we indicate you moved from where to where?

P004: You moved from38 to34.

M: You understand that in that manner?

Ps: Yes.

M: Okay, thank you. Okay all right.

M2: Okay, if a person who is living with HIV loses weight how do people respond to them? Let’s talk about the spouse, your partner, how do they respond when you lose weight?

(.)

M: In general in the community, as we are here in the focus group, don’t talk about yourselves only, you can also talk about others who you know that they are HIV positive and just the things that you sometimes hear, those which they tell you when you are together for instance, what did their partner say when they saw them lose lot of weight?

P004: My partner kept telling me that I should go to the clinic and get tested, I can just say that he was not honest with me , I used to see him going to the bedroom to take the medication, I did not understand, my sister is a professional nurse, I asked my sister and said, you know this one every evening at 9 he takes a tablet but according to what I have heard, it’s called ARVs, he takes them at the same time every day, I suspect that this person is taking a tablet but he did not tell me, you see I realised that it was going on and on, I could notice that my body was changing, I was changing non-stop, I was losing weight, getting weak but he was showing me in his own way you see by not hiding it when he was taking it, but he did not tell me straight you see *((name withheld for confidentiality reasons)) she said to me I must give her the name of the tablets, I told her and she said, yes, those are the ones, so that is how I approached to say how my man, why are you not telling me, he said he was afraid but the manner in which I was telling you was when I was asking you to go to the clinic to get tested since he used to encourage me to go for a test.

M: He used to make -comments about your weight loss?

P004: He asked me if I can see that I was losing weight and that my face was changing, I was attached by pimples and all that stuff, yes.

M: Okay, what about others, what do your partners say or what do we sometimes hear people say their partners say when they lose weight?

P003: My sister’s child is taking-treatment, she went and got tested when she realised that she was not okay and then her husband did not, it’s these people in polygamous relationship, so she came to me and said she had come to disclose, she said she went to the clinic and got tested and that she had been attending classes, the time had come for her to take treatment, I have a problem aunty, I am asking my partner to go and he does not want to. In the class that I am attending they are teaching us that the treatment will not go well since I will take the medication and I should ask him to use a condom, I am asking her, if you tell him that you are both needed at the clinic what does he say, she says he does not want to. So she attended the classes late, I asked her to persuade him again, she said he says she asks for money to attend the clinic and that she should tell him if she has HIV so that he can also inform his family that she has infected him with AIDS. So that was difficult since she realised that if she informs him that she has HIV, he will ask for a family meeting and say his wives have infected him. So that was a big problem, there is no other way out instead of leaving him with the children, she will opt to taking it, I went and signed for her but now the husband is sick and the other wife is not taking the treatment, she is sick, she told me that we need to meet her and advise her to visit the clinic which we did, she promised to go, she did go to the clinic and I found her in a queue, when I got there she left when we went to do a follow up the following day, she said when she got there counsellors were not available , the clinic said there were going to be available the following day, she did not go back there until today. She went out and she did not go back to the clinic for testing, she has lost a lot of weight and my sister’s daughter is worried since she knows the condition of her sickness, she has developed shingles and has said, she is being bewitched and all that. So that is just a problem that I am facing and the wife on the side does not know that that she has this thing.

M: What do our spouses usually say when they see us lose weight?

P007: My husband has been very supportive to such an extent that as I am taking the tablets sometimes I sleep early he would wake me up and say *((name withheld for confidentiality reasons)) do not forget to take your tablets, it’s after 8 now, and kids at home also they are very supportive so that thing makes you to be okay.

M: So others get -support, even before others disclose, they are afraid that they will be regarded as people who brought the virus, what do others say?

P002: The situation is the same with me also, my wife supports me a lot, we take them together, I take 9 tablets during bedtime so sometimes I sleep early so they wake me up and say no, do not forget to take the tablets, yes, they also wake me up when that time arrives, they wake me up and say dad here are the tablets, take your tablets.

M: So we get support most of the time, are there people who can say they did not get support from their partners or spouses or after they had lost weight as a result of HIV?

P003: I received -support from my husband, how it started is, our child got sick and that was before the treatment, I don’t know, they had tested her without telling us or what had happened, after that I fell pregnant, in 96, I went and got tested that is where they told me, so when I came back on that day, they told me and when I arrived home I told him, he was not angry, he accepted it so we were okay even before the treatment so what was bad is that he accepted he did not fight but he was promiscuous so this is what came between us, you see if we are already aware of our status, he goes and finds another one, who are we going to become, we did not get along, we discussed that we were going to lose lives for nothing. I left him so he was promiscuous since then, he didn’t have anyone to look after him, he continued, when I realised that I was not well, I started taking the treatment, I attended classes before I could get support, I took him as a supporter, on the following week he started, we were staying apart, he complied on that year, on that year we used to remind each other, phoning and telling each other it’s time, wake up and take your tablets at the same time]

M: [So it seems as if there was support initially when you were still together?

P003: There was, when he started staying on his own, he defaulted on treatment, he stopped taking it, I kept encouraging him, you should take the treatment, when he had other girlfriends, it was as if I am disturbing him, I then stopped and he ended up dying.

M: Oh my goodness, that is sad news, it seems we do get support from our partners.

Ps: Uhm.

P006: Yes.

M: What about children, what happens?

M2: They said children are supportive.

M: I think number 2 is the only person who spoke.

M2: They remind them to take medication.

P004: My children are aware.

M: Extended, the wider family, your aunts and others?

P007: My sisters are very supportive because they were the first people whom I informed before I informed my husband, I asked for an advice from them that I had tested positive I am afraid to inform my husband, they said no, tell him, they were the first people and my sisters are supportive, it’s like if something made me angry, the family as a whole, she will send me a message saying I must not stress a lot, you know the situation that you are at, you should not have a lot of stress. So they are very supportive.

M: Okay, what do others say?

P004: The same with me also, my sister is the one whom I informed first when I came back from the test.

M: Okay, the lady received support from the family. Are there perhaps those who say they did not get support from the extended family, are there those who blamed you and called you names?

P005: I did not get support from the family; I only received support from my children.

M: Okay you received -support from the children, are there people who can say yes about the extended family, I think it’s the lady only who touched on that point earlier on, what do others say in terms of extended family?

P006: if I were to tell the truth, in my family a person who knows is the one that I stay with, my mother and my child. But my aunt and my sisters as I stay with them at home as a family, none of them is aware, you see, the thing is I stay at a back room, there are things that they say, that we sometimes discuss, there are also things that they do not want us to discuss with me now it was difficult to explain this situation that I am at, you see.

M: You did not say, so you will not know what their reaction would have been.

P003: My extended family is also not aware

M: What about friends?

P001: I did not tell them, I have this one friend , my best friend, he is a person that I am still gaining strength to tell, the rest, I feel they are not matured enough for me to tell because they will just keep on spreading the wrong information about me which I think I will not be able to handle and then in the family only the elders are aware, the thing is in my family I am the one who plays a significant role, you see so it’s a bit tough to tell my nephews that I am supporting financially and otherwise so I am trying to be strong for them other family members that are there even my uncles have given up in life so I seem like a father of the household whereas I am just a grandchild, if something wrong happens they will not tell anyone, they will wait until I come back from work. Even now when I go back home they will tell me that my uncle did this and that, you see those type of things. So I told the responsible elders, I did not tell others because I think they will lose direction more than they have because I am the one when there is something I come up with something constructive and they are able to listen.

P005: I did not tell any of my friends, I have one friend, it’s only now that I told her, she found out that I have it when she was at home during the incident where they were insulting me so when she started getting sick, she asked me help her and guide her on what to do first. I asked her by saying she must go to the clinic so that she can also know what to do, they were insulting me at home but when she started getting sick I helped her.

M: Okay, you were her supporter?

P005: Yes.

M: Do we have other people who want to say something about friends?

P007: I told my friend, I cannot say it’s a person that I met here, she is my friend, she stays nearby, I told my friend from the xxxx (another province), so she supports me, even now she calls me, she does not stay with me, she stays in the same street, she is in xxxxx (area), she is the one whom I informed, other friends and neighbours I did not tell but her.

M: Perhaps if we do not talk about ourselves but talking about people who are positive in general, what kind of reaction do they get perhaps from friends if they have lost weight as a result of HIV, just that which we have heard, which we can say we are lucky it has not yet happened to us?

P004: Maybe my friends talk when I am not there but so far they are still supportive to me since you can never know if you are not there, you see, but when I am there, we actually talk about it a lot and also encourage one another.

M: Okay, the-community then, how does it respond to a person with a virus especially once they have lost weight?

P001: The community, I think old people have sympathy, we are the-youth, we have not experienced other things, you see if you have not experienced something, it’s difficult to be supportive to another person so old people even though they are not HIV positive maybe they have had a child or someone who has it, so old people are -supportive, that is what makes them to say do not forget to take the tablets, if you have a child, do not forget that you have to work for the upbringing of your child and stuff like that, you will also become okay. So the youth, I don’t think they are still supportive and you find that that’s why the youth finds out the hard way when they test positive themselves, you will find that if they sympathised with another person they would have been able to avoid other things you see, I think that the youth has a problem.

M: Okay, what do others say about the-community?

P003: It used to shock people previously but the way I see it, where I stay , it’s something you see, it’s like you are suffering from a common cold, it’s no longer the case that yoh you have AIDS and all that, you see. No one laughs at the other.

M: What about health care workers then like doctors, nurses, counsellors here at the clinic, how do they treat us when we visit the clinic after having lost weight as a result of HIV/AIDS?

P002: For instance last year, I noticed that they got very worried, yes, since I had lost weight, they said no, they said there are things that are not right that I need to fix maybe I am not using a condom or whatever, I said no, the problem is the family, at home, there are things that are stressing me out, I do not make a mistake with the tablets, I make sure that I take them on time even when I am away, I carry it along, even when I away from home until late, I have them in the car. So when it’s time, I open where I keep my tablets and take them.

M: Okay, so you are saying health care workers get concerned.

P002: Yes, a lot.

M: I agree with her.

Ps: Yes, they even scold at you when necessary.

P003: They regard you as their family, they get concerned.

M: Uhm okay, all right, so we as people who are living with the virus, how do we feel about these responses, I think we have indicated that we get more support than anything else and then we indicated that within the youth category that is where we could get concerned but the fact is that we have been getting a lot of-support most of the time, how does that make us feel? How are we feeling?

P002: No, it’s very much okay now, I can now say that am feeling good since we now know the situation that we are at as compared to previously. We didn’t know what was happening previously, as the lady says that this thing was frightening but now it’s similar to flue because what I have noticed is that even if a person does not have a virus, they can die before me, I can see that it means we are the same, when my time to die comes I will die from this thing, the time would have come for me to die, so I can say from my side, I am very much okay, I have told myself that I am a person who has to live like this until God keeps ((takes)) me.

M: As people for instance -number 5, people who experienced that which is similar to that of number 5, if a perhaps a person has a problem at home, how do we feel when we are treated in that manner as a result of living with the virus, I am not directing this question to number 5 only?

P001: I think when many people feel like that it is when they get suicidal thoughts or getting reckless, they will then say, I will not die alone, I will spread it or something like that, yes, you will find that a person ends up feeling like that, that is why the department has done this thing of having researchers like yourselves and these sessions where we talk, you see once you have accepted yourself at least you are going to be strong because you see once you start poisoning your mind, you will make it worse, if you are trying to control the disease in me whereas I am having other thoughts, I will infect other people so that mentality is tackled in support groups and the fact that support is important, yes.

M: So you mean that it makes us to be hopeless and helpless to an extent of wanting to kill ourselves and stuff like that. Okay, what do we think the following people will respond if an HIV positive person gains-weight, earlier on, we were talking about a person who has lost weight, now we are talking about a person who is HIV positive but who is gaining weight. If perhaps we start with a spouse, your partner, your husband, your wife, or the mother of the children or the father of the children? We are gaining now, how do people often perceive us? How do they usually respond when we gain weight?

P001: Gaining according to other people is perceived as a sign of fitness so they see all the best things in you to say that life continues so it’s like good results if you are taking ARVs instead of thinking that HIV is a -death sentence to say yes, he is about to die irrespective of what, so weight gain in a way is a positive thing.

M: Okay others see it in a positive way, what do others say, how do our partners usually perceive us when we gain weight?

P002: Yes, no, as the gentleman say, I trust that they say that, that actually that person is healthy, yes, those that know that no, you are positive, they are certain that you are using the treatment in a good way.

P006: I want to agree with them that if you gain they will be happy that things are going well. M: Life is good, okay, how do children usually respond?

P006: They are also happy also.

M: The extended family, how do they usually respond when they see us gain weight when we have HIV?

P003: The family becomes happy also.

M: They become -happy.

P004: They become happy and say actually you are beautiful this year, this year is good to you, it’s not the same as that one, no you had lost a bit then.

M: So that is supportive and positive. Did it ever happen that they have negative -comments?

P003: No.

P007: it sometimes happen that you find that when you get there they say you have gained weight it’s like you are taking tablets, you see, so you gained weight because of the tablets.

M: Do they mention the type of tablets or those for contraception?

P007: No, they do not mention that but you also know the type of tablets, they say this one gains weight it means they are taking tablets. For instance, we had a function on Sunday so one of our cousins pitched, they said, why are you this fat, are you taking treatment you know so you cannot say that they are happy. Our weight gain, when we are happy, they are perhaps seeing that no, they are not fat they tablets have made them swollen.

M: What about friends, how do they respond when they see us just gaining weight?

P005: My friend is happy, she usually says, it seems as if you are gaining weight, she will then say it means I am okay inside my heart. The family will just tell you that you are fat, one will say, why are you fat, full of the ARV acid, you are going to burst, you see, I just tell her that she must ignore them as they are not her God. I have become used to it, as long as I get support from my friend and my girl children that I stay with, I do not have a problem.

M: Guys, did anyone of you amongst us receive I a negative response from a friend after gaining weight (.)

The community? The community what do they usually say when they see us gaining weight?

P007: Maybe they do not tell us, you see, they will not tell us, yes, they will discuss it out there, you will not know what they are saying about you or about another person who is HIV positive.

M: So there is gossip, what about the-health care workers for instance the doctors, nurses, counsellors, how do they respond?

P004: Last time I was here, a professional nurse that was attending to me when I had come for the-treatment commended me a lot, she said I am commending you, you are healthy now, you have gained weight, he asked me how I feel, I told her and I did admit that I was feeling okay.

M: Okay, positive comment from the nurses, are there instances where perhaps we receive negative comments from the health care workers?

Ps: No.

M2: How do you feel about comments for weight gain?

P003: We feel good, they encourage us.

M: It’s encouraging?

P003: Uhm.

M2: What do others say?

P002: That is so, we feel good, that is a good thing indeed.

M2: Do you think that a shape of a person with HIV changes?

Ps: It changes.

M2: How does it change?

P004: As he mentioned that if you did not have breast before, you start having breast.

M: What else?

P004: You start having a flat bum, your legs become thin, you develop a big tummy.

P001: The skin tone.

M2: What about the skin tone?

P003: Sometimes it will look grey.

P002: And a rash, you just develop things that do not look good.

P001: The infections, for instance skin infection. Your skin is not good, you see, yes.

M2: Is it the same for women and men?

P002: Yes, it is the same.

M2: All the changes are the same?

P004: They are not the same.

M: In most instances, how are women’s changes like?

P003: Women will have thin legs, big tummy]

M: [That is for women only?

P002: It happens to men also.

P001: And then there are those which are the same for all of them, right, which is if people drink a lot, they will have a big tummy and the colour change, irrespective of whether a person is a male or a female. If you drink, there is this thing that will indicate that this person drinks; I think they are taking this treatment.

M: What is that?

P001: It’s a skin tone; it changes to pink or grey even if you apply something on it.

M: Or the face of a person that drinks a lot?

P002: It’s not the face of a person that drinks a lot, it’s greyish, it’s pale grey, you look as if you did not apply anything.

M: What do we think causes the changes that we have mentioned?

P001: It’s the lifestyle, the things that we put into our system.

M: If you say things that we put in, what are you referring to??

P001: Food, substances, and taking basically taking care of ourselves, the way we treat ourselves, that is what brings those changes.

M: Are there instances where we say, this thing was caused by HIV. For instance, when we started our discussion, some people said they came here because they were noticing this and that, now I want us to try and find out what it is that we saw as changes but which we think were caused by HIV on its own?

P001: I think HIV does not cause anything, it takes out what you initially had when you were born, so it -capitalises on your weakness, if you had asthma from long ago, HIV will cause a problem in your chest, just anything you were born with, HIV exposes those first, yes.

M: What do others say?

P003: I said to myself, I used to be bloated and had problems when I go to the toilet and sharp pains on my stomach, my stomach was always full, with gas and sharp pains, I went to the doctor and the doctor said I should buy and eat High Fibre Corn Flakes to remedy the situation, they are expensive and I am not working, I need to buy it and I do not have a fridge to put it in but when I went for a test and started taking treatment all these things of sharp pains subsided. I used to be frustrated, they asked me to not eat beans things that I used to eat without problems, I used to like beans, I used to eat a cabbage.

M: So your problem is that you used to be bloated, we are trying to find out the changes you used to have before we started taking ARVs, the changes in our bodies, those which we can say; those ones were caused by HIV?

P002: No, when I came to start taking ARVs I used to feel that no, I did not have strength the way I know myself to be but I used to eat anything, I was able to east anything, I was okay, I did not have power, feeling that no, man I am not okay, you see I used to sweat when I was sleeping at night, you see that thing, then I came and got tested.

M: Do we have other people who want to say something about-HIV?

P004: I used to feel drowsy, I was always drowsy, I did not know what that drowsiness was for, you will find that I did not have high blood pressure, eventually my eyes could not see clearly when I was reading, but when I started taking ARVs all that subsided, I could read with ease sometimes I do put on the spectacles and sometimes I don’t, I think it was the virus.

M: Is there a person who can say their body shape was changing, changed by HIV, the body shape now, the shape of a body, where a person can say, this started before I could take ARVs? Is there?

Ps: No.

M: Okay, all right, what else, we have mentioned some of the things that thought were caused by ARVs, and we said these changes are called by HIV right? We attended classes before we started taking treatment, did they explain to us this thing called lipodystrophy, the dislocation of fats in the body where maybe fats from a person’s face, a person will not have fats on their faces or maybe the buffalo hump, where they will grow a hump at the back of the neck or the stomach or the stomach will grow or perhaps fats from the bums, the arms and the legs as we have explained some as things that have happened to us, were we aware that there is something called lipodystrophy which is caused by ARVs?

Ps: No, we do not know it.

M: We were not aware?

Ps: No.

M: Okay, what you have explained are some of the things that are caused by ARVs, the dislocation of fats from one place to the other, for instance from the hips to another area. What does the community usually say when they see a person change shape as a result of ARVs lipodystrophy, a person who used not to have a stomach but whom you see the stomach growing speedily?

P003: They get shocked especially when they see my legs. I used to have legs that were proportional to my body, I had the same as this lady ((says P pointing at M2’s legs)). So if you are arguing with others, they will insult you and call you thin legs, these veins. M: So some of those are legs, for instance, let’s start with the spouses, how do they usually respond when they see our legs changing, we do not necessarily need to talk about ourselves, what did my partner say perhaps what do other people’s spouses say, other people that are living with the virus and who are taking ARVs.

P004: They are aware that you are taking the treatment?

M: Their spouses for instance, is aware that you are taking -treatment and they are aware that this change has been brought about ARVs, how do they usually respond, how do they take that?

P007: He comments and says that I have extra muscles and he will not say exactly that this is what the tablets are doing. I do not know exactly how he says it but that is when he is teasing me at that time, he will say my wife you have the extra fats, you see, I do not know where exactly that comes from.

M: Okay, sometimes you tease each other about it, what do others say? (.) What do others say when they see these changes. (.)

M2: The extended family?

(.)

M: Your aunts, your uncles?

P001: Ei sometimes they perceive it as success, if you are employed, they will think that you earn a good salary, you are living a good life, you see, they do not look at it along the lines of whether you are being healthy or not, they simply say, you also bought a house you are living a good life, look at your weight, you are the only one who knows that there is something that is helping me to live, so they do not look at it along the lines of being healthy, they look at it in terms of success those who know, will then comment by saying hey this thing is helping you but I don’t know if they say it in a manner of mocking you or they are encouraging you, you see, yes.

M: Friends?

P004: My friend once commented when I had one of my pants , she said what’s going on my friend, these pants are tight on you now, you have gained weight, as the lady said, you now have extra fats on waist area something that you didn’t have previously, it means things are going well.

M: Oh she also sees it in a positive light, what do friends say, we are finishing up now, we are just wrapping up (.) What does the -community say when we have these changes in our bodies or if we know in the area that someone is HIV positive and taking ARVs presents with these changes in the body?

P: The -community gossips about them.

M: What does it say when it gossips about her?

P003: I hear them say, it’s these tablets, can you see how you look, you are taking the tablets.

P001: I see it encouraging and also those that are aware but who are afraid to go and test, yes, so it encourages them in a way even though they have it, they will pretend as if they are laughing at you whereas you are giving them an idea that they also need to visit the clinic and look after their health.

M: Okay, what about the health care workers, the nurses and the doctors and the counsellors at the clinic when we are presenting with the body shapes that are no longer the same as the previous ones, how do they respond?

P001: I think their work is to make you to feel good so they will always say something positive that you should not stop taking the -treatment, you see at the end, even when they advise you to decrease the intake of something but they will start by commending you on the progress starting from day one I think that is a -motivation for person and then which is adjusted, isn’t it they will tell you that if you do this and that and they will guide you where it’s not going well and they will make you to feel confident about yourself, you see, they motivate us and they warn us against giving up.

M: Okay, all right, is there someone with something different?

Ps: No.

M: Okay it seems as if we are tired now ((Ps laugh)). Okay, all right, thank you for sharing the thoughts you have about the whole thing and for trusting and sharing with us these things are strangers, we appreciated it a lot, we will ask the lady to re-imburse you as we had explained earlier on and then we will go to the nurse that has our files, they were put aside for those that have not yet collected their treatment. Otherwise, we appreciated it a lot, thank you.

Ps: It’s our pleasure.

The time now, is 11h53 end of discussion
