# Supplementary material for: “If we lose it, we are worried”: Individual and provider level perceptions towards weight change among people living with HIV who undergo TB screening in routine health care settings in Gauteng Province, South Africa
Source: PLoS One. 2025 Sep 22;20(9):e0331904. doi: 10.1371/journal.pone.0331904 (PMC12453174; doi:10.1371/journal.pone.0331904)
Supplement: S4 File — (ZIP) [file pone.0331904.s004.zip › S4 Transcripts_final/FGD 6.docx]

FGD 6

Transcribing Conventions

- **...** Ellipses indicate talk omitted from the data segment
- **(( ))** The transcriber’s comments.
- **( )** Empty parentheses indicate some talk was not audible or interpretable at all (we include the line for instance 20:15)
- **(.)** A dot enclosed in parenthesis indicate a short silence.
- **[ ]** Square brackets indicating beginning and the end of overlapping speech.

A Group Discussion Starts

M: The time now is 13:42 we are conducting a focus group discussion, with 6 participants and others are still going to join us, we have 5 females and 1 male so far, thank you so much ladies and a gentleman for agreeing to come and talk with us this afternoon, we very much appreciate your time and we know very well that your schedules might not be the manner in which we will prefer them to be. I am going to ask that we perhaps introduce ourselves, we have introduced ourselves, but we did not give you that opportunity to that then. I am XXX [The facilitator] I am a social science researcher.

M2: I am XXX [ The facilitator] basically, I am a researcher by profession.

M: Thank you.

P006: I am a xxxx (position) at the xxxx Study.

M: xxxx (position), xxxx Study. If you don’t mind me asking, I know some of us will have very soft voices but for the sake of our recorder if we could try and speak as loud as we possibly can, it’s a request.

P005: Thank you, my name is *((name and surname withheld for confidentiality reasons)) I am xxx (position).

P004: My name is *((name and surname withheld for confidentiality reasons)) and I am a xxxx (position) in the xxxx (project name), I have a profession in xxxx (profession name).

P003: I am*((name and surname withheld for confidentiality reasons)) I am a xxxx (position).

P002: My name is *((name and surname withheld for confidentiality reasons)) I am a xxxx (position).

P001: I am *((name and surname withheld for confidentiality reasons)) xxxx (position)

M: We are very lucky to have the xxxx (position) grace us with their presence, we appreciate you. We welcome you to the group, I am XXX [Facilitator], my colleague here is XXX[Facilitator], we are trying to conduct a focus group discussion which is going to take something like one and a half hour. We have already signed the consent forms and for the sake of time, the fact that you are here, we think you have agreed to participate. If perhaps you could tell us who you are and your occupations here at xxxx (hospital name)? ((Some people enter the room and there is some disruption)). Can we quickly welcome you to the group and ask you to get something that you would put here ((tag with a number)) as a number; we were already at number 6, if we could have number 7, 8, 9 and 10. So now we have 2 males and 8 females.

P: Yes.

M: Can we kindly quickly introduce ourselves, by numbers, not using your names, I think you are number 7, 8, 9 and he will be 10 and you will be telling us whether you are a counsellor or whether you are a professional nurse.

P007: Hi, my name is number 7, ((laughs)) I am a xxx (position).

M: Okay good to meet you my sister.

P008: I am number 8, I am a xxxx (position).

M: You are a xxxx (position) also.

P009: Hi, I am number 9, I am a xxxx (position). ((They put the recorder in the middle)).

P010: Hi, I am number 10, I am a xxxx(position)

M: It’s good to have everyone around the table, the reason we are here this afternoon for this discussion, is to find out what patients think about their body weight changes does not always match that which is measured at the clinics. I am going to maybe start with our questions (.) For instance, when an HIV positive patient attends a clinic for an appointment do you ask them about their weight or if they have lost weight? We are primarily talking about the work that you do around weight loss.

P003: As a xxxx (position), basically-when the patients come in the weight is already measured and recorded on the charts and so it is very important for us, to check if the drug doses do not tally with whatever you know or which should happen, then we always refer to the progress of the patient. So in that manner, the weights are very very important but you find that the patients themselves, they are very very anxious, the questions they ask for instance when they come in, if it’s the first time they are taking ARVs, their question is what am I going to weigh now because I think because of the stigma now people might know that he or she might be basically be positive because he has lost weight.

M: Okay, so for some of us, the weight has already been measured.

Ps: Yes.

M: Okay, what about other people, do we ask them…, when they come for their appointments, do we ask them for their weight, if they have lost weight?

P: Yes, we do.

M: The counsellors do. Yes?

P004: Okay, from the nurses’ perspective, definitely every time the patient comes to the clinic, you need to check the weight but also compare it with the previous weight if this patient is losing weight because in terms of collaboration of TB, you will know that when you are taking some investigation, in relation to the TB even if they are going to start the treatment, you check the weight against prescription, so from the nurses perspective, we check weight at all times.

M: So the counsellors check, the nurses check and the pharmacists would read that which is already written there on the file and then number 5?

P005: With us as research assistants, we basically ask the patients if they are losing weight and then we measure them and we also compare the weight that they weigh and the height with which they were measured at the clinic site and we also measure them as well as research assistants.

M: Okay, all right, for instance, we have already have responses from pharmacists, the counsellors, the nurses, do we have amongst these people, the people that have already responded, someone who would say in my particular case even though I am a counsellor, a research assistant, a pharmacist, my case is different from that which has already been said or is it the same?

Ps: Yes.

M: Okay, great and then why do we ask them these, why do we ask patients if they have lost weight?

P005: In respect to the work that we are doing here at xxxx (hospital name), is because we wanted to notice if they are aware if their weight is going up or down. So we wanted them to see if they are aware with regard to their weight loss or weight gain.

M: Okay, all rights what do other people say, we have heard about the xxx (project name) Study team, what about others, counsellors- why do we ask these people if they have lost weight or not?

P: It’s very important because they have to identify that since it goes with the CD4 count, it’s that we explain to the patient that, if your weight goes down, then the CD4 count it means we will need to check it, if it goes up, it means you are getting there.

M: Uhum, okay, all right.

P003: Also for instance, if sometimes although you find that the weight of the patient is going down and that it has anything to do with the CD4 count because of medication like for instance if they are on Alluvia, although they may be responding in terms of CD4 count or whatever they might be losing their weight because of diarrhoea.

M: Okay to check on the side effects. Okay, do we have someone who will say we check for some other reasons which have not yet been mentioned?

P006: The other reason, as we are doing xxx (project name) for TB, we are integrating TB with HIV so whenever the patient is losing weight there is a query just checking if the patient is having TB or not.

M: Checking for opportunistic infections, okay all right, you had your hand up number 4?

P004: In addition to what she just said, the BMI will also tell you if the patient is at the risk of getting TB, loss of weight is one of the cardinal signs on the tuberculosis and our body mass index will be the one which show you, this patient is at risk and you must act swiftly.

M: Okay, I get you, anybody with anything different from that which has already been mentioned before we move into our next question?

(.)

Okay, all right, in what circumstances are you more likely to ask patients if they have lost weight or not? (.) Where you know that in this particular instance, I need to have to know? You are seated next to me; I would not be able to see you even when you want to talk?

P003: Can you repeat the question?

M: In what circumstances, would you find yourselves compelled if patients have lost weight, where it’s a definite case that you definitely need to know, other than these that we have mentioned that you are checking for TB and stuff like that?

P002: I think it will be to see if the patients are adhering to their regime, are they taking their medication properly or not because we have lot of cases where they were not taking their medication and to see if they are losing and then we can find out why they are not taking it correctly.

M: Okay, maybe let me put this question this way, do we always, all the time ask the patients if they have lost weight every time they come for their appointments or are there instances where we say, under these circumstances we don’t ask if they have lost weight or not?

P006: We always ask.

M: Okay, do we have instances where we are less likely to ask patients if they have lost weight or not?

P003: I am just saying that if for instance we see that the patient is healthy in those instances we will not be compelled to ask the patient if they have lost weight or not but if you see the patient looks sickly then that will be an instance where we will ask if the patient has lost weight.

M: Oh okay, that’s an instance where you are less likely, when they look healthy.

P003: Yes.

M: Any other situation or circumstance?

P010: Sometimes those who are not adherent, weight loss occur, poor adherence

M: Okay, all right, yes number 4?

P004: Yes, as we say that we are taking it all times our observation will tell us if the patient is losing weight or not, will be compelled, if you listen that the patient has lost weight and moreover as she also alluded if you see that this patient is showing that she is generally not looking well, even the body will be showing that this might not be his or her body but then you will need to know what caused this patient to look more malnourished.

M: All right, okay, perhaps now we should move onto the next question, do HIV positive patients voluntarily consult us because they lose weight? Do we ever have those instances where they come to us because they see that they are losing weight?

P006: Some, they come.

M: They do, okay. Do we all experience that, patients coming to us because they are losing weight?

P:006 Like I said some do come and others do not come because they think its expected for them to lose weight when they have HIV and do not want to be judged for losing weight.

M: Okay, all right. If HIV positive patients report to us they are losing weight, what do we as health care workers or researchers usually do? (.) I am going to ask number 7?

P007: We ask questions like are you sweating, are you eating well.

M: What else do we ask?

P003: Did you get tested?

M: Testing for?

P003: For HIV, what else do we ask?

P: Are you on diet, to check if it’s intentional weight loss.

M: What else do we ask them, whatever it is that we can think of at the top of our heads?

P005: Also to get a bit of clarity from the patient when I ask them, if you are saying you have lost weight, how much weight do they think they have lost?

M: The amount of weight lost, right.

P005: If they are noticing that their clothing are coming much more loser.

P: If they have tested for TB and HIV as well.

M: Okay, all right and what about personal circumstances? Number 4?

P004: I wanted to say sometimes we have to check the issue of stress, if a patient is having some stressors and because some of the people or patients cannot cope well when they are under stressful situations and also in terms of collaboration on HIV/TB we also have to go deeper to check the issue of the other STIs and also the issue of…, for women we also check the cancer because if the person is on that stage, they may seriously lose weight, you must check on those circumstances.

M: Will that be any form of cancer, or will that be any particular cancer in mind?

P004: Cervical.

M: All right and what about personal circumstances, do we go to an extent…, number 2?

P002: P003 had an interesting case where the father didn’t want the child to take ARVs, ja that is also very important where I think physicians and the family must work together, the child was losing a lot of weight, a child was xxx years (teen ager) but looked like a 7-year-old.

M: Oh no and what happened eventually?

P003: We had to try and talk to the father because the problem is the father, okay this patient was non-adherent because the patient was non-adherent, I wanted to find out the reasons, every time I ask the old lady, the granny, she will be cagey and she will not give me the reasons so this particular time she ((not clear)) and asked that the gran must go outside and that I should talk to the child on her own because I wanted her to tell me the actual truth because you find that with children, the problem with HIV is that, they cannot accept it as it was brought to them by their parents.

M: Did you say it was a xxxx (teen age)?

P003: Yes.

P002: Around there.

P003: She was very very skinny, very very thin, so I wanted to find out exactly what the problem was and I had to find that out from her and I find out that she was not on medication and that the father was saying she must not take the treatment, she must take traditional medicines. So I had to call the granny in, since I know what was happening and I ask the granny, the granny was so cagey and I asked them for a follow up visit, early next month again to see if there was any progress.

M: Okay, so the personal circumstance is prohibiting a child from using ARVs.

P003: Yes.

M: What other questions will you ask when a patient presents with drastic weight loss issues?

P002: We also had a case where a girl was not taking the medication, the medication is free, she was giving a lot of reasons why she was not coming for her medication and amongst them she couldn’t pay for transport and things like that but the medication here is free so I really didn’t understand why she can’t come here, maybe a transport is a problem but it’s not every day, it’s once a month, every three months, so I think they try to invent reasons because I don’t understand why they don’t want to take the medication, because we had this girl, she was very thin as well.

M: Okay, all right. Do we have other people who want to share with us some personal circumstances, the type of questions that they will ask the patient with regard to personal circumstances that might lead to them losing weight?

P006: Others would say unemployment, neh, there is no money to buy food in the family, which will ultimately lead to weight loss and then we observe the weight loss during the visit.

M: Okay, out of curiosity, do we sometimes get patients who would be losing weight because they are on diet? They are trying to lose weight on their own whilst they are on ARVs?

Ps: Yes.

M: Really? How often do we come across that? Would you say there is a particular group, where you could say, this is the particular group that will usually not want to gain weight?

P004: Ja, especially the adolescents, around the age of 18, when they start to gain weight because of ARVs then they will tell you that these things are now making us to become big so we better be on diet.

M: Okay, so it will usually be the younger ones?

Ps: Ja.

P: Ja, we do over weights ((the audio was not clear at this segment)).

M: Okay, do we at all ask them about lifestyle as it is that they might be doing that might cause them to lose weight?

P: Yes.

P009: Yes, we do?

M: Okay number 9, what do we ask them with regards to lifestyle?

P009: Maybe you are in a relationship, are they using protection, how they cook their food.

M: How they prepare their meals, uhm, ((not clear)) you are speaking so softly I can hardly hear you?

P: They mustn’t overcook cabbage and spinach, the must does not overcook food like that, ja.

P: It’s important also, it doesn’t go with ARVs, they need not eat more fats, more spices, they need to cook it properly.

M: Okay, all right and what tests might we arrange for these people as health care workers if they report that they are losing weight?

P009: HIV test.

P007: TB.

P004: Cancer of the cervix.

P010: Opportunistic infections.

M: What other tests do we recommend for these people, if we don’t them ourselves?

P: General testing.

M: What do we mean by general testing ((asks M with a chuckle)).

P: To test if everything is okay .

M: For instance, you mention that you also test for TB and I understand with my very little knowledge that you collect sputum from them and if there is no sputum, what do you usually do?

P001: Yes, it’s chest x-rays, it’s these general like physical things the doctor can do like listening to your breathing inside, so that we can hear, you know when you are breathing, we can hear like what do you call it like inside when there is a lot of phlegm and everything inside.

P004: Ja, ja.

P001: So when the chest is clear and then that one you might say, go for chest x-rays or something.

P: We also check for diabetes.

M: Okay, diabetes, number 1, number 9 and number 5, I think you wanted to say something, between the two of you let me allow one to talk.

P005: She was asking if we check the spit test for TB and I told her that I am not sure about that. We use sputum so I am not sure.

P003: There is a spit test here in the form that is why they are asking the questions.

M: Okay, I would have thought that xxxx (organisation name) will understand that better.

P006: when they talk about ((they all speak at once)).

M: Can we have one person speak at the time please)).

P006: but when they say that in the book, they refer to sputum, when they say go for spit test which is sputum test and then if you are unable to produce, we usually track then induce the sputa neh? If the sputa fail then there is x-ray, if the x-ray fails then there is sonar, a type of x-ray but which is a little bit expensive.

M: So you have ways of getting around these things, okay, the blood tests, we talked about HIV test, any other blood tests that we arrange for these people that have reported weight loss?

P006: TB culture, we do culture on them.

M: TB culture, other blood test?

P004: Haemoglobin.

P003: CD4 count and viral load.

M: Okay and what about referrals? What referrals do we usually arrange for these people that have come to us and have explained or complained that they are losing weight?

P: We refer them to the dietician.

M: Okay, under what circumstances do we usually refer to them to dieticians? Do we refer them all the time or would you have particular instances where we say this time around, we are sending this patient to a dietician?

P: I think when they are losing weight because they have complained that they are losing weight of about 5 to 10 kilograms within a short space of time.

M: Okay, those that lose weight drastically. Who else do we refer to other than dieticians?

P006: The doctors.

M: Usually, under what circumstances do we refer them to the doctors? Do we have particular instances where we say we will send to the doctor, it’s not for number 6 only?

P004: We check as we were elaborating just now what will the protocol say, if you check and you see that the patient is not improving and then you check all that you were supposed to check, you check TB, you check all the circumstances, for things like you will not get answers, you will refer for the second opinion, to the doctor maybe who will be able to check other tests.

M: Okay, after you have checked everything and what about social workers, do you refer to social workers?

Ps: Yes, yes.

M: For what purposes do we usually do that? Number 7, I saw you nod your head?

P007: I was saying yes, we do refer them to social workers?

M: Under what circumstances, number 8?

P008: I think it’s whereby maybe they are having a social problem, maybe the social worker will go and check if everything is okay.

M: If you say social problem, would you be able to state what the social problems are usually or maybe just one, yes number 9?

P009: Unemployment.

P008: I think with stigma also, when the patient is stigmatised.

M: What do you mean by stigmatised?

P008: Like at home, they don’t want you, you know, others told me that they don’t care about me and they reported for three months they don’t care of them, even if she was admitted, they didn’t come to visit, yes.

M: Okay, all right, number 3 wanted to say something?

P003: Yes, I wanted to say that we also refer to psychologists and that’s what basically from her, I have been reminded of one patient at home, because she is HIV positive she has her own plate, she has her own spoon, her own mug, her own, own. She has to see to finish.

M: That thing still happens?

P003: Argh, it’s terrible. It’s terrible so psychologists are still very very important people to refer to.

M: Okay, so you also refer to psychologists, so these people, whom you have mentioned, are these internal referrals or do we refer outside the institution, is it inter-departmental?

Ps: It’s internal.

M: Internal.

P003: People who are on denial and do not want to accept completely and they are religious people, you can always refer them to their pastor church person or whoever is in the church or whoever they trust or is who has taken their confidence so to speak.

M: Oh, okay. So you also refer to people they trust, outside.

P003: Outside.

M: Okay, all right and what about the follow ups, do we at all think that we manage to do follow ups?

P003: We try but not successfully.

M: What do we mean when we say, we are not successful?

P010: Usually, they will come but after some time, they will just disappear.

M: Oh because they disappear, you are not able to trace or to follow them up, okay, number 7?

P007: They usually ask, are you going to give us money but if you say no, maybe you keep calling them, they will say, we will come but they will not show up but once you give them money, they keep on coming.

M: So follow ups become possible only if you provide finance.

P007: Yes.

M: Okay, yes number 4?

P004: I just realised when we were doing this xxx(project name) that one of the things that is failing in terms of the follow up, the system.

M: Right, what about the system?

P004: The system is failing in this way like when we were checking, it was through when they were checking the IDs that this patient has passed away two months ago but the clinic has not find that this patient has passed on. No follow up was made and it was detected by the ID system which we are using for the patient to go through and that when you check, you find that the patient really has passed on and as I am saying, the system, you will find others when you are doing that, then what you call, when you check the file, you find that the patient since came here last October no follow up was made. I think our system need to be revitalised, to make sure that if the patient is missing, we report that that patient has not come and you will be able to know that I must make a follow up. I have got 12 patients that I must call tomorrow and find if they are here or I will check if it’s here in this clinic, you are checking if all are here so that you are able to know that your patients…, you find that patients passed away last year but the system did not catch up what happened.

M: Okay, number 8?

P008: Even if we give them a referral from here in the clinic, then maybe you say go to XXX clinic, it’s whereby they get a chance to disappear, they don’t want to go to local clinics, they want to attend here, so after 6 months or a year, she comes back and then she is very ill and then we need to start afresh.

M: They prefer not to be referred to local clinics.

P004: I think it’s the system again.

P009: It’s the system because if I refer to the clinic, I can find out that that person didn’t come and make sure that they go there.

M: Number 5?

P005: I also wanted to add on what number 4 said, that when we were doing the xxxx (project name), you find that to get your patient to come back for the follow up, you also have to call their next of kin because that is what we ask them when they are enrolling and they also give us their contact numbers, people that they are really close to, who know their situation so that we can actually ask them to pass the message forward. When they won’t even get their next of kin, so we try to ask for that person on their next visit to the clinic.

M: Uhm so somehow, we have some of the systems in place to try and deal with that issue follow up, which at times does not work well. And why do you think HIV positive patients actually lose weight? I know that we have mentioned a few, but can we touch on it briefly, number7?

P007: I think they lose weight because they don’t take medication on time or they hide it because they didn’t disclose their status to their families or their partners so they take the medication when the family or their partner is not around.

M: Okay, they don’t adhere to ARV treatment.

P003: But what will make them lose weight is before they can even come for the treatment ((Ps start speaking at once at this segment)).

P006: The level of acceptance will differ from one person to another. The other one will just accept you know from the first day after the test, others it takes a strain to deal with it.

M: And then this inability to accept the diagnosis, how can it actually make me to lose weight?

P006: It does.

P004: Stress. I think the issue of stress, immediately, you get stressed, you are no longer taking your food well, you are always thinking what will happen to your children and that thing makes you to lose weight bit by bit.

P009: Like when you disclose, will they give you support or not. ((They speak at once)).

M: Okay, let’s try and focus on this point on what make them to lose weight before they start taking ARVs, yes number 2?

P002: I would say it’s an immune deficiency problem so the immunity itself is failing so everything, now your body is…, everything can attach your body and you are not responding, you can’t actually fight for anything, so you become weak and you are going to start losing weight.

M: Number 3, you wanted to say something?

P003: Yes, I was going to say exactly what she has just said, that’s number 1 but I was going to continue saying, that there was a study where somewhere they were querying why people were still losing weight even though they had started taking their ARVs. So basically, I think the losing of the weight is in what they have just said, like it is intrinsic, it is definitely part of the disease more than anything else and that some other people because people are different, some other people since basically once they start taking their medication, they will continue to gain weight but there is still people who continue to lose weight even though they are already on ARVs.

M: Okay, now that we have already started about people losing weight when they are already on ARVs, I think we did get a response where a person were taking ARVs but then the adherence is not good because they have not yet disclose and the person is afraid that the family members will see. What else will make people that are already on ARVs lose weight?

P003: Stress.

M: Oh yes, we did mention stress. Anything else?

P002: Someone was saying not being able to buy food. So financially they can then they lose weight.

M: Financial circumstances, right, (.) why do we think patients may report weight loss. For instance, when you are there at the clinics, I think most of us indicated earlier on that we ask them if they have lost weight. In instances where they have lost weight, what would make them say they have lost weight? (.) What will indicate to our patients that they have lost weight, yes number 4?

P005: When they were wearing size 38 and now, they are]

M: [From size?

P005: I am just saying because of the loose clothes.

P004: Dress size and in some instances.

P: People’s comments on their appearance.

P005: People can see physically.

M: Anything else? (.) What else will make me to come to your clinic and say doctor or nurse or dietician or pharmacist, I have lost weight, other than the dress size?

P004: The comments of other people like those who are supporting you. They will be able to look at you and say, you are no longer the way we know you.

M: Okay, we just received that one, yes number 9?

P009: Maybe I haven’t been eating well.

P003: What about a patient who has a scale at home, who has really weighed herself and then found out that they have lost weight.

M: They have weighed themselves and realised they have lost weight, okay. Do we often have those patients who will have weighing scales at home?

Ps: Yes.

M: Okay, interesting, what else?

P006: Others will compare the weight that was done at the clinic the previous time, for the last three months I weighed and now I am weighing so much.

P002: Maybe they are feeling weaker.

M: Okay, I keep coming through this point of people feeling weaker can you explain to me in lay man’s terms what feeling weaker mean.

P002: There is no more laymen’s term in that feeling weaker means tiredness, ja, you don’t have that stamina, you know.

P004: You know in the morning, you will find them on the tables when you look at them you can see that they are tired but it’s still morning.

P002: I think]

P003: [And also walking up the stairs will take a strain on them.

M: Oh okay, now I understand and I have been asking myself what is this feeling weaker? And it has been coming up quite often. ((Ps laugh)). Thank you for the explanation. Okay, is there stigma attached to losing weight, I think we did hear something like that from either 7 or 8. Do we have stigma attached to people losing weight?

P003: Yes.

P004: Even before, if you lose weight, before you become HIV positive people can just see you are losing weight, they will tell you now, you are driving Z4, Z4 is HIV in the laymen’s language, so if they see, it’s like you are losing weight, they remind you, you must go and test, pastor maybe you are on the road. So there is stigma attached to the loss of weight.

P009: And others they can even say you are HIV positive when they see you coughing.

M: Just by coughing.

P010: Even when you are diabetic because of loss, they can say that.

M: Uhm.

P: And I think we have a black culture that a black woman has to be round ((Ps agreeing with one another)), they will just be worried, when number 4, they won’t even look at you.

M: Okay, just because you are losing weight, okay, anything else with regard to stigma? (.) You have already mentioned, driving a Z4, the names and the terms, any other name associated with losing weight and HIV, the terms?

P: *Amagama* *amathathu* ((Three words)).

P: WE must go and check OMO because those are three words.

M: So they say OMO because OMO has three alphabets?

Ps: Uhm.

P003: It’s HIV.

M: Interesting.

P009: And Z3.

P: *Ayaduma* *amabhasi ((we can hear the sound of the buses))*.

M: What does that mean?

P: We will bury him or her at any time ((there is laughter in the FGD room)).

P006: We are heading xxxx ((the name of a cemetery)).

P004: The bus represents the mortuary.

P003: I never heard of that.

M: From what we have shared with our patients, I am sure over the years, you have developed some relationships with these patients. For instance when they share some of these things with you, what do they say they feel about all this stigma which is directed towards them? Should you have been fortunate enough to have those patients that will share those with you?

P001: In my experience, I have been with people who have accepted the virus and living with it, they joke around it. When the weight loss issue comes in, one had flabby muscles around here, now I am losing weight so those who have been on treatment for a longer period accept that it’s their new life now, you know they adjust to it.

P003: Some of them call the medication sweets.

P001: Yes.

P003: Bring me my sweets.

P004: They call them Smarties.

M: Oh okay, otherwise what do they say, they feel about all this stigma which is directed towards them, if we have heard, yes number 5?

P005: There was a patient…, a participant basically who had an accident when I spoke to her and then, she basically (( )) she does have (( )) but she has learnt to deal with what she is going through and that she has support and also she reads motivational books and is working on you know getting her self-confidence back because she did feel that when people were talking about her, she was attending the HIV clinic, ja ((this segment was not clear at all 53:00-54:48)).

P003: You do find that other people although they have been on tablets for a long time the stigma issue, they have not accepted really like for instance I always now insist on people getting to know the type of medication that they are using because you will find that the drug is CIPLA, tomorrow it’s made by ADCOCK so to say this is a brown tablet or a yellow tablet or a white tablet or whatever doesn’t work. So I say to them this is your Efavirenz, this is your Stavudine, this is your whatever, whatever so whenever I sit down with a patient I ask what medication are you on because I think it’s very very important because it’s the medication that they will take for life and wherever they go they should get it. For instance if they had an accident in XXX[Name of the location]they must be able to say, look I am on ARVs and say it’s name but you find that those patients that have not accepted that they have a disease do not even want to know the name of the medication that they are on and they will be angry at you, I mean I have been on this medication of whatever many years, don’t you think I know them by now ((says P003 mimicking an angry patient)). You find things like that, even though they are not going to be telling you that there is stigma attached but the way they say it shows you that they have not fully accepted what they are on.

M: I get you. Let’s now move onto our next question, if a person is attending a clinic for HIV and is taking ARVs and is receiving counselling and stuff like that how do you think their weight should be?

P002: I think they should stabilise at least instead of going nowhere]

P005: [Underweight.

P002: Even if they are not picking up weight but not lose it.

M: Right, what do other people say, number 2 says it should stabilise, number 4?

P004: It must be in line with the BMI, you have ranges that a person of this weight and height should be and it’s the BMI so they must not be over or under weight.

M: It should be in line with the BMI, okay, do we have anybody else with something different from that which has already been said? (.) Okay, great and what about their shape, what should their body shape be if they attend ARV the clinic?

P005: Everybody has different shapes ((Ps laugh)).

M: So we will expect different shapes.

P003: But ARVs deform them, and they are very worried about that. They are getting even more worried about that now than the stigma.

M: Okay, we will come to that after this. What changes might happen to people’s weights, you have already indicated that they will need to stabilise and if the medication is not going well, other than deforming them, what else would you expect that this will happen to a person that is attending ARV care or HIV care if the treatment is not going well?

P003: I did not get the question.

M: If the treatment is not going well what would we expect would happen to our patients?

P003: The patient will get sick and it will depend on the type of sickness she is going to be affected with. If it’s going to be TB obviously, she is going to lose weight, diabetes, lose weight, meningitis, no, it just depends but since the patient is not taking the medication the CD4 count is low and the viral load is up, the opportunistic infections will come in and the patient is definitely going to get sick which is why maybe all of them that have defaulted, they will now come back to the clinic because now they are sick.

M: Okay, anything else other than them becoming deformed or becoming sick, is there anything else which expect will happen to a person if the medication is not going well?

P010: Some pass on.

P003: Ja.

M: Some will die, okay, all right and what about ideal body weight and shapes. I have just passed around the photos of people; if we could perhaps show this to one another in the group and then discuss which one is an ideal body shape ((they laugh at the silhouettes)).

P003: Oh I thought all are the same.

M: No, they are not, can we do that quickly? ((M counts them)) oh okay, it’s 9.

P002: I think number 9 is fine.

M: Can you read number at the back for me, what number is written on that photo.

P002: Number 4.

M: Number 2 suggests 4. How many people will think that number 4 is an ideal body shape? And why do we think number 4 is an ideal body shape?

P006: I am sorry before we go to these votes, are we just concentrating on the HIV side because it can happen that a person was like this before so I wouldn’t say it’s treatment, what if the person was like this before he was diagnosed or whatever]

P007: [Sometimes others do get fat once they start the treatment.

M: We understand the circumstances that could have happened before and after but right now, what we are trying to do is, let’s look at these photos that we have in front of us and amongst us decide which one we think is the best, is the one which is ideal. Let’s that quickly ((Ps are talking to one another discussing silhouettes)) I am trying to help you here, you are pressed with time and I am trying to get us to finish this quicker.

P002: You know I think hers is right, ((number 4)) when you normally, a normal sized person you kind of compare without going to Body Mass Index, it’s just a brief way of saying things, you look at your height and your weight should be like…, let’s say my height is 150, my weight should be around 50, 45 or 50 and even between the range 55 and 45, that’s the right weight. That’s on the general look so I would say a person someone who is like that should do like number 4.

M: Do we agree with number 2 or do we have people with different opinions than those of number 2?

P002: Let me see.

M: Okay, do we all say number 4 has the ideal body weight?

P006: No.

Ps: Where is that?

M: Here is number 4.

P009: Here is number 4, I have it.

P003: Oh okay.

P004: Let see others?

P005: This is what I am thinking, I don’t say it should be number 4 with the ideal shape because it will depend on the individual themselves, so I would say no, the ideal weight should be this.

M: That’s number?

P005: It should be not too big and not too skinny as well.

P002: What’s the difference in those two let’s see.

P005: It will depend on me because if I was a person who was this big, I would say that this is the ideal body that I like than that one.

M: I get you, perhaps do we have a person who is neither choosing number 4 or number 5 so that we can continue? (.) Are we all on 4 and 5 and can we perhaps by the show of hands indicate how many of us will go for 4 and how many of us will go for 5?

P: I was going to show you that other picture.

M: Which one?

P: That one.

P002: But that’s skinny.

P003: What do you regard as ideal?

P002: It’s number 3.

P005: Most of them are complaining because they don’t show their legs now, they wear long skirts and something like here, they will be small here but]

P002: I will look at it in terms of…, she made a very good point that everybody is different, she might go on a full diet while she is losing so much but she is not even there, you see.

M: So we have chosen 4, 3 and 5 neh?

Ps: Uhm.

M: All right, do we have a particular for men, where we would say this weight is ideal for men and that weight is ideal for women?

P003: No, BMI, BMI.

M: Same and it’s BMI guided, okay and what about the most attractive one?

P002: Number 3 is good, it’s nice.

M: It’s number 3 neh.

Ps: Yes, it’s three.

P002: I think even number 2 is not too bad, can wear nice.

M: It’s number 3 and number 2?

P004: The attractiveness and beauty lies in the eyes of a beholder ((Ps laugh)).

M: This discussion that we are having now, these things that we have mentioned and stuff like that, what is it informed by? We have influence from the community, the culture, the family, health services and the media, that which has given us opinions to say I like number 3, I like number 4. What would you say has informed your opinions predominantly in choosing these, number 2?

P002: For me, personally I like to be in such a way where I can run, I am physically in such a shape that it’s not difficult for me to be active and to do the sports I want to do and the things I want.

M: Okay, what do other people say? What we have just said, it is what we were taught at home when we were growing up? Is it our culture, number 5?

P005: It’s a personal view, it’s how you see yourself because there are many factors, you can come from this side but then you do lose weight and then you realised that I haven’t seen myself becoming number 3’s size, you are much more focused on that size because of the way you are right now and where you see yourself going.

M: It’s a personal view, it’s about wanting to be physically fit and be able to walk around and get around without hassles anything else?

P006: If you gain too much that the diseases will come.

P009: If you are like this ((number 8)), you have a problem because people come to the clinic with blood pressure and some suffer from heart attack.

M: When did you learn that, that if you are like number 8 you will get diseases like blood pressure and heart attack?

P006: Health education

P: Health care services.

M: Any other influence, number 5 wants to talk?

P005: To add on what she said, another influence could be family history, seeing your mother or a family member who was that size and they got sick and they got a heart attack or something and now you are also going in that direction as well.

M: Okay, all right, why do you think HIV positive patients will think that they have lost weight when the weighing scale indicates that they have not lost weight?

P002: Psychologically.

M: What do we mean by this, psychologically?

P002: They know they are sick, they are worried about it and they think that they are losing weight, it’s just maybe worrying them always and they think that they might be losing weight whereas in the meantime they are not.

M: Okay, what do other people say?

P001: I think also it’s protecting themselves from the public, when you look skinny people are diagnosing you so you rather be a bit fatter so that people don’t say that you are ‘driving a Z3’.

M: Okay, so you mean that in order for us to protect ourselves from the stigma, we would want to gain weight?

P003: Yes. I hear somebody say no, I think yes.

M: Oh, I didn’t get that one, okay and what do you think losing weight means to people that attend the HIV clinic? (.) I am attending the HIV clinic but I am losing weight?

P003: It’s the stress you are going through.

M: What does that mean to me as an HIV positive patient that is taking ARVs?

P005: Maybe you are not responding to your medication.

P004: Ja.

P005: The treatment is not working.

M: Number 4, you wanted to say something?

P004: You are on your way to the grave.

M: On my way to the grave. Anyone else, I am losing weight but I am attending HIV care, what does that mean to me?

P003: Maybe I have got another disease besides HIV.

M: Okay other sicknesses, yes, anything else, okay, all right. What do you think gaining weight means to a patient that is attending HIV care?

P003: That I am getting better.

P002: Ja.

M: Anything else?

P001: The treatment is working.

P002: I am not going to the grave ((there is laughter)).

M: Aren’t we just reversing everything, this one, naughty ((says M referring to number 2)) I am kidding, I am kidding and then why would this usually happen, I mean do you sometimes have patients who would come to the clinic and say they have gained weight when they have not?

P002: No, I have had a patient who was very happy now because she has settled, gaining weight and]

M: [Was the scale confirming that?

P002: From her, according to the file, it was quite familiar to the last time, she was here but she felt more confident, uhm, I think for her, it was more the situation she was in, where the situation was getting better, so she was more concentrated on her disease and getting better and stuff like that so.

M: If you are saying the situation was getting better was does that mean?

P002: I think her home situation, she was having some problems so her home situation was getting better and I think she was… she felt like she was concentrating on herself and her disease and that made her feel better.

M: I get you, anything else? I am gaining weight, what does that mean to me, other than the treatment working and other than these reverses that number 2 was mentioning? (.) Okay, in your experience, what do you think is the best way to ask people living with HIV about losing and gaining weight, number 4?

P004: Did you notice any weight change? The patient will explain whether he is losing or gaining. If we ask did you notice a weight change, whether losing or gaining

M: Any other? ((The first audio-recording ends)).

We are saying that we reassure them that life is more important than how they look. You have already said that you will refer them to the doctor who might when necessary change the treatment. Do you only recommend or send them to the doctors only?

P002: We counsel them also.

M: You counsel them, will that be all?

P006: ((Not clear at all 00:52)).

M: Anything else?

P003: We send them to the dietician because the dietician might try and do something about it but people differ.

M: All right, okay, these are all the people you send to, dieticians, the doctors]

P008: The counsellors.

M: Okay, the counsellors, in general, do you think the body shape changes in people living with HIV?

P003: Pardon?

M: In general, do you think that the body shape changes for people living with HIV?

P003: It’s not everyone.

P004: It depends on the individual for some they take more than 13 years taking treatment but they are still looking the way you met them so I think it depends on the individual.

M: Okay, some people’s body shapes will change and others will not and for those whose body shapes change, why do you think these changes occur?

P003: Mostly the side effects from the drugs they are taking.

M: The side effects of ARVs, anything else, other than that?

Ps: Nothing.

M: We are towards the end of our discussion and I think this is almost our last question, have your patients shared with you reaction from the following people in particular in response to their body shape changes. For instance their spouse? Have we had patients who will come to us and express concerns with regard to their spouses’ comments with regard to their body shapes, yes number 8?

P008: Some women share with us that their spouse tells them that they are changing, even the life in the house is changing because of ((not clear)).

M: What was changing in her?

P008: Here, the breasts and her legs here, thin thighs, the loss of fats in the face.

M: So we have had an example of a patient who has received comments from her spouse. The spouse complains. Do we have another exciting case with regard to the spouse responding to the partner’s lipodystrophy? (.) What about children?

P003: In terms of?

M: In terms of body shape changes, the comments or the reactions of children when they see maybe their parents change in terms of how they look?

Ps: No.

M: You have never come across it?

Ps: No.

M: Have we all not come across that? (.) Okay and what about the extended or wider family? Like your aunts, your grandma, your uncles?

P003: I think we have never directed those questions to them. They come to you and complain about their body weight but we have not asked like, it’s you who is worried about this weight maybe it’s something that we should start looking into so that while the patient has come to you complaining, maybe basically because some other people have either complained or they are teasing her or something like that, I don’t think that it’s anything that we have looked into. I think we are just concerned about them and then they come they tell you, I am worried about this but we have never really found out why, ja.

M: Okay and what about friends? If at all they have shared with us what their friends reactions have been with regard to them having their bodies change as a result of ARVs?

(.)

P004: This thing of other people has not been explored much. It will need to be explored, if we generate some support groups are the ones that are going to explain in details how the community view it because there are others who do not have a problem of disclosing outside but it has not been explored further in terms of the outside, how the people view it.

M: Okay and what about the communities where we come from, our neighbours?

P003: Our neighbours need to be educated about HIV never mind the body whatever neighbours still have to come and test, that’s the problem with all your TV, with all your radio and your educational talks, people come to the hospital or come for HIV testing only when they are sick, which is a big big problem, they should come long before that. It should be a yearly thing. If I were the department of health minister, nobody will come to my hospital without being tested.

P004: I think the re-engineering of nursing is trying to take people in the villages; they will no longer come to the clinic or to the hospital. The Department of Health is working on that PHC re-engineering to ensure that there will be people who will be working outside ensuring that these people are allocated in these places where you work around, it means there will be no one who has an HIV ((positive)) status that will help us with this.

P003: That will be better.

P002: But something I find very strange, my husband’s father was a doctor and he was explaining to me he was working in those rural areas and he was explaining to me when people die of AIDS, he wasn’t allowed on the death certificate to put AIDS, I think that is a big mistake because when the social worker or whoever the state knows that this person has died of AIDS, I mean you can start with the family, screening the family and therefore go around before…, because you start by that or at least start by that.

P003: I’ll answer that, when you take insurance, insurance have changed they have moved a mile or whatever if you are insured and you have died of AIDS at that time you were not going to get that insurance money so what used to happen was, I have died of TB, I have died of meningitis that’s it so I can’t write AIDS.

P002: But then when we don’t know, we don’t screen and that is when things get worse.

M: What about the community, is that what we are discussing, our community reacting to us once we have developed these changes in our body shapes, is that what you were discussing?

P004: Definitely.

P002: Yes.

P008: Everyone will know that you are sick because you are losing weight.

M: And how will they react when they see me losing the fats on the face?

P008: Sometimes they whisper.

P002: They get scared I guess.

M: They get scared of me as an individual and how do they usually portray…, what kind of actions or behaviours are associated with that?

P009: Discriminate.

M: Okay, they will discriminate against me. number 9, what do you mean when you say discriminate? What will they do.

P001: For an example, they will like not be your friend.

P008: No one comes to your house.

M: I won’t have visitors.

P002: And if people are not educated about how the virus is transmitted, so they ((people talk at once))]

M: [You are both talking at the same time, I couldn’t hear you sorry?

P002: What I am saying is if people are not educated on how the virus is transmitted, then they won’t know then they might even think of something or I don’t know that might transmit the virus because as *((name withheld for confidentiality reasons)) was saying some people have special plates that means they think this kind of thing will contaminate you.

M: In general, what do patients with HIV whose body shapes have changed as a result of the use of the ARVs, lipodystrophy. Have they shared with us as to what it is that they actually feel?

P003: They are not happy; they want something that can take the fats out of their bodies. They are asking all the time when is this going to go away, what can I do for this to go away?

P008: Asking for the surgery, to cut it, yes.

M: Do you not refer them for those, to remove the hump?

P006: Ja, it is]

P010: [There is a department called plastic surgeons.

M: Plastic surgeons, you did not mention them as one of the people you refer to. Okay, anything else at all which you want to say before we close? We really appreciated your participation. We have come to the end of the discussion; we appreciated your time and your input. We apologise, I think we took more time that we had thought we were going to take. Thank you. Okay, this is the end of our discussion; the time now is xxxx

End of interview
