# Supplementary material for: “If we lose it, we are worried”: Individual and provider level perceptions towards weight change among people living with HIV who undergo TB screening in routine health care settings in Gauteng Province, South Africa
Source: PLoS One. 2025 Sep 22;20(9):e0331904. doi: 10.1371/journal.pone.0331904 (PMC12453174; doi:10.1371/journal.pone.0331904)
Supplement: S4 File — (ZIP) [file pone.0331904.s004.zip › S4 Transcripts_final/FGD 7.docx]

FGD 7

Transcribing Conventions

- **...** Ellipses indicate talk omitted from the data segment
- **(( ))** The transcriber’s comments.
- **( )** Empty parentheses indicate some talk was not audible or interpretable at all (we include the line for instance 20:15)
- **(.)** A dot enclosed in parenthesis indicate a short silence
- **[ ]** Square brackets indicating beginning and the end of overlapping speech.

A Group Discussion Starts

M: It’s one o’clock now and we are at xxxxx boardroom, with 7 xxx (positions). Are we all xxx (position)?

Ps: We are xxx (another position)

M: How many (position)?

P: Two.

M: It’s 2 xxx (position) and 5 xxx (position), am I right?

Ps: Yes.

M: Are we dieticians from XXX [ Name of the hospital]?

P: We are from XXX [Name of the hospital].

M: The xxx position, are we all from XXX [Name of the hospital] as well?

Ps: Yes.

M: Okay, I think we can start.

M2: I think we can start for the sake of time, when an HIV patient attends your ARV clinic, they come to you and they talk about weight loss for example, what do you ask them?

P: Intentional or unintentional

M: Okay, just to rephrase that question, when an HIV positive patient attends the clinic for an appointment do we ask them whether they have lost weight in most instances?

Ps: Yes.

P: We do and we also have like their weight every time they come.

M: You do ask and, but you can also look at the

P: Look at the record, yes.

M: So do we all do that or is there someone who would say, we don’t ask at all?

P: No we do.

P: We ask.

P: We do.

P: That’s one of the things that we all try and do every time.

M: Okay we all ask, great and why do we ask them if they have lost weight?

P: We use it as a marker of the disease and also response to treatment, depending on how long they have been on treatment and they have been with us.

M2: This ((server)) is making noise and for the recorder and you are very low.

M: It does make a lot of noise, let’s just try and speak a little louder than we usually do for our recorder.

P006: So for me the reason I will ask weight loss or weight gain for that matter, is because it’s useful as a sign constitutional illness or active disease and if you have emaciated, wasted patient who you now have started on treatment and have identified disease process and they are gaining weight it’s a good marker for the response to treatment and I think what my colleague was saying in terms of unintentional and intentional is probably the first question because what we will ask because there are people trying to change their weight so ja.

P007: So we ask about the weight loss because of TB co-infection with HIV is very common and it’s one of the constitutional symptoms so before we start we do need to rule out TB because of the ((sounds like iris)) if the TB is there, we need to treat the TB before we start ARVs so it’s one of the constitutional symptoms of TB so that’s another reason to ask.

P: I think for us it’s also a marker of like their nutritional status for us obviously keeping up with nutrition as one of the ultimate treatment so that’s the first thing that we will check. They either need to lose weight or they need to gain weight so either way. It’s a sort of a marker of how they are doing nutritionally.

M: Okay.

P: The other thing you want to know is if they are losing weight is food security because if they have no access to food then they will definitely lose weight so it’s not necessary related to the disease but it’s just because I don’t have food.

P005: If they are on D4T, or Stavudine, then you get side effects of Lipodystrophy and then the weight changes and we also check for that sign as well in addition to asking them about losing weight or gaining weight.

M: Okay, so it is checking the side effects of D4T, it’s food security, it’s to check if there is this TB co-infection, the marker of another active disease and to monitor progress of the ARVs. Anything else other than these which we have just mentioned, number 1?

P001: Some people have psychological issues which are going on; they are stressed, relationship problems, so some people tend to lose weight when they don’t have appetite, so you also need to ask if they have got personal problems.

M: Psychological issues, do we ask these all the time or would we say that there are instances where we are less likely to ask them if they have lost weight.

P007: If we see that their weight is the same, it’s stable from the previous visits then we don’t have to ask.

P001: You don’t need to ask because we have their weight, unless is applicable to ask why.

P007: Some patient complain that they are losing weight, but when you check their weight, it’s the same so yes they are complaining but according to the weight, the previous visits, there is not difference. Ja.

M: In instances where the weight is stable, you do not ask. Is there any other instance where we feel that in these circumstances we don’t ask if they have lost weight other than stable weight?

P007: If the patient has the symptoms of TB, they are coughing and fever then we ask again weight loss questions.

M: So you are most likely to ask if they have got TB symptoms.

P007: Yes.

M: Okay, all right.

P007: and if the patient is on D4T, DDI then if the patient has other symptoms of hypolyctemia then we also, they also got weight loss with that.

M: Symptoms of hypolactemia, If you were to explain that to a lay person like myself, what does that mean?

P007: Lactic acidosis, you see it is the side effects of DDI and D4T, so we don’t have so many patients on that, now that regimen 1 and we are on regimen 2 so the patients are on both, it is not that common now but if patients who complain about weight loss and they are on D4T or DDI then we check for Lactic Acid because of the symptomatic lactic acid they do get weight loss also.

M: Oh okay, I could imagine, even though I am a lay person, but if I think of an acid, I think it could make a person lose weight. I am thinking about tartaric acid also ((laughter)). Okay, do we have instances where HIV positive patients voluntarily come to us as dieticians and as medical doctors because they are losing weight, it is not like it’s their appointment but they approach us to say Dr so and so I am having a problem I am losing weight.

P: Ja.

M: How common is this?

P004: It is not common.

M: Number 4 says it is not common, do we agree, is it not common or do other people have different experiences than number 4?

P005: It is not that common, ja.

M: Okay but they do come to say.

P005: Yes.

P001: What I have noticed is that it is mostly the patients who have got lipodystrophy they are the ones who are really more worried about their weight that’s one thing I have realised because they are saying, no, I no longer have my buttocks, they are gone, my legs are now thinner. So they are very worried and I have actually seen some patients wearing ((not clear sounds like default: tracksuits)) underneath because of this.

M: Okay, all right. If an HIV positive patient reports that they are losing weight, what do we usually do with them? (.) They have come to us and they have reported weight loss, what do we do?

P006: I’ll ask them why they think they are losing weight, because most of our patients don’t have scales at home. It is not an objective thing, and there is often another issue that they are dissatisfied with something else, and they report it as I am losing weight, I am losing power, I have got changes in my body, but it is not actually…, and I think there is possibly a degree of language barrier as well. English is not the first language of most of our patients but it is the language that we conduct most of our consultations and I think it is just something that patients say but they are not necessarily talking about weight loss per se. I always like to ask them what makes you think you are losing weight. Are your clothes getting bigger for you, have you seen changes in your body?

M: Okay so what you are saying is that in most instances if they come to us to report weight loss, one of the things that we ask is why they think they are losing weight, anything else that we do with them?

P001: We check to see if they are truly losing weight by checking their records and compare current with the ones which was done previously to see if they really are losing weight.

M: Okay, you check the records.

P007: We need to explain to patient that HIV is one of the causes of weight loss. We do a full investigation to find out what is the cause of weight loss.

M: I think earlier on, one of us indicated that they ask questions as to why they think they are losing weight, why do we ask them that?

P002: I think on our side it is important that if they think it is like dietary or if it’s like treatment that they can also identify, so it is not just us telling them all the time what they have to do when they have got problems, they need to be able to say it is because of this and they think it’s because of that. That also addresses the unintentional versus the intentional versus psychological.

M: Uhm okay.

P007: The nutritional status is very important because most of them say that they don’t have money to eat, they don’t have food to eat, so that is why they are losing weight.

P005: It may not be an objective weight loss, but maybe just a body perception problem they may see that they are losing weight, but not objectively, that is why it’s important to ask them why they think they are losing weight.

M: We said we also do a full investigation, why do we do that?

P004: To identify other underlying causes that could be contributing.

M: Okay, do we go to the extent of asking them about their personal circumstances?

P006: Yes, I think that is important.

P: Yes (majority).

P006: There is a lot of social issues, insecurity as mentioned, food security, social stressors, appetite, and the availability of adequate nutrition.

M: What about lifestyle, do you go to an extent of discussing the lifestyle or asking questions about their lifestyle issues?

P004: If they are gaining weight.

M: Ahhh, tell me more about that?

P: If they are gaining weight, we want to know if they are exercising or not.

M: Are we saying that we would be more concerned or we would only ask questions about exercising only if they gain weight?

P005: Unless it is intentional weight loss, then you know that is the underlying cause.

M: In some of the groups that we had with some participants, we don’t know how true this is, but we need your opinions or guidance on this. Some participants said that some HIV positive patients lose weight because they are not using condoms; they are drinking alcohol, they smoke as a result they lose weight. Is this possible?

P001: I don’t see what condom use will have with the weight loss.

P006: Unless they are getting themselves re-infected with the resistant virus ((All speaking at once)). I don’t think you can attribute that to condom use

P: What about alcohol and smoking?

M: Can we first start with the condom use; we finish with the condom and then get to alcohol use.

P006: No, I wouldn’t agree with that.

M: You don’t think that condom use may have impact at all?

P005: Not on weight loss.

P005: Not directly on weight loss, it’s indirectly it could affect the response to treatment or the resistance to treatment which could result in illness and weight loss because it is like they are not on treatment at all, but I think it is a bit of a far far step to go to no condoms and weight loss, there is stuff that happens in-between. That’s what I would say.

M: Oh and what about alcohol use?

P004: Not acutely, but if they drink chronically and that will be over year then that means over years, they develop cirrhosis but acutely no.

P006: Also possible with chronic alcohol use, there could be malnutrition just because if their money is going to be spent on alcohol then they can’t buy the food, lack of food security, but once again there are things happening in-between.

M: You said something along the lines of full investigation, you will do a full investigation, you will pardon me, I am not very familiar with the terms , I will try to understand and so when I keep asking for clarity, please do bear with me. When we were talking about the full investigation did we mean tests, were we including tests in that?

P004: Potentially.

M: What types of tests would we do, would we arrange for these people that have reported weight loss?

P007: We want to rule out TB, we might want to do an X-ray, chest x-ray, send a sputum, we might want to do an ultra sound scan for abdomen and then we have to look at other causes of weight loss like diabetes, hypothyroid, hypoglycaemia and TSH ((this part was not very clear)).

P: Certain malignancies.

P: Ja.

P004: We will send a patient for pap smear as well if a person is over 40 of 50 years of age, we will order a mammogram.

P006: I think the investigations will have to be guided by what you find during the clinical examination, there is no specific pattern on what you will order based on weight loss, that needs to be guided by what you will find, what you are told on the history and what you find on your examination, but I would agree with colleagues that some of the test you might order.

M: When number 4 talks about Pap smear, she reminds of previous groups that we had where some participants think that cervical cancer might be one of the tests that they will do as it might be related to the weight loss, is that true, is that correct?

P005: Any cancer.

P: Any malignancy.

M: Not just the cancer of the cervix?

P: No. Any.

P007: Cancer of the cervix is age defining, it is common that is why we do the pap smear, it is the common one.

M: We have mentioned the sputum test and you have mentioned the chest X-ray, the sonar and the ultrasound. What about blood tests, are there blood tests which we think we might arrange?

P005: We have to check the viral load, thyroid function, glucose and HBA1C

M: Please don’t laugh at me, I am learning ((Ms and Ps laugh)). Okay all right and in what circumstances will you arrange these tests, for instance the viral load?

P004: It is done routinely every six months.

M: The Thyroid function, under what circumstances?

P005: If the patient is complaining of weight loss and have symptoms of thyroid mal functions.

M: Which will include amongst others?

P005: Weight loss.

M: What about referrals, would you refer these particular patients?

P004: It will depend on the investigations and the results of that.

P005: We refer to the dieticians.

P: Ja, it depends.

M: As doctors you will usually refer them to dieticians.

P006: Also to a social worker.

P001: Psychologist, SASSA for the Disability Grant, ja.

M: These referrals that we are talking about, are they inter-departmental or do we have to refer them outside xxx (hospital name)?

P004: Inter-departmental.

P005: Except SASSA.

M: You don’t have social workers here?

P001: We do but they have to bring the forms and we have to fill them in.

M: Okay and what about pharmacist, do you sometimes refer patients to pharmacists?

P006: I don’t know if the prescription counts as a referral but I don’t think if we ever refer to pharmacists.

M: You don’t.

P: No, we don’t.

P001: We just send them to collect medication from the pharmacy.

M: The Vitamin supplements, do we sometimes]

P: [We do sometimes.

P006: We do.

M: Diet changes, do we sometimes discuss with them issues around changing the diet?

P001: Yes, but we usually send them to dieticians.

P007: If they have lipidemia, then we talk about dieting, but not about weight loss.

P006: If the problem is socio-economic, then I try to suggest cheap sources of protein and cheap sources of energy, often they are not getting the best of brand or whatever, we also give dietary advice.

M: Okay and what about follow–ups? As medical doctors and dieticians do we do any follow ups?

P006: I think anyone with documented weight loss, you would want to see sooner rather than later, you need to establish a trend and you need to get to the root cause, so I don’t think these are patients that you will send away for a long period of follow up, they will be seen sooner. So it’s a red flag.

M: Is what number 6 saying similar to all of us, do we think we all do follow ups?

Ps: Yes, we do (All).

M: How practical is it for us as a huge and a big institution like xxxx (hospital name) for us to be able to do these follow-ups? Is it practical, is it easy? Do we have a proper follow up system in place or something like that?

P004: Yes.

P006: This is a chronic clinic, so the patients are known to us, most follow up on a very regular basis so there is a system in place which support follow up and which encourages it.

M: Okay, that’s great, otherwise under normal circumstances, why do we think that HIV positive patients lose weight?

(.)

P004: It is multi-factorial.

M: Which factors?

P004: The HIV itself, associates with opportunistic infections, also because they are HIV, they are unable to work, which means they unable to get food into their house which means they don’t have food security. There is a psychological component to that as well if you are dealing with the disease itself. I think those are my top 4.

M: Any other reasons that come to mind? Why do they lose weight?

P001: Malnutrition.

M: Anything else?

P002: They are like other side effects associated with opportunistic infections, vomiting, diarrhoea and loss of appetite that is all associated with one of those 4.

M: I get you, on the groups that we have facilitated previously; stress seems to come out quite frequently as one of the causes of weight loss.

P001: It is a psychological, emotional factor.

M: How can stress actually make me to lose weight, if you were to explain that to a lay person like myself?

P007: You have loss of appetite if you are stressed.

M: Why do you think HIV positive patients report weight loss?

P001: Stigma as well, you see people are worried, they think that if they are losing weight then they are going to be labelled as this patient is sick and people will see that they have HIV, so that is why they are so worried about weight loss. I think there is also stigma.

P005: A lot of them had TB previously and lost weight now they are trying to gain so they are worried they might have TB again, they know that weight loss is associated with TB.

P006: There is a general awareness that you don’t want to lose weight, they get very worried.

M: For instance if they come for their consultations and they come to us and they say doctor or dietician I have lost weight, in most instances, what do they say it is that is making them to say that they have lost weight?

P004: Their clothes feel looser.

P005: Their family members might have told them that they are getting thin.

M: Comments from significant others, yes? What else, what will make me to come to you and say that doctor I have lost weight? What would I have seen in me that could make me to come to a conclusion that I have lost weight?

P004: Their appetite is not good or has changed; they think that they have lost weight because they are not eating the same amount they used to eat.

M: If I was not eating well before my visit, okay, what else?

P003: They have lots of vomiting or they have episodes of diarrhoea.

M They have been feeling sick, yes?

(.)

P006: Many are aware of their body shapes and they feel it has changed due to weight loss.

M: Their interpretation of body shape changes, okay, all right. I think we had already started talking about stigma and weight loss, in most instances do we think that there is stigma attached to people losing weight?

P001: It will depend on what amount of weight you have lost.

P005: People always attribute the stigma of weight loss with that people are either HIV positive, even though there may be other causes they just feel that if they are losing weight, they are HIV positive.

P002: That is because of the stigma in the community.

M: Okay, all right, welcome back, we are talking about stigma and I think there is one thing we did not mention whenever your phone rings feel free to go outside and respond to it but if possible, let’s try and put them on a silent mode so that when it rings and stuff like that they do obstruct the free flow of our conversation. We are talking about stigma right now; we are asking if at all there is any stigma attached to people losing weight, we are asking what have patients shared with you with regard to that?

P006: Just one other thing that I have more than one patient comment on is people who are casually employed if the employer sees that they are losing weight, they may lose their jobs because the employer thinks that they are HIV positive.

M: Those in casual employment might lose jobs. Okay and what about them being called names, we have heard some of the patients say that when they have HIV and once they start losing weight, some people have particular names that they call them with stigma attached to it, have we heard of that at all?

Ps: No.

P006: Not really.

M: Other than what the doctor has shared with us that some people might lose employment if they lose weight, is there anything else that you might say, our patients have shared us this with regard to weight loss and the stigma?

P003: A lot of them worry about the changes of body shape when they have been on medication for longer, those changes on the abdomen gaining more weight surrounding those areas and also losing fat on their face.

M: is there any stigma attached to that, are they laughed at once they lose perhaps fat on their face?

P002: There is stigma. We are further down the line, we are like a referral level that we will also ask why are you losing weight, for us they have lost weight and we can see that and if we ask why, often the response is that, they are on treatment, they have got HIV, it’s like all there is, it’s like that is why, so that’s often their response.

M: Okay, all right and then if a person is attending a clinic for HIV, how do you think their weight should be? (.) They come to the clinic, they get their medication, they get their treatment, they get counselled and stuff like that, how should their weight be?

P004: I think it is difficult to say that that they should be at an ideal BMI because we need to know what their starting weight is, as long as they are improving from their starting weight I think that will be an acceptable…, because you can’t say that the patient has a normal BMI because it is a bit …(not clear).

M: So?

P004: They should improve from their previous amounts.

P002: If they were under weight, not everyone. If when they started treatment they were at a normal body weight, then you don’t want them to gain weight or to become overweight.

P003: You want them to work towards their ideal body weight, if they were underweight, you want them to try and gain weight, the appropriate weight, if they are overweight then you want them to potentially lose.

M: When we talk about the ideal weight, what do we mean?

P003: BMI of less than 25 and above 18, between 18 and25 (Can’t make sense of what she says).

M: So you are defining the ideal weight in terms of BMI?

P003: Yes.

M: Let me see if I understand you clearly, you are saying that if they came to us when they were underweighted, once they are on ARV’s or attending HIV clinic, you expect them to improve a bit from their previous weight and then if they were on ideal body weight, you expect them to remain there. Is that what we are saying?

P005: Yes.

P003: Ja.

M: Oka and what about body shape, if they attend the HIV clinic, how do we expect their body shape to be?

M: Okay, maybe to facilitate this, let me pass around these silhouettes for us to look at the pictures and perhaps show them to one and then have our discussion revolve around them.

P006: Are you asking what we have seen in our experience, what the reality is or are you asking what we would want for our patients?

M: We are asking you what should be happening if we are talking about the ideal body weight, what should it be? If I am attending the ARV clinic, what should my body shape be? What should I look like? ((Ps start discussing silhouettes and they laugh on these side conversations)) can we send one so that each and every one of us will have something on their hands, we can perhaps show one another what we have and then should you think that you have a picture which you think is the ideal body shape then let’s hear what other people think of that. (.) ((Ps start side conversations)) Let’s have one discussion please. Okay we have passed these around and I can see already, I am very fascinated about this, it seems to me you have already started discussing this, let’s hear what we are saying amongst these that we have in our hands ((Ps laugh)) what is the ideal body weight and why? Number 2 and 3, please what are you saying?

P003: we are just discussing this picture here.

M: Which one?

P003: It will be like the middle of your BMI.

M: What’s the number?

P003: Is there a number?

M: At the back.

P003: Oh, it’s number 5.

M: What are we saying about number 5?

P002: I think it’s a good BMI.

P003: Yes.

M: Did you choose the same one?

P003: Yes, 5.

M: Really, interesting and what about number 6 and 7? Did we choose something similar?

P: We didn’t agree. I went with this one, number 4 and *((name withheld for confidentiality reasons)) went with number 3, she says that is what she wants to be. ((They laugh at number 3)).

M: So we have chosen number 5, 4 and 3.

Ps: Yes.

M: Why do we think that number 5, is ideal weight and shape?

P002: 6.

M: You are choosing 6?

P002: No, it’s 5. It looks like he has a healthy BMI.

P003: It looks like number 5 has a healthy BMI. Okay, why did we pick number 5? What else can we say about number 5?

P001: Flat tummy, he seems to have biceps, ja.

M: You are indicating to me that it’s muscles on arms because my recorder cannot get that, yes. (.)

M: You chose number 4. What made you choose number 4?

P006: To me, it looks like some healthy middle ground, not too fat not too thin. I don’t know what else to say.

M: Okay and what about number 3? I am told you liked number 3 ((P003 laughs)) what makes you think that number 3 has an ideal body shape and body weight?

P007: I mean it’s not too fat and not too thin.

P: But the legs are thin.

P007: But all the legs are like this, so I don’t know. Look at this ((they laugh uncontrollably)).

M: Number 5, 4 and 3. If for instance we are saying that the treatment was going well, would we think that the person should look like number 5, 4 and 3?

P006: No, no]

P003: [It depends.

P006: Ja, it depends

P003: Because some people even before they are even HIV positive, maybe they were never on that BMI. I had a lady that was referred to us to gain weight so when I spoke about a BMI of 25, she literally laughed at me because she was never a BMI of 25, so it depends where the starting point was.

M: So are you saying that her BMI changed for the better?

P003: No, she has always been a skinny lady, so even before she was HIV positive, she was never a BMI of like in the 20s, she has just always been a tiny lady, so yes, she has lost weight because of the HIV, but even if I do ask her to gain weight, I need to know the amount of weight she was.

M: It looks like 7 and 6 wanted to share something with us?

P006: We were just re-iterating that it’s dependent on where the patient started. If they started at a healthy weight, then we don’t want them to gain weight, but if they started overweight, then you might actually want them to lose weight. So it’s dependent on each individual.

M: What if the treatment was not going well, what would we expect on that particular patient?

P007: You have to check their viral load, that is how we will monitor if the treatment is working or not. So the viral load should be suppressed, if it is not suppressed, then it means treatment is not working for that patient or that that is not taking it and then the other things is that people can get opportunistic infections if the treatment is not working and the patient will get sick, those are the things how we monitor if the treatment is working or not..

M: Okay, all right, that is when the treatment is not working well. Anything else?

P006: People mention several times that there could be changes in body shape as a complication, I would consider that treatment not going well if the patient is getting side effects, it might prompt me to change the regimen.

M: Okay, we will get to body shape changes after this.

P007: Actually, with Stavudine if they have lipodystrophy, it means the patient is compliant to treatment, the patient is taking treatment, is complying, that is why they are getting this side effect of lipodystrophy.

M: Hmmm, so once you see them with lipodystrophy, then you know that they are indeed taking the treatment.

P007: The patient is compliant to treatment on that regimen.

M: This is very interesting. Who regard it as being effective, is the patient or the health care worker?

Ps: The health care worker.

M: What about men versus women, would we say that there is an ideal body weight for women and an ideal body weight for men or would we always refer to our BMI?

P003: Refer to BMI.

Ps: It’s BMI.

P: It’s always BMI.

M Is it BMI all the way?

Ps: Yes.

M: These pictures that we just handed out to you, which one would you have regarded as the most attractive ones?

P006: It’s the same one I picked earlier on ((Ps agree with one another and they laugh)).

M: Number 4 is going with the number 5 that she chose earlier on.

P006: Number 5.

M: You are also choosing number 5.

P007: I am sticking with 4.

M You are also sticking with 4. Or you changing from 3 to 4, you think that it is the most attractive.

P: Ja.

M: Okay and why do you think that these are most attractive?

P006: They look healthy.

M: Anything else other than them looking healthy on the eye? ((P001 laughs)). (.) Okay these opinions that we have, what is it that has informed our choices? You chose 5, you chose 4, you chose 3, and now you are choosing 5, 4 and 4, what informs our thinking with regard to body shapes and body weight? (.) Is it informed by culture, community, media, our discipline, the professions that we are in or what?

P006: I would like to say that we are professionals and we know what is healthy, but I think in truth is probably largely affected by popular culture]

01: [Ja.

P006: and what we are exposed to in the media.

M: So it will be culture, the media and our profession.

P006: I hope so.

M: That’s what number 6 think, what do others think?

P005: I agree with what she says.

M: What about our family? Would you say that when we were growing up we were told this that and that?

P004: But then our families’ perceptions are also influenced by culture and media.

M What about the community, where we come from?

P005: Ja, that also.

P006: Ja, I think that does.

M: Why do you think HIV positive patients would report that their weight has changed, but when you put them on the weighing scale, the weighing scale doesn’t show any weight changes at all?

P007: I think it is psychological, they think that they have got HIV, they have got a disease and they think they are losing weight and it’s psychological.

P005: The fact that there is stigma with HIV and the fact that they have got HIV, they expect to be losing weight.

P006: It is something that they are really worried about, so they are very sensitive to it.

M: Anything else that would make them say that they have gained or lost weight even though the scale does not confirm that which they report?

P006: I think sometimes if they are dissatisfied with another aspect of their care or their treatment, uhum.

M: Tell us more about that?

P006: Maybe they are experiencing some side effects from their treatment or they are just not feeling well constitutionally.

M: Not feeling well.

P006: I often find that when you ask more, when they have complaints, the first thing they will tell you, if you ask them if they are having problems, they will tell you that they are losing weight, but then if you keep on asking questions, you actually get the whole lot of other complaints and problems.

M: Hmm, okay. Under normal circumstances, what do you think losing weight means to a patient that is attending HIV care, if they ever share that with us?

P004: A lot of them are fearful that they are extremely ill.

P006: A lot of our patients have been very ill when or before they started treatment and I think many of them are afraid of going back to square one basically. Anyone is going to be anxious if you think there is something wrong with you that you are sick anyway.

P004: They fear for TB as well because TB and HIV are associated not only by the hospital but by the community as well, they are scared of TB.

P006: A lot of people actually request TB testing.

M: In some of the focus group discussions we’ve had, some participants have shared with us that should they be losing weight and they are attending HIV clinic, it means that the treatment is not working. Is that true, have you come across patients that would say that?

P005: I think some patients do have that perception.

P006: Yes, some think that the treatment is not strong enough for that person, if they are losing weight. Even if in reality it’s another problem.

M: Have we come across that, when patients who are attending the clinic, are losing weight, and they say the treatment is not working?

P002: No.

M: Okay, all right and then if the patient reports weight gain but the weighing scale does not confirm that, why do you think patients will do that? Why does that happen?

P004: I think psychologically they want to feel that they are getting better.

P006: If they have noticed an increase in their appetite.

M: Anything else? What would make me come to your clinic after having taken ARVs; I come and say I have gained weight? What would I have noticed that could have made me to say that I have gained weight?

P001: Maybe the clothes cannot fit.

M: The dress size issue.

P005: The body shape might have changed.

P001: Ja.

M: In general, what do you think gaining weight means to a patient that is attending an HIV clinic?

P001: It means that that they are getting better.

M: Okay, anything else?

P007: Sometimes they think that it’s the side effects of tablet that they are gaining weight.

M: Okay, if you say it’s a side effect, will it be for those who do not like to gain weight?

In your experience as healthcare professionals, what do you think is the best way to ask people with HIV if they are losing or gaining weight? (.)

P004: Just ask them straight out, are you losing or gaining weight?

M: How do you usually ask this?

P004: Are you losing or gaining weight?

M: Is that what we all ask?

Ps: Yes.

M: Do we have those who ask it in a different manner?

P007: I check the weight.

P006: If I see that the patient doesn’t understand, then I might try something more around like, have you noticed that your clothes don’t fit you as well as they used to before? Something like that but I think mostly, you first try and ask directly and of course we measure it objectively as set at our clinics.

M: Do you think that the patient will understand if we were to ask them this question “Have you lost more than a dress or trouser size unintentionally in the last 6 months”?

P005: I think they will understand.

M: You think so?

P004: Yes.

M: What does this question mean? I brought along some pants and some skirts, if we could perhaps have a volunteer that could show us by these pants’ and skirts’ sizes what we mean by losing more than one skirt size? Can we perhaps have people that will indicate to us what we actually mean? (.) Maybe let’s start with the skirts.

P001: From 34, 36, 38.

P006: So if you lose 38-36 that is one dress size.

P001: Ja that is what I am saying.

M: From 30?

P: 8 to 36.

M: 38 to 36.

Ps: That is one size.

P006: So if you lose more than that that is what she is asking.

P001: 38 to 34 that is more than one dress size.

M: Okay, our understanding is from 38 to 34, that is more than one dress size.

P: 38 to 36, it’s one dress size.

M: Okay, great and with our pants over there? I think we have 28 to 32.

P006: If they go from size 32 to 28, that’s more than one trouser size.

M: Okay, great, thanks for sharing that with us. Do you think our patients understand “unintentionally”?

P006: We do need to clarify.

P002: You might not say the actual word ‘unintentionally’, you might say ‘without trying’ or to want to lose weight or have you tried to lose weight?

P005: Or are you dieting, are you exercising?

P002: So we need to make sure that they understand.

M: So you will not just say unintentionally, you will go to the extent of explaining what you mean by unintentional.

P004: Yes.

P007: Like if I see that a patient who was 70 and is now 65, I always ask, are you trying to lose weight? Most of them will say yes, but if they are losing unintentionally, then they would complain.

M: Okay, hmmm. Otherwise do you have an alternative suggestion of how we can actually…, of a question that we can have, that will help us get an accurate answer as to whether our HIV positive patients have lost or have gained weight?

(.)

If we could perhaps get an alternative question that can help us get an accurate answer, a question of whether they have lost or they have gained weight?

P003: Without weighing them? Or is this now in a place of weighing them?

M: It maybe there is a scale maybe whatever question we come up with but we will still have a space for a weighing scale to confirm.

P002: I think like we said before: Has your dress size changed, could be the alternative question.

P: Ja.

P006: Or are your clothes you have no longer fitting you well?

P007: And then you can ask sometimes, if anyone, your friend or family complained that your body shape has changed and if you have lost weight as well?

P001: Or just ask them directly, do you think you have lost weight, if so why?

P007: This whole thing of going round and about is more confusing for everybody.

P001: Ja. Do you think you have lost weight?

M: So we are direct and in addition to asking those direct questions we then get them to the weighing scale.

P006: Ja.

P007: Especially the changes in their body shape, they always deny, most patients deny it, no, I am fine. So then I ask them, did anyone in your family or friends say that your body shape has changed? I have always that that works better.

M: Okay, all right and now that you have already started talking about body shape changes, you have actually brought us to our last section of our discussion. When an HIV positive patient attends a clinic for appointments, do you ask about their body shape changes or do you recommend if you see or notice something?

P005: I only ask if they are on Stavudine and/or if I notice that they have an abnormal body shape.

M: When we say an abnormal body shape, what do we mean?

P005: Like a buffalo hump or thin face like features of lipodystrophy.

P006: Big stomach but that is very common ((says 01 with a chuckle)).

P001: Thin legs.

M: Number 6 speaks as if a big tummy is very common?

P001: It is common (laughter).

P002: Big tummy is common and it’s very big problem.

M: You guys are laughing. Okay, so you do ask buy you are saying you only ask under these circumstances, if you notice these changes. When you ask, how do they usually respond?

P007: I have noticed that some of the patients don’t realise it, but when you ask them, have you realised that your legs have gone thin, your face has become thin, then only they will say I have noticed that. When you ask them for how long, maybe for two years so why didn’t you tell, “I didn’t know it was the side effects”, you know but we always ask. We don’t see the patient like follow up the same patient you know sometimes the other doctors see the patient but usually we don’t see the same patient but we don’t know everything that happens. But we ask them especially if they are on Stavudine or Alluvia or regimen 2, we ask those questions, even their blood for cholesterol and other things on the patient, but the thing is some patients don’t realise it, only when you ask them they will say I have noticed that but some patients they come to you to complain that my breast is getting big and my legs are getting thin, and they complain about their body shape changes.

M: So the reaction will be that for some of them you have helped them realise the condition.

P007: Yes.

M: How do other patients respond?

P006: I have had patients who flatly deny it, you can see that they have signs of lipodystrophy, but they say no, they have always been like this. Even if you show them their own ID photo, they won’t sort of accept it or admit it and some patients don’t want to change their treatment regimen, even if they are experiencing those side effects, they will just say no, I am fine.

M: That’s very interesting to know.

P006: Not everyone, some people are bothered by it, but some people who are affected quite severely I think, don’t seem troubled by it and they don’t want to try and change it.

M: Are we saying that it is only those that are severely affected that will come forward and request the change in the regimen or are we saying that there are some that just want to stick to their treatment?

P006: No, I am saying that there are some who want to stick to their treatment despite the doctor actually saying to them you are getting these side effects, it is going to become a permanent, don’t you want to change, and they say no.

M: Hmm.

P006: But it is depended on the individuals, some people are very sensitive to it and they will come to you with their buffalo hump and it will be quite subtle but it really really bothers them and for other people the changes are drastic, but they don’t seem to be troubled by it. So I think it dependent on people’s own body image and their own personality. Ja, it’s very much dependent on the individual.

M: Do you sometimes get patients that will come to us to consult; they will leave their homes to come to you just because they have noticed body changes?

P001: They do come.

P: Yes.

P: Yes.

P002: Yes.

M: What do you usually do when they come other than changing the regimen like you have mentioned earlier on?

P001: For me, I had two who have come and they are on second line and in that case they don’t have many options because they have failed their first line, and they are on this regimen so that is what they are supposed to be on. So sometimes there are more options, sometimes there is no way out.

M: What do you do?

P005: You counsel them.

P006: You just assure them.

M: When you say that you reassure them, what do you mean?

P006: You explain to them everything that she has said.

P: Ja, ja.

P006: That there are no alternatives.

P001: You can’t go back to what you were taking before because it won’t work for you.

M: Oak and referrals, do you ever refer people that are experiencing body shape changes?

P007: Plastic surgeon for buffalo hump.

P001: Some get breast reductions done.

M: Breast reduction, is that not expensive?

P001: They have a list, I think.

P006: It is sponsored, it’s done by plastic surgeon so it’s part of the government health provision, but the waiting list is very long, but it is done as a government procedure.

M: Okay, I used to think you need to have a lot of money to do that.

P001: In private of course it will be very expensive, but they are doing it here by government and its long list.

M: Number 7 you wanted to say something?

P007: I am saying, these are the patients not on Stavudine, not on regimen 2 and they also complain about body shape, so we just counsel them that HIV itself can cause lipodystrophy. So they have to change their lifestyle and exercise, modify the diet and other than that we send them to dieticians, so because the first regimen now doesn’t have that many side effects of lipodystrophy, but HIV itself can cause lipodystrophy.

M: Oh, it can?

P007: Yes, it can. ((M allows another M to facilitate)).

M: Uhm I didn’t know that. Okay, all right, in general can you describe the changes between men and women, like how would a man look like versus what a female would look like in terms of body changes?

P006: Are we talking about on or off treatment?

M: On treatment?

P006: There is not any difference, except breasts.

P001: Ja, some men can have breast.

P007: I haven’t seen buffalo humps in men, it is mostly in females.

P004: Men get gynecomastia.

M: What is that?

P005: Breast development in males as a side effect.

P006: Just in terms of not related to side effects in males as they improve, you will just expect them to increase their muscles mass, their lean mass because they would be very wasted in HIV and I think you will expect a male to gain more muscle bulk than their female, I think that is a gender specific side of HIV.

M: Generally, how do they feel like, for example you have mentioned that a man will for example develop breast, what does that make them feel like?

P001: They don’t like it, most of them don’t like it. I had I think two referrals from clinic, we just tried to explain that it’s a side effect and stuff like that.

M: I am sorry, ((says another M jumping in)) We have heard instances with some of our focus group discussion participants who would say that some family members will comment on these negative shapes as a result of ARVs and then they will influence the person that is taking ARV’s to stop taking ARVs. Do you come across that?

P001: Somewhat, I came across that, one lady who was on Stavudine for 7 years, unfortunately she was not taking her treatment well and then developed a biological failure but then and she was also complaining about lipodystrophy. So she went to the clinic and told them that this medication is not good for me, look at my body but then they couldn’t change her then because she was failing the treatment, so she ended up giving up and then she just stopped taking treatment because she felt that they were not helping.

M: You said she had a failure, what do you mean?

P001: Her viral load was very high, so they couldn’t change her then before they could change her; she just stopped taking her treatment.

M: But is she alive or fine if you know?

P001: Yes, she is. I have seen her.

M: Have patients shared with you reactions of stigma from people commenting about their body shape? Maybe things that the community will say with regard to their body shape or family members or spouse?

P005: Not really.

M: But do you ask the]

P001: [I have one lady who and said doctor, I don’t like my body anymore, even my husband is commenting, I cannot look at myself in the mirror, you know. So for some people it can be that bad, ja and it just depends and one lady now wears 3…, she no longer wears dresses, she wears jeans only and she puts like two layers of leggings before she wears a jean so that at least her legs can look that big, ja.

M: Some ladies have reported thin legs and develop calf muscles which they didn’t have before?

P006: I think that comes from prominent because there is fat loss, so that shape looks more of a masculine lady shape

M: In terms of their facial look?

P006: It can be disfiguring, lipodystrophy has a skull like face.

M: What about stigma as in from the healthcare workers, from doctors, nurses, counsellors, when you see them what do you say?

P006: I don’t think there is stigma from health professionals, we actually give support other stigmatise the patients.

M: But does that apply to only doctors or everyone in the health profession?

P003: Actually, we encourage them as patients.

P006: You want to get information from the patients so you don’t want to make anyone feel uncomfortable. So I think most professionals will try and make them feel comfortable.

M: What about children? We have had patients that will say things along the lines of their children commenting about their body shape.

P001: Not really.

P004: Not that I know of.

M: What about the wider family or extended family, like your aunt, uncles, people that they interact with, do they tell you what they say about them?

P006: We don’t know about that.

P004: Maybe we don’t get to ask them about that.

P007: From friends, like I will hear from my domestic talking about her friend, she will say, someone is losing weight, maybe she has HIV.

M: So they will comment more about body weight. Have any of your patients shared with you how the stigma makes them feel?

P007: Not like that, we refer them to the clinic, and when we refer them to the clinic, most of them, they say no because it’s the HIV clinic because everyone knows that and they don’t want to go there because at the clinic, there is separate area for HIV and separate area for the normal patients without HIV. They say we don’t want to go there because then they separate you from the other patients and then everyone knows that you are HIV positive.

M: It’s sort of they will just see, okay, any other stories they tell you?

P007: They don’t want their employers to know that they have got HIV because they are scared they will lose their jobs.

P006: Like if they are late for work when you write a note for them, they often ask, you not saying anything about my status; it’s something that people are private about. People who are private about it, they don’t want a lot of people to know still.

M: What about you, from your side as xxx (position)?

P003: We don’t really get to talk that much about the social stuff with them so all we usually get is that they are referred for weight loss or weight gain, so we don’t really get into the stigma and deep personal stuff.

P002: Unless they offer to tell us themselves.

M: Okay, on that note, we are done then. Thank you so much for your time, we are done.

M: We appreciated your input. The time is now 14h27

Focus Group discussion ends
