# Supplementary material for: “If we lose it, we are worried”: Individual and provider level perceptions towards weight change among people living with HIV who undergo TB screening in routine health care settings in Gauteng Province, South Africa
Source: PLoS One. 2025 Sep 22;20(9):e0331904. doi: 10.1371/journal.pone.0331904 (PMC12453174; doi:10.1371/journal.pone.0331904)
Supplement: Attachment — Submitted filename: PONE-D-24-15618_R1_reviewer.pdf [file pone.0331904.s007.pdf]

PLOS ONE

“If we lose it, we are worried”: Individual and provider level perceptions and attitudes towards weight change among people living with HIV screening for tuberculosis in routine health care settings in Gauteng Province, South Africa.

--Manuscript Draft--

Manuscript Number:	PONE-D-24-15618R1
Article Type:	Research Article
Full Title:	“If we lose it, we are worried”: Individual and provider level perceptions and attitudes towards weight change among people living with HIV screening for tuberculosis in routine health care settings in Gauteng Province, South Africa.
Short Title:	Perceptions and attitudes towards weight change among people living with HIV in South Africa.
Corresponding Author:	Tanyaradzwa Nicolette Dube, MA<br>The Aurum Institute<br>Johannesburg, SOUTH AFRICA
Keywords:	HIV, TB screening, Weight change, ART, South Africa, WHO screening tool
Abstract:	Background<br>HIV weakens the immune system, increasing the risk of tuberculosis (TB) in people with HIV (PLHIV). People living with HIV and on antiretroviral treatment (ART) often experience physical body size changes. Studies have found a significant discrepancy between PLHIV’s self-reported weight loss and their measured weight loss when being screened for TB using the WHO tool. To understand this inconsistency, a qualitative sub-study was conducted to explore perceptions and attitudes towards weight change among adults attending HIV care, as well as health care workers in public clinics in Gauteng, South Africa.<br>Methods<br>Our qualitative study was nested within the XPHACTOR study. A total of seven focus group discussions were conducted, five with adult participants attending for HIV care and two with health care workers and research staff in clinics around Gauteng. Inductive thematic analysis was used to analyse the data.<br>Findings<br>The majority of PLHIV preferred to gain weight due to fear of stigma associated with weight loss. Weight loss is associated with HIV/AIDS, suggesting that people attending HIV care may underreport weight loss in the context of a TB symptoms screening tool because they fear stigma. Participants reported that weight changes impacted their daily lives and had psychological effects on them. Some PLHIV described lipodystrophy as disproportional weight gain. Culture and media have an influence on the perception of ideal body size and shape for both men and women.<br>Conclusions<br>Underreporting weight loss might result in poor sensitivity of the WHO TB screening tool and suggests that we need either alternative ways to determine weight loss or screening tools for TB that are less dependent on reported symptoms.
Order of Authors:	Tanyaradzwa Nicolette Dube, MA
	Fezeka Mboniswa, BPsych (Honors)
	Samkelisiwe Ethel Qwana, MA
	Salome Charalambous, PhD
	Nontobeko Ndlovu, Msc
	Alison D Grant, PhD
	Yasmeen Hanifa, PhD
	Johannes Machinya, PhD
Response to Reviewers:	Editors Comments<br>1.Please ensure that your manuscript meets PLOS ONE's style requirements,

Powered by Editorial Manager® and ProduXion Manager® from Aries Systems Corporation

including those for file naming.

Response: Agreed. This has been done.

2. In the online submission form you indicate that your data is not available for proprietary reasons and have provided a contact point for accessing this data. Please note that your current contact point is a co-author on this manuscript. According to our Data Policy, the contact point must not be an author on the manuscript and must be an institutional contact, ideally not an individual. Please revise your data statement to a non-author institutional point of contact, such as a data access or ethics committee, and send this to us via return email. Please also include contact information for the third party organization, and please include the full citation of where the data can be found.

Response: Agreed. Data has been submitted with the revised manuscript.

3.Please upload a copy of Figure 4 and 5, to which you refer in your text on page 12 and 18. If the figure is no longer to be included as part of the submission please remove all reference to it within the text.

Figures with Stunkard body image figures have been added to the manuscript (Fig1 and Fig 2). The figures have male and female body image figures ranging from 1 to 9 and participants were choosing the image they preferred.

4.Please include captions for your Supporting Information files at the end of your manuscript, and update any in-text citations to match accordingly. Please see our Supporting Information guidelines for more information:
http://journals.plos.org/plosone/s/supporting-information.
Response: Done

5.The work submitted is noted. Could the authors address the constructive Reviewer comments?
Discuss the significance of the study findings in today's terms and add the relevant practical and policy implications the data is old so please justify, especially noting the chronicity of HIV in South Africa
Response: Our study findings are significant in today's terms as South Africa still has a high estimated HIV prevalence rate of 17.8 among adults (15-49 years) and 75% of PLHIV are on ART(23). TB remains a leading cause of death among PLHIV so understanding their perceptions around weight change and how this may affect TB symptom screening is important. In addition, since this work, the new first-line treatment regimen for PLHIV includes dolutegravir, which has been found to have weight gain as a side effect. Hence, perceptions on weight gain highlighted in our paper remain relevant (18,24).

Page 20 line 431-436

The policy implications of our paper suggest that in addition to TB screening, we should continue using innovative TB testing strategies such as Targeted Universal Test and Treat (TUTT) which tests high risk groups including PLHIV irrespective of reporting TB symptoms so that PLHIV with TB are not missed due to inaccurate self-reporting on TB symptoms. See line 458 page 20

Reviewer 1
The aspect of weight loss in relation to the tool used for screening tuberculosis seems to appear only at the background. Considering the relationship between HIV/AIDS and TB, as well as the high chances of people living with HIV/AIDS to contract tuberculosis due to the compromise of their immune system, it would be nice to have it appear up front even from the title level. This will certainly give a sense of concern to the people living with HIV/AIDS and the "why we should worry" about their perceptions and attitudes on any weight change particularly the weight loss.

Response: Agreed. The title has been revised to include TB screening among PLHIV.

Powered by Editorial Manager® and ProduXion Manager® from Aries Systems Corporation

	“If we lose it, we are worried”: Individual and provider level perceptions and attitudes towards weight change among people living with HIV screening for tuberculosis in routine health care settings in Gauteng Province, South Africa. Also the risk of developing TB among people living with HIV was included on page 4 (line72-73) in the background<br>2. Lastly, on data availability and restrictions, if the author indeed de-identified the transcripts with a unique study number, then there should be no fears of bridging confidentiality if made public.<br>Response: Agreed. Data has been submitted with the revised manuscripts<br><br>Reviewer 2<br><br>1. this is misleading. your population comes from one part of the country (Gauteng) ..so your tiltle should reflect this. Are these findings generalizable ..then i wont have a problem<br>Response: Agreed. The title has been changed to reflect Gauteng Province<br>“If we lose it, we are worried”: Individual and provider level perceptions and attitudes towards weight change among people living with HIV screening for tuberculosis in routine health care settings in Gauteng Province, South Africa.<br><br>2. Line 125<br>Research Assistants and HCWs were conducted via email<br><br>do you mean contacted? If not, then this sentence does not make sense!!<br><br>Response: Yes. We meant contacted. This has been edited on page 6 line 126<br><br>3. Quote on line 236:<br>When I thought about the scale I was so excited because I have been weighing 152, so I had to hit the gym whatever it took. So today when I thought about coming to the scale I was excited. I wanted to see if this 152 had gone down. ((They all laugh)). When I got there I found out that it had gone down it’s 62.5. and I was happy (FGD 3, Females).<br><br>Reviewer’s comment: not sure if this is kgs ..also the drop in weight then is significant!<br><br>Response: Yes, the participant meant kg’s. We agree that the weight loss reported her was massive and we quoted a participant who may have gotten the figures wrong. We have decided to use a different quote to support the finding that other participants had a positive attitude towards weight loss on page 12 line 244<br><br>I was happy today because I had lost a lot of weight (FGD 3, Females).<br><br>4. This sentence contradicts what you have been saying all along that PLHIV under-report weight loss but here you are saying that they report weight loss even though they have not lost weight!!!<br><br>Response: We agree with the reviewer that the data primarily support the idea that PLHIV are likely to underreport weight loss and have removed the two sentences in the discussion that talk about overreporting.<br><br>5. So maybe somewhere in your manuscript you need to highlight the probability of TB diagnosis in PLHIV.<br><br>also is weight loss a function of the drug or a symptom of TB in these patients or both?<br><br>Response: The risk of developing active TB disease is estimated to be 16 (uncertainty interval 14–18) times greater in PLHIV than those without HIV infection. (WHO, 2023). The statement has been added to the background line 72-73.<br><br>Line 198-204 highlight participant’s perceptions on possible causes of weight loss which includes side effects of medication and TB disease. Line 246 refers to the idea that ART might cause weight loss. A quote has been added to support the statement regarding TB causing weight loss.

Powered by Editorial Manager® and ProduXion Manager® from Aries Systems Corporation

	“I am saying, another thing that can make a person to lose weight is that because I am HIV positive, I must check whether I have TB or not because that also causes a person to lose weight” (FGD 3, Females).<br><br>6. Line 386 Statement: A potential response could be to weigh PLHIV instead of asking them the question on weight loss<br><br>Comment: Surely this applies to anyone from this community regardless of their HIV status?<br><br>Response: Agreed. This has been changed to: A potential response could be to weigh people seeking health care services instead of asking them the question on weight loss. (Lines 388-389) page 18<br><br>7. Statement: It is important to educate PLHIV that excessive weight gain may lead to obesity and increase the risk of non-communicable diseases<br><br>Comment: surely here a better measure would be BMI. the researchers had the opportunity to weigh the participants. i am not sure if the height was taken. if so a BMI could have been calculated and then the quest of excessive weight gain would have been easy to recognize in the context of a BMI score<br><br>Response: Yes BMI was calculated as part of the study procedure. In our paper, we recommend educating PLHIV about the negative effects of weight gain which they perceived to be favorable from the qualitative findings. Page 18 Line 400<br><br>8. Statement: The psychological impact of weight changes leading to non-adherence found in our study has been reported in other studies[7,12,20].<br><br>Comment: where is this reported and non-adherence to what?<br>Response: It was reported on page 12 (lines 245-253). Some participants reported that they would consider stopping treatment if they kept losing weight. We have clarified the treatment they would consider stopping which is antiretroviral treatment (line 247).<br><br>9. 9. Line 408 It is important to show the outcomes of similar studies in different settings or cultures and comment on these in the context of your findings.<br>Response:<br>The discussion section (pages 18-19) refers to studies conducted in other parts of South Africa like Cape Town (lines 415-422) and Durban (lines 411-414) as well as studies on weight perceptions conducted in other countries such as Tanzania (lines 406-409) and Uganda (lines 378-481).<br><br>10. How confident are the researchers that asking about a change of dress size can be used as proxy for weight loss or gain. what if they simply dont answer truthfully to this question? Are there studies that have shown dress size to be a reliable proxy for weight gain or loss? Studies<br><br>Response:<br>Since this was a qualitative study, we asked about perceptions of weight loss. Participants in our study reported that changes in dress sizes made them realize they lost weight. HCWs in our study recommended that asking participants changes in clothing size could be better than directly asking unintentional weight loss questions. Asking clothing size is a self-report measure which can get biased responses, but we feel is more acceptable to patients rather than directly asking about weight loss. A study in Netherlands found that self-reported clothing size appeared to predict cancer risk independently of body mass index, suggesting that self-reported clothing size can be a useful measure to consider in epidemiologic studies.<br><br>Hughes et al (2009) Self-reported clothing size as a proxy measure for body size
**Additional Information:**	
**Question**	**Response**

Powered by Editorial Manager® and ProduXion Manager® from Aries Systems Corporation

**Financial Disclosure**<br><br>Enter a financial disclosure statement that describes the sources of funding for the work included in this submission. Review the submission guidelines for detailed requirements. View published research articles from *PLOS ONE* for specific examples.<br><br>This statement is required for submission and **will appear in the published article** if the submission is accepted. Please make sure it is accurate.<br><br>**Funded studies**<br>Enter a statement with the following details:<br>• Initials of the authors who received each award<br>• Grant numbers awarded to each author<br>• The full name of each funder<br>• URL of each funder website<br>• Did the sponsors or funders play any role in the study design, data collection and analysis, decision to publish, or preparation of the manuscript?<br><br>Did you receive funding for this work?	Yes
Please add funding details.<br>as follow-up to "**Financial Disclosure**<br><br>Enter a financial disclosure statement that describes the sources of funding for the work included in this submission. Review the submission guidelines for detailed requirements. View published research articles from *PLOS ONE* for specific examples.<br><br>This statement is required for submission and **will appear in the published article** if the submission is accepted. Please make sure it is accurate.	ADG received the award<br><br>Bill and Melinda Gates Foundation, grant number OPP1034523<br><br>https://www.gatesfoundation.org/<br><br>The funders did not play a role in the study design, data collection and analysis, decision to publish, or preparation of the manuscript.

Powered by Editorial Manager® and ProduXion Manager® from Aries Systems Corporation

**Funded studies**<br>Enter a statement with the following details:<br>• Initials of the authors who received each award<br>• Grant numbers awarded to each author<br>• The full name of each funder<br>• URL of each funder website<br>• Did the sponsors or funders play any role in the study design, data collection and analysis, decision to publish, or preparation of the manuscript?<br><br>Did you receive funding for this work?"	
Please select the country of your main research funder (please select carefully as in some cases this is used in fee calculation).<br>as follow-up to "**Financial Disclosure**<br><br>Enter a financial disclosure statement that describes the sources of funding for the work included in this submission. Review the submission guidelines for detailed requirements. View published research articles from *PLOS ONE* for specific examples.<br><br>This statement is required for submission and **will appear in the published article** if the submission is accepted. Please make sure it is accurate.<br><br>**Funded studies**<br>Enter a statement with the following details:<br>• Initials of the authors who received each award<br>• Grant numbers awarded to each author<br>• The full name of each funder<br>• URL of each funder website<br>• Did the sponsors or funders play any role in the study design, data collection and analysis, decision to publish, or preparation of the manuscript?<br><br>Did you receive funding for this work?"	UNITED STATES - US
**Competing Interests**	The authors have declared that no competing interests exist.

Powered by Editorial Manager® and ProduXion Manager® from Aries Systems Corporation

Use the instructions below to enter a competing interest statement for this submission. On behalf of all authors, disclose any competing interests that could be perceived to bias this work—acknowledging all financial support and any other relevant financial or non-financial competing interests.<br><br>This statement is required for submission and **will appear in the published article** if the submission is accepted. Please make sure it is accurate and that any funding sources listed in your Funding Information later in the submission form are also declared in your Financial Disclosure statement.<br><br>View published research articles from *PLOS ONE* for specific examples.<br><br>**NO authors have competing interests**<br><br>Enter: *The authors have declared that no competing interests exist.*<br><br>**Authors with competing interests**<br><br>Enter competing interest details beginning with this statement:<br><br>*I have read the journal's policy and the authors of this manuscript have the following competing interests: [insert competing interests here]*<br><br>* typeset	
**Ethics Statement**<br><br>Enter an ethics statement for this submission. This statement is required if the study involved:<br><br>• Human participants<br>• Human specimens or tissue<br>• Vertebrate animals or cephalopods<br>• Vertebrate embryos or tissues	Ethical considerations<br>The study protocol was approved by the University of the Witwatersrand Human Research Ethics Committee, Johannesburg (M 120343); the University of Cape Town, Cape Town Human Research Ethics Committee, South Africa (106/2012); and the London School of Hygiene & Tropical Medicine Research Ethics Committee UK (6165). All participants were informed about the study and gave written consent to participate and be recorded during the FGDs. Audio recordings, field notes, and transcripts were identified only by a unique study number. All study records were stored securely in locked filing cabinets where access to the records is restricted to specified study team members.

Powered by Editorial Manager® and ProduXion Manager® from Aries Systems Corporation

• Field research<br><br>Write "N/A" if the submission does not require an ethics statement.<br><br>General guidance is provided below. Consult the submission guidelines for detailed instructions. **Make sure that all information entered here is included in the Methods section of the manuscript.**	

Powered by Editorial Manager® and ProduXion Manager® from Aries Systems Corporation

**Format for specific study types**<br><br>**Human Subject Research (involving human participants and/or tissue)**<br>• Give the name of the institutional review board or ethics committee that approved the study<br>• Include the approval number and/or a statement indicating approval of this research<br>• Indicate the form of consent obtained (written/oral) or the reason that consent was not obtained (e.g. the data were analyzed anonymously)<br><br>**Animal Research (involving vertebrate animals, embryos or tissues)**<br>• Provide the name of the Institutional Animal Care and Use Committee (IACUC) or other relevant ethics board that reviewed the study protocol, and indicate whether they approved this research or granted a formal waiver of ethical approval<br>• Include an approval number if one was obtained<br>• If the study involved *non-human primates*, add *additional details* about animal welfare and steps taken to ameliorate suffering<br>• If anesthesia, euthanasia, or any kind of animal sacrifice is part of the study, include briefly which substances and/or methods were applied<br><br>**Field Research**<br><br>Include the following details if this study involves the collection of plant, animal, or other materials from a natural setting:<br>• Field permit number<br>• Name of the institution or relevant body that granted permission	
**Data Availability**<br><br>Authors are required to make all data underlying the findings described fully available, without restriction, and from the time of publication. PLOS allows rare exceptions to address legal and ethical concerns. See the PLOS Data Policy and FAQ for detailed information.	Yes - all data are fully available without restriction

A Data Availability Statement describing where the data can be found is required at submission. Your answers to this question constitute the Data Availability Statement and **will be published in the article**, if accepted.<br><br>**Important:** Stating 'data available on request from the author' is not sufficient. If your data are only available upon request, select 'No' for the first question and explain your exceptional situation in the text box.<br><br>Do the authors confirm that all data underlying the findings described in their manuscript are fully available without restriction?	
**Describe where the data may be found in full sentences. If you are copying our sample text, replace any instances of XXX with the appropriate details.**<br><br>• If the data are **held or will be held in a public repository**, include URLs, accession numbers or DOIs. If this information will only be available after acceptance, indicate this by ticking the box below. For example: *All XXX files are available from the XXX database (accession number(s) XXX, XXX.*).<br>• If the data are all contained **within the manuscript and/or Supporting Information files**, enter the following: *All relevant data are within the manuscript and its Supporting Information files.*<br>• If neither of these applies but you are able to provide **details of access elsewhere**, with or without limitations, please do so. For example:<br><br>*Data cannot be shared publicly because of [XXX]. Data are available from the XXX Institutional Data Access / Ethics Committee (contact via XXX) for researchers who meet the criteria for access to confidential data.*<br><br>*The data underlying the results presented in the study are available from (include the name of the third party*	The data analyzed for this manuscript has been provided.

and contact information or URL).<br>• This text is appropriate if the data are owned by a third party and authors do not have permission to share the data.<br><br>* typeset	
Additional data availability information:	

Manuscript
Click here to access/download;Manuscript;Manuscript_clean.docx

"*If we lose it, we are worried*": Individual and provider level perceptions and attitudes towards weight change among people living with HIV screening for tuberculosis in routine health care settings in Gauteng Province, South Africa.

Authors

Tanyaradzwa Nicolette Dube[1], Fezeka Mboniswa[1], Samkelisiwe Ethel Qwana[2] Salome Charalambous[1,4], Nontobeko Ndlovu[1] Alison D. Grant [3,5], Yasmeen Hanifa[3] Johannes Machinya[6]

[1]The Aurum Institute, Johannesburg, South Africa; [2]Research Consulting Specialists CC Johannesburg, South Africa [3]TB centre, London School of Hygiene & Tropical Medicine, London, United Kingdom, [4]School of Public Health, Faculty of Health Sciences, University of the Witwatersrand, Johannesburg, South Africa, [5]Africa Health Research Institute, School of Laboratory Medicine and Medical Sciences, College of Health Sciences, University of KwaZulu-Natal, Durban, South Africa. [6] Department of Sociology, University of the Witwatersrand, Johannesburg, South Africa

Corresponding Author: Tanyaradzwa Dube, The Aurum Institute, Aurum House, The Ridge, 29 Queens Road, Parktown, Johannesburg, 2193, South Africa, Telephone: +27 (0)10 590 1600, Fax: . Email: tndube@auruminstitute.org

Abstract

Background

HIV weakens the immune system, increasing the risk of tuberculosis (TB) in people with HIV (PLHIV). People living with HIV and on antiretroviral treatment (ART) often experience physical body size changes. Studies have found a significant discrepancy between PLHIV's self-reported weight loss and their measured weight loss when being screened for TB using the WHO tool. To understand this inconsistency, a qualitative sub-study was conducted to explore perceptions and attitudes towards weight change among adults attending HIV care, as well as health care workers in public clinics in Gauteng, South Africa.

Methods

Our qualitative study was nested within the XPHACTOR study. A total of seven focus group discussions were conducted, five with adult participants attending for HIV care and two with health care workers and research staff in clinics around Gauteng. Inductive thematic analysis was used to analyse the data.

Findings

The majority of PLHIV preferred to gain weight due to fear of stigma associated with weight loss. Weight loss is associated with HIV/AIDS, suggesting that people attending HIV care may underreport weight loss in the context of a TB symptoms screening tool because they fear stigma. Participants reported that weight changes impacted their daily lives and had psychological effects on them. Some PLHIV described lipodystrophy as disproportional weight gain. Culture and media have an influence on the perception of ideal body size and shape for both men and women.

Conclusions

Underreporting weight loss might result in poor sensitivity of the WHO TB screening tool and suggests that we need either alternative ways to determine weight loss or screening tools for TB that are less dependent on reported symptoms.

Keywords: HIV, TB screening**,** Weight change, ART, South Africa, WHO screening tool

Abstract word count 270/300

Manuscript word count 4655

Background

Tuberculosis (TB) is one of the most common opportunistic infections in people living with the human immunodeficiency virus (PLHIV) and a major contributor to morbidity and mortality[1,2]. The risk of developing active TB disease is estimated to be 16 (uncertainty interval 14–18) times greater in PLHIV than those without HIV infection[3]. Weight loss is a common symptom of TB, and the World Health Organization (WHO) recommends screening people living with HIV for TB using a tool that comprises questions on cough, fever, night sweats, and unintentional weight loss[4]. Unintentional weight loss is a cardinal symptom of TB and reporting of weight loss is a key entry point to testing for TB, so understanding perceptions and reporting of weight loss is important with regards to finding people with active TB.

People living with HIV and on antiretroviral treatment (ART) often experience physical body size changes such as weight change, and other body shape changes that can lead to stigmatization[5–10]. In South Africa, changes in body shape were particularly common among PLHIV taking stavudine, which was routinely used in ART between 2004 and 2010 but is now rarely used because of its side effect profile[11].

Two studies conducted in South Africa found a mismatch between PLHIV's self-reported and measured weight change: one was a quantitative study and the other conducted in-depth interviews focusing on adolescents only[12,13]. It is important to understand perceptions of PLHIV regarding weight and body shape changes because these may impact on responses to the weight loss component of the WHO TB symptom screening tool, as well as influence adherence to ART and TB treatment. A better understanding of weight perceptions may help improve TB screening and guide

policies and recommendations for supporting PLHIV when they experience weight and body shape changes.

We conducted an in-depth qualitative study to understand weight perceptions among adults living with HIV as part of the XPHACTOR study. XPHACTOR was a cohort study investigating TB diagnostic pathways among adults attending routine HIV care in South Africa between 2012 and 2014 when standard ART regimens in South Africa included stavudine[14–16]. In XPHACTOR, PLHIV were screened for TB using the WHO screening tool and significant discrepancies were noted between participants' self-reported weight loss and their measured weight loss. To understand this inconsistency, a qualitative sub-study was conducted to explore perceptions and attitudes towards weight change among adults attending HIV care, as well as among health care workers (HCWs) in public clinics in Gauteng, South Africa. We were interested to know whether attitudes to weight and body size might affect how people responded to the questions in the WHO TB symptom screening tool and whether people with stavudine-related lipodystrophy might describe the change in body shape as weight loss.

Methodology

Study design

This was an exploratory qualitative sub-study.

Study setting and population.

Focus group discussions (FGDs) were conducted at public clinics providing care for PLHIV in Gauteng. Participants were adults attending the clinics for routine HIV care at XPHACTOR study sites, HCWs, and XPHACTOR research staff.

Data collection

Seven FGDs with a semi-structured guide were conducted: five FGDs with purposively sampled adults attending for HIV care and two FGDs with HCWs and research staff. The FGDs with PLHIV included two groups with male-only participants, two groups with female-only participants, and one mixed group. For HCWs, there were two FGDs, one FGD was done with doctors and dieticians and the other with other HCWs and research staff. All the FGDs were conducted at the clinics where the study was being conducted and where PLHIV and health workers were attending or working. Adult PLHIV who were not XPHACTOR study participants were approached for participation in the study face to face via an ART support group linked to the clinic. Research Assistants and HCWs were contacted via email. The FGDs were conducted face-to-face by an experienced independent female research consultant (SEQ) with a Masters' degree. All participants completed the FGD. To ensure that important points were discussed, the moderator led the discussions using FGD guides for PLHIV (S1 annexure 1) and HCWs and research staff (S2 annexure 2). There was no pre-existing relationship between the moderator and participants. FGDs were conducted within the clinic setting where participants collect their medication.

The length of the FGDs ranged between 83 minutes and 202 minutes. All sessions were audio recorded. The moderator took some field notes. No one else was present in the FGDs besides the participants and researchers. We initially planned to conduct around three FGDs but eventually conducted seven FGDs to reach saturation. There were no repeat interviews done. All participants in the FGDs except those who are

members of the research team were given ZAR 100=USD 15 to compensate for their time.

In addition to using a moderator guide, the interviewer probed on the questions and was able to gather more detailed responses from the participants. Participants were asked questions about their perceptions on ideal body weight, what informed these perceptions, personal experiences of, and attitudes towards body weight changes. In addition, they were asked questions about causes of weight change and reasons why weight changes measured at the clinic may differ from those reported by the patient. The HCWs and research staff were also asked questions about recent experiences of consultations with PLHIV around body weight. Images of body figures of different body shapes developed by Stunkard were used to explore perceptions about body size and body image. The figures ranged from one to nine where one is the thinnest body type and nine is the largest type[17]. There were separate figures for females and males. Participants were shown the images and were asked which body image represented them, which body image they preferred, and the most attractive body image.

Fig 1 The Stunkard scale of female body images shown to participants[17]

Fig 2 The Stunkard scale of male body images shown to participants[17].

Discussions were conducted in local languages IsiZulu, Sesotho, Setswana, and English. All discussions were tape-recorded to ensure that all the points were

captured. All interviews were transcribed verbatim, and interviews done in local languages were translated to English.

Ethical considerations

The study protocol was approved by the University of the Witwatersrand Human Research Ethics Committee, Johannesburg (M 120343); the University of Cape Town, Cape Town Human Research Ethics Committee, South Africa (106/2012); and the London School of Hygiene & Tropical Medicine Research Ethics Committee UK (6165). All participants were informed about the study and gave written consent to participate and be recorded during the FGDs. Audio recordings, field notes, and transcripts were identified only by a unique study number. All study records were stored securely in locked filing cabinets where access to the records was restricted to specified study team members.

Data analysis

A codebook was developed by two researchers (TD and FM) using inductive coding. Data were analysed manually using Excel. The two researchers independently inductively coded the transcripts and agreed on the emerging themes. Two data coders manually coded the data (TD and FM). TD has a Masters' degree and FM has an Honours degree. The senior author is an experienced qualitative researcher with a doctoral degree and supervised the data analysis (JM). The data coders manually coded a sample of the transcripts and the senior author (JM) qualitatively and quantitatively checked intercoder reliability between the coders. Inductive thematic analysis was employed to develop themes. Both males and females were included to account for variation in perceptions by gender. We used an Excel spreadsheet to monitor the number of steps that were followed during the manual coding process.

Transcripts and findings were not returned to participants for comment. The COREQ (Consolidated criteria for REporting Qualitative research) checklist was used to check the content of this manuscript.

Results

Seven FGDs (labelled 1-7) were held between 6th and 21st August 2014, with 6-12 participants in each focus group. The main identified themes were: i) negative attitudes towards weight loss; ii) stigma and loss of social support associated with weight loss; iii) psychological impact of weight loss; iv) changes in dressing due to weight changes.

Main theme 1: Perceptions and attitudes towards weight change

Sub-theme (i): Perceptions towards weight change

Participants in all the FGDs for adult PLHIV attending for HIV care expressed high level of awareness of the possible physical body changes experienced by PLHIV and on ART such as weight loss, weight gain and associated changes to body shape. The participants were aware of the main causes of weight loss, and they linked weight loss to poor diet, stress, active TB, HIV/TB treatment side effects, and non-adherence to HIV/TB treatment. It was reported that denial or failure to accept and embrace one's HIV positive status also resulted in weight loss.

> *I am saying, another thing that can make a person to lose weight is that because I am HIV positive, I must check whether I have TB or not because that also causes a person to lose weight (FGD 3, Females).*

Participants in the separate groups were asked how they knew that they were losing weight. Both male and female participants reported that they noticed that they had lost weight if their clothes were not fitting well.

HCWs and research staff indicated that weight loss could be caused by other health conditions among PLHIV. As such, when they noted weight loss in a PLHIV attending for HIV care, they reported that they would usually or ideally check the PLHIV's viral load, CD4 count, investigate for TB and other opportunistic infections, screen for cervical cancer, and test for diabetes.

Both PLHIV and HCWs identified ART side effects as one of the causes of weight gain and change in body shape among PLHIV:

> *Let me say, you can see that I am slender but if I take these tablets, you see and acquire a big stomach, they will then say he is in this condition, he is now taking these tablets, isn't these tablets are making him to have a big stomach* (FGD 1, Males).

Participants from the PLHIV's FGDs were happy to gain weight. They highlighted that weight gain was a sign that they were adhering to HIV treatment and that they were responding well to treatment:

> *… if we gain weight, I believe that we are happy, because it means that no, we are happy, we are eating well, we are doing things the right way and that our medication is working well but if we lose it, we are worried because it's like we are not taking these tablets* (FGD 5, Mixed).

PLHIV also perceived weight gain as a sign that one has accepted and embraced their HIV positive status:

> *It means that you are relaxed you are not obsessing over your HIV status, you see, you do not put yourself under stress, you think that I do have tablets. If I take tablets everything is okay* (FGD 1, Males).

Some PLHIV described lipodystrophy as disproportional weight gain. While PLHIV indicated that they would prefer weight gain to weight loss, they highlighted that sometimes when one gains weight, it might not be proportional to one's body size because they will gain weight on the upper body and lose weight on the lower part of the body, and this worried them:

> *You feel good when you gain, but what you do not like is that sometimes you can gain on the upper part and lose weight on the lower part. So that is what makes us sad* (FGD 4 Females).

Sub-theme 2: Attitudes towards weight loss

There were both positive and negative attitudes towards weight loss. Some participants revealed that excessive weight gain was not healthy, so they adopted healthy lifestyle including regular exercise and celebrated weight loss after gaining excessive weight, as shown in the quote below:

> *I was happy today because I had lost a lot of weight (FGD 3, Females).*

Majority of the participants demonstrated negative attitudes toward weight loss and some associated weight loss with the side effects of ART. Such participants reported that they would consider stopping ART to gain weight:

> *For me sometimes, I get a feeling that maybe these ARVs do not get along with my system and then have another feeling that hey I will stop taking them since I do not see its results, I keep losing weight, that is why most of the time you find that after people have started ARVs.., you find that a person does not take them anymore and they will say, if I don't take them I regain my weight,* (FGD 4, Females).

Participants further reported that there were some nurses who reproached them for delaying seeking health care when they visited the clinic with sickness and showing signs of weight loss:

> *You see at the clinic, the nurses scold at you if you visited them when you were really too sick and badly affected, [they] can see that you have lost weight, the nurse scolds at you and say, you are this and that why did you not come and visit me (says P mimicking a female nurse) they scold at you there.* (FGD, 1 Males).

The HCWs reported that they would become inquisitive about adult PLHIVs lifestyles if they showed signs of gaining weight as highlighted by a dietician in the HCWs group:

> *If they are gaining weight, we want to know if they are exercising or not* (FGD 7, Dieticians and doctors).

In addition to weighing PLHIV on a scale, HCWs stated that they used alternative ways to identify if PLHIV had lost weight without openly using the words "weight loss" to get accurate answers and avoid being seen as stigmatizing PLHIV. The HCWs reported that they would instead ask participants if the size of their dress has changed or if family members had commented on changes in their body shape:

> '*Has your dress size changed?' could be the alternative question* (FGD 7, Dieticians and doctors).

> *And then you can ask sometimes, if anyone, your friend, or family complained that your body shape has changed* (FGD 7, Dieticians and doctors).

Main theme 2 : Ideal body size and shape

The participants were aware that weight change also alters both body size and shape. To gain insights into the participants' thoughts on their perceptions of ideal body size

and shape, they were shown both male and female Stunkard body image figures(17). The FGDs were shown both male and female images. The majority of male participants selected body image figure number five in the middle as the ideal figure for men Some men suggested that women should be the same size as men and others reported that women should be slightly bigger because they bear children. Female participants selected body image figure number six as the ideal body type because the image had a mid-sized proportional body. The choice of ideal body size was influenced by media for both women and men as indicated in the responses below:

> *I think influence in terms of being muscular has a lot to do with media. TV gives you the suggestion that someone who is muscular is more acceptable to the community (FGD 2, Males).*

> *they make jokes out of us, as women, they say check her out she has unnecessary fats on the waist area, you know it would have been better if I found someone like Beyoncé [female celebrity], you see. (FGD 4, Females).*

Participants further reported that culture also influenced how people perceive ideal body shape especially for women.

> *My sister is right about culture, Zulus prefer fat women. They say she is well-fed (FGD 3, Females).*

> *And I think we have a black culture that a black woman has to be round (Participants agreeing with one another), they will just be worried, when number 4, they won't even look at you (FGD 6, HCW and Research Staff).*

HCWs revealed they would ask PLHIV who had signs of lipodystrophy whether other people had commented on the changes in their body shape or size. According to the HCWs, PLHIV were more worried about changes in body shape or size and not so

much about weight loss. It was uncommon for PLHIV to visit the clinic because they were worried about weight loss, but they usually visited the clinic when they noticed changes in body shape:

> *What I have noticed is that it is mostly PLHIV who have got lipodystrophy, they are the ones who are more worried about their weight that's one thing I have realised because they are saying, no, I no longer have my buttocks, they are gone, my legs are now thinner. (FGD 7, Dieticians and doctors).*

HCWs stated that when they noticed changes in body shape and size among the PLHIV, they changed the regimen for those who were taking stavudine. PLHIV on the second-line ART regimen (at the time of the study, 2014, azidothymidine, lamivudine, lopinavir/ritonavir), were counselled and some were referred to government-funded plastic surgery services. HCWs also encouraged PLHIV to exercise and improve their diet.

FGDs with HCWs and research staff revealed that PLHIV who lost weight were referred to dieticians, social workers, psychologists, and religious leaders for support. PLHIV with socio-economic challenges were referred to the South African Social Security Agency for the Disability Grant. HCWs also revealed that they involved the families of PLHIV experiencing weight loss to explore potential causes.

Main theme 3: Negative impact of weight loss on participant's daily life

Sub-theme 1: Stigma and loss of social support

Participants reported that changes in physical body size, particularly weight loss, resulted in them being stigmatized in the community. Some of the participants reported that they experienced body shaming, name-calling, gossip, and discrimination because they had lost too much weight. Participants in the FGDs lamented that people

in the communities would quickly conclude that a person has AIDS once one begins to lose weight. The participants reported that those who show signs of weight loss are said to have bought OMO (washing powder) because the HIV "washed" way the person's body the way OMO washes dirt:

> *They will say, so and so bought OMO can you actually see so and so, so in that manner they call you by those names when you are on the passing by, and you would think my goodness. They do this so that you cannot understand you see, you may not know why you are regarded as having bought OMO, when you look around no one bought OMO, they regard you as that since you lost weight* (FGD 1, Males).

Participants with lipodystrophy were stigmatized and were called names such as "high heels" and KFC because of having thin legs and a fat upper body:

> *Thin legs. Do you know what a KFC drumstick looks like? When they see you, they say: It's KFC! She went to buy KFC* (FGD 3, Females).

Participants also reported stigmatizing attitudes from both community and family members which include being ignored and people keeping some distance from them:

> *Mostly, if they know that you are positive through appearance, the loss of weight, they start ignoring you and mostly the names like killer* (FGD, 2 Males).

> *Even your family still does that thing, they say hey, don't stand too close to me you will infect me with this thing* (FGD 4 Females).

Some participants in the FGDs reported that PLHIV who show signs of weight changes may lose social support from their families and they attributed this to a lack of adequate knowledge on HIV:

So, this is what happened …, my mother is an elderly person so she does not know the difference between HIV and AIDS. When my brother took a cup and drank from it, she would want nothing to do with that cup. So, I called her and tried to teach her about what HIV and AIDS are. I said I'm asking you to please support him (FGD 3, Females).

Sub-theme 2: Psychological impact of weight loss

The participants in focus group discussions revealed that when some PLHIV discover that they have lost weight, they become so stressed:

The thing is, you just become stressed out, you just cannot concentrate on the things that you are doing at home, do you understand, you become short-tempered, you ask yourself what is going on, what did I do, what mistake did I make in order for me to lose weight in this manner (FGD, 4 Female).

Some PLHIV revealed they feel anxious and stressed when they are asked to stand on the scale and be weighed at the clinic:

Before standing on the scale, I was afraid that they are about to weigh me now and it's possible that I may have lost weight (FGD1, Males).

Sub-theme 3: Changes in dressing

When they notice that they have lost weight, participants reported that they would change the way they dress to cover signs of weight loss:

So, now that I have lost weight, when I get dressed and wear something that hides my bums they say why are you making yourself a granny? You must wear the latest fashion, and I say: hey I am not a child (FGD, 3 Female).

Discussion

Participants demonstrated a strong awareness that some physical body changes such as weight gain and weight loss may be symptoms of HIV and AIDS. The participants further indicated that negative weight changes, i.e., weight loss, attract stigmatizing attitude from society and that stigma remains a huge concern for PLHIV. Because of this, the participants preferred gaining to losing weight. These findings are consistent with findings from other studies in South Africa that found that PLHIV would rather gain weight than be thin and have people assume that they are infected with HIV[5,12]. Corroborating our finding, a study in Uganda found that women living with HIV did not want to lose weight because weight loss could lead to people suspecting their HIV seropositive status. Women living with HIV who lost weight in the same study also reported that they felt stigmatized, and it affected their adherence to ART[18]. The finding that it was rare for PLHIV to seek healthcare services because of weight loss concerns may negatively affect efforts to screen for and diagnose TB early.

In our study, HCWs reported that because of the stigma attached to weight loss, they avoid asking PLHIV direct questions about weight loss because the fear of the stigma associated with weight loss may hinder PLHIV from telling the truth. The stigma associated with weight loss may make the WHO screening tool less sensitive, specifically the question on weight loss. A potential response could be to weigh people seeking health care services instead of asking them the question on weight loss. The stigma associated with HIV and TB needs to be addressed for example by educating, through community outreach campaigns, both PLHIV, family members, and the community on HIV and TB using tool kits that have been developed to address TB-related stigma[19–21].

Despite the preference to gain rather than lose weight amongst the majority of participants, some participants were aware that excessive weight gain has repercussions and reported adopting a healthy lifestyle including regular exercise and celebrated weight loss. A study conducted in Cape Town, South Africa found that participants preferred to be overweight and at risk of acquiring cardiovascular diseases, rather than to lose weight and be stigmatised as infected with HIV[5]. It is important to educate PLHIV that excessive weight gain may lead to obesity and increase the risk of non-communicable diseases. Therefore, PLHIV should be encouraged to exercise and eat well to reduce the risk of these diseases[8,13,22].

The psychological impact of weight changes leading to non-adherence found in our study has been reported in other studies[8,13,18,22]. Lipodystrophy is a concern among PLHIV. The finding that changes in body image affect PLHIV psychologically, with some considering stopping treatment, is consistent with other studies. A study on lipodystrophy among PLHIV in Tanzania found that changes in body shape may affect PLHIV psychosocial function, quality of life and may lead to PLHIV considering stopping treatment[22].

Our study found that fuller women were considered to have the ideal body shape. This finding is consistent with findings from other previous studies. A study conducted among adolescents in Durban revealed that female respondents desired to be thick and curvaceous, and males desired to be bigger and muscular, and this was linked to the influence of cultural norms on understanding of an ideal body shape[13]. Similarly, a study conducted in Cape Town found that cultural beliefs appeared to have a significant influence on the women interviewed, despite more than half of the participants having a high school education. Some women in this study reported that women are culturally expected to have a fuller figure[5].

Stavudine is now rarely used and so few PLHIV should newly develop lipodystrophy. Those with established lipodystrophy should have been switched to a different regimen which does not cause lipodystrophy, but these people may need support and reassurance to continue their current ART[11].

Participants in our study reported that changes in dress sizes made them realize they lost weight. HCWs in our study recommended that asking participants changes in clothing size could be better than directly asking unintentional weight loss questions. Asking clothing size is a self-report measure which can get biased responses, but we feel is more acceptable to patients rather than directly asking about weight loss. A study in Netherlands found that self-reported clothing size appeared to predict cancer risk independently of body mass index, suggesting that self-reported clothing size can be a useful measure to consider in epidemiologic studies[23].

Our study findings are significant in today's terms as TB remains a leading cause of death among PLHIV so understanding their perceptions around weight change and how this may affect TB symptom screening is important. In addition, since this work, the new first-line treatment regimen for PLHIV includes dolutegravir, which has been found to have weight gain as a side effect. Hence, perceptions on weight gain highlighted in our paper remain relevant [18,23].

Strengths and limitations

One of the strengths of this study is that we conducted FGDs with both males and females and managed to get perceptions on weight change from both genders. However, the study was not without limitations. The sample size of the PLHIV who were interviewed was small and may not be representative of all PLHIV at the facilities. Again, PLHIV may not have been able to disclose sensitive personal information in

focus groups. Despite these limitations, we managed to gain insights from PLHIV as well as HCWs on people's perceptions of weight change among people who live with HIV. The potential bias was minimised by the fact that the facilitator was an independent researcher, therefore would have been perceived to have adopted a neutral stance on the issues.

Conclusion

The majority of PLHIV preferred to gain weight due to fear of stigma associated with weight loss. Weight loss is associated with HIV /AIDS, suggesting that people attending for HIV care may underreport weight loss in the context of a TB symptom screening tool. This would result in reduced sensitivity of the WHO TB screening tool. Findings suggest that we need either alternative ways to determine weight loss, or screening tools for TB which are less dependent on reported symptoms. Furthermore, the policy implications of our paper suggest the importance of strengthening innovative TB testing strategies such as Targeted Universal Test and Treat (TUTT) which tests high risk groups including PLHIV irrespective of reporting TB symptoms or ART status [24].

Acknowledgments

The authors are grateful to the Department of Health nurses and staff at the facilities, the XPHACTOR research staff, and all the participants.

Author contributions

ADG, SC, and YH conceptualized the study. SEQ conducted the FGDs. TD and FM performed the data analysis with guidance from JM. NN was the project manager. TD drafted the paper and all authors provided critical reviews, edited drafts, and approved the version to be published.

Reference List

1. Adamu AL, Gadanya MA, Abubakar IS, Jibo AM, Bello MM, Gajida AU, et al. High mortality among tuberculosis patients on treatment in Nigeria: A retrospective cohort study. BMC Infect Dis. 2017;17(1):1–12.
2. Tornheim JA, Dooley KE. Tuberculosis Associated with HIV Infection. American Society for Microbiology. 2017;5(1):1–16.
3. World Health Organization. Tuberculosis Key Facts [Internet]. 2023 [cited 2023 Jul 5]. Available from: https://www.who.int/news-room/fact-sheets/detail/tuberculosis
4. WHO. WHO Consolidated Guidelines on Tuberculosis. Module 2 Screening [Internet]. Who. 2021 [cited 2024 Sep 10]. Available from: https://iris.who.int/bitstream/handle/10665/340255/9789240022676-eng.pdf?sequence=1
5. Matoti-Mvalo T, Puoane T. Perceptions of body size and its association with HIV/AIDS. South African Journal of Clinical Nutrition. 2011;24(1):40–5.
6. Li N, Spiegelman D, Drain P, Mwiru RS, Mugusi F, Chalamilla G, et al. Predictors of weight loss after HAART initiation among HIV-infected adults in Tanzania. Aids. 2011;26(5):577–85.
7. Tate T, Willig AL, Willig JH, Raper JL, Moneyham L, Kempf MC, et al. HIV infection and obesity: Where did all the wasting go? Antivir Ther. 2012;17(7):1281–9.
8. Power R, Tate HL, McGill SM, Taylor C. A qualitative study of the psychosocial implications of lipodystrophy syndrome on HIV positive individuals. Sex Transm Infect. 2003;79(2):137–41.
9. Lakey W, Yang LY, Yancy W, Chow SC, Hicks C. Short Communication: From wasting to obesity: Initial antiretroviral therapy and weight gain in HIV-infected persons. AIDS Res Hum Retroviruses. 2013;29(3):435–40.
10. Sax PE, Erlandson KM, Lake JE, McComsey GA, Orkin C, Esser S, et al. Weight gain following initiation of antiretroviral therapy: Risk factors in randomized comparative clinical trials. Clinical Infectious Diseases. 2020;71(6):1379–89.
11. The South African Antiretroviral Treatment Guidelines 2010 [Internet]. 2010 [cited 2024 Apr 19]. Available from: https://sahivsoc.org/Files/Summary_The_South_African_Antiretroviral_Treatment_2010.pdf
12. Hurley E, Coutsoudis A, Giddy J, Knight SE, Loots E, Esterhuizen TM. Weight evolution and perceptions of adults living with HIV following initiation of antiretroviral therapy in a South African urban setting. South African Medical Journal. 2011;101(9):645–50.

13. Nyamaruze P, Govender K. € I like the way i am, but i feel like i could get a little bit bigger": Perceptions of body image among adolescents and youth living with HIV in Durban, South Africa. PLoS One [Internet]. 2020;15(1):1–10. Available from: http://dx.doi.org/10.1371/journal.pone.0227583

14. Hanifa Y, Fielding KL, Chihota VN, Adonis L, Charalambous S, Foster N, et al. A clinical scoring system to prioritise investigation for tuberculosis among adults attending HIV clinics in South Africa. PLoS One. 2017 Aug 1;12(8).

15. Pillai N, Foster N, Hanifa Y, Ndlovu N, Fielding K, Churchyard G, et al. Patient costs incurred by people living with HIV/AIDS prior to ART initiation in primary healthcare facilities in Gauteng, South Africa. PLoS One. 2019 Feb 1;14(2).

16. Auld AF, Kerkhoff AD, Hanifa Y, Wood R, Charalambous S, Liu Y, et al. Derivation and external validation of a risk score for predicting HIV-associated tuberculosis to support case finding and preventive therapy scale-up: A cohort study. PLoS Med. 2021 Sep 1;18(9).

17. Sorensen TIA, Stunkard AJ. Does obesity run in families because of genes? An adoption study using silhouettes as a measure of obesity. Acta Psychiatr Scand. 1993;67–72.

18. Alhassan Y, Twimukye A, Malaba T, Myer L, Waitt C, Lamorde M, et al. "It's only fatness, it doesn't kill": a qualitative study on perceptions of weight gain from use of dolutegravir-based regimens in women living with HIV in Uganda. BMC Womens Health. 2022 Dec 1;22(1).

19. Kidd Ross, International HIV/AIDS Alliance., ZAMBART Urban Tuberculosis Project. Understanding and challenging TB stigma toolkit for action : introduction to TB stigma, more understanding and less fear about TB [Internet]. International HIV/AIDS Alliance; 2009 [cited 2024 Sep 10]. 68 p. Available from: https://assets.publishing.service.gov.uk/media/57a08b3ced915d622c000bb7/TB_and_Stigma_May09.pdf

20. AIDS Foundation of South Africa. Reducing Stigma related to Gender, Culture and HIV/AIDS [Internet]. 2017 [cited 2024 Sep 10]. Available from: https://www.aids.org.za/wp-content/uploads/2018/05/Facilitator%E2%80%99s-Guide-Stigma-Related-to-Gender-Culture-HIV.pdf

21. Nyblade L, Stangl A, Weiss E, Ashburn K. Combating HIV stigma in health care settings: What works? Vol. 12, Journal of the International AIDS Society. John Wiley and Sons Inc.; 2009.

22. Njelekela M, Mpembeni R, Muhihi A, Ulenga N, Aris E, Kakoko D. Lipodystrophy among HIV-Infected Patients Attending Care and Treatment Clinics in Dar es Salaam. 2017;2017(October 2004).

23. Brennan AT, Nattey C, Kileel EM, Rosen S, Maskew M, Stokes AC, et al. Change in body weight and risk of hypertension after switching from efavirenz

to dolutegravir in adults living with HIV: evidence from routine care in Johannesburg, South Africa. EClinicalMedicine. 2023 Mar 1;57.

24. Martinson NA, Nonyane BAS, Genade LP, Berhanu RH, Naidoo P, Brey Z, et al. Evaluating systematic targeted universal testing for tuberculosis in primary care clinics of South Africa: A cluster-randomized trial (The TUTT Trial). PLoS Med. 2023 May 1;20(5 May).

Figure

Click here to access/download;Figure;Fig 1 The Stunkard scale of female body images.tif

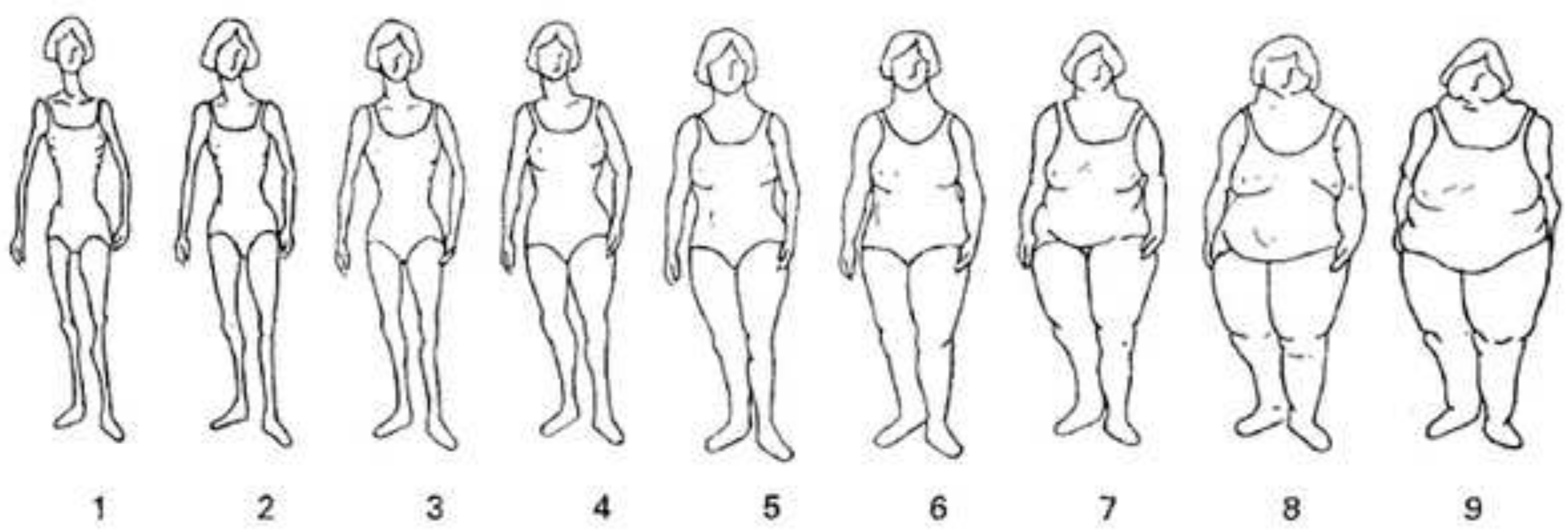

Figure

Click here to access/download;Figure;Fig 2 Figure 2 The Stunkard scale of male body images.tif

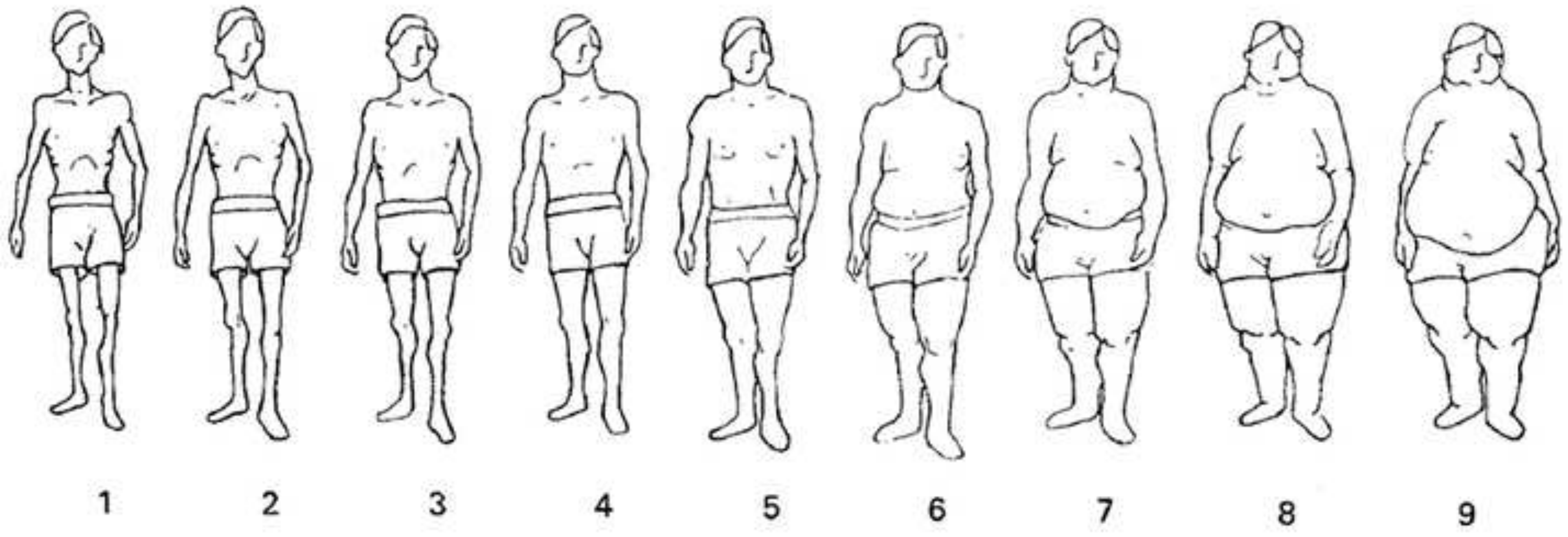

Click here to access/download
Supporting Information
S3 CoreQ _Checklist_November.pdf

Click here to access/download
Supporting Information
S5 Ethics Approval MHREC Approval version 5.1.pdf

Click here to access/download
Supporting Information
S6 Ethics
Aproval_Grant_A487_6165_approval_06012014.pdf

Click here to access/download

Supporting Information - Compressed/ZIP File Archive

S4 Transcripts (Data).zip

"*If we lose it, we are worried*": Individual and provider level perceptions and attitudes towards weight change among people living with ~~PL~~HIV screening for tuberculosis~~TB~~ in routine health care settings in Gauteng Province, South Africa.

~~Perceptions and attitudes towards weight change among adults attending HIV care and health care workers in public clinics in South Africa.~~

Authors

Tanyaradzwa Nicolette Dube[1], Fezeka Mboniswa[1] , Samkelisiwe Ethel Qwana[2] Salome Charalambous[1,4], Nontobeko Ndlovu[1] Alison D. Grant [3,5], Yasmeen Hanifa[3] Johannes Machinya[6]

[1]The Aurum Institute, Johannesburg, South Africa; [2]Research Consulting Specialists CC Johannesburg, South Africa [3]TB cent~~e~~re, London School of Hygiene & Tropical Medicine, London, United Kingdom, [4]School of Public Health, Faculty of Health Sciences, University of the Witwatersrand, Johannesburg, South Africa, [5]Africa Health Research Institute, School of Laboratory Medicine and Medical Sciences, College of Health Sciences, University of KwaZulu-Natal, Durban, South Africa. [6] Department of Sociology, University of the Witwatersrand, Johannesburg, South Africa

Corresponding Author: Tanyaradzwa Dube, The Aurum Institute, Aurum House, The Ridge, 29 Queens Road, Parktown, Johannesburg, 2193, South Africa, Telephone: +27 (0)10 590 1600, Fax: . Email: tndube@auruminstitute.org

Abstract

Background

HIV weakens the immune system, increasing the risk of tTuberculosis (TB) in people with HIV (PLHIV). People living with HIV and on antiretroviral treatment (ART) often experience physical body size changes. Studies have found a significant discrepancy between PLHIV's self-reported weight loss and their measured weight loss when being screened for TB using the WHO tool. To understand this inconsistency, a qualitative sub-study was conducted to explore perceptions and attitudes towards weight change among adults attending HIV care, as well as health care workers in public clinics in Gauteng, South Africa.

Methods

Our qualitative study was nested within the XPHACTOR study. A total of seven focus group discussions were conducted, five with adult participants attending for HIV care and two with health care workers and research staff in clinics around Gauteng. Inductive thematic analysis was used to analyse the data.

Findings

The majority of PLHIV preferred to gain weight due to fear of stigma associated with weight loss. Weight loss is associated with HIV/AIDS, suggesting that people attending HIV care may underreport weight loss in the context of a TB symptoms screening tool because they fear stigma. Participants reported that weight changes impacted their daily lives and had psychological effects on them. Some PLHIV described lipodystrophy as disproportional weight gain. Culture and media have an influence on the perception of ideal body size and shape for both men and women.

Conclusions

~~Underreporting~~ Misreporting weight loss might result in poor sensitivity of the WHO TB screening tool and suggests that we need either alternative ways to determine weight loss or screening tools for TB that are less dependent on reported symptoms.

Keywords: HIV, TB screening**,** Weight change, ART, South Africa, WHO screening tool

Abstract word count 251/300

Manuscript word count 4382

Background

Tuberculosis (TB) is one of the most common opportunistic infections in people living with the human immunodeficiency virus (PLHIV) and a major contributor to morbidity and mortality[(1,2]). The risk of developing active TB disease is estimated to be 16 (uncertainty interval 14–18) times greater in PLHIV than those without HIV infection[(3]). Weight loss is a common symptom of TB, and the World Health Organization (WHO) recommends screening people living with HIV for TB using a tool that comprises questions on cough, fever, night sweats, and unintentional weight loss(4). Unintentional weight loss is a cardinal symptom of TB and reporting of weight loss is a key entry point to testing for TB, so understanding perceptions and reporting of weight loss is important with regards to finding people with active TB.

People living with HIV and on antiretroviral treatment (ART) often experience physical body size changes such as weight change, and other body shape changes that can lead to stigmatization[(5–10]). In South Africa, changes in body shape were particularly common among PLHIV taking stavudine, which was routinely used in ART between 2004 and 2010 but is now rarely used because of its side effect profile[(11]).

Two studies conducted in South Africa found a mismatch between PLHIV’s self-reported and measured weight change: one was a quantitative study and the other conducted in-depth interviews focusing on adolescents only[(12,13]). It is important to understand perceptions of PLHIV regarding weight and body shape changes because these may impact on responses to the weight loss component of the WHO TB symptom screening tool, as well as influence adherence to ART and TB treatment. A better understanding of weight perceptions may help improve TB screening and guide

policies and recommendations for supporting PLHIV when they experience weight and body shape changes.

We conducted an in-depth qualitative study to understand weight perceptions among adults living with HIV as part of the XPHACTOR study. XPHACTOR was a cohort study investigating TB diagnostic pathways among adults attending routine HIV care in South Africa between 2012 and 2014 when standard ART regimens in South Africa included stavudine[(14–16]). In XPHACTOR, PLHIV were screened for TB using the WHO screening tool and significant discrepancies were noted between participants' self-reported weight loss and their measured weight loss. To understand this inconsistency, a qualitative sub-study was conducted to explore perceptions and attitudes towards weight change among adults attending HIV care, as well as among health care workers (HCWs) in public clinics in Gauteng, South Africa. We were interested to know whether attitudes to weight and body size might affect how people responded to the questions in the WHO TB symptom screening tool and whether people with stavudine-related lipodystrophy might describe the change in body shape as weight loss.

Methodology

Study design

This was an exploratory qualitative sub-study.

Study setting and population.

Focus group discussions (FGDs) were conducted at public clinics providing care for PLHIV in Gauteng. Participants were adults attending the clinics for routine HIV care at XPHACTOR study sites, HCWs, and XPHACTOR research staff.

Data collection

Seven FGDs with a semi-structured guide were conducted: five FGDs with purposively sampled adults attending for HIV care and two FGDs with HCWs and research staff. The FGDs with PLHIV included two groups with male-only participants, two groups with female-only participants, and one mixed group. For HCWs, there were two FGDs, one FGD was done with doctors and dieticians and the other with other HCWs and research staff. All the FGDs were conducted at the clinics where the study was being conducted and where PLHIV and health workers were attending or working. Adult PLHIV who were not XPHACTOR study participants were approached for participation in the study face to face via an ART support group linked to the clinic. Research Assistants and HCWs were ~~conducted~~ -contacted via email. The FGDs were conducted face-to-face by an experienced independent female research consultant (SEQ) with a Masters' degree. ~~None of the participants dropped out of participation.~~ All participants completed the FGD. To ensure that important points were discussed, the moderator led the discussions using FGD guides for PLHIV (annexure 1) and HCWs and research staff (annexure 2). There was no pre-existing relationship between the moderator and participants. FGDs were conducted within the clinic setting where participants collect their medication.

Formatted: Font: (Default) Arial, 12 pt

The length of the FGDs ranged between 83 minutes and 202 minutes. All sessions were audio recorded. The moderator took some field notes. No one else was present in the FGDs besides the participants and researchers. We initially planned to conduct around three FGDs but eventually conducted seven FGDs to reach saturation. There were no repeat interviews done. All participants in the FGDs except those who are

members of the research team were given~~-~~ ZAR 100=USD 15 to compensate for their time.

~~Despite~~In addition to~~"~~ using a moderator guide, the interviewer probed on the questions and was able to gather more detailed responses from the participants. Participants were asked questions about their perceptions on ideal body weight, what informed these perceptions, personal experiences of, and attitudes towards body weight changes. In addition, they were asked questions about causes of weight change and reasons why weight changes measured at the clinic may differ from those reported by the patient. The HCWs and research staff were also asked questions about recent experiences of consultations with PLHIV around body weight. Images of body figures of different body shapes developed by Stunkard were used to explore perceptions about body size and body image. The figures ranged from one to nine where one is the thinnest body type and nine is the largest type[~~(~~17]~~)~~. There were separate figures for females and males. Participants were shown the images and were asked which body image represented them, which body image they preferred, and the most attractive body image.

Fig 1 The Stunkard scale of female body images shown to participants[~~(~~17]~~)~~

Fig 2 The Stunkard scale of male body images shown to participants[~~(~~17]~~)~~.

Discussions were conducted in local languages IsiZulu, Sesotho, Setswana, and English. All discussions were tape-recorded to ensure that all the points were captured. All interviews were transcribed verbatim, and interviews done in local languages were translated to English.

Ethical considerations

The study protocol was approved by the University of the Witwatersrand Human Research Ethics Committee, Johannesburg (M 120343); the University of Cape Town, Cape Town Human Research Ethics Committee, South Africa (106/2012); and the London School of Hygiene & Tropical Medicine Research Ethics Committee UK (6165). All participants were informed about the study and gave written consent to participate and be recorded during the FGDs. Audio recordings, field notes, and transcripts were identified only by a unique study number. All study records were stored securely in locked filing cabinets where access to the records ~~i~~was restricted to specified study team members.

Data analysis

A codebook was developed by two researchers (TD and FM) using inductive coding. Data were analysed manually using Excel. The two researchers independently inductively coded the transcripts and agreed on the emerging themes. Two data coders manually coded the data (TD and FM). TD has a Masters’~~s~~ degree and FM has an Honours degree. The senior author is an experienced qualitative researcher with a doctoral degree and supervised the data analysis (JM). The data coders manually coded a sample of the transcripts and the senior author (JM) qualitatively and quantitatively checked intercoder reliability between the coders. Inductive thematic analysis was employed to develop themes. Both males and females were included to account for variation in perceptions by gender. We used an Excel spreadsheet to monitor the number of steps that were followed during the manual coding process. Transcripts and findings were not returned to participants for comment. The COREQ

(Consolidated criteria for REporting Qualitative research) checklist was used to check the content of this manuscript.

Results

Seven FGDs (labelled 1-7) were held between 6th and 21st August 2014, with 6-12 participants in each focus group. The main identified themes were: i) negative attitudes towards weight loss; ii) stigma and loss of social support associated with weight loss; iii) psychological impact of weight loss; iv) changes in dressing due to weight changes.

Main theme 1: Perceptions and attitudes towards weight change

Sub-theme (i): Perceptions towards weight change

Participants in all the FGDs for adult PLHIV attending for HIV care expressed high level of awareness of the possible physical body changes experienced by PLHIV and on ART such as weight loss, weight gain and associated changes to body shape. The participants were aware of the main causes of weight loss, and they linked weight loss to poor diet, stress, active TB ~~infection~~, HIV/TB treatment side effects, and non-adherence to HIV/TB treatment. It was reported that denial or failure to accept and embrace one's HIV positive status also resulted in weight loss.

> *I am saying, another thing that can make a person to lose weight is that because I am HIV positive, I must check whether I have TB or not because that also causes a person to lose weight (FGD 3, Females).*

Participants in the separate groups were asked how they knew that they were losing weight. Both male and female participants reported that they noticed that they had lost weight if their clothes were not fitting well.

HCWs and research staff indicated that weight loss could be caused by other health conditions among PLHIV. As such, when they noted weight loss in a PLHIV attending for HIV care, they reported that they would usually or ideally check the PLHIV's viral load, CD4 count, investigate for TB and other opportunistic infections, screen for cervical cancer, and test for diabetes.

Both PLHIV and HCWs identified ART side effects as one of the causes of weight gain and change in body shape among PLHIV:

> *Let me say, you can see that I am slender but if I take these tablets, you see and acquire a big stomach, they will then say he is in this condition, he is now taking these tablets, isn't these tablets are making him to have a big stomach* (FGD 1, Males).

Participants from the PLHIV's FGDs were happy to gain weight. They highlighted that weight gain was a sign that they were adhering to HIV treatment and that they were responding well to treatment:

> *… if we gain weight, I believe that we are happy, because it means that no, we are happy, we are eating well, we are doing things the right way and that our medication is working well but if we lose it, we are worried because it's like we are not taking these tablets* (FGD 5, Mixed).

PLHIV also perceived weight gain as a sign that one has accepted and embraced their HIV positive status:

> *It means that you are relaxed you are not obsessing over your HIV status, you see, you do not put yourself under stress, you think that I do have tablets. If I take tablets everything is okay* (FGD 1, Males).

Some PLHIV described lipodystrophy as disproportional weight gain. While PLHIV indicated that they would prefer weight gain to weight loss, they highlighted that sometimes when one gains weight, it might not be proportional to one's body size because they will gain weight on the upper body and lose weight on the lower part of the body, and this worried them:

> *You feel good when you gain, but what you do not like is that sometimes you can gain on the upper part and lose weight on the lower part. So that is what makes us sad* (FGD 4 Females).

Sub-theme 2: Attitudes towards weight loss

There were both positive and negative attitudes towards weight loss. Some participants revealed that excessive weight gain was not healthy, so they adopted healthy lifestyle including regular exercise and celebrated weight loss after gaining excessive weight, as shown in the quote below:

> ~~*When I thought about the scale I was so excited because I have been weighing 152, so I had to hit the gym whatever it took. So today when I thought about coming to the scale I was excited. I wanted to see if this 152 had gone down. ((They all laugh)). When I got there I found out that it had gone down it's 62.5. and I was happy*~~ ~~(FGD 3, Females).~~

> *I was happy today because I had lost a lot of weight (FGD 3, Females).*

Majority of the participants demonstrated negative attitudes toward weight loss and some associated weight loss~~it~~ with the side effects of ART. Such participants reported that they would consider stopping ART to gain weight:

For me sometimes, I get a feeling that maybe these ARVs do not get along with my system and then have another feeling that hey I will stop taking them since I do not see its results, I keep losing weight, that is why most of the time you find that after people have started ARVs.., you find that a person does not take them anymore and they will say, if I don't take them I regain my weight, (FGD 4, Females).

Participants further reported that there were some nurses who reproached them for delaying seeking health care when they visited the clinic with sickness and showing signs of weight loss:

You see at the clinic, the nurses scold at you if you visited them when you were really too sick and badly affected, [they] can see that you have lost weight, the nurse scolds at you and say, you are this and that why did you not come and visit me (says P mimicking a female nurse) they scold at you there. (FGD, 1 Males).

The HCWs reported that they would become inquisitive about adult PLHIVs lifestyles if they showed signs of gaining weight as highlighted by a dietician in the HCWs group:

If they are gaining weight, we want to know if they are exercising or not (FGD 7, Dieticians and doctors).

In addition to weighing PLHIV on a scale, HCWs stated that they used alternative ways to identify if PLHIV had lost weight without openly using the words "weight loss" to get accurate answers and avoid being seen as stigmatizing PLHIV. The HCWs reported that they would instead ask participants if the size of their dress has changed or if family members had commented on changes in their body shape:

'*Has your dress size changed?' could be the alternative question* (FGD 7, Dieticians and doctors).

And then you can ask sometimes, if anyone, your friend, or family complained that your body shape has changed (FGD 7, Dieticians and doctors).

Main theme 2 : Ideal body size and shape

The participants were aware that weight change also alters both body size and shape. To gain insights into the participants' thoughts on their perceptions of ideal body size and shape, they were shown both male and female Stunkard body image figures(17). The FGDs were shown both male and female images. The majority of male participants selected body image figure number five5 in the middle as the ideal figure for men. Some men suggested that women should be the same size as men and others reported that women should be slightly bigger because they bear children. Female participants selected body image figure number six as the ideal body type because the image had a mid-sized proportional body. The choice of ideal body size was influenced by media for both women and men as indicated in the responses below:

I think influence in terms of being muscular has a lot to do with media. TV gives you the suggestion that someone who is muscular is more acceptable to the community (FGD 2, Males).

they make jokes out of us, as women, they say check her out she has unnecessary fats on the waist area, you know it would have been better if I found someone like Beyoncé [female celebrity], you see. (FGD 4, Females).

Participants further reported that culture also influenced how people perceive ideal body shape especially for women.

My sister is right about culture, Zulus prefer fat women. They say she is well-fed (FGD 3, Females).

> *And I think we have a black culture that a black woman has to be round (Participants agreeing with one another), they will just be worried, when number 4, they won't even look at you (FGD 6, HCW and Research Staff).*

HCWs revealed they would ask PLHIV who had signs of lipodystrophy whether other people had commented on the changes in their body shape or size. According to the HCWs, PLHIV were more worried about changes in body shape or size and not so much about weight loss. It was uncommon for PLHIV to visit the clinic because they were worried about weight loss, but they usually visited the clinic when they noticed changes in body shape:

> *What I have noticed is that it is mostly PLHIV who have got lipodystrophy, they are the ones who are more worried about their weight that's one thing I have realised because they are saying, no, I no longer have my buttocks, they are gone, my legs are now thinner. (FGD 7, Dieticians and doctors).*

HCWs stated that when they noticed changes in body shape and size among the PLHIV, they changed the regimen for those who were taking stavudine. PLHIV on the second-line ART regimen (at the time of the study, 2014, azidothymidine, lamivudine, lopinavir/ritonavir), were counseled and some were referred to government-funded plastic surgery services. HCWs also encouraged PLHIV to exercise and improve their diet~~s~~.

FGDs with HCWs and research staff revealed that PLHIV who lost weight were referred to dieticians, social workers, psychologists, and religious leaders for support. PLHIV with socio-economic challenges were referred to the South African Social Security Agency for the Disability Grant. HCWs also revealed that they involved the families of PLHIV experiencing weight loss to explore potential causes.

Main theme 3: Negative impact of weight loss on participant's daily life

Sub-theme 1: Stigma and loss of social support

Participants reported that changes in physical body size, particularly weight loss, resulted in them being stigmatized in the community. Some of the participants reported that they experienced body shaming, name-calling, gossip, and discrimination because they had lost too much weight. Participants in the FGDs lamented that people in the communities would quickly conclude that a person has AIDS once one begins to lose weight. The participants reported that those who show signs of weight loss are said to have bought OMO (washing powder) because the HIV "washed" way the person's body the way OMO washes dirt:

> *They will say, so and so bought OMO can you actually see so and so, so in that manner they call you by those names when you are on the passing by, and you would think my goodness. They do this so that you cannot understand you see, you may not know why you are regarded as having bought OMO, when you look around no one bought OMO, they regard you as that since you lost weight* (FGD 1, Males).

Participants with lipodystrophy were stigmatized and were called names such as "high heels" and KFC because of having thin legs and a fat upper body:

> *Thin legs. Do you know what a KFC drumstick looks like? When they see you, they say: It's KFC! She went to buy KFC* (FGD 3, Females).

Participants also reported stigmatizing attitudes from both community and family members which include being ignored and people keeping some distance from them:

Mostly, if they know that you are positive through appearance, the loss of weight, they start ignoring you and mostly the names like killer (FGD, 2 Males).

Even your family still does that thing, they say hey, don't stand too close to me you will infect me with this thing (FGD 4 Females).

Some participants in the FGDs reported that PLHIV who show signs of weight changes may lose social support from their families and they attributed this to a lack of adequate knowledge on HIV:

So, this is what happened …, my mother is an elderly person so she does not know the difference between HIV and AIDS. When my brother took a cup and drank from it, she would want nothing to do with that cup. So, I called her and tried to teach her about what HIV and AIDS are. I said I'm asking you to please support him (FGD 3, Females).

Sub-theme 2: Psychological impact of weight loss

The participants in focus group discussions revealed that when some PLHIV discover that they have lost weight, they become so stressed:

The thing is, you just become stressed out, you just cannot concentrate on the things that you are doing at home, do you understand, you become short-tempered, you ask yourself what is going on, what did I do, what mistake did I make in order for me to lose weight in this manner (FGD, 4 Female).

Some PLHIV revealed they feel anxious and stressed when they are asked to stand on the scale and be weighed at the clinic:

Before standing on the scale, I was afraid that they are about to weigh me now and it's possible that I may have lost weight (FGD1, Males).

Sub-theme 3: Changes in dressing

When they notice that they have lost weight, participants reported that they would change the way they dress to cover signs of weight loss:

> *So, now that I have lost weight, when I get dressed and wear something that hides my bums they say why are you making yourself a granny? You must wear the latest fashion, and I say: hey I am not a child (FGD, 3 Female).*

Discussion

Participants demonstrated a strong awareness that some physical body changes such as weight gain and weight loss may be symptoms of HIV and AIDS. The participants further indicated that negative weight changes, i.e., weight loss, attract stigmatizing attitude from society and that stigma remains a huge concern for PLHIV. Because of this, the participants preferred gaining to losing weight. These findings are consistent with findings from other studies in South Africa that found that PLHIV would rather gain weight than be thin and have people assume that they are infected with HIV[(5,12]). Corroborating our finding, a study in Uganda found that women living with HIV did not want to lose weight because weight loss could lead to people suspecting that their HIV seropositive status. Women living with HIV who lost weight in the same study also reported that they felt stigmatized, and it affected their adherence to ART[(18]). The awareness by PLHIV that physical body changes, particularly weight loss, can be a symptom of HIV and AIDS and the stigma associated stigma towith weight loss may have had a self-fulfilling effect on PLHIV self-reporting on weight changes, which can thus explain the discrepancy between PLHIV self-reported and measured weight loss. The discrepancy may mean that PLHIV are more sensitive about weight loss since they are so aware of its association with HIV and AIDS, and this can make them report

Formatted: Pattern: Clear (Yellow)

Formatted: Pattern: Clear (Yellow)

Formatted: Pattern: Clear (Yellow)

~~weight loss even if they have not lost weight.~~ The finding that it was rare for PLHIV to seek healthcare services because of weight loss concerns may negatively affect efforts to screen for and diagnose TB ~~and~~ early ~~diagnosis~~.

In our study, HCWs reported that because of the stigma attached to weight loss, they avoid asking PLHIV direct questions about weight loss because the fear of the stigma associated with weight loss may hinder PLHIV from telling the truth. The stigma associated with weight loss may make the WHO screening tool less sensitive, specifically the question on weight loss. A potential response could be to weigh people seeking health care services ~~PLHIV~~ instead of asking them the question on weight loss. The stigma associated with HIV and TB needs to be addressed for example by educating, through community outreach campaigns, both PLHIV, family members, and the community on HIV and TB using tool kits that have been developed to address TB-related stigma[~~(~~19–21]~~)~~.

Despite the preference to gain rather than lose weight amongst the majority of participants, some participants were aware that excessive weight gain has repercussions and reported adopting a healthy lifestyle including regular exercise and celebrated weight loss. A study conducted in Cape Town, South Africa found that participants preferred to be overweight and at risk of acquiring cardiovascular diseases, rather than to lose weight and be stigmatised as infected with HIV(5). It is important to educate PLHIV that excessive weight gain may lead to obesity and increase the risk of non-communicable diseases. Therefore, PLHIV should be encouraged to exercise and eat well to reduce the risk of these diseases[~~(~~8,13,22]~~)~~.

The psychological impact of weight changes leading to non-adherence found in our study has been reported in other studies[~~(~~8,13,18,22]~~)~~. Lipodystrophy is a concern

among PLHIV~~HIV-positive people~~. The finding that changes in body image affect PLHIV psychologically, with some considering stopping treatment, is consistent with other studies. A study on lipodystrophy among PLHIV in Tanzania found that changes in body shape may affect PLHIV psychosocial function, quality of life and may lead to PLHIV considering stopping treatment[~~(~~22]~~)~~.

Our study found that fuller women were considered to have the ideal body shape. This finding is consistent with findings from other previous studies. A study conducted among adolescents in Durban revealed that female respondents desired to be thick and curvaceous, and males desired to be bigger and muscular, and this was linked to the influence of cultural norms on understanding of an ideal body shape[~~(~~13]~~)~~. Similarly, a study conducted in Cape T~~t~~own found that cultural beliefs appeared to have a significant influence on the women interviewed, despite more than half of the participants having a high school education. Some women in this study reported that women are culturally expected to have a fuller figure[~~(~~5]~~)~~

Stavudine is now rarely used and so few PLHIV should newly develop lipodystrophy. Those with established lipodystrophy should have been switched to a different regimen which does not cause lipodystrophy, but these people may need support and reassurance to continue their current ART[~~(~~11]~~)~~.

Our study findings are significant in today's terms as South Africa still has a high estimated HIV prevalence rate of 17.8 among adults (15-49 years) and 75% of PLHIV are on ART[~~(~~23]~~)~~. TB remains a leading cause of death among PLHIV so understanding their perceptions around weight change~~s~~ and how this may affect TB symptom screening is important. In addition, since this work, the new first-line treatment regimen for PLHIV includes dolutegravir, which has been found to have

weight gain as a side effect. Hence, perceptions on weight gain highlighted in our paper remain relevant [(18,24]).

Strengths and limitations

One of the strengths of this study is that we conducted FGDs with both males and females and managed to get perceptions on weight change from both genders. However, the study was not without limitations. The sample size of the PLHIV who were interviewed was small and may not be representative of all PLHIV at the facilities. Again, PLHIV may not have been able to disclose sensitive personal information in focus groups. Despite these limitations, we managed to gain insights from PLHIV as well as HCWs on people's perceptions of weight change among people who live with HIV. The potential bias was minimised by the fact that the facilitator was an independent researcher, therefore would have been perceived to have adopted a neutral stance on the issues.

Conclusion

The majority of PLHIV preferred to gain weight due to fear of stigma associated with weight loss. Weight loss is associated with HIV /AIDS, suggesting that people attending for HIV care may underreport weight loss in the context of a TB symptom screening tool. This would result in reduced sensitivity of the WHO TB screening tool. Findings suggest that we need either alternative ways to determine weight loss, or screening tools for TB which are less dependent on reported symptoms. Furthermore, the policy implications of our paper suggest the importance of strengthening innovative TB testing strategies such as Targeted Universal Test and Treat (TUTT) which tests high risk groups including PLHIV irrespective of reporting TB symptoms or ART status [(25]).

Acknowledgments

The authors are grateful to the Department of Health nurses and staff at the facilities, the XPHACTOR research staff, and all the participants.

Author contributions

ADG, SC, and YH conceptualized the study. SEQ conducted the FGDs. TD and FM performed the data analysis with guidance from JM. NN was the project manager. TD drafted the paper and all authors provided critical reviews, edited drafts, and approved the version to be published.

Reference List

1. Adamu AL, Gadanya MA, Abubakar IS, Jibo AM, Bello MM, Gajida AU, et al. High mortality among tuberculosis patients on treatment in Nigeria: A retrospective cohort study. BMC Infect Dis. 2017;17(1):1–12.
2. Tornheim JA, Dooley KE. Tuberculosis Associated with HIV Infection. American Society for Microbiology. 2017;5(1):1–16.
3. World Health Organization. Tuberculosis Key Facts [Internet]. 2023 [cited 2023 Jul 5]. Available from: https://www.who.int/news-room/fact-sheets/detail/tuberculosis
4. WHO. WHO Consolidated Guidelines on Tuberculosis. Module 2 Screening [Internet]. Who. 2021 [cited 2024 Sep 10]. Available from: https://iris.who.int/bitstream/handle/10665/340255/9789240022676-eng.pdf?sequence=1
5. Matoti-Mvalo T, Puoane T. Perceptions of body size and its association with HIV/AIDS. South African Journal of Clinical Nutrition. 2011;24(1):40–5.
6. Li N, Spiegelman D, Drain P, Mwiru RS, Mugusi F, Chalamilla G, et al. Predictors of weight loss after HAART initiation among HIV-infected adults in Tanzania. Aids. 2011;26(5):577–85.
7. Tate T, Willig AL, Willig JH, Raper JL, Moneyham L, Kempf MC, et al. HIV infection and obesity: Where did all the wasting go? Antivir Ther. 2012;17(7):1281–9.
8. Power R, Tate HL, McGill SM, Taylor C. A qualitative study of the psychosocial implications of lipodystrophy syndrome on HIV positive individuals. Sex Transm Infect. 2003;79(2):137–41.
9. Lakey W, Yang LY, Yancy W, Chow SC, Hicks C. Short Communication: From wasting to obesity: Initial antiretroviral therapy and weight gain in HIV-infected persons. AIDS Res Hum Retroviruses. 2013;29(3):435–40.
10. Sax PE, Erlandson KM, Lake JE, McComsey GA, Orkin C, Esser S, et al. Weight gain following initiation of antiretroviral therapy: Risk factors in randomized comparative clinical trials. Clinical Infectious Diseases. 2020;71(6):1379–89.
11. The South African Antiretroviral Treatment Guidelines 2010 [Internet]. 2010 [cited 2024 Apr 19]. Available from: https://sahivsoc.org/Files/Summary_The_South_African_Antiretroviral_Treatment_2010.pdf
12. Hurley E, Coutsoudis A, Giddy J, Knight SE, Loots E, Esterhuizen TM. Weight evolution and perceptions of adults living with HIV following initiation of antiretroviral therapy in a South African urban setting. South African Medical Journal. 2011;101(9):645–50.

13. Nyamaruze P, Govender K. € I like the way i am, but i feel like i could get a little bit bigger": Perceptions of body image among adolescents and youth living with HIV in Durban, South Africa. PLoS One [Internet]. 2020;15(1):1–10. Available from: http://dx.doi.org/10.1371/journal.pone.0227583

14. Hanifa Y, Fielding KL, Chihota VN, Adonis L, Charalambous S, Foster N, et al. A clinical scoring system to prioritise investigation for tuberculosis among adults attending HIV clinics in South Africa. PLoS One. 2017 Aug 1;12(8).

15. Pillai N, Foster N, Hanifa Y, Ndlovu N, Fielding K, Churchyard G, et al. Patient costs incurred by people living with HIV/AIDS prior to ART initiation in primary healthcare facilities in Gauteng, South Africa. PLoS One. 2019 Feb 1;14(2).

16. Auld AF, Kerkhoff AD, Hanifa Y, Wood R, Charalambous S, Liu Y, et al. Derivation and external validation of a risk score for predicting HIV-associated tuberculosis to support case finding and preventive therapy scale-up: A cohort study. PLoS Med. 2021 Sep 1;18(9).

17. Sorensen TIA, Stunkard AJ. Does obesity run in families because of genes? An adoption study using silhouettes as a measure of obesity. Acta Psychiatr Scand. 1993;67–72.

18. Alhassan Y, Twimukye A, Malaba T, Myer L, Waitt C, Lamorde M, et al. "It's only fatness, it doesn't kill": a qualitative study on perceptions of weight gain from use of dolutegravir-based regimens in women living with HIV in Uganda. BMC Womens Health. 2022 Dec 1;22(1).

19. Kidd Ross, International HIV/AIDS Alliance., ZAMBART Urban Tuberculosis Project. Understanding and challenging TB stigma toolkit for action : introduction to TB stigma, more understanding and less fear about TB [Internet]. International HIV/AIDS Alliance; 2009 [cited 2024 Sep 10]. 68 p. Available from: https://assets.publishing.service.gov.uk/media/57a08b3ced915d622c000bb7/TB_and_Stigma_May09.pdf

20. AIDS Foundation of South Africa. Reducing Stigma related to Gender, Culture and HIV/AIDS [Internet]. 2017 [cited 2024 Sep 10]. Available from: https://www.aids.org.za/wp-content/uploads/2018/05/Facilitator%E2%80%99s-Guide-Stigma-Related-to-Gender-Culture-HIV.pdf

21. Nyblade L, Stangl A, Weiss E, Ashburn K. Combating HIV stigma in health care settings: What works? Vol. 12, Journal of the International AIDS Society. John Wiley and Sons Inc.; 2009.

22. Njelekela M, Mpembeni R, Muhihi A, Ulenga N, Aris E, Kakoko D. Lipodystrophy among HIV-Infected Patients Attending Care and Treatment Clinics in Dar es Salaam. 2017;2017(October 2004).

23. World Health Organisation. South Africa HIV Country Profile 2023 [Internet]. [cited 2024 Sep 11]. Available from: https://cfs.hivci.org/index.html

24. Brennan AT, Nattey C, Kileel EM, Rosen S, Maskew M, Stokes AC, et al. Change in body weight and risk of hypertension after switching from efavirenz to dolutegravir in adults living with HIV: evidence from routine care in Johannesburg, South Africa. EClinicalMedicine. 2023 Mar 1;57.

25. Martinson NA, Nonyane BAS, Genade LP, Berhanu RH, Naidoo P, Brey Z, et al. Evaluating systematic targeted universal testing for tuberculosis in primary care clinics of South Africa: A cluster-randomized trial (The TUTT Trial). PLoS Med. 2023 May 1;20(5 May).

PLOS ONE

1265 Battery Street, Suite 200

San Francisco, CA 94111

United States

16 April 2024

Dear Editors in chief

We would like to thank the editor and reviewers for the very valuable comments made. They have certainly enabled us to strengthen the quality of our paper and increase its relevance. In the present letter, we have a table showing the comments raised and our responses. Both clean and marked-up copies of the revised manuscript are attached.

Regards,

Tanyaradzwa Dube, corresponding author.

Responses to the Editors Comments

1. Please ensure that your manuscript meets PLOS ONE's style requirements, including those for file naming.	Agreed. This has been done.
2. In the online submission form you indicate that your data is not available for proprietary reasons and have provided a contact point for accessing this data. Please note that your current contact point is a co-author on this manuscript. According to our Data Policy, the contact point must not be an author on the manuscript and must be an institutional contact, ideally not an individual. Please revise your data statement to a non-author institutional point of contact, such as a data access or ethics committee, and send this to us via return email. Please also include contact information for the third party organization, and please include the full citation of where the data can be found.	Agreed. Data has been submitted with the revised manuscript.

3. Please upload a copy of Figure 4 and 5, to which you refer in your text on page 12 and 18. If the figure is no longer to be included as part of the submission please remove all reference to it within the text.	Figures with Stunkard body image figures have been added to the manuscript (Fig1 and Fig 2). The figures have male and female body image figures ranging from 1 to 9 and participants were choosing the image they preferred.
4. Please include captions for your Supporting Information files at the end of your manuscript, and update any in-text citations to match accordingly. Please see our Supporting Information guidelines for more information: http://journals.plos.org/plosone/s/supporting-information.	Done
5. The work submitted is noted. Could the authors address the constructive Reviewer comments?<br><br>Discuss the significance of the study findings in today's terms and add the relevant practical and policy implications<br><br>the data is old so please justify, especially noting the chronicity of HIV in South Africa	Our study findings are significant in today's terms as South Africa still has a high estimated HIV prevalence rate of 17.8 among adults (15-49 years) and 75% of PLHIV are on ART(23). TB remains a leading cause of death among PLHIV so understanding their perceptions around weight change and how this may affect TB symptom screening is important. In addition, since this work, the new first-line treatment regimen for PLHIV includes dolutegravir, which has been found to have weight gain as a side effect. Hence, perceptions on weight gain highlighted in our paper remain relevant (18,24).<br><br>Page 20 line 431-436<br><br>The policy implications of our paper suggest that in addition to TB screening, we should continue using innovative TB testing strategies such as Targeted Universal Test and Treat (TUTT) which tests high risk groups including PLHIV irrespective of reporting TB symptoms so that PLHIV with TB are not missed due to inaccurate self-reporting on TB symptoms.<br><br>See line 458 page 20

Responses to Reviewer 1

Comment	Response
1. The aspect of weight loss in relation to the tool used for screening tuberculosis seems to appear only at the background. Considering the relationship between HIV/AIDS and TB, as well as the high chances of people living with HIV/AIDS to contract tuberculosis due to the compromise of their immune system, it would be nice to have it appear up front even from the title level. This will certainly give a sense of concern to the people living with HIV/AIDS and the "why we should worry" about their perceptions and attitudes on any weight change particularly the weight loss.	Agreed. The title has been revised to include TB screening among PLHIV.<br><br>"*If we lose it, we are worried"*: Individual and provider level perceptions and attitudes towards weight change among people living with HIV screening for tuberculosis in routine health care settings in Gauteng Province, South Africa.<br><br>Also the risk of developing TB among people living with HIV was included on page 4 (line72-73) in the background
2. Lastly, on data availability and restrictions, if the author indeed de-identified the transcripts with a unique study number, then there should be no fears of bridging confidentiality if made public.	Agreed. Data has been submitted with the revised manuscripts.

Responses to Reviewer 2

Comment	Response
1. this is misleading. your population comes from one part of the country (Gauteng) ..so your tiltle should reflect this. Are these findings generalizable ..then i wont have a problem!	Agreed. The title has been changed to reflect Gauteng Province<br><br>"*If we lose it, we are worried"*: Individual and provider level perceptions and attitudes towards weight change among people living with HIV screening for tuberculosis in routine health care settings in Gauteng Province, South Africa.

2. Line 125<br>Research Assistants and HCWs were conducted via email<br><br>do you mean contacted? If not, then this sentence does not make sense!!	Yes. We meant contacted. This has been edited on page 6 line 126
3. Quote on line 236:<br>*When I thought about the scale I was so excited because I have been weighing 152, so I had to hit the gym whatever it took. So today when I thought about coming to the scale I was excited. I wanted to see if this 152 had gone down. ((They all laugh)). When I got there I found out that it had gone down it's 62.5. and I was happy* (FGD 3, Females).<br><br>Reviewer's comment: not sure if this is kgs ..also the drop in weight then is significant!	Yes, the participant meant kg's. We agree that the weight loss reported her was massive and we quoted a participant who may have gotten the figures wrong. We have decided to use a different quote to support the finding that other participants had a positive attitude towards weight loss on page 12 line 244<br><br>*I was happy today because I had lost a lot of*<br><br>*weight (FGD 3, Females).*
4. This sentence contradicts what you have been saying all along that PLHIV under-report weight loss but here you are saying that they report weight loss even though they have not lost weight!!!	We agree with the reviewer that the data primarily support the idea that PLHIV are likely to underreport weight loss and have removed the two sentences in the discussion that talk about overreporting.
5. So maybe somewhere in your manuscript you need to highlight the probability of TB diagnosis in PLHIV.<br><br>also is weight loss a function of the drug or a symptom of TB in these patients or both?	The risk of developing active TB disease is estimated to be 16 (uncertainty interval 14–18) times greater in PLHIV than those without HIV infection. (WHO, 2023). The statement has been added to the background line 72-73.<br><br>Line 198-204 highlight participant's perceptions on possible causes of weight loss which includes side effects of medication and TB disease. Line 246 refers to the idea that ART might cause weight loss. A quote has been added to support the statement regarding TB causing weight loss.<br>*"I am saying, another thing that can make a person to lose weight is that because I am HIV positive, I must check whether I have TB or not because that also causes a person to lose weight" (FGD 3, Females).*

6. Line 386 Statement: A potential response could be to weigh PLHIV instead of asking them the question on weight loss<br><br>Comment: Surely this applies to anyone from this community regardless of their HIV status?	Agreed. This has been changed to: A potential response could be to weigh people seeking health care services instead of asking them the question on weight loss. (Lines 388-389) page 18
7. Line 396-398<br><br>Statement: It is important to educate PLHIV that excessive weight gain may lead to obesity and increase the risk of non-communicable diseases<br><br>Comment: surely here a better measure would be BMI. the researchers had the opportunity to weigh the participants. i am not sure if the height was taken. if so a BMI could have been calculated and then the quest of excessive weight gain would have been easy to recognize in the context of a BMI score	Yes BMI was calculated as part of the study procedure. In our paper, we recommend educating PLHIV about the negative effects of weight gain which they perceived to be favorable from the qualitative findings. Page 18 Line 400
8. Line 400<br>Statement: The psychological impact of weight changes leading to non-adherence found in our study has been reported in other studies[7,12,20].<br><br>Comment: where is this reported and non-adherence to what?	It was reported on page 12 (lines 245-253). Some participants reported that they would consider stopping treatment if they kept losing weight. We have clarified the treatment they would consider stopping which is antiretroviral treatment (line 247).
9. Line 408 It is important to show the outcomes of similar studies in different settings or cultures and comment on these in the context of your findings.	The discussion section (pages 18-19) refers to studies conducted in other parts of South Africa like Cape Town (lines 415-422) and Durban (lines 411-414) as well as studies on weight perceptions conducted in other countries such as Tanzania (lines 406-409) and Uganda (lines 378-481).
10. How confident are the researchers that asking about a change of dress size can be used as proxy for weight loss or gain. what if they simply dont answer truthfully to this question? Are there studies that have shown dress size to be a reliable proxy for weight gain or loss? Studies	Since this was a qualitative study, we asked about perceptions of weight loss. Participants in our study reported that changes in dress sizes made them realize they lost weight. HCWs in our study recommended that asking participants changes in clothing size could be better than directly asking unintentional weight loss questions. Asking clothing size is a self-report measure which can get biased responses, but we feel is more acceptable to patients

	rather than directly asking about weight loss. A study in Netherlands found that self-reported clothing size appeared to predict cancer risk independently of body mass index, suggesting that self-reported clothing size can be a useful measure to consider in epidemiologic studies.<br><br>Hughes et al (2009) *Self-reported clothing size as a proxy measure for body size*
