# Supplementary material for: “If we lose it, we are worried”: Individual and provider level perceptions towards weight change among people living with HIV who undergo TB screening in routine health care settings in Gauteng Province, South Africa
Source: PLoS One. 2025 Sep 22;20(9):e0331904. doi: 10.1371/journal.pone.0331904 (PMC12453174; doi:10.1371/journal.pone.0331904)
Supplement: Attachment — Submitted filename: PONE-D-24-15618_R2_reviewer august 2025.pdf [file pone.0331904.s009.pdf]

PLOS ONE

"If we lose it, we are worried": Individual and provider level perceptions towards weight change among people living with HIV who undergo TB screening in routine health care settings in Gauteng Province, South Africa.

--Manuscript Draft--

Manuscript Number:	PONE-D-24-15618R2
Article Type:	Research Article
Full Title:	"If we lose it, we are worried": Individual and provider level perceptions towards weight change among people living with HIV who undergo TB screening in routine health care settings in Gauteng Province, South Africa.
Short Title:	Individual and provider level perceptions towards weight change among people living with HIV South Africa.
Corresponding Author:	Tanyaradzwa Nicolette Dube, MA<br>The Aurum Institute<br>Johannesburg, SOUTH AFRICA
Keywords:	HIV, TB screening, Weight change, ART, South Africa, WHO screening tool
Abstract:	Background<br>HIV weakens the immune system, increasing the risk of tuberculosis (TB) in people with living HIV (PLHIV). People living with HIV and on antiretroviral treatment (ART) often experience physical body size changes. Studies have found a significant discrepancy between PLHIV's self-reported weight loss and their measured weight loss when being screened for TB using the WHO tool. To understand this inconsistency, a qualitative sub-study was conducted to explore perceptions and attitudes towards weight change among adults attending HIV care, as well as health care workers in public clinics in Gauteng, South Africa.<br>Methods<br>Our qualitative study was nested within the XPHACTOR study. A total of seven focus group discussions were conducted, five with adult participants attending for HIV care and two with health care workers and research staff in clinics around Gauteng. Inductive thematic analysis was used to analyse the data.<br>Findings<br>The majority of PLHIV preferred to gain weight due to fear of stigma associated with weight loss. Weight loss is associated with HIV/AIDS, suggesting that people attending HIV care may underreport weight loss in the context of a TB symptoms screening tool because they fear stigma. Participants reported that weight changes impacted their daily lives and had psychological effects on them. Some PLHIV described lipodystrophy as disproportional weight gain. Culture and media have an influence on the perception of ideal body size and shape for both men and women.<br>Conclusions<br>Underreporting weight loss might result in poor sensitivity of the WHO TB screening tool and suggests that we need either alternative ways to determine weight loss or screening tools for TB that are less dependent on reported symptoms.
Order of Authors:	Tanyaradzwa Nicolette Dube, MA
	Fezeka Mboniswa, BPsych (Honors)
	Samkelisiwe Ethel Qwana, MA
	Salome Charalambous, PhD
	Nontobeko Ndlovu, Msc
	Alison D Grant, PhD
	Yasmeen Hanifa, PhD
	Johannes Machinya, PhD
Response to Reviewers:	Comment<br>We note that this data set consists of interview transcripts. Can you please confirm that

Powered by Editorial Manager® and ProduXion Manager® from Aries Systems Corporation

all participants gave consent for interview transcript to be published?

If they DID provide consent for these transcripts to be published, please also confirm that the transcripts do not contain any potentially identifying information (or let us know if the participants consented to having their personal details published and made publicly available). We consider the following details to be identifying information:

- Names, nicknames, and initials
- Age more specific than round numbers
- GPS coordinates, physical addresses, IP addresses, email addresses
- Information in small sample sizes (e.g. 40 students from X class in X year at X university)
- Specific dates (e.g. visit dates, interview dates)
- ID numbers

Response:
Yes, the participants consented to having their de identified data shared in published journals.

The transcripts have been checked to remove any identifying information as recommended.

Comment 1

Line 1

“If we lose it, we are worried”: Individual and provider level perceptions and attitudes towards weight change among people living with HIV screening for tuberculosis in routine health care settings in Gauteng Province, South Africa.

please read this title again. The grammar seems incorrect...please check!
perhaps consider : perceptions and
attitudes towards weight change among people living with HIV who undergo TB screening

Response 1

Line 1, page 1

Agreed changed to

Individual and provider level perceptions towards weight change among people living with HIV who undergo TB screening in routine health care settings in Gauteng Province, South Africa.

Comment 2

Line 104 page 5
We were interested to know whether attitudes to weight and body size might affect how people responded to the questions in the WHO TB symptom screening tool and whether people with stavudine-related lipodystrophy might describe the change in body shape as weight loss.

Comment: This sentence should read “We sought to investigate whether…….
Line 103 page 5

Response 2

Agreed and changed to: We sought to investigate whether attitudes to weight and body size might affect how people responded to the questions in the WHO TB symptom screening tool and whether people with stavudine-related lipodystrophy might describe

Powered by Editorial Manager® and ProduXion Manager® from Aries Systems Corporation

the change in body shape as weight loss.
Comment 3

Line 119, page 6

Tell us something more about the Focus groups...you say they were all male, all female and mixed. did you have a target group size? How many came from the XPHACTOR study and how many were invited from the ART support group? similarly, how many staff were art of the initial group and how many were invited? how did you determine sample size per group?

Response 3

Line 123-125 page 6

Adult PLHIV who were not XPHACTOR study participants were approached in-person for participation in the study via an ART support group linked to the clinic.

Lines 134 to 142

We initially planned to conduct around three FGDs with about eight to twelve participants in each group but eventually conducted seven FGDs to reach saturation. The number of discussions increased because we separated male and female FGD's (two groups per gender) plus a mixed gender discussion to capture sufficient diversity in perspectives and experiences. Sample size per group was determined by availability of participants and previous qualitative research which recommends six to twelve participants per FGD(17,18). This range ensures sufficient diversity, a balanced interaction to allow all participants to contribute as well as effective facilitation of the discussion by the moderator.

Line 200-205 page 9

Seven FGDs (labelled 1-7) were held between 6th and 21st August 2014, with 7-12 participants in each focus group. The five adult PLHIV FGDs (two males, two females and one mixed gender) had 7-12 participants in each group. The HCWs and research staff FGD comprised of ten participants (seven routine HCWs and three research staff). The doctors and dieticians FGD had seven participants (five doctors and two dieticians) who were all routine staff.

Comment 4

Line 124, page 6

Adult PLHIV who were not XPHACTOR study participants were approached for participation in the study face to face via an ART support group linked to the clinic.

Comment: sentence should read as

"Adult PLHIV who were not XPHACTOR study participants were approached in-person for participation in the study via an ART support group linked to the clinic."
Line 123 page 7

Response 4

Sentence changed as suggested.

Adult PLHIV who were not XPHACTOR study participants were approached in-person for participation in the study via an ART support group linked to the clinic
Comment 5

Powered by Editorial Manager® and ProduXion Manager® from Aries Systems Corporation

	Line 135, page 6<br><br>We initially planned to conduct around three FGDs but eventually conducted seven FGDs to reach saturation<br><br>Comment:<br>could you expand on this ....this related to the methodological approach taken:<br><br>Response 5<br>Line 136-142 page 7<br>The number of discussions increased because we separated male and female FGD's (two groups per gender) plus a mixed gender discussion to capture sufficient diversity in perspectives and experiences. Sample size per group was determined by availability of participants and previous qualitative research which recommends six to twelve participants per FGD. This range ensures sufficient diversity, a balanced interaction to allow all participants to contribute as well as effective facilitation of the discussion by the moderator.<br>Comment 6<br><br>Line 161, page 8<br><br>All interviews were transcribed verbatim, and interviews done in local languages were translated to English<br><br>tell us more on this process and language translation is a complex process. one has to make sure that the nuances are not lost during the translation process!!<br><br>Response 6<br><br>Line 161-171 page 8<br><br>More details have been added:<br><br>Translations and transcriptions were done by experienced researchers fluent in the study languages. Transcriptions were done verbatim to keep nuances for instance, almost all deliberations were captured including non-verbal cues such as short silences and laughs. Quality assurance was conducted whereby a second researcher checked the accuracy of the transcripts against audio recordings.<br><br>Comment 7<br><br>Line 186, Page 9<br>could you add a few lines on the process to reach saturation?<br><br>Response 7<br><br>Line 193-196 page 9<br><br>TD, FM and JM reviewed and refined emerging themes repeatedly until saturation was achieved when no additional themes or categories could be identified.<br><br>Comment 8<br><br>Line 189, page 9<br><br>so there were main themes and sub-themes! the main themes identified are not what you are reporting in this sentence...they are sub-themes...separate them and be clear about the main and sub-themes!<br><br>Response 8<br><br>Line 206-211 page 10

Powered by Editorial Manager® and ProduXion Manager® from Aries Systems Corporation

The main themes and sub themes were separated as following;

We identified three main themes and five sub themes. The main themes were i. perceptions and attitudes towards weight change; ii ideal body size and shape; iii negative impact of weight loss on participant's daily life. The sub themes were i) perceptions towards weight change; ii) attitudes towards weight loss; iii) stigma and loss of social support; psychological impact of weight loss; and iv) changes in dressing.

Comment 9
Line 375, page 17

please correct the grammar...you cannot start a sentence with "because"

Response 9

Line 393, page 18

Changed sentence to "As a result, the participants preferred gaining to losing weight".
Comment 10

Line 378 page 17

Corroborating our finding, a study in Uganda found that women living with HIV did not want to lose weight because weight loss could lead to people suspecting their HIV seropositive status. Women living with HIV who lost weight in the same study also reported that they felt stigmatized, and it affected their adherence to ART[18]

This should read as "The results obtained in our study were similar to the findings of a Ugandan study that found that women living with HIV did not want to lose weight as this could lead to people suspecting that they were HIV positive.

Response 10

Line 397-401, page 18

Agreed and the sentence changed as suggested.

The results obtained in our study were similar to the findings of a Ugandan study that found that women living with HIV did not want to lose weight as this could lead to people suspecting that they were HIV positive
Comment 11

Line 381 page 17

The finding that it was rare for PLHIV to seek healthcare services because of weight loss concerns may negatively affect efforts to screen for and diagnose TB early.

did your study find this or was this from the literature?

Response 11

Line 401-404 page 18
This was a potential implication based on previous studies that have shown an association between loss of weight and delayed TB diagnosis/ seeking care.

The finding that it was rare for PLHIV to seek healthcare services because of weight loss concerns may negatively affect efforts to screen for and diagnose TB early. Several studies have found an association between weight loss and delay in TB diagnosis(21–23). A study consisting of 185 people with TB in KwaZulu Natal South Africa found that weight loss was associated with delayed care(21). The researchers suggested that stigma of weight loss because of its association with other diseases could have been a key factor contributing to this delay (21).

Comment 12

Line 388, page 17

A potential alternative to asking questions about weight loss when PLHIV present for treatment of check-ups is to measure their weight at the visit and compare this to previous weight measures recorded in their file.

Response 12
Line 413-415 page 19

Agreed and changed as suggested.

Comment 13

Line 389, page 17

The stigma associated with HIV and TB needs to be addressed for example by educating, through community outreach campaigns, both PLHIV, family members, and the community on HIV and TB using tool kits that have been developed to address TB-related stigma.

please correct the grammar ..this sentence does not make sense!
Line 426-428 page 9

Response 13
Agreed sentenced revised to:

The stigma associated with HIV and TB needs to be addressed for example by educating PLHIV, family members, and the community on HIV and TB. The education can be done through community outreach campaigns using tool kits that have been developed to address TB-related stigma

Comment 14
Line 397, page 18

A study conducted in Cape Town, South Africa found that participants preferred to be overweight and at risk of acquiring cardiovascular diseases, rather than to lose weight and be stigmatised as infected with HIV.

for better context, perhaps add a few lines about this study ...how many participants, what was the objective of this study, methodology used, etc.

Response

Line 424-440 page 19

Done, additional was added.

A mixed method study including 513 adult women was conducted in Cape Town, South Africa to explore the perception among black South African women that people who are thin are infected with HIV or have AIDS. The study found that participants preferred to be overweight and at risk of acquiring cardiovascular diseases, rather than to lose weight and be stigmatised as infected with HIV[5]

Comment 15

Line 410, page 18

Our study found that fuller women were considered to have the ideal body shape. This finding is consistent with findings from other previous studies.

Comment
Add references:

Powered by Editorial Manager® and ProduXion Manager® from Aries Systems Corporation

	Response 15<br>References cited. Line 440<br><br>Matoti-Mvalo T, Puoane T. Perceptions of body size and its association with HIV/AIDS. South African Journal of Clinical Nutrition. 2011;24(1):40–5.<br><br>Nyamaruze P, Govender K. I like the way i am, but i feel like i could get a little bit bigger": Perceptions of body image among adolescents and youth living with HIV in Durban, South Africa. PLoS One [Internet]. 2020;15(1):1–10. Available from: http://dx.doi.org/10.1371/journal.pone.0227583<br><br>Comment 16<br><br>Line 431, page 19<br><br>Our study findings are significant in today's terms as TB remains a leading cause of death among PLHIV<br><br>Delete "in today's terms" in the sentence<br><br><br><br>Response 16<br>Deleted as suggested. Line 462<br><br>Our study findings are significant as TB remains a leading cause of death among PLHIV so understanding their perceptions around weight change and how this may affect TB symptom screening is important<br>Comment 17<br><br>The other concern I have is the discussion section where you compare or quote other studies ..please provide a bit more context to those studies so the reader has a sense of how similar or different the settings, context, methods, etc were.<br><br>Response 17<br>Additional information has been added to the studies cited in the discussion.<br><br>Lines 397, 404, 424, 436, 441, 458<br>Comment 18<br><br>there is an unacceptable high number of grammatical and language errors! This is a scientific journal and these issues must be addressed. For example, you cannot start a sentence with "Because" or say "In today's terms...."<br><br>Response 18<br>Incorrect grammar has been revised in the manuscript<br>Lines 89, 104, 144, 168, 216, 261, 264, 298, 358, 393, 406, 454, 457, 458, 484
**Additional Information:**	
**Question**	**Response**
**Financial Disclosure**<br><br>Enter a financial disclosure statement that describes the sources of funding for the work included in this submission. Review the submission guidelines for detailed requirements. View published research articles from *PLOS ONE* for specific examples.	Yes

This statement is required for submission and **will appear in the published article** if the submission is accepted. Please make sure it is accurate.<br><br>**Funded studies**<br>Enter a statement with the following details:<br>• Initials of the authors who received each award<br>• Grant numbers awarded to each author<br>• The full name of each funder<br>• URL of each funder website<br>• Did the sponsors or funders play any role in the study design, data collection and analysis, decision to publish, or preparation of the manuscript?<br><br>Did you receive funding for this work?	
Please add funding details.<br>as follow-up to "**Financial Disclosure**<br><br>Enter a financial disclosure statement that describes the sources of funding for the work included in this submission. Review the submission guidelines for detailed requirements. View published research articles from *PLOS ONE* for specific examples.<br><br>This statement is required for submission and **will appear in the published article** if the submission is accepted. Please make sure it is accurate.<br><br>**Funded studies**<br>Enter a statement with the following details:<br>• Initials of the authors who received each award<br>• Grant numbers awarded to each author<br>• The full name of each funder<br>• URL of each funder website<br>• Did the sponsors or funders play any role in the study design, data collection and analysis, decision to publish, or preparation of the manuscript?	ADG received the award<br><br>Bill and Melinda Gates Foundation, grant number OPP1034523<br><br>https://www.gatesfoundation.org/<br><br>The funders did not play a role in the study design, data collection and analysis, decision to publish, or preparation of the manuscript.

Did you receive funding for this work?"	
Please select the country of your main research funder (please select carefully as in some cases this is used in fee calculation).<br>as follow-up to "**Financial Disclosure**<br><br>Enter a financial disclosure statement that describes the sources of funding for the work included in this submission. Review the submission guidelines for detailed requirements. View published research articles from *PLOS ONE* for specific examples.<br><br>This statement is required for submission and **will appear in the published article** if the submission is accepted. Please make sure it is accurate.<br><br>**Funded studies**<br>Enter a statement with the following details:<br>• Initials of the authors who received each award<br>• Grant numbers awarded to each author<br>• The full name of each funder<br>• URL of each funder website<br>• Did the sponsors or funders play any role in the study design, data collection and analysis, decision to publish, or preparation of the manuscript?<br><br>Did you receive funding for this work?"	UNITED STATES - US
**Competing Interests**<br><br>Use the instructions below to enter a competing interest statement for this submission. On behalf of all authors, disclose any competing interests that could be perceived to bias this work—acknowledging all financial support and any other relevant financial or non-financial competing interests.<br><br>This statement is required for submission and **will appear in the published article** if	The authors have declared that no competing interests exist.

the submission is accepted. Please make sure it is accurate and that any funding sources listed in your Funding Information later in the submission form are also declared in your Financial Disclosure statement.<br><br>View published research articles from *PLOS ONE* for specific examples.<br><br>**NO authors have competing interests**<br><br>Enter: *The authors have declared that no competing interests exist.*<br><br>**Authors with competing interests**<br><br>Enter competing interest details beginning with this statement:<br><br>*I have read the journal's policy and the authors of this manuscript have the following competing interests: [insert competing interests here]*<br><br>* typeset	
**Ethics Statement**<br><br>Enter an ethics statement for this submission. This statement is required if the study involved:<br><br>• Human participants<br>• Human specimens or tissue<br>• Vertebrate animals or cephalopods<br>• Vertebrate embryos or tissues<br>• Field research<br><br>Write "N/A" if the submission does not require an ethics statement.<br><br>General guidance is provided below. Consult the submission guidelines for detailed instructions. **Make sure that all information entered here is included in the Methods section of the manuscript.**	Ethical considerations<br>The study protocol was approved by the University of the Witwatersrand Human Research Ethics Committee, Johannesburg (M 120343); the University of Cape Town, Cape Town Human Research Ethics Committee, South Africa (106/2012); and the London School of Hygiene & Tropical Medicine Research Ethics Committee UK (6165). All participants were informed about the study and gave written consent to participate and be recorded during the FGDs. Audio recordings, field notes, and transcripts were identified only by a unique study number. All study records were stored securely in locked filing cabinets where access to the records is restricted to specified study team members.

**Format for specific study types**<br><br>**Human Subject Research (involving human participants and/or tissue)**<br>• Give the name of the institutional review board or ethics committee that approved the study<br>• Include the approval number and/or a statement indicating approval of this research<br>• Indicate the form of consent obtained (written/oral) or the reason that consent was not obtained (e.g. the data were analyzed anonymously)<br><br>**Animal Research (involving vertebrate animals, embryos or tissues)**<br>• Provide the name of the Institutional Animal Care and Use Committee (IACUC) or other relevant ethics board that reviewed the study protocol, and indicate whether they approved this research or granted a formal waiver of ethical approval<br>• Include an approval number if one was obtained<br>• If the study involved *non-human primates*, add *additional details* about animal welfare and steps taken to ameliorate suffering<br>• If anesthesia, euthanasia, or any kind of animal sacrifice is part of the study, include briefly which substances and/or methods were applied<br><br>**Field Research**<br><br>Include the following details if this study involves the collection of plant, animal, or other materials from a natural setting:<br>• Field permit number<br>• Name of the institution or relevant body that granted permission	
**Data Availability**<br><br>Authors are required to make all data underlying the findings described fully available, without restriction, and from the time of publication. PLOS allows rare exceptions to address legal and ethical concerns. See the PLOS Data Policy and FAQ for detailed information.	Yes - all data are fully available without restriction

A Data Availability Statement describing where the data can be found is required at submission. Your answers to this question constitute the Data Availability Statement and **will be published in the article**, if accepted.<br><br>**Important:** Stating 'data available on request from the author' is not sufficient. If your data are only available upon request, select 'No' for the first question and explain your exceptional situation in the text box.<br><br>Do the authors confirm that all data underlying the findings described in their manuscript are fully available without restriction?	
**Describe where the data may be found in full sentences. If you are copying our sample text, replace any instances of XXX with the appropriate details.**<br><br>• If the data are **held or will be held in a public repository**, include URLs, accession numbers or DOIs. If this information will only be available after acceptance, indicate this by ticking the box below. For example: *All XXX files are available from the XXX database (accession number(s) XXX, XXX.*).<br>• If the data are all contained **within the manuscript and/or Supporting Information files**, enter the following: *All relevant data are within the manuscript and its Supporting Information files.*<br>• If neither of these applies but you are able to provide **details of access elsewhere**, with or without limitations, please do so. For example:<br><br>*Data cannot be shared publicly because of [XXX]. Data are available from the XXX Institutional Data Access / Ethics Committee (contact via XXX) for researchers who meet the criteria for access to confidential data.*<br><br>*The data underlying the results presented in the study are available from (include the name of the third party*	All relevant data are within the manuscript and its Supporting Information files. The data analyzed for this manuscript has been provided.

and contact information or URL).<br>• This text is appropriate if the data are owned by a third party and authors do not have permission to share the data.<br><br>* typeset	
Additional data availability information:	

Manuscript Click here to access/download;Manuscript;Manuscript.docx

"If we lose it, we are worried": Individual and provider level perceptions towards weight change among people living with HIV who undergo TB screening in routine health care settings in Gauteng Province, South Africa.

Authors

Tanyaradzwa Nicolette Dube[1], Fezeka Mboniswa[1], Samkelisiwe Ethel Qwana[2] Salome Charalambous[1,4], Nontobeko Ndlovu[1] Alison D. Grant [3,5], Yasmeen Hanifa[3] Johannes Machinya[6]

[1]The Aurum Institute, Johannesburg, South Africa; [2]Research Consulting Specialists CC Johannesburg, South Africa [3]TB centre, London School of Hygiene & Tropical Medicine, London, United Kingdom, [4]School of Public Health, Faculty of Health Sciences, University of the Witwatersrand, Johannesburg, South Africa, [5]Africa Health Research Institute, School of Laboratory Medicine and Medical Sciences, College of Health Sciences, University of KwaZulu-Natal, Durban, South Africa. [6] Department of Sociology, University of the Witwatersrand, Johannesburg, South Africa

Corresponding Author: Tanyaradzwa Dube, The Aurum Institute, Aurum House, The Ridge, 29 Queens Road, Parktown, Johannesburg, 2193, South Africa, Telephone: +27 (0)10 590 1600, Fax: . Email: tndube@auruminstitute.org

Abstract

Background

HIV weakens the immune system, increasing the risk of tuberculosis (TB) in people with living HIV (PLHIV). People living with HIV and on antiretroviral treatment (ART) often experience physical body size changes. Studies have found a significant discrepancy between PLHIV's self-reported weight loss and their measured weight loss when being screened for TB using the WHO tool. To understand this inconsistency, a qualitative sub-study was conducted to explore perceptions and attitudes towards weight change among adults attending HIV care, as well as health care workers in public clinics in Gauteng, South Africa.

Methods

Our qualitative study was nested within the XPHACTOR study. A total of seven focus group discussions were conducted, five with adult participants attending for HIV care and two with health care workers and research staff in clinics around Gauteng. Inductive thematic analysis was used to analyse the data.

Findings

The majority of PLHIV preferred to gain weight due to fear of stigma associated with weight loss. Weight loss is associated with HIV/AIDS, suggesting that people attending HIV care may underreport weight loss in the context of a TB symptoms screening tool because they fear stigma. Participants reported that weight changes impacted their daily lives and had psychological effects on them. Some PLHIV described lipodystrophy as disproportional weight gain. Culture and media have an influence on the perception of ideal body size and shape for both men and women.

Conclusions

Underreporting weight loss might result in poor sensitivity of the WHO TB screening tool and suggests that we need either alternative ways to determine weight loss or screening tools for TB that are less dependent on reported symptoms.

Keywords: HIV, TB screening**,** Weight change, ART, South Africa, WHO screening tool

Abstract word count 271/300

Manuscript word count 4981

Background

Tuberculosis (TB) is one of the most common opportunistic infections in people living with the human immunodeficiency virus (PLHIV) and a major contributor to morbidity and mortality [1,2]. The risk of developing active TB disease is estimated to be 16 (uncertainty interval 14–18) times greater in PLHIV than those without HIV infection [3]. Weight loss is a common symptom of TB, and the World Health Organization (WHO) recommends screening people living with HIV for TB using a tool that comprises questions on cough, fever, night sweats, and unintentional weight loss [4]. Unintentional weight loss is a cardinal symptom of TB and reporting of weight loss is a key entry point to testing for TB, so understanding perceptions and reporting of weight loss is important with regards to finding people with active TB.

People living with HIV and on antiretroviral treatment (ART) often experience physical body size changes such as weight change, and other body shape changes that can lead to stigmatization [5–10]. In South Africa, changes in body shape were particularly common among PLHIV taking stavudine, which was routinely used in ART between 2004 and 2010 but is now rarely used because of its side effect profile [11].

Two studies conducted in South Africa found a mismatch between PLHIV's self-reported and measured weight change: one was a quantitative study and the other conducted in-depth interviews focusing on adolescents only [12,13]. It is important to understand perceptions of PLHIV regarding weight and body shape changes because these may impact responses to the weight loss component of the WHO TB symptom screening tool, as well as influence adherence to ART and TB treatment. A better understanding of weight perceptions may help improve TB screening and guide

policies and recommendations for supporting PLHIV when they experience weight and body shape changes.

We conducted an in-depth qualitative study to understand weight perceptions among adults living with HIV as part of the XPHACTOR study. XPHACTOR was a cohort study investigating TB diagnostic pathways among adults attending routine HIV care in South Africa between 2012 and 2014 when standard ART regimens in South Africa included stavudine [14–16]. In XPHACTOR, PLHIV were screened for TB using the WHO screening tool and significant discrepancies were noted between participants' self-reported weight loss and their measured weight loss. To understand this inconsistency, a qualitative sub-study was conducted to explore perceptions and attitudes towards weight change among adults attending HIV care, as well as among health care workers (HCWs) in public clinics in Gauteng, South Africa. We sought to investigate whether attitudes to weight and body size might affect how people responded to the questions in the WHO TB symptom screening tool and whether people with stavudine-related lipodystrophy might describe the change in body shape as weight loss.

Methodology

Study design

This was an exploratory qualitative sub-study.

Study setting and population.

Focus group discussions (FGDs) were conducted at public clinics providing care for PLHIV in Gauteng. Participants were adults attending the clinics for routine HIV care at XPHACTOR study sites, HCWs, and XPHACTOR research staff.

Data collection

Seven FGDs with a semi-structured guide were conducted: five FGDs with purposively sampled adults attending for HIV care and two FGDs with HCWs and research staff. The FGDs with PLHIV included two groups with male-only participants, two groups with female-only participants, and one mixed group. For HCWs, there were two FGDs, one FGD was done with doctors and dieticians and the other with other HCWs and research staff. All the FGDs were conducted at the clinics where the study was being conducted and where PLHIV and health workers were attending or working. Adult PLHIV who were not XPHACTOR study participants were approached in-person for participation in the study via an ART support group linked to the clinic. Research Assistants and HCWs were contacted via email. The FGDs were conducted face-to-face by an experienced independent female research consultant (SEQ) with a Masters' degree and was assisted by a co-facilitator with a bachelor's degree. All participants completed the FGD. To ensure that important points were discussed, the moderators led the discussions using FGD guides for PLHIV (S1 annexure 1) and HCWs and research staff (S2 annexure 2). There was no pre-existing relationship between the moderators and participants. FGDs were conducted within the clinic setting where participants collect their medication.

The length of the FGDs ranged between 83 minutes and 202 minutes. All sessions were audio recorded. The moderators took some field notes. No one else was present in the FGDs besides the participants and researchers. We initially planned to conduct around three FGDs with about eight to twelve participants in each group but eventually conducted seven FGDs to reach saturation. The number of discussions increased because we separated male and female FGD's (two groups per gender) plus a mixed gender discussion to capture sufficient diversity in perspectives and experiences.

Sample size per group was determined by availability of participants and previous qualitative research which recommends six to twelve participants per FGD [17,18]. This range ensures sufficient diversity, a balanced interaction to allow all participants to contribute as well as effective facilitation of the discussion by the moderators. There were no repeat interviews done. All participants in the FGDs except those who were members of the research team were given ZAR 100=USD 15 to compensate for their time.

In addition to using a moderator guide, the interviewer probed on the questions and was able to gather more detailed responses from the participants. Participants were asked questions about their perceptions on ideal body weight, what informed these perceptions, personal experiences of, and attitudes towards body weight changes. In addition, they were asked questions about causes of weight change and reasons why weight changes measured at the clinic may differ from those reported by the patient. The HCWs and research staff were also asked questions about recent experiences of consultations with PLHIV around body weight. Images of body figures of different body shapes developed by Stunkard were used to explore perceptions about body size and body image. The figures ranged from one to nine where one is the thinnest body type and nine is the largest type [19]. There were separate figures for females and males. Participants were shown the images and were asked which body image represented them, which body image they preferred, and the most attractive body image.

Fig 1 The Stunkard scale of female body images shown to participants [19]

Fig 2 The Stunkard scale of male body images shown to participants [19].

Discussions were conducted in local languages IsiZulu, Sesotho, Setswana, and English. All discussions were tape-recorded to ensure that all the points were captured. All interviews were transcribed and interviews done in local languages were translated to English. Translations and transcriptions were done by experienced researchers fluent in the study languages. Transcriptions were done verbatim to capture nuances and deliberations including non-verbal cues such as short silences and laughs. Quality assurance was conducted whereby a second researcher checked the accuracy of the transcripts against audio recordings.

Ethical considerations

The study protocol was approved by the University of the Witwatersrand Human Research Ethics Committee, Johannesburg (M 120343); the University of Cape Town, Cape Town Human Research Ethics Committee, South Africa (106/2012); and the London School of Hygiene & Tropical Medicine Research Ethics Committee UK (6165). All participants were informed about the study and gave written consent to participate and be recorded during the FGDs. Audio recordings, field notes, and transcripts were identified only by a unique study number. All study records were stored securely in locked filing cabinets where access to the records was restricted to specified study team members.

Data analysis

A codebook was developed by two researchers (TD and FM) using inductive coding. Data were analysed manually using Excel. The two researchers independently inductively coded the transcripts and agreed on the emerging themes. Two data coders manually coded the data (TD and FM). TD has a Masters' degree and FM has

an Honours degree. The senior author is an experienced qualitative researcher with a doctoral degree and supervised the data analysis (JM). The data coders manually coded a sample of the transcripts and the senior author (JM) qualitatively and quantitatively checked intercoder reliability between the coders. Inductive thematic analysis was employed to develop themes. Both males and females were included to account for variation in perceptions by gender. We used an Excel spreadsheet to monitor the number of steps that were followed during the manual coding process. TD, FM and JM reviewed and refined emerging themes repeatedly during analysis until inductive thematic saturation was reached, where no new themes or categories emerged from the data [20]. Transcripts and findings were not returned to participants for comment. The COREQ (Consolidated criteria for REporting Qualitative research) checklist was used to check the content of this manuscript.

Results

Seven FGDs (labelled 1-7) were held between 6th and 21st August 2014, with 7-12 participants in each focus group. The five adult PLHIV FGDs (two males, two females and one mixed gender) had 7-12 participants in each group. The HCWs and research staff FGD comprised of ten participants (seven routine HCWs and three research staff). The doctors and dieticians FGD had seven participants (five doctors and two dieticians) who were all routine staff.

We identified three main themes and five sub themes. The main themes were: 1) perceptions and attitudes towards weight change; 2) ideal body size and shape; 3) negative impact of weight loss on participant's daily life. The sub themes were i) perceptions towards weight change; ii) attitudes towards weight loss; iii) stigma and

loss of social support; iv) psychological impact of weight loss and v) changes in dressing due to weight changes.

Main theme 1: Perceptions and attitudes towards weight change

Sub-theme (i): Perceptions towards weight change

Participants in all the FGDs for adult PLHIV attending for HIV care expressed high level of awareness of the possible physical body changes experienced by PLHIV and those on ART such as weight loss, weight gain and associated changes to body shape. The participants were aware of the main causes of weight loss, and they linked weight loss to poor diet, stress, active TB, HIV/TB treatment side effects, and non-adherence to HIV/TB treatment. It was reported that denial or failure to accept and embrace one's HIV positive status also resulted in weight loss.

> *I am saying, another thing that can make a person to lose weight is that because I am HIV positive, I must check whether I have TB or not because that also causes a person to lose weight (FGD 3, Females).*

Participants in the separate groups were asked how they knew that they were losing weight. Both male and female participants reported that they noticed that they had lost weight if their clothes were not fitting well.

HCWs and research staff indicated that weight loss could be caused by other health conditions among PLHIV. As such, when they noted weight loss in a PLHIV attending for HIV care, they reported that they would usually or ideally check the PLHIV's viral load, CD4 count, investigate for TB and other opportunistic infections, screen for cervical cancer, and test for diabetes.

Both PLHIV and HCWs identified ART side effects as one of the causes of weight gain and change in body shape among PLHIV:

> *Let me say, you can see that I am slender but if I take these tablets, you see and acquire a big stomach, they will then say he is in this condition, he is now taking these tablets, isn't these tablets are making him to have a big stomach* (FGD 1, Males).

Participants from the PLHIV's FGDs were happy to gain weight. They highlighted that weight gain was a sign that they were adhering to HIV treatment and that they were responding well to treatment:

> *… if we gain weight, I believe that we are happy, because it means that no, we are happy, we are eating well, we are doing things the right way and that our medication is working well but if we lose it, we are worried because it's like we are not taking these tablets* (FGD 5, Mixed).

PLHIV also perceived weight gain as a sign that one has accepted and embraced their HIV positive status:

> *It means that you are relaxed you are not obsessing over your HIV status, you see, you do not put yourself under stress, you think that I do have tablets. If I take tablets everything is okay* (FGD 1, Males).

Some PLHIV described lipodystrophy as disproportional weight gain. While PLHIV indicated that they would prefer weight gain to weight loss, they highlighted that sometimes when one gains weight, it might not be proportional to one's body size because they will gain weight on the upper body and lose weight on the lower part of the body, and this worried them:

You feel good when you gain, but what you do not like is that sometimes you can gain on the upper part and lose weight on the lower part. So that is what makes us sad (FGD 4 Females).

Sub-theme 2: Attitudes towards weight loss

There were both positive and negative attitudes towards weight loss. Some participants revealed that excessive weight gain was not healthy, so they adopted a healthy lifestyle including regular exercise and celebrated weight loss after gaining excessive weight, as shown in the quote below:

I was happy today because I had lost a lot of weight (FGD 3, Females).

The majority of the participants demonstrated negative attitudes toward weight loss and some associated weight loss with the side effects of ART. Such participants reported that they would consider stopping ART to gain weight:

For me sometimes, I get a feeling that maybe these ARVs do not get along with my system and then have another feeling that hey I will stop taking them since I do not see its results, I keep losing weight, that is why most of the time you find that after people have started ARVs.., you find that a person does not take them anymore and they will say, if I don't take them I regain my weight, (FGD 4, Females).

Participants further reported that there were some nurses who reproached them for delaying seeking health care when they visited the clinic with sickness and showing signs of weight loss:

You see at the clinic, the nurses scold at you if you visited them when you were really too sick and badly affected, [they] can see that you have lost weight, the

nurse scolds at you and say, you are this and that why did you not come and visit me (says P mimicking a female nurse) they scold at you there. (FGD, 1 Males).

The HCWs reported that they would become inquisitive about adult PLHIVs lifestyles if they showed signs of gaining weight as highlighted by a dietician in the HCWs group:

If they are gaining weight, we want to know if they are exercising or not (FGD 7, Dieticians and doctors).

In addition to weighing PLHIV on a scale, HCWs stated that they used alternative ways to identify if PLHIV had lost weight without openly using the words "weight loss" to get accurate answers and avoid being seen as stigmatizing PLHIV. The HCWs reported that they would instead ask participants if the size of their dress has changed or if family members had commented on changes in their body shape:

'*Has your dress size changed?' could be the alternative question* (FGD 7, Dieticians and doctors).

And then you can ask sometimes, if anyone, your friend, or family complained that your body shape has changed (FGD 7, Dieticians and doctors).

Main theme 2: Ideal body size and shape

The participants were aware that weight change also alters both body size and shape. Participants were shown both male and female Stunkard body image figures during FGDs to elicit their perceptions on ideal body size and shape [19]. The majority of male participants selected body image figure number five in the middle as the ideal figure for men. Some men suggested that women should be the same size as men and others reported that women should be slightly bigger because they bear children. Female participants selected body image figure number six as the ideal body type

because the image had a mid-sized proportional body. The choice of ideal body size was influenced by media for both women and men as indicated in the responses below:

> *I think influence in terms of being muscular has a lot to do with media. TV gives you the suggestion that someone who is muscular is more acceptable to the community (FGD 2, Males).*

> *they make jokes out of us, as women, they say check her out she has unnecessary fats on the waist area, you know it would have been better if I found someone like Beyoncé [female celebrity], you see. (FGD 4, Females).*

Participants further reported that culture also influenced how people perceive ideal body shape especially for women.

> *My sister is right about culture, Zulus prefer fat women. They say she is well-fed (FGD 3, Females).*

> *And I think we have a black culture that a black woman has to be round (Participants agreeing with one another), they will just be worried, when number 4, they won't even look at you (FGD 6, HCW and Research Staff).*

HCWs revealed they would ask PLHIV who had signs of lipodystrophy whether other people had commented on the changes in their body shape or size. According to the HCWs, PLHIV were more worried about changes in body shape or size and not so much about weight loss. It was uncommon for PLHIV to visit the clinic because they were worried about weight loss, but they usually visited the clinic when they noticed changes in body shape:

What I have noticed is that it is mostly PLHIV who have got lipodystrophy, they are the ones who are more worried about their weight that's one thing I have realised because they are saying, no, I no longer have my buttocks, they are gone, my legs are now thinner. (FGD 7, Dieticians and doctors).

HCWs stated that when they noticed changes in body shape and size among the PLHIV, they changed the regimen for those who were taking stavudine. PLHIV on the second-line ART regimen (at the time of the study, 2014, azidothymidine, lamivudine, lopinavir/ritonavir), were counselled and some were referred to government-funded plastic surgery services. HCWs also encouraged PLHIV to exercise and improve their diet.

FGDs with HCWs and research staff revealed that PLHIV who lost weight were referred to dieticians, social workers, psychologists, and religious leaders for support. PLHIV with socio-economic challenges were referred to the South African Social Security Agency for the Disability Grant. HCWs also revealed that they involved the families of PLHIV experiencing weight loss to explore potential causes.

Main theme 3: Negative impact of weight loss on participant's daily life

Sub-theme 1: Stigma and loss of social support

Participants reported that changes in physical body size, particularly weight loss, resulted in them being stigmatized in the community. Some of the participants reported that they experienced body shaming, name-calling, gossip, and discrimination because they had lost too much weight. Participants in the FGDs lamented that people in the communities would quickly conclude that a person has AIDS once one begins to lose weight. The participants reported that those who show signs of weight loss are

said to have bought OMO (washing powder) because the HIV “washed” way the person’s body the way OMO washes dirt:

They will say, so and so bought OMO can you actually see so and so, so in that manner they call you by those names when you are on the passing by, and you would think my goodness. They do this so that you cannot understand you see, you may not know why you are regarded as having bought OMO, when you look around no one bought OMO, they regard you as that since you lost weight (FGD 1, Males).

Participants with lipodystrophy were stigmatized and were called names such as “high heels” and KFC because of having thin legs and a fat upper body:

Thin legs. Do you know what a KFC drumstick looks like? When they see you, they say: It’s KFC! She went to buy KFC (FGD 3, Females).

Participants also reported stigmatizing attitudes from both community and family members which include being ignored and people keeping some distance from them:

Mostly, if they know that you are positive through appearance, the loss of weight, they start ignoring you and mostly the names like killer (FGD, 2 Males).

Even your family still does that thing, they say hey, don’t stand too close to me you will infect me with this thing (FGD 4 Females).

Some participants in the FGDs reported that PLHIV who show signs of weight changes may lose social support from their families and they attributed this to a lack of adequate knowledge on HIV:

So, this is what happened …, my mother is an elderly person so she does not know the difference between HIV and AIDS. When my brother took a cup and

> *drank from it, she would want nothing to do with that cup. So, I called her and tried to teach her about what HIV and AIDS are. I said I'm asking you to please support him* (FGD 3, Females).

Sub-theme 2: Psychological impact of weight loss

The participants in focus group discussions revealed that when some PLHIV discover that they have lost weight, they become so stressed:

> *The thing is, you just become stressed out, you just cannot concentrate on the things that you are doing at home, do you understand, you become short-tempered, you ask yourself what is going on, what did I do, what mistake did I make in order for me to lose weight in this manner* (FGD, 4 Female).

Some PLHIV revealed they feel anxious and stressed when they are asked to stand on the scale and be weighed at the clinic:

> *Before standing on the scale, I was afraid that they are about to weigh me now and it's possible that I may have lost weight* (FGD1, Males).

Sub-theme 3: Changes in dressing

When they notice that they have lost weight, participants reported that they would change the way they dress to cover signs of weight loss:

> *So, now that I have lost weight, when I get dressed and wear something that hides my bums they say why are you making yourself a granny? You must wear the latest fashion, and I say: hey I am not a child (FGD, 3 Female).*

Discussion

Participants demonstrated a strong awareness that some physical body changes such as weight gain and weight loss may be symptoms of HIV and AIDS. The participants further indicated that negative weight changes, i.e., weight loss, attract stigmatizing attitudes from society and that stigma remains a huge concern for PLHIV. As a result, the participants preferred gaining to losing weight. These findings are consistent with findings from other studies in South Africa that found that PLHIV would rather gain weight than be thin and have people assume that they are infected with HIV [5,12,21]. The results obtained in our study were similar to the findings of a qualitative Ugandan study that found that women living with HIV did not want to lose weight as this could lead to people suspecting that they were HIV positive. Women living with HIV who lost weight in the same study also reported that they felt stigmatized, and it affected their adherence to ART [21]. The finding that it was rare for PLHIV to seek healthcare services because of weight loss concerns may negatively affect efforts to screen for and diagnose TB early. Several studies have found an association between weight loss and delay in TB diagnosis [22–24]. A quantitative study consisting of 185 people with TB in KwaZulu Natal South Africa found that weight loss was associated with delayed care [22]. The researchers suggested that the stigma of weight loss because of its association with other diseases could have been a key factor contributing to this delay [22].

In our study, HCWs reported that because of the stigma attached to weight loss, they avoid asking PLHIV direct questions about weight loss because the fear of the stigma associated with weight loss may hinder PLHIV from telling the truth. The stigma associated with weight loss may make the WHO screening tool less sensitive, specifically the question on weight loss. A potential alternative to asking questions about weight loss when PLHIV present for treatment of check-ups is to measure their

weight at the visit and compare this to previous weight measures recorded in their file. The stigma associated with HIV and TB needs to be addressed for example by educating PLHIV, family members, and the community on HIV and TB. The education can be done through community outreach campaigns using tool kits that have been developed to address TB-related stigma [25–27].

Despite the preference to gain rather than lose weight amongst the majority of participants, some participants were aware that excessive weight gain has repercussions and reported adopting a healthy lifestyle including regular exercise and celebrated weight loss.

A mixed method study including 513 adult women was conducted in Cape Town, South Africa to explore the perception among black South African women that people who are thin are infected with HIV or have AIDS. The study found that participants preferred to be overweight and at risk of acquiring cardiovascular diseases, rather than to lose weight and be stigmatised as infected with HIV [5]. It is important to educate PLHIV that excessive weight gain may lead to obesity and increase the risk of non-communicable diseases. Therefore, PLHIV should be encouraged to exercise and eat well to reduce the risk of these diseases [8,13,28].

The psychological impact of weight changes leading to non-adherence found in our study has been reported in other studies [8,13,21,28]. Lipodystrophy is a concern among PLHIV. The finding that changes in body image affect PLHIV psychologically, with some considering stopping treatment, is consistent with other studies. A cross-sectional quantitative study on lipodystrophy among PLHIV in Tanzania found that changes in body shape may affect PLHIV psychosocial function, quality of life and may lead to PLHIV considering stopping treatment [28].

Our study found that fuller women were considered to have the ideal body shape. This finding is consistent with findings from other previous studies [5,13]. A qualitative study conducted among 18 adolescents in Durban using in-depth interviews revealed that female respondents desired to be thick and curvaceous, and males desired to be bigger and muscular, and this was linked to the influence of cultural norms on understanding of an ideal body shape [13]. Similarly, a study conducted in Cape Town found that cultural beliefs appeared to have a significant influence on the women interviewed, despite more than half of the participants having a high school education. Some women in this study reported that women are culturally expected to have a fuller figure [5].

Stavudine is now rarely used and so few PLHIV should newly develop lipodystrophy. Those with established lipodystrophy should have been switched to a different regimen which does not cause lipodystrophy, but these people may need support and reassurance to continue their current ART [11].

Participants in our study reported that changes in dress sizes made them realize they had lost weight. HCWs in our study recommended that asking participants about changes in clothing size was more useful than directly asking about unintentional weight loss. Asking clothing size is a self-report measure which can get biased responses, but we feel it is more acceptable to patients rather than directly asking about weight loss. A case cohort quantitative study in the Netherlands found that self-reported clothing size appeared to predict cancer risk independently of body mass index, suggesting that self-reported clothing size can be a useful measure to consider in epidemiologic studies [29].

Our study findings are significant as TB remains a leading cause of death among PLHIV so understanding their perceptions around weight change and how this may affect TB symptom screening is important. In addition, since this work, the new first-line treatment regimen for PLHIV includes dolutegravir, which has been found to have weight gain as a side effect. Hence, perceptions on weight gain highlighted in our paper remain relevant [21,30].

Strengths and limitations

One of the strengths of this study is that we conducted FGDs with both males and females and managed to get perceptions on weight change from both genders. However, the study was not without limitations. The sample size of the PLHIV who were interviewed was small and may not be representative of all PLHIV at the facilities. Again, PLHIV may not have been able to disclose sensitive personal information in focus groups. Despite these limitations, we managed to gain insights from PLHIV as well as HCWs on people's perceptions of weight change among people who live with HIV. The potential bias was minimised by the fact that the facilitator was an independent researcher, therefore would have been perceived to have adopted a neutral stance on the issues.

Conclusion

The majority of PLHIV preferred to gain weight due to fear of stigma associated with weight loss. Weight loss is associated with HIV /AIDS, suggesting that people attending for HIV care may underreport weight loss in the context of a TB symptom screening tool. This would result in reduced sensitivity of the WHO TB screening tool. Our findings suggest that we need either alternative ways to determine weight loss, or screening tools for TB which are less dependent on reported symptoms. Furthermore,

the policy implications of our paper suggest the importance of strengthening innovative TB testing strategies such as Targeted Universal Test and Treat (TUTT) which tests high risk groups including PLHIV irrespective of reporting TB symptoms or ART status [31].

Acknowledgments

The authors are grateful to the Department of Health nurses and staff at the facilities, the XPHACTOR research staff, and all the participants.

Author contributions

ADG, SC, and YH conceptualized the study. SEQ conducted the FGDs. TD and FM performed the data analysis with guidance from JM. NN was the project manager. TD drafted the paper, and all authors provided critical reviews, edited drafts, and approved the version to be published.

Reference List

1. Adamu AL, Gadanya MA, Abubakar IS, Jibo AM, Bello MM, Gajida AU, et al. High mortality among tuberculosis patients on treatment in Nigeria: A retrospective cohort study. BMC Infect Dis. 2017;17(1):1–12.
2. Tornheim JA, Dooley KE. Tuberculosis Associated with HIV Infection. American Society for Microbiology. 2017;5(1):1–16.
3. World Health Organization. Tuberculosis Key Facts [Internet]. 2023 [cited 2023 Jul 5]. Available from: https://www.who.int/news-room/fact-sheets/detail/tuberculosis
4. WHO. WHO Consolidated Guidelines on Tuberculosis. Module 2 Screening [Internet]. Who. 2021 [cited 2024 Sep 10]. Available from: https://iris.who.int/bitstream/handle/10665/340255/9789240022676-eng.pdf?sequence=1
5. Matoti-Mvalo T, Puoane T. Perceptions of body size and its association with HIV/AIDS. South African Journal of Clinical Nutrition. 2011;24(1):40–5.
6. Li N, Spiegelman D, Drain P, Mwiru RS, Mugusi F, Chalamilla G, et al. Predictors of weight loss after HAART initiation among HIV-infected adults in Tanzania. Aids. 2011;26(5):577–85.
7. Tate T, Willig AL, Willig JH, Raper JL, Moneyham L, Kempf MC, et al. HIV infection and obesity: Where did all the wasting go? Antivir Ther. 2012;17(7):1281–9.
8. Power R, Tate HL, McGill SM, Taylor C. A qualitative study of the psychosocial implications of lipodystrophy syndrome on HIV positive individuals. Sex Transm Infect. 2003;79(2):137–41.

9. Lakey W, Yang LY, Yancy W, Chow SC, Hicks C. Short Communication: From wasting to obesity: Initial antiretroviral therapy and weight gain in HIV-infected persons. AIDS Res Hum Retroviruses. 2013;29(3):435–40.

10. Sax PE, Erlandson KM, Lake JE, McComsey GA, Orkin C, Esser S, et al. Weight gain following initiation of antiretroviral therapy: Risk factors in randomized comparative clinical trials. Clinical Infectious Diseases. 2020;71(6):1379–89.

11. The South African Antiretroviral Treatment Guidelines 2010 [Internet]. 2010 [cited 2024 Apr 19]. Available from: https://sahivsoc.org/Files/Summary_The_South_African_Antiretroviral_Treatment_2010.pdf

12. Hurley E, Coutsoudis A, Giddy J, Knight SE, Loots E, Esterhuizen TM. Weight evolution and perceptions of adults living with HIV following initiation of antiretroviral therapy in a South African urban setting. South African Medical Journal. 2011;101(9):645–50.

13. Nyamaruze P, Govender K. € I like the way i am, but i feel like i could get a little bit bigger": Perceptions of body image among adolescents and youth living with HIV in Durban, South Africa. PLoS One [Internet]. 2020;15(1):1–10. Available from: http://dx.doi.org/10.1371/journal.pone.0227583

14. Hanifa Y, Fielding KL, Chihota VN, Adonis L, Charalambous S, Foster N, et al. A clinical scoring system to prioritise investigation for tuberculosis among adults attending HIV clinics in South Africa. PLoS One. 2017 Aug 1;12(8).

15. Pillai N, Foster N, Hanifa Y, Ndlovu N, Fielding K, Churchyard G, et al. Patient costs incurred by people living with HIV/AIDS prior to ART initiation in primary healthcare facilities in Gauteng, South Africa. PLoS One. 2019 Feb 1;14(2).

16. Auld AF, Kerkhoff AD, Hanifa Y, Wood R, Charalambous S, Liu Y, et al. Derivation and external validation of a risk score for predicting HIV-associated tuberculosis to support case finding and preventive therapy scale-up: A cohort study. PLoS Med. 2021 Sep 1;18(9).

17. Li Ping W, P WL. Focus group discussion: a tool for health and medical research. Singapore Med J. 2008;49(3):256–61.

18. Dilshad RM, Latif MI. Focus Group Interview as a Tool for Qualitative Research: An Analysis. Vol. 33, Pakistan Journal of Social Sciences (PJSS). 2013.

19. Sorensen TIA, Stunkard AJ. Does obesity run in families because of genes? An adoption study using silhouettes as a measure of obesity. Acta Psychiatr Scand. 1993;67–72.

20. Saunders B, Sim J, Kingstone T, Baker S, Waterfield J, Bartlam B, et al. Saturation in qualitative research: exploring its conceptualization and operationalization. Qual Quant. 2018 Jul 1;52(4):1893–907.

21. Alhassan Y, Twimukye A, Malaba T, Myer L, Waitt C, Lamorde M, et al. "It's only fatness, it doesn't kill": a qualitative study on perceptions of weight gain from use of dolutegravir-based regimens in women living with HIV in Uganda. BMC Womens Health. 2022 Dec 1;22(1).

22. Chiposi L, Cele LP, Mokgatle M. Prevalence of delay in seeking tuberculosis care and the health care seeking behaviour profile of tuberculous patients in a rural district of Kwazulu Natal, South Africa. Pan African Medical Journal. 2021 May 1;39.

23. Bojovic O, Medenica M, Zivkovic D, Rakocevic B, Trajkovic G, Kisic-Tepavcevic D, et al. Factors associated with patient and health system delays in diagnosis and treatment of tuberculosis in Montenegro, 2015–2016. PLoS One. 2018 Mar 1;13(3).

24. Belkina T V., Khojiev DS, Tillyashaykhov MN, Tigay ZN, Kudenov MU, Tebbens JD, et al. Delay in the diagnosis and treatment of pulmonary tuberculosis in Uzbekistan: A cross-sectional study. BMC Infect Dis. 2014;14(1).

25. Kidd Ross, International HIV/AIDS Alliance., ZAMBART Urban Tuberculosis Project. Understanding and challenging TB stigma toolkit for action : introduction to TB stigma, more understanding and less fear about TB [Internet]. International HIV/AIDS Alliance; 2009 [cited 2024 Sep 10]. 68 p. Available from: https://assets.publishing.service.gov.uk/media/57a08b3ced915d622c000bb7/TB_and_Stigma_May09.pdf

26. AIDS Foundation of South Africa. Reducing Stigma related to Gender, Culture and HIV/AIDS [Internet]. 2017 [cited 2024 Sep 10]. Available from: https://www.aids.org.za/wp-content/uploads/2018/05/Facilitator%E2%80%99s-Guide-Stigma-Related-to-Gender-Culture-HIV.pdf

27. Nyblade L, Stangl A, Weiss E, Ashburn K. Combating HIV stigma in health care settings: What works? Vol. 12, Journal of the International AIDS Society. John Wiley and Sons Inc.; 2009.

28. Njelekela M, Mpembeni R, Muhihi A, Ulenga N, Aris E, Kakoko D. Lipodystrophy among HIV-Infected Patients Attending Care and Treatment Clinics in Dar es Salaam. 2017;2017(October 2004).

29. Hughes LAE, Schouten LJ, Goldbohm RA, Van Den Brandt PA, Weijenberg MP. Self-reported clothing size as a proxy measure for body size. Epidemiology. 2009 Sep;20(5):673–6.

30. Brennan AT, Nattey C, Kileel EM, Rosen S, Maskew M, Stokes AC, et al. Change in body weight and risk of hypertension after switching from efavirenz to dolutegravir in adults living with HIV: evidence from routine care in Johannesburg, South Africa. EClinicalMedicine. 2023 Mar 1;57.

31. Martinson NA, Nonyane BAS, Genade LP, Berhanu RH, Naidoo P, Brey Z, et al. Evaluating systematic targeted universal testing for tuberculosis in primary care clinics of South Africa: A cluster-randomized trial (The TUTT Trial). PLoS Med. 2023 May 1;20(5 May).

Figure

Click here to access/download;Figure;Fig 1 The Stunkard scale of female body images.tif

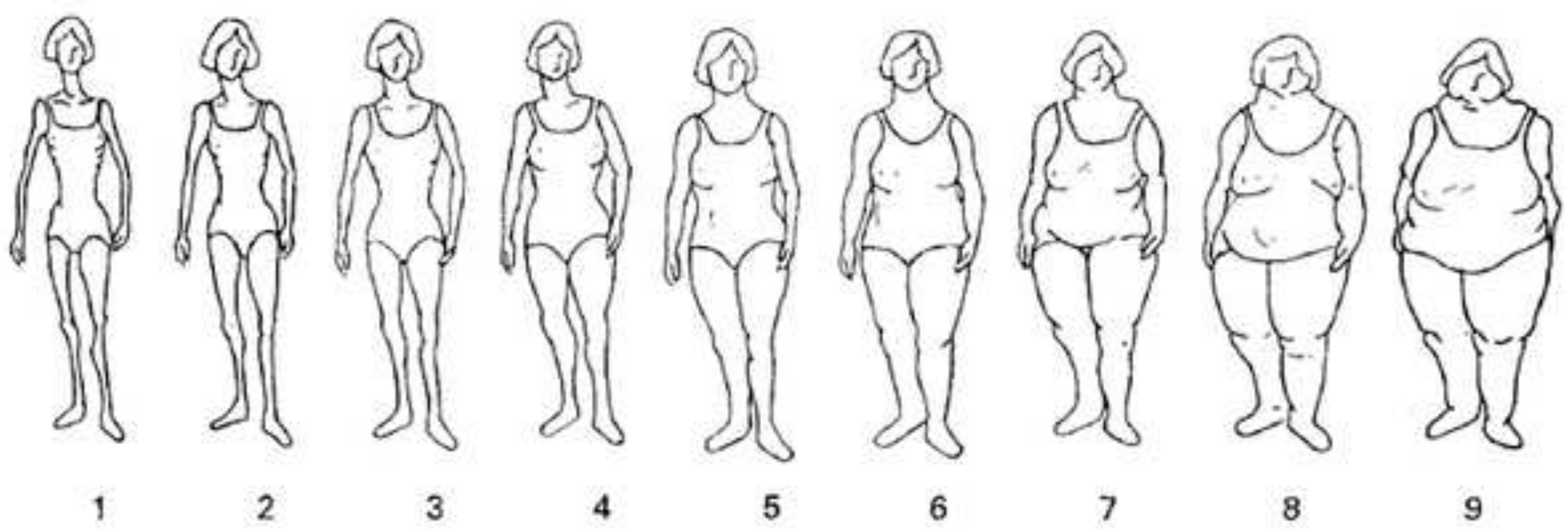

Figure

Click here to access/download;Figure;Fig 2 Figure 2 The Stunkard scale of male body images.tif

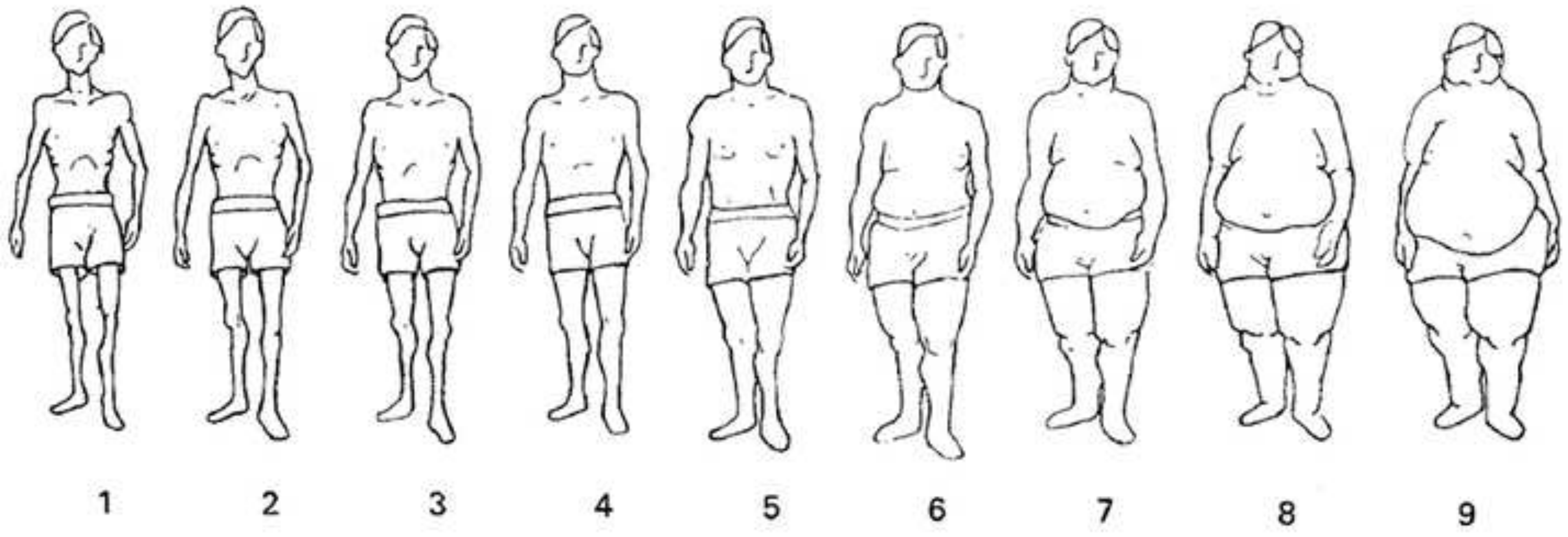

Click here to access/download
Supporting Information
S3 CoreQ _Checklist_November.pdf

Click here to access/download
Supporting Information
S5 Ethics Approval MHREC Approval version 5.1.pdf

Click here to access/download
Supporting Information
S6 Ethics
Aproval_Grant_A487_6165_approval_06012014.pdf

Click here to access/download
Supporting Information - Compressed/ZIP File Archive
S4 Transcripts_21 July.zip

Your MS Word document "Manuscript_revised with tracked changes_9 July.docx" cannot be opened or processed. Please see the list of common problems and suggested resolutions below.

Common Problems When Creating a PDF from Microsoft Word Documents

When you open your document in MS Word, an alert may appear. This message may relate to margins or document size. You will need to find the piece of your Word document that is causing the problem. Selectively remove various pieces of the file, saving the modified file with a temporary file name. Then try to open the modified file. Repeat this process until the alert no longer appears when you open the document.

Embedded Macros

Your submission file should not contain macros. If it does, an alert may appear when you open your document (this alert prevents EM from automatically converting your Word document into the PDF that Editors and Reviewers will use). You must remove these macros from your Word document.

Read-Only and Password-Protected Files

EM cannot process read-only or password-protected submission files. If your file is read-only or password-protected and you receive an error, please disable the document protection, save, and re-submit the file.

Corrupted Tables

Your document may contain a table that cannot be rendered correctly. This will be indicated by an alert. Correct the content of the table causing the problem so that the alert no longer appears.

Older MS Word files

EM supports files in MS Word 2000 and older versions. If you are using a more recent version of MS Word, try saving your Word document in the more recent format and resubmit to EM.

Other Problems

If you can get your Word document to open with no alert messages appearing and you have submitted it in a current MS Word format, and you still see an error message in your PDF file (where the Word document should be appearing), please contact the publication via the 'Contact Us' link on the EM Navigation Bar.' You will need to reformat your Word document and then re-submit it.

PLOS ONE

1265 Battery Street, Suite 200

San Francisco, CA 94111

United States

9 July 2025

Dear Editors in chief

We would like to thank the editor and reviewers for the very valuable comments made. They have certainly enabled us to strengthen the quality of our paper and increase its relevance. In the present letter, we have a table showing the comments raised and our responses. Both clean and marked-up copies of the revised manuscript are attached.

Regards,

Tanyaradzwa Dube, corresponding author.

Desk Review Comments

Comment	Response
1. We note that this data set consists of interview transcripts. Can you please confirm that all participants gave consent for interview transcript to be published?<br><br>If they DID provide consent for these transcripts to be published, please also confirm that the transcripts do not contain any potentially identifying information (or let us know if the participants consented to having their personal details published and made publicly available). We consider the following details to be identifying information:<br><br>- Names, nicknames, and initials<br>- Age more specific than round numbers	Yes, the participants consented to having their de identified data shared in published journals.<br><br>The transcripts have been checked to remove any identifying information as recommended.

- GPS coordinates, physical addresses, IP addresses, email addresses<br>- Information in small sample sizes (e.g. 40 students from X class in X year at X university)<br>- Specific dates (e.g. visit dates, interview dates)<br>- ID numbers	

Responses to the Editors Comments

Responses Reviewer 2

Comment	Response
Line 1<br><br>"*If we lose it, we are worried"*: Individual and provider level perceptions and attitudes towards weight change among people living with HIV screening for tuberculosis in routine health care settings in Gauteng Province, South Africa.<br><br>please read this title again. The grammar seems incorrect...please check!<br>perhaps consider : perceptions and attitudes towards weight change among people living with HIV who undergo TB screening	Line 1, page 1<br><br>Agreed changed to<br><br>Individual and provider level perceptions towards weight change among people living with HIV who undergo TB screening in routine health care settings in Gauteng Province, South Africa.
Line 104 page 5<br>We were interested to know whether attitudes to weight and body size might affect how people responded to the questions in the WHO TB symptom screening tool and whether people with stavudine-related lipodystrophy might describe the change in body shape as weight loss.<br><br>Comment: This sentence should read "We sought to investigate whether…….	Line 103 page 5<br><br>Agreed and changed to: We sought to investigate whether attitudes to weight and body size might affect how people responded to the questions in the WHO TB symptom screening tool and whether people with stavudine-related lipodystrophy might describe the change in body shape as weight loss.
Line 119, page 6	Line 123-125 page 6

Tell us something more about the Focus groups...you say they were all male, all female and mixed. did you have a target group size? How many came from the XPHACTOR study and how many were invited from the ART support group? similarly, how many staff were art of the initial group and how many were invited? how did you determine sample size per group?	Adult PLHIV who were not XPHACTOR study participants were approached in-person for participation in the study via an ART support group linked to the clinic.<br><br>Lines 134 to 142<br><br>We initially planned to conduct around three FGDs with about eight to twelve participants in each group but eventually conducted seven FGDs to reach saturation. The number of discussions increased because we separated male and female FGD’s (two groups per gender) plus a mixed gender discussion to capture sufficient diversity in perspectives and experiences. Sample size per group was determined by availability of participants and previous qualitative research which recommends six to twelve participants per FGD(17,18). This range ensures sufficient diversity, a balanced interaction to allow all participants to contribute as well as effective facilitation of the discussion by the moderator.<br><br>Line 200-205 page 9<br><br>Seven FGDs (labelled 1-7) were held between 6th and 21st August 2014, with 7-12 participants in each focus group. The five adult PLHIV FGDs (two males, two females and one mixed gender) had 7-12 participants in each group. The HCWs and research staff FGD comprised of ten participants (seven routine HCWs and three research staff). The doctors and dieticians FGD had seven participants (five doctors and two dieticians) who were all routine staff.
Line 124, page 6<br><br>Adult PLHIV who were not XPHACTOR study participants were approached for participation in the study face to face via an ART support group linked to the clinic.<br><br>Comment: sentence should read as<br><br>"Adult PLHIV who were not XPHACTOR study participants were approached in-person for	Line 123 page 7<br><br>Sentence changed as suggested.<br><br>Adult PLHIV who were not XPHACTOR study participants were approached in-person for participation in the study via an ART support group linked to the clinic

participation in the study via an ART support group linked to the clinic."	
Line 135, page 6<br><br>We initially planned to conduct around three FGDs but eventually conducted seven FGDs to reach saturation<br><br>Comment:<br>could you expand on this ....this related to the methodological approach taken:	Line 136-142 page 7<br><br>The number of discussions increased because we separated male and female FGD's (two groups per gender) plus a mixed gender discussion to capture sufficient diversity in perspectives and experiences. Sample size per group was determined by availability of participants and previous qualitative research which recommends six to twelve participants per FGD. This range ensures sufficient diversity, a balanced interaction to allow all participants to contribute as well as effective facilitation of the discussion by the moderator.
Line 161, page 8<br><br>All interviews were transcribed verbatim, and interviews done in local languages were translated to English<br><br>tell us more on this process and language translation is a complex process. one has to make sure that the nuances are not lost during the translation process!!	Line 161-171 page 8<br><br>More details have been added:<br><br>Translations and transcriptions were done by experienced researchers fluent in the study languages. Transcriptions were done verbatim to keep nuances for instance, almost all deliberations were captured including non-verbal cues such as short silences and laughs. Quality assurance was conducted whereby a second researcher checked the accuracy of the transcripts against audio recordings.
Line 186, Page 9<br>could you add a few lines on the process to reach saturation?	Line 193-196 page 9<br><br>TD, FM and JM reviewed and refined emerging themes repeatedly until saturation was achieved when no additional themes or categories could be identified.
Line 189, page 9<br><br>so there were main themes and sub-themes! the main themes identified are not what you are reporting in this sentence...they are sub-themes...separate them and be clear about the main and sub-themes!	Line 206-211 page 10<br><br>The main themes and sub themes were separated as following;<br><br>We identified three main themes and five sub themes. The main themes were i. perceptions and attitudes towards weight change; ii ideal body size and shape;

	iii negative impact of weight loss on participant's daily life. The sub themes were i) perceptions towards weight change; ii) attitudes towards weight loss; iii) stigma and loss of social support; psychological impact of weight loss; and iv) changes in dressing.
Line 375, page 17<br><br>please correct the grammar...you cannot start a sentence with "because"	Line 393, page 18<br><br>Changed sentence to "As a result, the participants preferred gaining to losing weight".
Line 378 page 17<br><br>Corroborating our finding, a study in Uganda found that women living with HIV did not want to lose weight because weight loss could lead to people suspecting their HIV seropositive status. Women living with HIV who lost weight in the same study also reported that they felt stigmatized, and it affected their adherence to ART[18]<br><br>This should read as "The results obtained in our study were similar to the findings of a Ugandan study that found that women living with HIV did not want to lose weight as this could lead to people suspecting that they were HIV positive.	Line 397-401, page 18<br><br>Agreed and the sentence changed as suggested.<br><br>The results obtained in our study were similar to the findings of a Ugandan study that found that women living with HIV did not want to lose weight as this could lead to people suspecting that they were HIV positive
Line 381 page 17<br><br>The finding that it was rare for PLHIV to seek healthcare services because of weight loss concerns may negatively affect efforts to screen for and diagnose TB early.<br><br>did your study find this or was this from the literature?	Line 401-404 page 18<br>This was a potential implication based on previous studies that have shown an association between loss of weight and delayed TB diagnosis/ seeking care.<br><br>The finding that it was rare for PLHIV to seek healthcare services because of weight loss concerns may negatively affect efforts to screen for and diagnose TB early. Several studies have found an association between weight loss and delay in TB diagnosis(21–23). A study consisting of 185 people with TB in KwaZulu Natal South Africa found that weight loss was associated with delayed care(21). The researchers suggested that stigma of weight loss because of its association with other diseases could have been a key factor contributing to this delay (21).

Line 388, page 17<br><br>A potential alternative to asking questions about weight loss when PLHIV present for treatment of check-ups is to measure their weight at the visit and compare this to previous weight measures recorded in their file.	Line 413-415 page 19<br><br>Agreed and changed as suggested.
Line 389, page 17<br><br>The stigma associated with HIV and TB needs to be addressed for example by educating, through community outreach campaigns, both PLHIV, family members, and the community on HIV and TB using tool kits that have been developed to address TB-related stigma.<br><br>please correct the grammar ..this sentence does not make sense!	Line 426-428 page 9<br><br>Agreed sentenced revised to:<br><br>The stigma associated with HIV and TB needs to be addressed for example by educating PLHIV, family members, and the community on HIV and TB. The education can be done through community outreach campaigns using tool kits that have been developed to address TB-related stigma
Line 397, page 18<br><br>A study conducted in Cape Town, South Africa found that participants preferred to be overweight and at risk of acquiring cardiovascular diseases, rather than to lose weight and be stigmatised as infected with HIV.<br><br>for better context, perhaps add a few lines about this study ...how many participants, what was the objective of this study, methodology used, etc.	Line 424-440 page 19<br><br>Done, additional was added.<br><br>A mixed method study including 513 adult women was conducted in Cape Town, South Africa to explore the perception among black South African women that people who are thin are infected with HIV or have AIDS. The study found that participants preferred to be overweight and at risk of acquiring cardiovascular diseases, rather than to lose weight and be stigmatised as infected with HIV[5]
Line 410, page 18<br><br>Our study found that fuller women were considered to have the ideal body shape. This finding is consistent with findings from other previous studies.<br><br>Comment<br>Add references:	References cited. Line 440<br><br>Matoti-Mvalo T, Puoane T. Perceptions of body size and its association with HIV/AIDS. South African Journal of Clinical Nutrition. 2011;24(1):40–5.<br><br>Nyamaruze P, Govender K. I like the way i am, but i feel like i could get a little bit bigger": Perceptions of body image among adolescents and youth living with HIV in Durban, South Africa. PLoS One [Internet].

	2020;15(1):1–10. Available from: http://dx.doi.org/10.1371/journal.pone.0227583
Line 431, page 19<br><br>Our study findings are significant in today's terms as TB remains a leading cause of death among PLHIV<br><br>Delete "in today's terms" in the sentence	Deleted as suggested. Line 462<br><br>Our study findings are significant as TB remains a leading cause of death among PLHIV so understanding their perceptions around weight change and how this may affect TB symptom screening is important
The other concern I have is the discussion section where you compare or quote other studies ..please provide a bit more context to those studies so the reader has a sense of how similar or different the settings, context, methods, etc were.	Additional information has been added to the studies cited in the discussion.<br><br>Lines 397, 404, 424, 436, 441, 458
there is an unacceptable high number of grammatical and language errors! This is a scientific journal and these issues must be addressed. For example, you cannot start a sentence with "Because" or say "In today's terms...."	Incorrect grammar has been revised in the manuscript Lines 89, 104, 144, 168, 216, 261, 264, 298, 358, 393, 406, 454, 457, 458, 484
