# Supplementary material for: “If we lose it, we are worried”: Individual and provider level perceptions towards weight change among people living with HIV who undergo TB screening in routine health care settings in Gauteng Province, South Africa
Source: PLoS One. 2025 Sep 22;20(9):e0331904. doi: 10.1371/journal.pone.0331904 (PMC12453174; doi:10.1371/journal.pone.0331904)
Supplement: Attachment — Submitted filename: Responses to reviewers 22Nov.docx [file pone.0331904.s006.docx]

PLOS ONE
1265 Battery Street, Suite 200
San Francisco, CA 94111
United States

16 April 2024

Dear Editors in chief

We would like to thank the editor and reviewers for the very valuable comments made. They have certainly enabled us to strengthen the quality of our paper and increase its relevance. In the present letter, we have a table showing the comments raised and our responses. Both clean and marked-up copies of the revised manuscript are attached.

Regards,

Tanyaradzwa Dube, corresponding author.

**Responses to the Editors Comments**

| 1. Please ensure that your manuscript meets PLOS ONE's style requirements, including those for file naming. | Agreed. This has been done. |
| --- | --- |
| 2. In the online submission form you indicate that your data is not available for proprietary reasons and have provided a contact point for accessing this data. Please note that your current contact point is a co-author on this manuscript. According to our Data Policy, the contact point must not be an author on the manuscript and must be an institutional contact, ideally not an individual. Please revise your data statement to a non-author institutional point of contact, such as a data access or ethics committee, and send this to us via return email. Please also include contact information for the third party organization, and please include the full citation of where the data can be found. | Agreed. Data has been submitted with the revised manuscript. |
| 3. Please upload a copy of Figure 4 and 5, to which you refer in your text on page 12 and 18. If the figure is no longer to be included as part of the submission please remove all reference to it within the text. | Figures with Stunkard body image figures have been added to the manuscript (Fig1 and Fig 2). The figures have male and female body image figures ranging from 1 to 9 and participants were choosing the image they preferred. |
| 4. Please include captions for your Supporting Information files at the end of your manuscript, and update any in-text citations to match accordingly. Please see our Supporting Information guidelines for more information: <http://journals.plos.org/plosone/s/supporting-information>. | Done |
| 5. The work submitted is noted. Could the authors address the constructive Reviewer comments?   Discuss the significance of the study findings in today's terms and add the relevant practical and policy implications  the data is old so please justify, especially noting the chronicity of HIV in South Africa | Our study findings are significant in today’s terms as South Africa still has a high estimated HIV prevalence rate of 17.8 among adults (15-49 years) and 75% of PLHIV are on ART(23). TB remains a leading cause of death among PLHIV so understanding their perceptions around weight change and how this may affect TB symptom screening is important. In addition, since this work, the new first-line treatment regimen for PLHIV includes dolutegravir, which has been found to have weight gain as a side effect. Hence, perceptions on weight gain highlighted in our paper remain relevant (18,24).  Page 20 line 431-436  The policy implications of our paper suggest that in addition to TB screening, we should continue using innovative TB testing strategies such as Targeted Universal Test and Treat (TUTT) which tests high risk groups including PLHIV irrespective of reporting TB symptoms so that PLHIV with TB are not missed due to inaccurate self-reporting on TB symptoms.  See line 458 page 20 |

**Responses to Reviewer 1**

| Comment | Response |
| --- | --- |
| 1. The aspect of weight loss in relation to the tool used for screening tuberculosis seems to appear only at the background. Considering the relationship between HIV/AIDS and TB, as well as the high chances of people living with HIV/AIDS to contract tuberculosis due to the compromise of their immune system, it would be nice to have it appear up front even from the title level. This will certainly give a sense of concern to the people living with HIV/AIDS and the "why we should worry" about their perceptions and attitudes on any weight change particularly the weight loss. | Agreed. The title has been revised to include TB screening among PLHIV.    “*If we lose it, we are worried”*: Individual and provider level perceptions and attitudes towards weight change among people living with HIV screening for tuberculosis in routine health care settings in Gauteng Province, South Africa.  Also the risk of developing TB among people living with HIV was included on page 4 (line72-73) in the background |
| 2. Lastly, on data availability and restrictions, if the author indeed de-identified the transcripts with a unique study number, then there should be no fears of bridging confidentiality if made public. | Agreed. Data has been submitted with the revised manuscripts. |

**Responses to Reviewer 2**

| Comment | Response |
| --- | --- |
| 1. this is misleading. your population comes from one part of the country (Gauteng) ..so your tiltle should reflect this. Are these findings generalizable ..then i wont have a problem! | Agreed. The title has been changed to reflect Gauteng Province  “*If we lose it, we are worried”*: Individual and provider level perceptions and attitudes towards weight change among people living with HIV screening for tuberculosis in routine health care settings in Gauteng Province, South Africa. |
| 2. Line 125  Research Assistants and HCWs were conducted via email  do you mean contacted? If not, then this sentence does not make sense!! | Yes. We meant contacted. This has been edited on page 6 line 126 |
| 3. Quote on line 236:  *When I thought about the scale I was so excited because I have been weighing 152, so I had to hit the gym whatever it took. So today when I thought about coming to the scale I was excited. I wanted to see if this 152 had gone down. ((They all laugh)). When I got there I found out that it had gone down it’s 62.5. and I was happy* (FGD 3, Females).  Reviewer’s comment: not sure if this is kgs ..also the drop in weight then is significant! | Yes, the participant meant kg’s. We agree that the weight loss reported her was massive and we quoted a participant who may have gotten the figures wrong. We have decided to use a different quote to support the finding that other participants had a positive attitude towards weight loss on page 12 line 244  *I was happy today because I had lost a lot of weight (FGD 3, Females).* |
| 4. This sentence contradicts what you have been saying all along that PLHIV under-report weight loss but here you are saying that they report weight loss even though they have not lost weight!!! | We agree with the reviewer that the data primarily support the idea that PLHIV are likely to underreport weight loss and have removed the two sentences in the discussion that talk about overreporting. |
| 5. So maybe somewhere in your manuscript you need to highlight the probability of TB diagnosis in PLHIV.  also is weight loss a function of the drug or a symptom of TB in these patients or both? | The risk of developing active TB disease is estimated to be 16 (uncertainty interval 14–18) times greater in PLHIV than those without HIV infection. (WHO, 2023). The statement has been added to the background line 72-73.  Line 198-204 highlight participant’s perceptions on possible causes of weight loss which includes side effects of medication and TB disease. Line 246 refers to the idea that ART might cause weight loss. A quote has been added to support the statement regarding TB causing weight loss.  *“I am saying, another thing that can make a person to lose weight is that because I am HIV positive, I must check whether I have TB or not because that also causes a person to lose weight” (FGD 3, Females).* |
| 6. Line 386 Statement: A potential response could be to weigh PLHIV instead of asking them the question on weight loss  Comment: Surely this applies to anyone from this community regardless of their HIV status? | Agreed. This has been changed to: A potential response could be to weigh people seeking health care services instead of asking them the question on weight loss. (Lines 388-389) page 18 |
| 7. Line 396-398   Statement: It is important to educate PLHIV that excessive weight gain may lead to obesity and increase the risk of non-communicable diseases  Comment: surely here a better measure would be BMI. the researchers had the opportunity to weigh the participants. i am not sure if the height was taken. if so a BMI could have been calculated and then the quest of excessive weight gain would have been easy to recognize in the context of a BMI score | Yes BMI was calculated as part of the study procedure. In our paper, we recommend educating PLHIV about the negative effects of weight gain which they perceived to be favorable from the qualitative findings. Page 18 Line 400 |
| 8. Line 400  Statement: The psychological impact of weight changes leading to non-adherence found in our study has been reported in other studies[7,12,20].  Comment: where is this reported and non-adherence to what? | It was reported on page 12 (lines 245-253). Some participants reported that they would consider stopping treatment if they kept losing weight. We have clarified the treatment they would consider stopping which is antiretroviral treatment (line 247). |
| 9. Line 408 It is important to show the outcomes of similar studies in different settings or cultures and comment on these in the context of your findings. | The discussion section (pages 18-19) refers to studies conducted in other parts of South Africa like Cape Town (lines 415-422) and Durban (lines 411-414) as well as studies on weight perceptions conducted in other countries such as Tanzania (lines 406-409) and Uganda (lines 378-481). |
| 10. How confident are the researchers that asking about a change of dress size can be used as proxy for weight loss or gain. what if they simply dont answer truthfully to this question? Are there studies that have shown dress size to be a reliable proxy for weight gain or loss? Studies | Since this was a qualitative study, we asked about perceptions of weight loss. Participants in our study reported that changes in dress sizes made them realize they lost weight. HCWs in our study recommended that asking participants changes in clothing size could be better than directly asking unintentional weight loss questions. Asking clothing size is a self-report measure which can get biased responses, but we feel is more acceptable to patients rather than directly asking about weight loss. A study in Netherlands found that self-reported clothing size appeared to predict cancer risk independently of body mass index, suggesting that self-reported clothing size can be a useful measure to consider in epidemiologic studies.  Hughes et al (2009) *Self-reported clothing size as a proxy measure for body size* |
