# Supplementary material for: “If we lose it, we are worried”: Individual and provider level perceptions towards weight change among people living with HIV who undergo TB screening in routine health care settings in Gauteng Province, South Africa
Source: PLoS One. 2025 Sep 22;20(9):e0331904. doi: 10.1371/journal.pone.0331904 (PMC12453174; doi:10.1371/journal.pone.0331904)
Supplement: Attachment — Submitted filename: Responses to reviewers.docx [file pone.0331904.s008.docx]

PLOS ONE
1265 Battery Street, Suite 200
San Francisco, CA 94111
United States

9 July 2025

Dear Editors in chief

We would like to thank the editor and reviewers for the very valuable comments made. They have certainly enabled us to strengthen the quality of our paper and increase its relevance. In the present letter, we have a table showing the comments raised and our responses. Both clean and marked-up copies of the revised manuscript are attached.

Regards,

Tanyaradzwa Dube, corresponding author.

**Desk Review Comments**

| **Comment** | **Response** |
| --- | --- |
| 1. We note that this data set consists of interview transcripts. Can you please confirm that all participants gave consent for interview transcript to be published?  If they DID provide consent for these transcripts to be published, please also confirm that the transcripts do not contain any potentially identifying information (or let us know if the participants consented to having their personal details published and made publicly available). We consider the following details to be identifying information:  - Names, nicknames, and initials  - Age more specific than round numbers  - GPS coordinates, physical addresses, IP addresses, email addresses  - Information in small sample sizes (e.g. 40 students from X class in X year at X university)  - Specific dates (e.g. visit dates, interview dates)  - ID numbers | Yes, the participants consented to having their de identified data shared in published journals.  The transcripts have been checked to remove any identifying information as recommended. |

**Responses to the Editors Comments**

**Responses Reviewer 2**

| Comment | Response |
| --- | --- |
| Line 1  “*If we lose it, we are worried”*: Individual and provider level perceptions and attitudes towards weight change among people living with HIV screening for tuberculosis in routine health care settings in Gauteng Province, South Africa.  please read this title again. The grammar seems incorrect...please check!  perhaps consider : perceptions and  attitudes towards weight change among people living with HIV who undergo TB screening | Line 1, page 1  Agreed changed to  Individual and provider level perceptions towards weight change among people living with HIV who undergo TB screening in routine health care settings in Gauteng Province, South Africa. |
| Line 104 page 5  We were interested to know whether attitudes to weight and body size might affect how people responded to the questions in the WHO TB symptom screening tool and whether people with stavudine-related lipodystrophy might describe the change in body shape as weight loss.  Comment: This sentence should read “We sought to investigate whether……. | Line 103 page 5  Agreed and changed to: We sought to investigate whether attitudes to weight and body size might affect how people responded to the questions in the WHO TB symptom screening tool and whether people with stavudine-related lipodystrophy might describe the change in body shape as weight loss. |
| Line 119, page 6  Tell us something more about the Focus groups...you say they were all male, all female and mixed. did you have a target group size? How many came from the XPHACTOR study and how many were invited from the ART support group? similarly, how many staff were art of the initial group and how many were invited? how did you determine sample size per group? | Line 123-125 page 6  Adult PLHIV who were not XPHACTOR study participants were approached in-person for participation in the study via an ART support group linked to the clinic.  Lines 134 to 142  We initially planned to conduct around three FGDs with about eight to twelve participants in each group but eventually conducted seven FGDs to reach saturation. The number of discussions increased because we separated male and female FGD’s (two groups per gender) plus a mixed gender discussion to capture sufficient diversity in perspectives and experiences. Sample size per group was determined by availability of participants and previous qualitative research which recommends six to twelve participants per FGD(17,18). This range ensures sufficient diversity, a balanced interaction to allow all participants to contribute as well as effective facilitation of the discussion by the moderator.  Line 200-205 page 9  Seven FGDs (labelled 1-7) were held between 6^th^ and 21^st^ August 2014, with 7-12 participants in each focus group. The five adult PLHIV FGDs (two males, two females and one mixed gender) had 7-12 participants in each group. The HCWs and research staff FGD comprised of ten participants (seven routine HCWs and three research staff). The doctors and dieticians FGD had seven participants (five doctors and two dieticians) who were all routine staff. |
| Line 124, page 6  Adult PLHIV who were not XPHACTOR study participants were approached for participation in the study face to face via an ART support group linked to the clinic.  Comment: sentence should read as  "Adult PLHIV who were not XPHACTOR study participants were approached in-person for participation in the study via an ART support group linked to the clinic." | Line 123 page 7  Sentence changed as suggested.  Adult PLHIV who were not XPHACTOR study participants were approached in-person for participation in the study via an ART support group linked to the clinic |
| Line 135, page 6  We initially planned to conduct around three FGDs but eventually conducted seven FGDs to reach saturation  Comment:  could you expand on this ....this related to the methodological approach taken: | Line 136-142 page 7  The number of discussions increased because we separated male and female FGD’s (two groups per gender) plus a mixed gender discussion to capture sufficient diversity in perspectives and experiences. Sample size per group was determined by availability of participants and previous qualitative research which recommends six to twelve participants per FGD. This range ensures sufficient diversity, a balanced interaction to allow all participants to contribute as well as effective facilitation of the discussion by the moderator. |
| Line 161, page 8  All interviews were transcribed verbatim, and interviews done in local languages were translated to English  tell us more on this process and language translation is a complex process. one has to make sure that the nuances are not lost during the translation process!! | Line 161-171 page 8  More details have been added:  Translations and transcriptions were done by experienced researchers fluent in the study languages. Transcriptions were done verbatim to keep nuances for instance, almost all deliberations were captured including non-verbal cues such as short silences and laughs. Quality assurance was conducted whereby a second researcher checked the accuracy of the transcripts against audio recordings. |
| Line 186, Page 9  could you add a few lines on the process to reach saturation? | Line 193-196 page 9  TD, FM and JM reviewed and refined emerging themes repeatedly until saturation was achieved when no additional themes or categories could be identified. |
| Line 189, page 9  so there were main themes and sub-themes! the main themes identified are not what you are reporting in this sentence...they are sub-themes...separate them and be clear about the main and sub-themes! | Line 206-211 page 10  The main themes and sub themes were separated as following;  We identified three main themes and five sub themes. The main themes were i. perceptions and attitudes towards weight change; ii ideal body size and shape; iii negative impact of weight loss on participant’s daily life. The sub themes were i) perceptions towards weight change; ii) attitudes towards weight loss; iii) stigma and loss of social support; psychological impact of weight loss; and iv) changes in dressing. |
| Line 375, page 17  please correct the grammar...you cannot start a sentence with "because" | Line 393, page 18  Changed sentence to “As a result, the participants preferred gaining to losing weight”. |
| Line 378 page 17  Corroborating our finding, a study in Uganda found that women living with HIV did not want to lose weight because weight loss could lead to people suspecting their HIV seropositive status. Women living with HIV who lost weight in the same study also reported that they felt stigmatized, and it affected their adherence to ART[18]  This should read as "The results obtained in our study were similar to the findings of a Ugandan study that found that women living with HIV did not want to lose weight as this could lead to people suspecting that they were HIV positive. | Line 397-401, page 18  Agreed and the sentence changed as suggested.  The results obtained in our study were similar to the findings of a Ugandan study that found that women living with HIV did not want to lose weight as this could lead to people suspecting that they were HIV positive |
| Line 381 page 17  The finding that it was rare for PLHIV to seek healthcare services because of weight loss concerns may negatively affect efforts to screen for and diagnose TB early.  did your study find this or was this from the literature? | Line 401-404 page 18  This was a potential implication based on previous studies that have shown an association between loss of weight and delayed TB diagnosis/ seeking care.  The finding that it was rare for PLHIV to seek healthcare services because of weight loss concerns may negatively affect efforts to screen for and diagnose TB early. Several studies have found an association between weight loss and delay in TB diagnosis(21–23). A study consisting of 185 people with TB in KwaZulu Natal South Africa found that weight loss was associated with delayed care(21). The researchers suggested that stigma of weight loss because of its association with other diseases could have been a key factor contributing to this delay (21). |
| Line 388, page 17  A potential alternative to asking questions about weight loss when PLHIV present for treatment of check-ups is to measure their weight at the visit and compare this to previous weight measures recorded in their file. | Line 413-415 page 19  Agreed and changed as suggested. |
| Line 389, page 17  The stigma associated with HIV and TB needs to be addressed for example by educating, through community outreach campaigns, both PLHIV, family members, and the community on HIV and TB using tool kits that have been developed to address TB-related stigma.  please correct the grammar ..this sentence does not make sense! | Line 426-428 page 9  Agreed sentenced revised to:  The stigma associated with HIV and TB needs to be addressed for example by educating PLHIV, family members, and the community on HIV and TB. The education can be done through community outreach campaigns using tool kits that have been developed to address TB-related stigma |
| Line 397, page 18  A study conducted in Cape Town, South Africa found that participants preferred to be overweight and at risk of acquiring cardiovascular diseases, rather than to lose weight and be stigmatised as infected with HIV.  for better context, perhaps add a few lines about this study ...how many participants, what was the objective of this study, methodology used, etc. | Line 424-440 page 19  Done, additional was added.  A mixed method study including 513 adult women was conducted in Cape Town, South Africa to explore the perception among black South African women that people who are thin are infected with HIV or have AIDS. The study found that participants preferred to be overweight and at risk of acquiring cardiovascular diseases, rather than to lose weight and be stigmatised as infected with HIV[5] |
| Line 410, page 18  Our study found that fuller women were considered to have the ideal body shape. This finding is consistent with findings from other previous studies.  Comment  Add references: | References cited. Line 440  Matoti-Mvalo T, Puoane T. Perceptions of body size and its association with HIV/AIDS. South African Journal of Clinical Nutrition. 2011;24(1):40–5.  Nyamaruze P, Govender K. I like the way i am, but i feel like i could get a little bit bigger”: Perceptions of body image among adolescents and youth living with HIV in Durban, South Africa. PLoS One [Internet]. 2020;15(1):1–10. Available from: <http://dx.doi.org/10.1371/journal.pone.0227583> |
| Line 431, page 19  Our study findings are significant in today’s terms as TB remains a leading cause of death among PLHIV  Delete “in today’s terms” in the sentence | Deleted as suggested. Line 462  Our study findings are significant as TB remains a leading cause of death among PLHIV so understanding their perceptions around weight change and how this may affect TB symptom screening is important |
| The other concern I have is the discussion section where you compare or quote other studies ..please provide a bit more context to those studies so the reader has a sense of how similar or different the settings, context, methods, etc were. | Additional information has been added to the studies cited in the discussion.  Lines 397, 404, 424, 436, 441, 458 |
| there is an unacceptable high number of grammatical and language errors! This is a scientific journal and these issues must be addressed. For example, you cannot start a sentence with "Because" or say "In today's terms...." | Incorrect grammar has been revised in the manuscript  Lines 89, 104, 144, 168, 216, 261, 264, 298, 358, 393, 406, 454, 457, 458, 484 |
